# Exploring prognostic and immunological characteristics of pancreatic ductal adenocarcinoma through comprehensive genomic analysis of tertiary lymphoid structures and CD8 + T-cells

**DOI:** 10.1007/s00432-024-05824-0

**Published:** 2024-06-08

**Authors:** Hao Hu, Yang Xu, Qiang Zhang, Xiangnan Ai, Tengfei Wang, Huixing Li, Changguo Jin, Caiguo Ouyang, Zhenyu Wu

**Affiliations:** Department of Hepatobiliary Surgery, Aerospace Center Hospital, No. 15, Yuquan Road, Haidian District, Beijing, 100049 China

**Keywords:** Single-cell, Pancreatic ductal adenocarcinoma, Tertiary lymphoid structure, CD8 + T-cell, Immunological characteristics

## Abstract

**Purpose:**

Tertiary lymphoid structures (TLSs) and CD8 + T-cells are potential prognostic indicators for pancreatic ductal adenocarcinoma (PDAC). We established a novel scoring system for evaluating the risk for PDAC based on TLS- and CD8 + T-cell-related genes.

**Methods:**

We analyzed single-cell sequence data from PDAC patients in the Genome Sequence Archive. Bioinformatics and machine algorithms established and validated a scoring method (T-C score) based on PDAC survival-related genes highly expressed in TLSs and CD8 + T-cells. Patients were stratified into the low- and high-T-C score groups. Differences in survival, pathway enrichment, mutation status, immune cell infiltration, expression of immune checkpoint-associated genes, tumor stemness, and response to antitumor therapy were compared through computer simulation methods.

**Results:**

Overall survival differed significantly between the training and validation cohorts’ low- and high-T-C score groups. The low-T-C score group correlated with lower tumor mutation burden and lower levels of tumor stemness compared with the high-T-C score group. Patients with lower T-C scores exhibited advantages in immunotherapeutic responses and might be more sensitive to the chemotherapeutic regimen and multi-kinase inhibitors.

**Conclusion:**

The T-C score could serve as an effective model for predicting the survival and therapeutic responses of patients with PDAC.

**Supplementary Information:**

The online version contains supplementary material available at 10.1007/s00432-024-05824-0.

## Introduction

Neoadjuvant and adjuvant treatments, including chemo-, radio-, and immunotherapy, achieve limited success due to the heterogeneity of the pancreatic ductal adenocarcinoma (PDAC) tumor microenvironment (TME). CD8 + T-cells are the main component of tumor-infiltrating lymphocytes in the TME of PDAC; they harbor immune-monitoring functions and can detect antigens secreted by malignant cells (Fitzgerald et al. 2021; Pearce et al. 2023). Usually, a high level of tumor-infiltrated CD8 + T-cells indicates a good prognosis, which is also a key factor in immune checkpoint blockade (ICB) therapy (Gubin et al. 2014; Sade-Feldman et al. 2019). Tumor mutational burden (TMB) is crucial for the response to ICB in lung cancer and melanoma (Samstein et al. 2019). However, due to the low TMB in PDAC, few tumor-related antigens are released into the TME, resulting in low levels of CD8 + T-cell infiltration.

In addition to T-cells and macrophages, the main component of the adaptive immune system consists of B-cells. In tumors, B-cells take part in the formation of a distinct tumor immune microenvironmental (TIME) feature, the tertiary lymphoid structures (TLSs). TLSs are ectopic lymphoid organs with very similar structure and development to lymph nodes, and mature TLSs are composed of germinal B-cell centers and dendritic cells surrounded by a rim of T-cells (Workel et al. 2019). TLSs usually occur at sites of chronic inflammation as well as various cancer types, including pancreatic cancer (Gunderson et al. 2021). However, unlike primary and secondary lymphoid structures, antigenic stimulation is crucial during the development of TLSs, and the formation of TLSs represents an ongoing adaptive immune response (Lutz et al. 2014). Tumor-associated TLSs, which are usually correlated with a good prognosis, are also thought to be a site for immune cell recruitment and activation (Helmink et al. 2020). The presence of TLSs is closely related to the ICB response in melanomas, sarcomas, and urothelial cancers, and TLS infiltration can be increased by ICB treatment (Cabrita et al. 2020; Helmink et al. 2020; Petitprez et al. 2020). In addition, the formation of TLSs is relevant to the CD8 + T-cell infiltration; TLSs with significant plasma cell infiltration are correlated with improved CD8 + T-cell infiltration and overall survival (OS) in ovarian cancer (Kroeger et al. 2016). Recently, a positive correlation between tumor-infiltrating CD8 + T-cells and TLSs in PDAC was shown by Takeshi Tanaka et al. (Tanaka et al. 2023). However, the relationship between the degree of TLSs and CD8 + T-cell infiltration and the ICB therapeutic responses in PDAC remains unclear. In addition, whether high TLSs and CD8 + T-cell infiltration contribute to the OS and therapeutic response in patients with PDAC and the biological mechanism behind them are still poorly illustrated.

In this study, we aimed to establish a novel scoring system for evaluating the risk of PDAC based on TLSs- and CD8 + T-cell-related genes. To do this, we characterized TLSs and CD8 + T-cell-related gene clusters to identify the potential biological functions of TLSs and CD8 + T-cells in PDAC. Firstly, weighted gene co-expression network analysis (WGCNA) was performed to identify CD8 + T-cell-associated genes in PDAC. Secondly, unsupervised cluster analysis was carried out to identify two distinct TLSs and CD8 + T-cells related subtypes. Furthermore, the TLSs-CD8 + T-cell (T-C) score was developed and then tested for its ability to predict survival and responses of patients to chemotherapeutic agents and ICBs. This scoring system can potentially serve as an effective model for predicting the survival and therapeutic responses of patients with PDAC.

## Materials and methods

A preprint has previously been published (Hu et al. 2023). The procedures were described in the Supplementary File in detail.

## Results

### Identification of survival-related TLSAGs and CTCRGs (T-C genes)

**Fig. 1:**
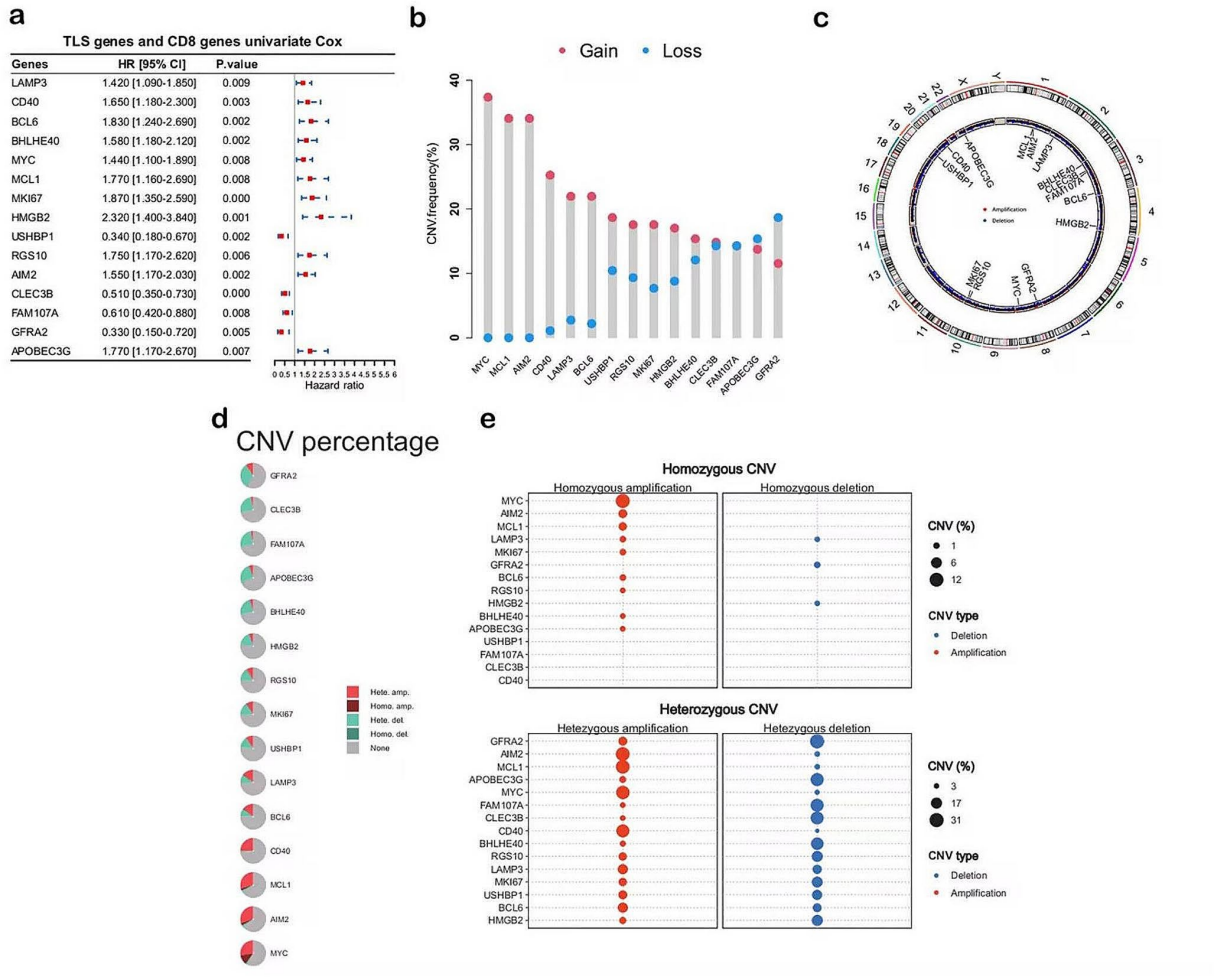
Identification of T-C genes and genetic mutational landscape of the T-C genes in PDAC. (**a**) Eight TLSAGs and seven CTCRGs were recognized by the univariate Cox analysis as T-C genes with *p* < 0.01, forest plot showing the results of the univariate Cox regression analysis. (**b**) Frequencies of CNV gain, loss, and non-CNV among T-C genes. (**c**) Circus plots of chromosome distributions of T-C genes. (**d**-**e**) CNV of T-C genes in TCGA-PAAD from the GSCA Lite database, including heterozygous and homozygous CNV. T-C genes, TLSs- and CD8 + genes; TLSAGs, tertiary lymphoid structure associated genes; CTCRGs, CD8 + T Cell Related Genes; CNV, copy number variation

CTCRGs in PDAC were identified (please see the Supplementary File, Figures S1-S3). Tumor-infiltrating CD8 + T-cells and TLSs are positively correlated in PDAC [15]. So, it is likely that the degree of TLSs, together with CD8 + T-cell infiltration, relate to the overall ICB therapeutic responses in PDAC. However, this has not yet been established. Therefore, for this study, we identified TLSAGs and CTCRGs related to survival. As we described before, 37 TLSAGs were obtained from previously published articles (Table S1), and 31 of these TLSAGs were commonly expressed across databases (the median expression levels of these genes are also displayed in Table S1). These 31 TLSAGs were combined with the previously obtained CTCRGs for univariate Cox regression analysis. As a result, eight TLSAGs and seven CTCRGs with *p* < 0.01 were significantly related to survival, so we named these genes T-C genes. The univariate Cox analysis of the T-C genes showed that four genes were protective factors with HR < 1, and 11 genes were risk factors with HR > 1 for PDAC prognosis (Fig. 1a). In addition, we detected widespread CNVs in the T-C genes. Copy number gains were most frequent, and MYC, MCL1, AIM2, CD40, LAMP3, BCL6, USHBP1, RGS10, MKI67, HMGB2, and BHLHE40 showed extensive CNVs in amplifications, whereas FAM107A, APOBEC3G, and GFRA2 showed copy number losses (Fig. 1b). The chromosomal locations of the T-C genes with CNVs are shown in Fig. 1c. Through the GSCA Lite database, we found the most frequent CNVs associated with the T-C genes were heterozygous. MYC, AIM2, MCL1, and CD40 were characterized by high amplification, while GFRA2, CLEC3B, FAM107A, APOBEC3G harbored more deletions (Fig. 1d-e).

### Identification of TLS and CD8 + T cell related subtypes (T-C cluster) in PDAC

**Fig. 2:**
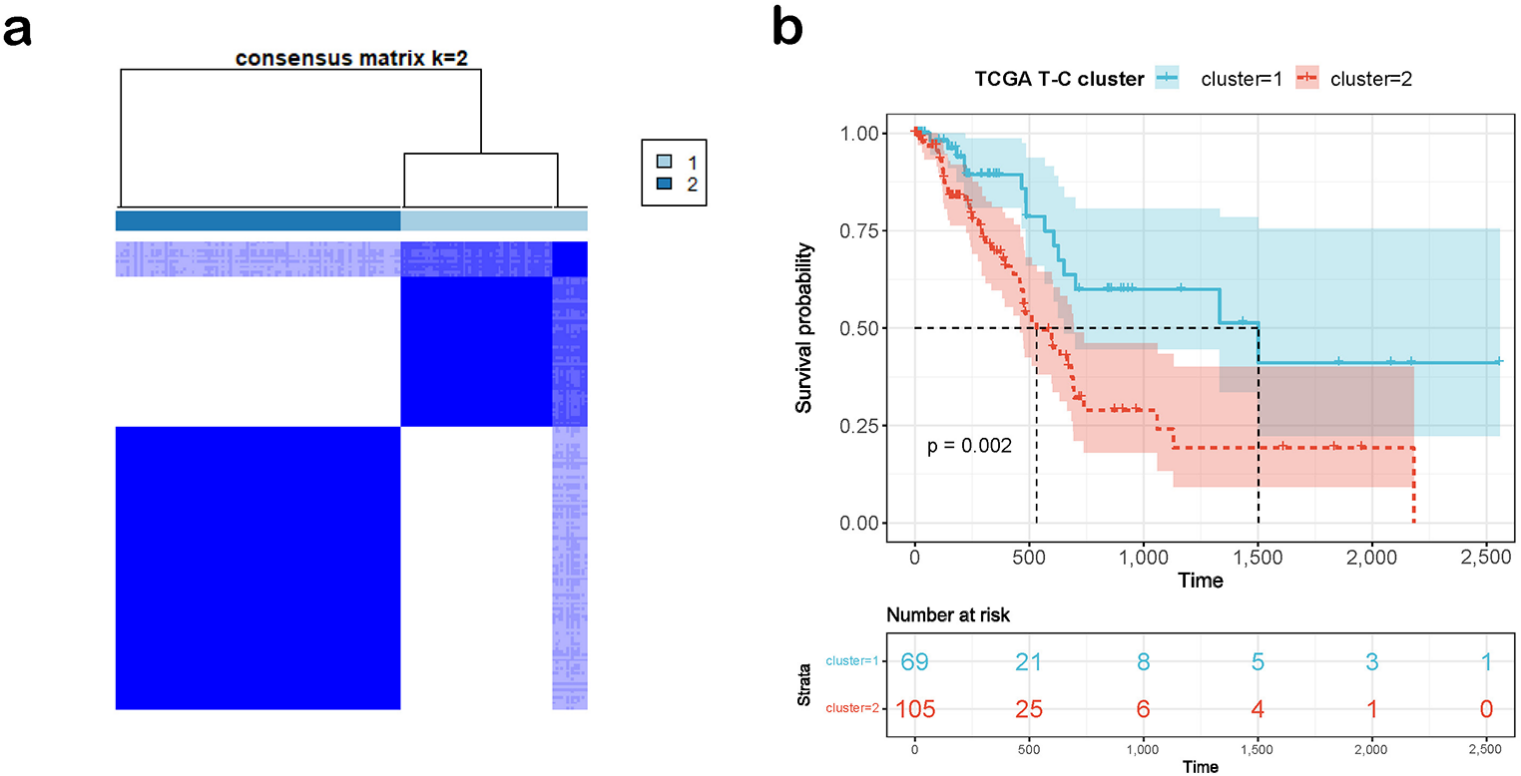
Stratifying the PDAC patients in the TCGA-PAAD cohort according to the expression profile of T-C genes and the mutational landscape between T-C clusters. (**a**) Clustering heatmap of the T-C genes in the TCGA-PAAD cohort (*n* = 174). (**b**) Survival analysis of the two T-C clusters. (*p < 0.05; **p < 0.01; ***p < 0.001; ****p < 0.0001; Ns, not significant). TMB, tumor mutation burden

To explore the relationship between TLSs, CD8 + T Cell, and tumorigenesis, we conducted unsupervised clustering upon the 174 PDAC patients of TCGA-PAAD samples based on the mRNA expression level of the 15 T-C genes. We tried k from 2 to 5, and k = 2 produced the best results in terms of clustering and OS analysis (Fig. 2a, Figure S4a). The two subgroups were designated as T-C cluster-1 and T-C cluster-2, and the patients in T-C cluster-1 had significantly better survival (*p* = 0.002, Fig. 2b). Furthermore, different TME and mutational features were observed in the two clusters (Supplementary File, Figure S5).

### Construction of the T-C score

**Fig. 3:**
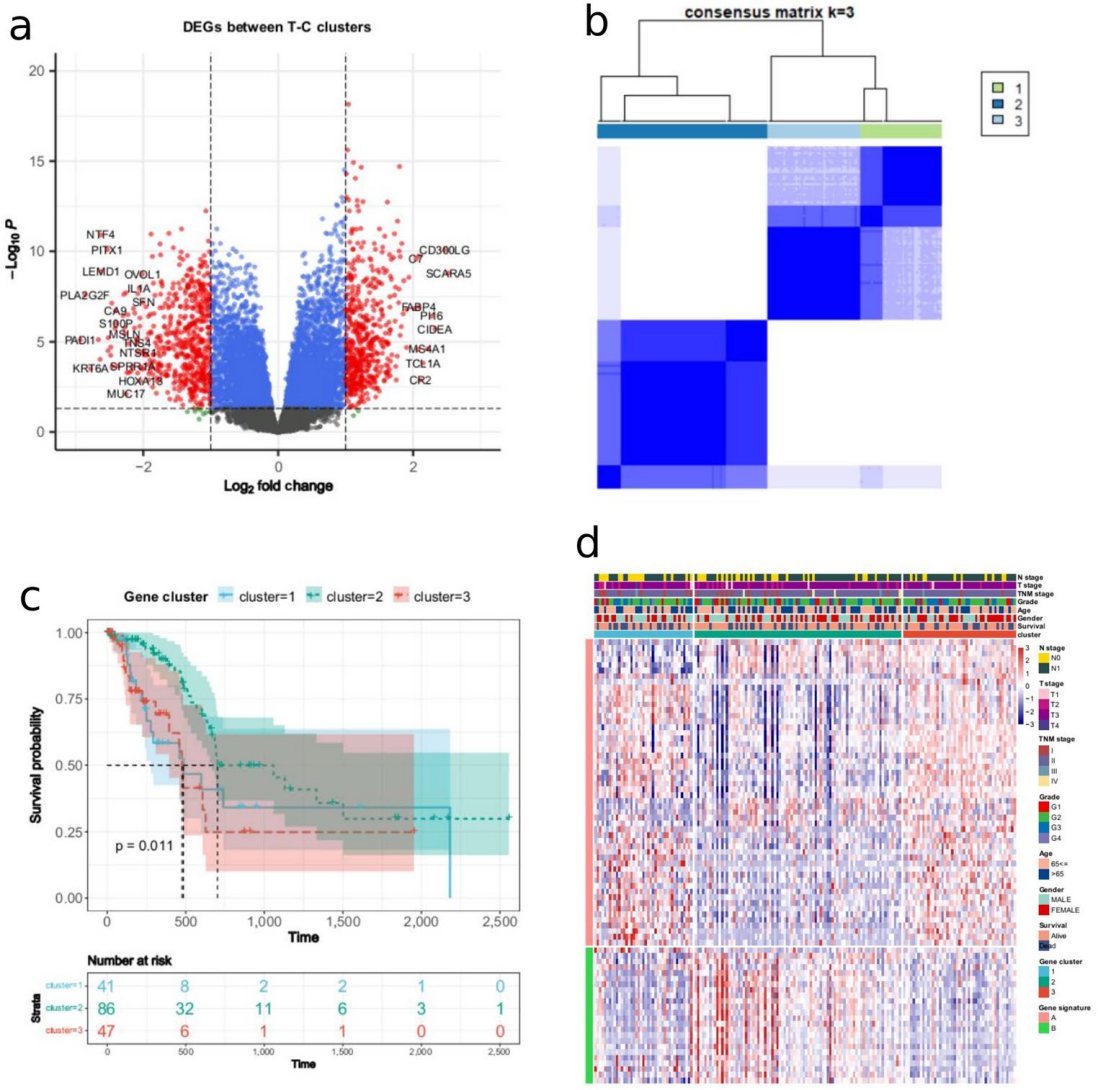
The construction of the T-C score. (**a**) The volcano map shows the differentially expressed genes (DEGs) between the two T-C clusters. (**b**) Consensus matrix heatmap defining three gene clusters according to the DEGs. (**c**) Survival analysis of the PAAD patients in three gene clusters (log-rank *p* = 0.011). (**d**) Unsupervised clustering of the DEGs further stratified PAAD patients into three gene clusters. Heat map showing the clinical features of the three gene cluster

**Fig. 4:**
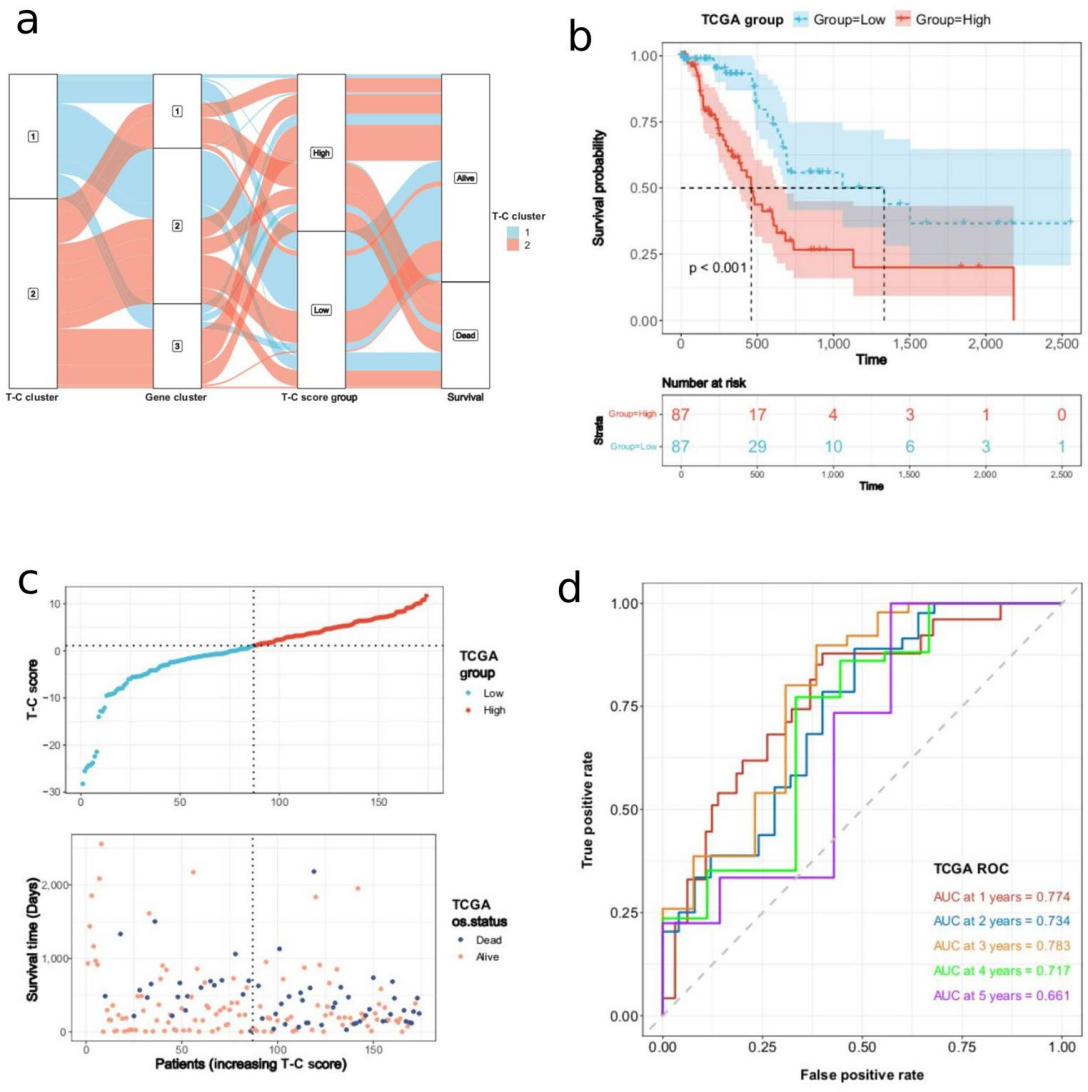
The construction of the T-C score. (**a**) A Sankey plot was used to reveal the correlation between T-C clusters, gene clusters, T-C score groups, and survival of the PDAC patients in the TCGA-PAAD cohort. (**b**) Survival analysis of the PDAC patients of TCGA-PAAD in Low and High T-C score groups (log-rank *p* < 0.001). (**c**) Distribution of the T-C score and survival status of PDAC patients. (**d**) ROC curves for the 1-, 2-, 3-, 4-, and 5-year survival times based on the T-C score

As we had established two patient clusters with differences in survival, we could then use this information to fully investigate DEGs between the clusters and to identify genes critical for the prognosis of patients with PDAC. Based on the ‘limma’ package, differential expression analysis between the two clusters was performed, and 1049 DEGs were identified (Fig. 3a). According to these DEGs, the unsupervised clustering method was utilized to divide the TCGA-PAAD cohort into three gene clusters with distinct survival (Fig. 3b). The Kaplan-Meier analysis revealed that compared with other gene clusters, the patients in gene cluster-2 had significantly longer OS (log-rank *p* = 0.011). In contrast, the patients in gene clusters-1 and − 3 had relatively poor prognoses (Fig. 3c). Based on the T-C gene signatures A and B (Table S2), the dimension reduction was performed by the Boruta algorithm. The transcriptomic profile of the 78 most abundant DEGs identified among the gene clusters was displayed as a heatmap (Fig. 3d). Based on the 78 most abundant DEGs, we calculated the T-C score for each PDAC sample in the TCGA-PAAD cohort using PCA (Table S3). Then, the PDAC samples were stratified into high or low T-C score groups based on the median T-C score of 1.121. Figure 4a depicts the distribution of samples among T-C clusters, gene clusters, and T-C score groups. Compared with the patients in the low T-C score group, those with high T-C scores indicated a poorer prognosis (log-rank *p* < 0.001, Fig. 4b). The scatter plot depicted the survival status of individual patients and the T-C score distribution (Fig. 4c). The AUC values for predicting 1-year, 2-year, 3-year, 4-year, and 5-year survival were 0.774, 0.734, 0.783, 0.717, and 0.661, respectively (Fig. 4d).

### Prognostic value of the T-C score for PDAC patients

**Fig. 5:**
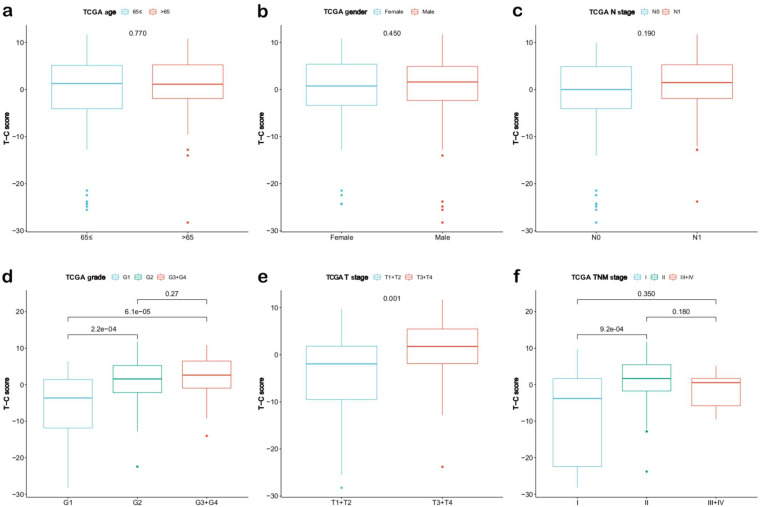
The prognostic value of the T-C score in PDAC. Comparison of the T-C score among clinical features including age (**a**), gender (**b**), N stage (**c**), histological grade (**d**), T stage (**e**), and TNM stage (**f**). OS, overall survival; AUC, area under curve

**Fig. 6:**
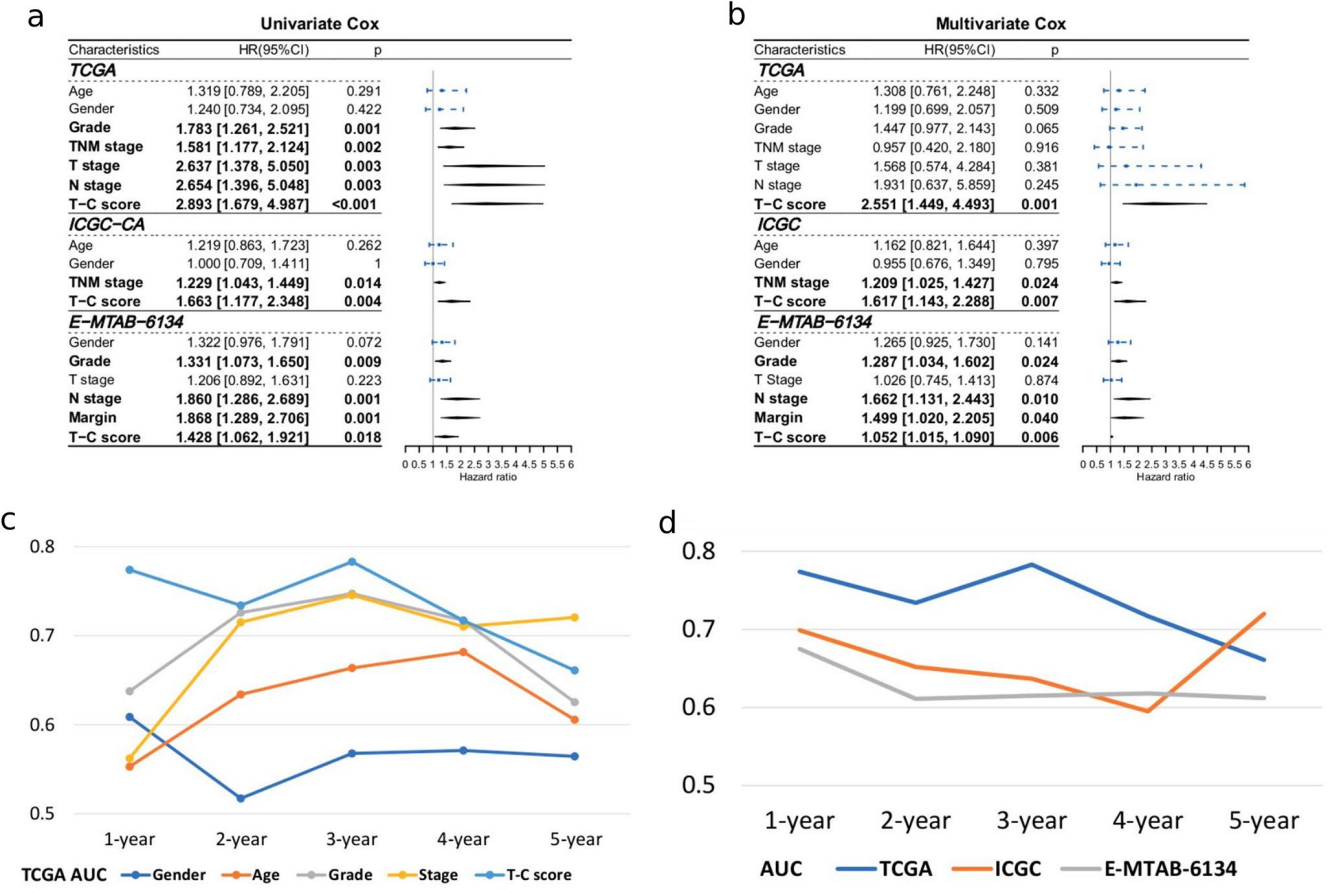
The prognostic value of the T-C score in PDAC. Both univariate (**a**) and multivariate (**b**) Cox regression analyses suggested that the T-C score was a significant prognostic factor for OS in the training (TCGA-PAAD) and validation (ICGC-CA and E-MTAB-6134) cohorts. (**c**) The time-dependent AUC values of gender, age, histological grade, TNM stage, and T-C score for the OS prediction in the TCGA-PAAD cohort. (**d**) The time-dependent AUC values of the T-C score for the OS prediction in the TCGA-PAAD, ICGC-CA, and E-MTAB-6134 cohort. OS, overall survival; AUC, area under curve

Next, we validated the prognostic value of the T-C score. The relationship between clinical features and T-C score in the TCGA-PAAD cohort is depicted in Fig. 5a-f. No significant differences were observed in age, gender, and N stage (Fig. 5a-c). At the same time, PDAC, which was better differentiated, smaller in tumor size, or early in the TNM stage, had a significantly smaller T-C score (Fig. 5d-f). Then, the univariate and multivariate Cox regression analyses were performed in the TCGA-PAAD cohort (Fig. 6a-b), demonstrating that the T-C score was an independent risk factor for PDAC. According to time-dependent AUC values, the T-C score displayed good predictive ability for OS in comparison with age, gender, differentiation grade, or stage in the TCGA-PAAD cohort (Fig. 6c). Furthermore, we calculated T-C scores for our validation cohort, including ICGC-CA, E-MTAB-6134, and the joint cohort of GSE71729 and GSE85916, and in each cohort, the median T-C score was used to stratify patients into the high or low T-C score groups. Then Kaplan-Meier analyses (Figure S4c-e) and univariate and multivariate Cox regression analyses were carried out (Fig. 4g-h), and the same trend as in the TCGA cohort was found throughout all validation cohorts. The survival status of individual patients and the distribution of the T-C score for each validation cohort are displayed in Figure S4f-h. ROC curves for each validation set are depicted in Figure S4i-k, and Fig. 6d shows the comparison of AUC values for the TCGA-PAAD, ICGC-CA, and E-MTAB-6134 cohorts.

### Functional enrichment analysis between the T-C score groups

Additionally, GSVA analysis was performed to elucidate the difference in bioinformatic functions between the T-C score groups. As depicted by the bar plot, the high T-C score group showed significant enrichment in tumorigenesis-related pathways, such as KEGG_P53_SIGNALING_PATHWAY, KEGG_WNT_SIGNALING_PATHWAY, KEGG_NOTCH_SIGNALING_PATHWAY, KEGG_CELL_CYCLE, KEGG_HOMOLOGOUS_RECOMBINATION, KEGG_TIGHT_JUNCTION, KEGG_THYROID_CANCER, KEGG_SMALL_CELL_LUNG_CANCER, KEGG_BLADDER_CANCER, KEGG_PANCREATIC_CANCER. In contrast, the low T-C score group was mainly enriched in pathways related to benign diseases (Figure S6a).

### The correlation between the T-C score and TIME

To further illuminate the intrinsic immune status that contributed to the distinct survival pattern, the correlation between the T-C score and the PDAC TIME was investigated. The ESTIMATE algorithm was applied to the TCGA-PAAD cohort, and the correlation analyses indicated that the T-C score was significantly and negatively correlated with the ESTIMATE score (*R*=-0.23, *p* = 0.002; tau=-0.170, *p* = 8.95e-04), immune score (*R*=-0.24, *p* = 0.002; tau=-0.171, *p* = 8.31e-04), and stromal scores (*R*=-0.19, *p* = 0.01; tau=-0.144, *p* = 4.86e-03) (Figure S6b). Meanwhile, the low T-C score group demonstrated significantly higher levels of ESTIMATE score, immune score, and stromal score (Figure S6b). Furthermore, the relationship between tumor-infiltrated immune cells and the T-C score was evaluated. The ssGSEA heatmap (Figure S6c) showing the infiltration of 28 immune cell types indicated the low T-C score group was more immune infiltrated compared to the high T-C score group. In the boxplot (Figure S7a), the low T-C score group had significantly higher levels of infiltration in 14 out of 16 immune cell types that were differently infiltrated between the groups. The CIBERSORTx web tool was also used to predict immune status between the T-C score groups, which exhibited significantly escalated infiltration of naive B-cells, CD8 + T-cells, and monocytes. It activated NK cells in the low T-C score group (Figure S6d). Similar results could be found in immune analyses using xCELL, EPIC, and MCPCounter algorithms (Figure S7b-d), which indicated that the low T-C score group could be an immune-active phenotype. The higher levels of B-cells and monocytes may also indicate a stronger signal for TLS infiltration. Moreover, the expression levels of the ICPs-related genes also showed significant differences between the two groups. Among the 28 differentially expressed ICPs-related genes, 21 genes were significantly upregulated in the low T-C score group (Figure S6e), which suggested that the low T-C score group might benefit more from ICB.

### Relationship between T-C score and PDAC mutation burdens

Given the tight correlation between genomic alterations and tumor immunity, we implemented somatic mutation and CNV analyses to investigate the genomic alterations between T-C score groups. The top 25 genes with the most frequent alteration between T-C score groups in the TCGA cohort were visualized in oncoplots (Figure S8-a, b). The patients in the high T-C score group exhibited a higher frequency of genomic alterations in general (98.81% in the high T-C score group vs. 63.51% in the low T-C score group). As the most mutated genes in PDAC, the mutation frequencies of KRAS and TP53 in the high T-C score group were 88% and 75%, respectively, which were remarkably higher than those in the low T-C score group (31% and 41%, respectively) (Figure S8c). The alteration frequencies of RNF213 and ADGRL2 also significantly differed between the high and low T-C score groups (Figure S8c). Furthermore, extensive co-occurrence was found among the top 25 mutated genes, including between TP53 and KRAS, CDKN2A and KRAS, CDKN2A and TP53, CACNA1B and SMAD4, RREB1 and TTN, RREB1 and MUC16, ADGRL2 and RNF213, PCDH9 and ADAMTS16 (Figure S8d). Positive correlations between T-C score and amplifications (*R* = 0.27, *p* < 0.001; tau = 0.189, *p* = 2.54e-04), deletions (*R* = 0.47, *p* < 0.001; tau = 0.331, *p* = 2.22e-16), and tumor mutation burden (TMB) (*R* = 0.41, *p* < 0.001; tau = 0.276, *p* = 3.58e-07) were discovered in the TCGA cohort in CNV analysis (Figure S8e-g). Moreover, patients in the high T-C score group exhibited significantly higher gene alterations involved in amplifications, deletions, and TMB (Figure S9a-c). The results demonstrated that patients with high T-C scores had worse survival, which coincided with the notion that the higher mutation accumulation in cancer negatively correlated with the OS of patients (Pleasance et al. 2020). Furthermore, the T-C score is related to PDAC tumor stemness (Supplementary File, Figure S10), and relationships were observed between the T-C score and the sensitivity to anti-cancer treatments (Supplementary File, Figure S11).

## Discussion

In this study, we demonstrated the presence of TLSs was coupled with higher infiltration of tumor-associated CD8 + T cells from a single-cell perspective and then established a scoring method (T-C score) based on the consensus clustering of the survival-related TLSs- and CD8 + T-cell-associated gene expression profiles in PDAC patients.

Previous studies mainly focused on the role of T cells, highlighting the association of tumor-infiltrating T lymphocytes with patients’ prognosis in numerous tumors, including PDAC (Balachandran et al. 2017; Carstens et al. 2017; Fridman et al. 2017; Germain et al. 2014; Rouanne et al. 2021; Sautes-Fridman et al. 2016; Wirsing et al. 2014). However, recently, B-cells and TLSs were also discovered to be critical components in the TIME in terms of patient prognosis and response to immunotherapy (Cabrita et al. 2020; Germain et al. 2014; Helmink et al. 2020; Petitprez et al. 2020; Thommen et al. 2018). Inducing the production of TLSs has been suggested as a new direction for individualized immunotherapy for patients with pancreatic cancer (Zhou et al. 2021). TLSs represent a special TIME status and serve as a beacon for antigen presentation and T-cell activation. Previous publications also demonstrated that different types of TLSs may exist in individual tumors independent of the spatial location of the TLS (Cabrita et al. 2020; Horeweg et al. 2022). In this study, the investigation of 31 TLSAGs identified eight significantly related to survival and included in the T-C score, supporting the previous research to some degree.

This study identified 638 CTCTGs closely correlated with CD8 + T cell abundance in PDAC samples. A close relationship between CD8 + T-cells and TLSs is found in many cancers. Cabrita et al. (Cabrita et al. 2020) found that the presence of TLSs was coupled with increased tumor-associated CD8 + T-cell infiltration in melanoma, and the combination of infiltration of both TLSs and CD8 + T-cells was associated with better survival outcomes compared to CD8 + T-cells infiltration alone. A positive correlation between CD8 + T-cell infiltration and TLS presence was also observed in PDAC by Gunderson et al. (Gunderson et al. 2021).

Although the roles of TLSs and CD8 + T-cells in tumor biology and antitumor immunity have been widely studied, the potential clinical translation is still hampered by the heterogeneity of TLSs and their complex relationship with CD8 + T-cells. By focusing on developing a scoring method that utilized the gene signatures of both TLSs and CD8 + T-cells in PDAC, we aimed to better stratify pancreatic cancer. In this study, the survival-associated TLSAGs and CTCRGs were selected by univariate Cox analysis, and based on that, PDAC samples were initially stratified into two T-C clusters based on consensus clustering. The patients in T-C cluster-1 had relatively better survival and lower TMB compared to T-C cluster-2 patients, which was consistent with the notion that mutation accumulation in cancer has a negative correlation with the OS of patients (Pleasance et al. 2020). The T-C cluster-1 also had higher TLSAG and CTCRG expression and higher immune cell infiltration than T-C cluster-2. These findings together suggested the TLSs and CD8 + T-cells might act synergistically to promote immune infiltration and patient survival. Therefore, an integrative characterization of the TLSs and CD8 + T-cells at the gene level would be a better approach to stratify PDAC patients for further individualized assessment and therapy.

The current study established the T-C score by consensus clustering and the Boruta algorithm. Patients with lower T-C scores in TCGA-PAAD showed better survival, suggesting that the T-C score could be a prognostic indicator. The predictive ability of the T-C score in PDAC for 1-, 2-, 3-, 4-, and 5-year survival was confirmed by the ROC curves. Further, the T-C score was verified as an independent risk factor for OS through the univariate and multivariate Cox regression analyses. The good predictive ability of the T-C score was further validated in the ICGC- CA, E-MTAB-6134, and the joint cohort of GSE71729 and GSE85916 via K-M analyses, time-dependent AUCs, univariate, and multivariate Cox regression analyses. Collectively, our current study confirmed the good predictive ability of the T-C score, which suggested the T-C score could be used in clinical practice to predict OS for PDAC patients. PDAC is a genetic disease. The most prevalent form of genetic alteration in PDAC is single-nucleotide variants (SNVs) (Connor et al. 2019; Notta et al. 2016). KRAS, TP53, CDKN2A, and SMAD4 are among the earliest genetic variants in PDAC (Bazzichetto et al. 2020). In the current research, though not all significant, higher mutation frequencies in KRAS (88% vs. 31%), TP53 (75% vs. 41%), CDKN2A (24% vs. 12%), and SMAD4 (26% vs. 19%) were found in the high T-C score group, the mutation analysis revealed a positive correlation between the TMB and T-C score, as well as between CNV and T-C score. Notably, most patients in the T-C cluster-2 were in the high T-C score group. These findings suggested that gene mutations in PDAC might drive the profile of TLSs and CD8 + T-cell infiltration toward a pattern of high T-C score in PDAC. Stem-like cell phenotypes are closely related to many key steps in PDAC progression and the cancer metastatic cascade, as cancer cells expressing those features exhibit increased cell motility and invasiveness (Yang et al. 2022; ZhangLiu et al. 2022; Zhou et al. 2022). Therefore, we estimated PDAC stemness using the OCLR algorithm. Higher levels of stemness score were found in the high T-C score group, and a positive correlation was found between T-C score and stemness score, indicating more mutations in genes that encode oncogenes and epigenetic modifiers, frequent perturbations in specific mRNA/miRNA transcriptional networks, and more deregulation of signaling pathways for PDAC patients in the high T-C score group (Malta et al. 2018).

The TIME is closely related to the clinical prognosis of patients with tumors (Chen et al. 2020). Different types of immune cell populations, such as lymphocytes and myeloid-originated cells, comprise a major part of the TIME (Zhang et al. 2020). Through ssGSEA and CIBERSORTx, we recognized that the low T-C score group was a more immune-activated phenotype with significantly higher infiltrations of naïve B cells, CD8 + T cells, activated NK cells, and monocytes. In contrast, the high T-C score group was heavily infiltrated by M0 macrophages, which was found to be an independent risk factor for various cancers (Farha et al. 2020; Huang et al. 2020; Jairath et al. 2020; ZhangZou et al. 2022). Recent advances have indicated that the active pre-treatment immune status could positively impact immunotherapy (Chabanon et al. 2021; D’Alise et al. 2021). More tumor-infiltrating CD8 + T cells in the low T-C score group would be expected to have a marked influence on immunotherapy, as the classical mechanism behind the anti-PD-1/PD-L1 treatment is that the mutual suppression effect conducted by tumor cells and tumor-infiltrating CD8 + cytotoxic T cells (CTLs) could be abolished by ICBs. So, CD8 + CTLs could regain their cytotoxicity against tumor cells (Ishii et al. 2020). In light of the previously reported capability of TIME status in predicting sensitivity to ICB treatment in patients with cancer (Ouyang et al. 2021), we analyzed the ICP gene expression, and a higher level was found in the low T-C score group. In accordance with this result, a significantly higher response rate to ICBs was observed in the same group in TIDE analysis. Though classically, a high TMB has been regarded as a predictive factor for the response to ICB treatment (Chen et al. 2019; Cristescu et al. 2018), a different result was recently published regarding all solid cancer types (McGrail et al. 2021). Thus, the T-C score may be an alternative marker for predicting therapeutic response to ICBs. Emerging evidence has exhibited that multi-kinase inhibitors alone or synergistically used with HDAC inhibitors could suppress PDAC tumor growth (Dent et al. 2021; Yu et al. 2022). In the current study, the IC50 and CMap analysis demonstrated a possible higher sensitivity for using multi-kinase and HDAC inhibitors in the low-risk group, which provided another therapeutic possibility for these PDAC patients.

This study had limitations. Bioinformatics-based investigations are subjected to the limitations of the algorithms used. A batch effect may occur even after correction due to different RNA sequencing methods and platforms. The ICB therapeutic response was predicted, and the clinical data in the ICGC-CA, E-MTAB-6134, and TCGA-PAAD cohorts did not exactly match, which may limit the validation process. Large-scale prospective studies are required to verify the value of the T-C score in predicting clinical benefits for PDAC patients.

Nevertheless, this study proposes a novel score for the prognosis of PDAC. Part of the score can be obtained from the pathological examination of the surgical specimen. The other part of the score relies upon advanced gene amplification and quantification single-cell techniques. We agree that such techniques might not be accessible to all hospitals yet, but such technologies are becoming more reliable and available and less expensive. Hence, this score could be determined by any laboratory possessing adequate equipment and could contribute to improving the prognosis of PDAC.

## Conclusion

In summary, we integrated transcriptome data and the expression of TLSs- and CD8 + T-cell-related genes to establish a new independent prognostic marker for PDAC, the T-C score. This score could be used for the prediction of OS, immune infiltration status, tumor stemness, tumor mutation, and sensitivity to immune therapy and chemotherapeutics for PDAC. Therefore, the T-C score may assist with diagnosing and treating patients with PDAC.

## Electronic supplementary material

Below is the link to the electronic supplementary material.

Supplementary Material 1

Supplementary Material 2

Supplementary Material 3

Supplementary Material 4

Supplementary Material 5

Supplementary Material 6

Supplementary Material 7

Supplementary Material 8

Supplementary Material 9

Supplementary Material 10

Supplementary Material 11

Supplementary Material 12

Supplementary Material 13

Supplementary Material 14

Supplementary Material 15

Supplementary Material 16

Supplementary Material 17

## Data Availability

Publicly available datasets were analyzed in this study: The raw expression data for scRNA-seq analysis of tumor samples from 24 PDAC patients was downloaded from the GSA (Genome Sequence Archive) database (GSA: https://bigd.big.ac.cn/gsa), under accession number CRA001160. The STAR raw counts data, the corresponding information of somatic mutations in TCGA-PAAD patients, and the related clinical information of PDAC patients were extracted from the PAAD cohort in The Cancer Genome Atlas (TCGA) database (https://portal.gdc.cancer.gov/projects/TCGA-PAAD/). The raw counts data and the corresponding clinical data for the ICGC-CA cohort were obtained from the International Cancer Genome Consortium (ICGC) database (ICGC-CA, https://dcc.icgc.org/releases/current/Projects/PACA-CA). The raw data of the E-MTAB-6134 cohort were downloaded from the ArrayExpress database (https://www.ebi.ac.uk/arrayexpress/experiments/E-MTAB-6134). The expression data of PDAC cases in GSE71729 and GSE85916 were downloaded from Gene Expression Omnibus (GEO) database (GSE71729, https://www.ncbi.nlm.nih.gov/geo/query/acc.cgi?acc=GSE71729/; GSE85916, https://www.ncbi.nlm.nih.gov/geo/query/acc.cgi?acc=GSE85916/).

## References

[CR1] Balachandran VP, Luksza M, Zhao JN et al (2017) Identification of unique neoantigen qualities in long-term survivors of pancreatic cancer. Nature 551:512–516. 10.1038/nature24462 29132146 10.1038/nature24462 PMC6145146

[CR2] Bazzichetto C, Luchini C, Conciatori F et al (2020) Morphologic and molecular Landscape of Pancreatic Cancer variants as the basis of new therapeutic strategies for Precision Oncology. Int J Mol Sci 21. 10.3390/ijms21228841 10.3390/ijms21228841 PMC7700259 33266496

[CR3] Cabrita R, Lauss M, Sanna A et al (2020) Tertiary lymphoid structures improve immunotherapy and survival in melanoma. Nature 577:561–565. 10.1038/s41586-019-1914-8 31942071 10.1038/s41586-019-1914-8

[CR4] Carstens JL, Correa de Sampaio P, Yang D et al (2017) Spatial computation of intratumoral T cells correlates with survival of patients with pancreatic cancer. Nat Commun 8:15095. 10.1038/ncomms15095 28447602 10.1038/ncomms15095 PMC5414182

[CR5] Chabanon RM, Rouanne M, Lord CJ et al (2021) Targeting the DNA damage response in immuno-oncology: developments and opportunities. Nat Rev Cancer 21:701–717. 10.1038/s41568-021-00386-6 34376827 10.1038/s41568-021-00386-6

[CR6] Chen YP, Wang YQ, Lv JW et al (2019) Identification and validation of novel microenvironment-based immune molecular subgroups of head and neck squamous cell carcinoma: implications for immunotherapy. Ann Oncol 30:68–75. 10.1093/annonc/mdy470 30407504 10.1093/annonc/mdy470

[CR7] Chen Z, Zhou L, Liu L et al (2020) Single-cell RNA sequencing highlights the role of inflammatory cancer-associated fibroblasts in bladder urothelial carcinoma. Nat Commun 11:5077. 10.1038/s41467-020-18916-5 33033240 10.1038/s41467-020-18916-5 PMC7545162

[CR8] Connor AA, Denroche RE, Jang GH et al (2019) Integration of genomic and transcriptional features in pancreatic Cancer reveals increased cell cycle progression in Metastases. Cancer Cell 35:267–282e267. 10.1016/j.ccell.2018.12.010 30686769 10.1016/j.ccell.2018.12.010 PMC6398439

[CR9] Cristescu R, Mogg R, Ayers M et al (2018) Pan-tumor genomic biomarkers for PD-1 checkpoint blockade-based immunotherapy. Science 362. 10.1126/science.aar3593 10.1126/science.aar3593 PMC6718162 30309915

[CR10] D’Alise AM, Leoni G, De Lucia M et al (2021) Maximizing cancer therapy via complementary mechanisms of immune activation: PD-1 blockade, neoantigen vaccination, and Tregs depletion. J Immunother Cancer 9. 10.1136/jitc-2021-003480 10.1136/jitc-2021-003480 PMC8627409 34824160

[CR11] Dent P, Poklepovic A, Booth L et al (2021) The development of multi-kinase inhibitors as pancreatic cancer therapeutics. Anticancer Drugs 32:779–785. 10.1097/CAD.0000000000001177 34397447 10.1097/CAD.0000000000001177

[CR12] Farha M, Jairath NK, Lawrence TS et al (2020) Characterization of the Tumor Immune Microenvironment identifies M0 macrophage-enriched cluster as a poor prognostic factor in Hepatocellular Carcinoma. JCO Clin Cancer Inf 4:1002–1013. 10.1200/CCI.20.00077 10.1200/CCI.20.00077 PMC7713549 33136432

[CR13] Fitzgerald AA, Wang S, Agarwal V et al (2021) DPP inhibition alters the CXCR3 axis and enhances NK and CD8 + T cell infiltration to improve anti-PD1 efficacy in murine models of pancreatic ductal adenocarcinoma. J Immunother Cancer 9. 10.1136/jitc-2021-002837 10.1136/jitc-2021-002837 PMC8578994 34737215

[CR14] Fridman WH, Zitvogel L, Sautes-Fridman C et al (2017) The immune contexture in cancer prognosis and treatment. Nat Rev Clin Oncol 14:717–734. 10.1038/nrclinonc.2017.101 28741618 10.1038/nrclinonc.2017.101

[CR15] Germain C, Gnjatic S, Tamzalit F et al (2014) Presence of B cells in tertiary lymphoid structures is associated with a protective immunity in patients with lung cancer. Am J Respir Crit Care Med 189:832–844. 10.1164/rccm.201309-1611OC 24484236 10.1164/rccm.201309-1611OC

[CR16] Gubin MM, Zhang X, Schuster H et al (2014) Checkpoint blockade cancer immunotherapy targets tumour-specific mutant antigens. Nature 515:577–581. 10.1038/nature13988 25428507 10.1038/nature13988 PMC4279952

[CR22] Gunderson J, Rajamanickam A, Bui V C et al (2021) Germinal center reactions in tertiary lymphoid structures associate with neoantigen burden, humoral immunity and long-term survivorship in pancreatic cancer. Oncoimmunology 10:1900635. 10.1080/2162402X.2021.1900635 33796412 10.1080/2162402X.2021.1900635 PMC7993148

[CR17] Helmink BA, Reddy SM, Gao J et al (2020) B cells and tertiary lymphoid structures promote immunotherapy response. Nature 577:549–555. 10.1038/s41586-019-1922-8 31942075 10.1038/s41586-019-1922-8 PMC8762581

[CR18] Horeweg N, Workel HH, Loiero D et al (2022) Tertiary lymphoid structures critical for prognosis in endometrial cancer patients. Nat Commun 13:1373. 10.1038/s41467-022-29040-x 35296668 10.1038/s41467-022-29040-x PMC8927106

[CR19] Hu H, Xu Y, Ai XN et al (2023) Comprehensive genomic analysis of the prognostic and immunological characteristics of Tertiary lymphoid structures and CD8 + T-cells in pancreatic ductal adenocarcinoma, PREPRINT (Version 1) available at Research Square [10.21203/rs.3.rs-2860058/v1]. 10.1007/s00432-024-05824-0 PMC11162401 38850373

[CR20] Huang L, Wang Z, Chang Y et al (2020) EFEMP2 indicates assembly of M0 macrophage and more malignant phenotypes of glioma. Aging 12:8397–8412. 10.18632/aging.103147 32396873 10.18632/aging.103147 PMC7244085

[CR21] Ishii K, Shimizu M, Kogo H et al (2020) A combination of check-point blockade and alpha-galactosylceramide elicits long-lasting suppressive effects on murine hepatoma cell growth in vivo. Immunobiology 225:151860. 10.1016/j.imbio.2019.10.009 31812347 10.1016/j.imbio.2019.10.009

[CR23] Jairath NK, Farha MW, Jairath R et al (2020) Prognostic value of intratumoral lymphocyte-to-monocyte ratio and M0 macrophage enrichment in tumor immune microenvironment of melanoma. Melanoma Manag 7:MMT51. 10.2217/mmt-2020-0019 33318782 10.2217/mmt-2020-0019 PMC7727784

[CR24] Kroeger DR, Milne K, Nelson BH (2016) Tumor-infiltrating plasma cells are Associated with Tertiary lymphoid structures, cytolytic T-Cell responses, and Superior Prognosis in Ovarian Cancer. Clin Cancer Res 22:3005–3015. 10.1158/1078-0432.CCR-15-2762 26763251 10.1158/1078-0432.CCR-15-2762

[CR25] Lutz ER, Wu AA, Bigelow E et al (2014) Immunotherapy converts nonimmunogenic pancreatic tumors into immunogenic foci of immune regulation. Cancer Immunol Res 2:616–631. 10.1158/2326-6066.CIR-14-0027 24942756 10.1158/2326-6066.CIR-14-0027 PMC4082460

[CR26] Malta TM, Sokolov A, Gentles AJ et al (2018) Machine learning identifies stemness features Associated with Oncogenic Dedifferentiation. Cell 173:338–354e315. 10.1016/j.cell.2018.03.034 29625051 10.1016/j.cell.2018.03.034 PMC5902191

[CR27] McGrail DJ, Pilie PG, Rashid NU et al (2021) High tumor mutation burden fails to predict immune checkpoint blockade response across all cancer types. Ann Oncol 32:661–672. 10.1016/j.annonc.2021.02.006 33736924 10.1016/j.annonc.2021.02.006 PMC8053682

[CR28] Notta F, Chan-Seng-Yue M, Lemire M et al (2016) A renewed model of pancreatic cancer evolution based on genomic rearrangement patterns. Nature 538:378–382. 10.1038/nature19823 27732578 10.1038/nature19823 PMC5446075

[CR29] Ouyang W, Jiang Y, Bu S et al (2021) A prognostic risk score based on Hypoxia-, Immunity-, and Epithelialto-Mesenchymal transition-related genes for the prognosis and Immunotherapy Response of Lung Adenocarcinoma. Front Cell Dev Biol 9:758777. 10.3389/fcell.2021.758777 35141229 10.3389/fcell.2021.758777 PMC8819669

[CR30] Pearce H, Croft W, Nicol SM et al (2023) Tissue-Resident Memory T cells in pancreatic ductal adenocarcinoma coexpress PD-1 and TIGIT and functional inhibition is reversible by dual antibody blockade. Cancer Immunol Res 11:435–449. 10.1158/2326-6066.Cir-22-0121 36689623 10.1158/2326-6066.CIR-22-0121 PMC10068448

[CR31] Petitprez F, de Reynies A, Keung EZ et al (2020) B cells are associated with survival and immunotherapy response in sarcoma. Nature 577:556–560. 10.1038/s41586-019-1906-8 31942077 10.1038/s41586-019-1906-8

[CR32] Pleasance E, Titmuss E, Williamson L et al (2020) Pan-cancer analysis of advanced patient tumors reveals interactions between therapy and genomic landscapes. Nat Cancer 1:452–468. 10.1038/s43018-020-0050-6 35121966 10.1038/s43018-020-0050-6

[CR33] Rouanne M, Arpaia N, Marabelle A (2021) CXCL13 shapes tertiary lymphoid structures and promotes response to immunotherapy in bladder cancer. Eur J Cancer 151:245–248. 10.1016/j.ejca.2021.03.054 33972155 10.1016/j.ejca.2021.03.054

[CR34] Sade-Feldman M, Yizhak K, Bjorgaard SL et al (2019) Defining T Cell States Associated with response to Checkpoint Immunotherapy in Melanoma. Cell 176:404. 10.1016/j.cell.2018.12.034 30633907 10.1016/j.cell.2018.12.034 PMC6647017

[CR35] Samstein RM, Lee CH, Shoushtari AN et al (2019) Tumor mutational load predicts survival after immunotherapy across multiple cancer types. Nat Genet 51:202–206. 10.1038/s41588-018-0312-8 30643254 10.1038/s41588-018-0312-8 PMC6365097

[CR36] Sautes-Fridman C, Lawand M, Giraldo NA et al (2016) Tertiary lymphoid structures in cancers: Prognostic Value, Regulation, and manipulation for therapeutic intervention. Front Immunol 7:407. 10.3389/fimmu.2016.00407 27752258 10.3389/fimmu.2016.00407 PMC5046074

[CR37] Tanaka T, Masuda A, Inoue J et al (2023) Integrated analysis of tertiary lymphoid structures in relation to tumor-infiltrating lymphocytes and patient survival in pancreatic ductal adenocarcinoma. J Gastroenterol. 10.1007/s00535-022-01939-8 36705749 10.1007/s00535-022-01939-8

[CR38] Thommen DS, Koelzer VH, Herzig P et al (2018) A transcriptionally and functionally distinct PD-1(+) CD8(+) T cell pool with predictive potential in non-small-cell lung cancer treated with PD-1 blockade. Nat Med 24:994–1004. 10.1038/s41591-018-0057-z 29892065 10.1038/s41591-018-0057-z PMC6110381

[CR39] Wirsing AM, Rikardsen OG, Steigen SE et al (2014) Characterisation and prognostic value of tertiary lymphoid structures in oral squamous cell carcinoma. BMC Clin Pathol 14:38. 10.1186/1472-6890-14-38 25177210 10.1186/1472-6890-14-38 PMC4148494

[CR40] Workel HH, Lubbers JM, Arnold R et al (2019) A transcriptionally distinct CXCL13(+)CD103(+)CD8(+) T-cell Population is Associated with B-cell recruitment and Neoantigen load in Human Cancer. Cancer Immunol Res 7:784–796. 10.1158/2326-6066.CIR-18-0517 30872264 10.1158/2326-6066.CIR-18-0517

[CR41] Yang Y, Cao L, Guo Z et al (2022) Deubiquitinase UCHL5 stabilizes ELK3 to potentiate cancer stemness and tumor progression in pancreatic adenocarcinoma (PAAD). Exp Cell Res 421:113402. 10.1016/j.yexcr.2022.113402 36328194 10.1016/j.yexcr.2022.113402

[CR42] Yu T, Tan H, Liu C et al (2022) Integratively genomic analysis reveals the prognostic and immunological characteristics of Pyroptosis and ferroptosis in pancreatic Cancer for Precision Immunotherapy. Front Cell Dev Biol 10:826879. 10.3389/fcell.2022.826879 35242763 10.3389/fcell.2022.826879 PMC8885993

[CR43] Zhang Y, Liu Q, Liao Q (2020) Long noncoding RNA: a dazzling dancer in tumor immune microenvironment. J Exp Clin Cancer Res 39:231. 10.1186/s13046-020-01727-3 33148302 10.1186/s13046-020-01727-3 PMC7641842

[CR44] Zhang Y, Liu X, Wang Y et al (2022a) The m(6)a demethylase ALKBH5-mediated upregulation of DDIT4-AS1 maintains pancreatic cancer stemness and suppresses chemosensitivity by activating the mTOR pathway. Mol Cancer 21:174. 10.1186/s12943-022-01647-0 36056355 10.1186/s12943-022-01647-0 PMC9438157

[CR45] Zhang Y, Zou J, Chen R (2022b) An M0 macrophage-related prognostic model for hepatocellular carcinoma. BMC Cancer 22:791. 10.1186/s12885-022-09872-y 35854246 10.1186/s12885-022-09872-y PMC9294844

[CR46] Zhou L, Xu B, Liu Y et al (2021) Tertiary lymphoid structure signatures are associated with survival and immunotherapy response in muscle-invasive bladder cancer. Oncoimmunology 10:1915574. 10.1080/2162402X.2021.1915574 34104539 10.1080/2162402X.2021.1915574 PMC8143239

[CR47] Zhou T, Liu J, Xie Y et al (2022) ESE3/EHF, a promising target of rosiglitazone, suppresses pancreatic cancer stemness by downregulating CXCR4. Gut 71:357–371. 10.1136/gutjnl-2020-321952 33674341 10.1136/gutjnl-2020-321952 PMC9422994

