# Supplementary material for: Exploring prognostic and immunological characteristics of pancreatic ductal adenocarcinoma through comprehensive genomic analysis of tertiary lymphoid structures and CD8 + T-cells
Source: J Cancer Res Clin Oncol. 2024 Jun 8;150(6):300. doi: 10.1007/s00432-024-05824-0 (PMC11162401; doi:10.1007/s00432-024-05824-0)
Supplement: Supplementary file 1 — Supplementary Material 1 [file 432_2024_5824_MOESM1_ESM.docx]

**Table S1. TLSs Associated Genes and Their Median Expression Levels in Databases**

|  | TCGA | ICGC | MTAB | GSE_Combined |
| --- | --- | --- | --- | --- |
| MEF2B | not commonly expressed | not commonly expressed | not commonly expressed | not commonly expressed |
| SPIB | not commonly expressed | not commonly expressed | not commonly expressed | not commonly expressed |
| MZB1 | not commonly expressed | not commonly expressed | not commonly expressed | not commonly expressed |
| JCHAIN | not commonly expressed | not commonly expressed | not commonly expressed | not commonly expressed |
| CD24 | not commonly expressed | not commonly expressed | not commonly expressed | not commonly expressed |
| CD43 | not commonly expressed | not commonly expressed | not commonly expressed | not commonly expressed |
| CD79B | 2.684 | 2.817 | 2.833 | 2.779 |
| TCF3 | 5.222 | 5.199 | 5.091 | 5.138 |
| LAMP3 | 2.866 | 2.692 | 2.65 | 2.71 |
| MEF2C | 3.742 | 3.608 | 3.79 | 3.784 |
| CD40 | 4.901 | 4.965 | 4.86 | 4.787 |
| DOCK8 | 2.546 | 2.551 | 2.483 | 2.501 |
| POU2AF1 | 2.94 | 2.552 | 2.588 | 2.826 |
| CD69 | 2.474 | 2.643 | 2.332 | 2.479 |
| AICDA | 0.874 | 0.854 | 0.917 | 0.896 |
| CD83 | 4.111 | 4.067 | 4.026 | 4.159 |
| BACH2 | 1.535 | 1.504 | 1.492 | 1.54 |
| CCR6 | 0.809 | 0.874 | 0.857 | 0.889 |
| BCL6 | 5.369 | 5.311 | 5.314 | 5.238 |
| CD86 | 3.344 | 3.067 | 3.163 | 3.191 |
| CXCR4 | 6.89 | 7.066 | 6.911 | 6.93 |
| CCR7 | 3.244 | 3.207 | 3.373 | 3.391 |
| BHLHE40 | 8.809 | 8.88 | 8.812 | 8.692 |
| MYC | 6.164 | 6.181 | 6.088 | 6.093 |
| CCL21 | 4.48 | 4.14 | 4.334 | 4.091 |
| IRF4 | 2.04 | 2.101 | 2.136 | 2.099 |
| IRF8 | 4.408 | 4.422 | 4.353 | 4.257 |
| MCL1 | 7.913 | 7.898 | 7.839 | 7.793 |
| MKI67 | 3.506 | 3.591 | 3.271 | 3.379 |
| CXCL13 | 2.013 | 1.737 | 1.817 | 1.924 |
| CXCR5 | 1.519 | 1.551 | 1.682 | 1.618 |
| HMGB2 | 4.78 | 4.75 | 4.638 | 4.658 |
| EBF1 | 2.814 | 2.703 | 2.878 | 2.814 |
| CCL19 | 5.107 | 4.767 | 5.041 | 5.164 |
| SELL | 3.031 | 2.845 | 2.891 | 2.756 |
| EIF1AY | 2.796 | 1.961 | 2.562 | 2.422 |
| L1CAM | 2.77 | 2.71 | 2.953 | 2.856 |
