# Supplementary material for: Exploring prognostic and immunological characteristics of pancreatic ductal adenocarcinoma through comprehensive genomic analysis of tertiary lymphoid structures and CD8 + T-cells
Source: J Cancer Res Clin Oncol. 2024 Jun 8;150(6):300. doi: 10.1007/s00432-024-05824-0 (PMC11162401; doi:10.1007/s00432-024-05824-0)
Supplement: Supplementary file 2 — Supplementary Material 2 [file 432_2024_5824_MOESM2_ESM.docx]

**Table S2. Gene Clusters Before Boruta**

| **Genes** | **Signature** |
| --- | --- |
| CHMP4C | A |
| TMC7 | A |
| IQGAP3 | A |
| PTK6 | A |
| NTF4 | A |
| FAM83H | A |
| PLEK2 | A |
| OTX1 | A |
| PITX1 | A |
| PARD6B | A |
| NEK2 | A |
| PLEKHN1 | A |
| SH2D4A | A |
| EPHA2 | A |
| IGSF9 | A |
| KIF14 | A |
| NUF2 | A |
| E2F8 | A |
| GRB7 | A |
| KCNK1 | A |
| FA2H | A |
| EFNA4 | A |
| CGN | A |
| ANXA2P1 | A |
| CENPF | A |
| ASPM | A |
| HJURP | A |
| CEP55 | A |
| LEMD1 | A |
| GPR39 | A |
| BUB1B | A |
| OVOL1 | A |
| TMEM139 | A |
| ANLN | A |
| PPP1R13L | A |
| DLGAP5 | A |
| S100A11 | A |
| E2F7 | A |
| CKAP2L | A |
| ANXA2P3 | A |
| RHBDL2 | A |
| KRTCAP3 | A |
| AHNAK2 | A |
| RAB25 | A |
| TBX6 | A |
| FXYD3 | A |
| EXO1 | A |
| TMEM105 | A |
| PLK1 | A |
| CDC25C | A |
| MST1R | A |
| NGEF | A |
| KIF18B | A |
| CCNB2 | A |
| CENPI | A |
| MAL2 | A |
| MET | A |
| PKP3 | A |
| S100A5 | A |
| KCNN4 | A |
| TP73 | A |
| UBE2C | A |
| CBLC | A |
| TFAP2A | A |
| SLC39A4 | A |
| ADAM8 | A |
| IL1A | A |
| TPX2 | A |
| MCM10 | A |
| CDCA2 | A |
| CDH3 | A |
| CATSPER1 | A |
| ESCO2 | A |
| S100A6 | A |
| MKI67 | A |
| NMU | A |
| FAM111B | A |
| STYK1 | A |
| CLIC3 | A |
| FOXQ1 | A |
| SDCBP2 | A |
| SIM2 | A |
| OIP5 | A |
| PDE4C | A |
| BUB1 | A |
| PBK | A |
| B3GNT3 | A |
| LAMB3 | A |
| PLA2G2F | A |
| FOXL1 | A |
| PSORS1C2 | A |
| SH2D3A | A |
| FRMD5 | A |
| PRR15 | A |
| LAMA3 | A |
| KIF18A | A |
| DIAPH3 | A |
| HMMR | A |
| PPARG | A |
| POLQ | A |
| PLA2G4D | A |
| KIF20A | A |
| LAMC2 | A |
| P2RY2 | A |
| EVPL | A |
| NEIL3 | A |
| SLC2A1 | A |
| LBX2 | A |
| TTK | A |
| STEAP3 | A |
| HK2 | A |
| EPS8L1 | A |
| XDH | A |
| SFN | A |
| CABP4 | A |
| MELK | A |
| ADAMTSL5 | A |
| TRIM29 | A |
| TM4SF1 | A |
| GRTP1 | A |
| KRT19 | A |
| ERCC6L | A |
| DNAH3 | A |
| TM6SF2 | A |
| MUC1 | A |
| RHOD | A |
| PRSS22 | A |
| ESPN | A |
| FOXH1 | A |
| LRRC8E | A |
| FERMT1 | A |
| RNF39 | A |
| IGF2BP2 | A |
| S100A14 | A |
| RXFP4 | A |
| ALS2CL | A |
| NXNL2 | A |
| DMBX1 | A |
| GSDMB | A |
| C3orf52 | A |
| ANXA3 | A |
| DEPDC1 | A |
| ANKRD18A | A |
| CA9 | A |
| LY6E | A |
| RAD54L | A |
| CENPA | A |
| MYEOV | A |
| FOXM1 | A |
| GJB3 | A |
| SERPINB5 | A |
| GPRC5A | A |
| TRIM7 | A |
| CREG2 | A |
| BIK | A |
| FER1L4 | A |
| SERINC2 | A |
| COL17A1 | A |
| TSPAN1 | A |
| PROM2 | A |
| NQO1 | A |
| SLC16A3 | A |
| DCST1 | A |
| CAPN8 | A |
| KRT9 | A |
| SLPI | A |
| PHLDA2 | A |
| TMPRSS4 | A |
| GPRC5D | A |
| PKMYT1 | A |
| CDC20 | A |
| GDPD2 | A |
| TJP3 | A |
| TMEM92 | A |
| KLF5 | A |
| MMEL1 | A |
| RASAL1 | A |
| KRT3 | A |
| LAD1 | A |
| IRX5 | A |
| HPDL | A |
| ITGB4 | A |
| MSX2 | A |
| SLC4A11 | A |
| SPOCD1 | A |
| S100P | A |
| VILL | A |
| RP1L1 | A |
| SLURP1 | A |
| TMEM40 | A |
| RP1 | A |
| PLA2G10 | A |
| SEC14L4 | A |
| BTBD16 | A |
| TROAP | A |
| ATP6V1B1 | A |
| RTN4R | A |
| DNAH2 | A |
| C11orf86 | A |
| MSLNL | A |
| PPP1R1C | A |
| TNNT1 | A |
| GRHL2 | A |
| PLA2G4F | A |
| CLDN4 | A |
| OASL | A |
| C5orf46 | A |
| C15orf48 | A |
| SH2D5 | A |
| PSORS1C1 | A |
| EFNA3 | A |
| IFI27 | A |
| GCKR | A |
| FAM83B | A |
| SDR16C5 | A |
| SOX11 | A |
| IGFL2 | A |
| GPR78 | A |
| RHOV | A |
| GJB2 | A |
| ARL14 | A |
| FUT3 | A |
| POU2F3 | A |
| FAM83E | A |
| ACSL5 | A |
| KRT78 | A |
| BIRC7 | A |
| MSLN | A |
| SLC16A5 | A |
| IL1RN | A |
| SLCO4A1 | A |
| CYP26A1 | A |
| PTPRR | A |
| GJB4 | A |
| GPR35 | A |
| ABCA12 | A |
| FAM83A | A |
| MMP13 | A |
| STK31 | A |
| PAEP | A |
| CA14 | A |
| HOXB6 | A |
| LYPD2 | A |
| CALHM3 | A |
| TBX4 | A |
| COX6B2 | A |
| KREMEN2 | A |
| SPATA12 | A |
| PADI1 | A |
| ACRV1 | A |
| HKDC1 | A |
| CDCA7 | A |
| LIPH | A |
| WFDC10B | A |
| TMC5 | A |
| CRABP2 | A |
| CCL20 | A |
| SLC23A3 | A |
| UPK2 | A |
| SPACA4 | A |
| MALL | A |
| CALB2 | A |
| OMP | A |
| KRT15 | A |
| ANKRD22 | A |
| GALNT5 | A |
| IL20RB | A |
| BCL2L14 | A |
| OR2B6 | A |
| CA12 | A |
| PKD1L2 | A |
| TNS4 | A |
| ADAP1 | A |
| C6orf223 | A |
| SYT8 | A |
| HMGA2 | A |
| ACTBL2 | A |
| DUOXA1 | A |
| VSIG2 | A |
| SERPINB3 | A |
| PNPLA3 | A |
| UNC93A | A |
| SLAMF9 | A |
| KISS1 | A |
| CKLF | A |
| ALDH3B2 | A |
| RAPGEFL1 | A |
| IBSP | A |
| SLC7A11 | A |
| SCGB3A2 | A |
| CHST6 | A |
| LY6G6C | A |
| IL31RA | A |
| ABO | A |
| PPFIA4 | A |
| ITGB6 | A |
| ALDH3A1 | A |
| ARHGAP8 | A |
| TACSTD2 | A |
| CYP2S1 | A |
| CASP14 | A |
| LHB | A |
| HAS3 | A |
| RNF183 | A |
| NTSR1 | A |
| ICAM5 | A |
| ZIC2 | A |
| KLK10 | A |
| TRIM31 | A |
| CYP2W1 | A |
| NPR3 | A |
| TBX15 | A |
| KRT6C | A |
| MAPK15 | A |
| SH3RF2 | A |
| DQX1 | A |
| SCEL | A |
| EREG | A |
| HTR1B | A |
| SRMS | A |
| TNFSF9 | A |
| PGLYRP3 | A |
| WFDC3 | A |
| PCSK9 | A |
| WFDC13 | A |
| PI3 | A |
| CLCN1 | A |
| KRT79 | A |
| MYPN | A |
| AHSG | A |
| PRSS8 | A |
| TMEM171 | A |
| HYAL4 | A |
| GUCY1B2 | A |
| KRT7 | A |
| PPP1R14D | A |
| PSCA | A |
| ST8SIA2 | A |
| SEMA7A | A |
| CTSE | A |
| TKTL1 | A |
| ANKRD33 | A |
| SLC38A5 | A |
| SCT | A |
| KRT17 | A |
| EPN3 | A |
| CRCT1 | A |
| FGF20 | A |
| EGLN3 | A |
| TNNT2 | A |
| POF1B | A |
| ULBP2 | A |
| CLDN18 | A |
| GBP6 | A |
| PRSS33 | A |
| SIX1 | A |
| ITPKA | A |
| PTGES | A |
| GPR20 | A |
| PADI3 | A |
| SMPX | A |
| ELF5 | A |
| COL11A1 | A |
| APOBEC1 | A |
| SULT1C2 | A |
| SERPINB7 | A |
| MOGAT3 | A |
| PNCK | A |
| SPRR1A | A |
| WNT7B | A |
| GRP | A |
| LY6D | A |
| DKK1 | A |
| SOX21 | A |
| RPTN | A |
| CSF2 | A |
| KLHDC7B | A |
| AGR2 | A |
| SERPINB13 | A |
| BCAS1 | A |
| LGR6 | A |
| CP | A |
| ALPP | A |
| EFNA2 | A |
| AQP2 | A |
| KRT80 | A |
| KRT13 | A |
| PLAC1 | A |
| S100A2 | A |
| CEACAM5 | A |
| KRT6A | A |
| WNT2 | A |
| CST1 | A |
| PKP1 | A |
| SFTA2 | A |
| CYP24A1 | A |
| VGLL1 | A |
| EPYC | A |
| SLC26A9 | A |
| SERPINB4 | A |
| COL7A1 | A |
| IL22RA1 | A |
| WNT7A | A |
| GAD1 | A |
| SLC24A2 | A |
| SPRR1B | A |
| ABCA13 | A |
| AKR7A3 | A |
| ZBED2 | A |
| A2ML1 | A |
| KANK4 | A |
| HSD17B2 | A |
| KLK8 | A |
| OBP2B | A |
| GPR87 | A |
| TRIM10 | A |
| AQP6 | A |
| GSDMC | A |
| EVX1 | A |
| KLK7 | A |
| UPK3B | A |
| NKAIN4 | A |
| EDAR | A |
| SFTPC | A |
| FGFBP1 | A |
| CREB3L3 | A |
| DUSP9 | A |
| TUBAL3 | A |
| MMP11 | A |
| HOXB9 | A |
| LCN2 | A |
| KLK4 | A |
| PDZK1IP1 | A |
| CDA | A |
| IGF2BP3 | A |
| LRRC31 | A |
| HES2 | A |
| FOXA1 | A |
| TNNI2 | A |
| HPR | A |
| GATA4 | A |
| COL10A1 | A |
| CXCL5 | A |
| DLX3 | A |
| ANXA10 | A |
| ANKFN1 | A |
| KLK11 | A |
| ZSCAN4 | A |
| AQP5 | A |
| C4BPB | A |
| HOXA10 | A |
| HHIP | A |
| TNNI3 | A |
| CES3 | A |
| CXCL3 | A |
| WNT10A | A |
| SLC13A5 | A |
| MUC4 | A |
| DSG3 | A |
| SLC9A2 | A |
| CYP2C18 | A |
| KCNA7 | A |
| S100A7 | A |
| HOXA13 | A |
| C12orf56 | A |
| GJB6 | A |
| SPRR2D | A |
| SERPINB2 | A |
| DMBT1 | A |
| SVOPL | A |
| CALML5 | A |
| HOXD10 | A |
| OR10H1 | A |
| SLC6A20 | A |
| LGR5 | A |
| KRT4 | A |
| SPINK7 | A |
| EPS8L3 | A |
| CYP4F12 | A |
| VSIG1 | A |
| SHH | A |
| PTGS2 | A |
| TGM5 | A |
| MOGAT2 | A |
| CAPN9 | A |
| HOXB1 | A |
| GJB5 | A |
| ERN2 | A |
| HAPLN1 | A |
| TRIM15 | A |
| AGR3 | A |
| PSAPL1 | A |
| CACNG8 | A |
| TRIM54 | A |
| FABP6 | A |
| PRB2 | A |
| ODAM | A |
| KRT14 | A |
| DKK4 | A |
| FAM169B | A |
| AADAC | A |
| LRRC15 | A |
| FIBCD1 | A |
| FXYD4 | A |
| BARX1 | A |
| NR1I2 | A |
| CEACAM6 | A |
| CLCA4 | A |
| CYP2C19 | A |
| MARCO | A |
| UCA1 | A |
| TERT | A |
| SPRR3 | A |
| CXCL17 | A |
| DUOXA2 | A |
| SULT1B1 | A |
| PIK3C2G | A |
| KLK6 | A |
| SPDEF | A |
| HOXA11 | A |
| MUC17 | A |
| MMP7 | A |
| C6orf15 | A |
| CST6 | A |
| DUOX2 | A |
| PRSS21 | A |
| PAX9 | A |
| NPSR1 | A |
| HOXC10 | A |
| PHACTR3 | A |
| DHRS9 | A |
| CACNG6 | A |
| OFCC1 | A |
| KRT6B | A |
| TINAG | A |
| CCK | A |
| ATP10B | A |
| PAX7 | A |
| PLA2G3 | A |
| IVL | A |
| OLFM4 | A |
| NLRP2 | A |
| B3GNT6 | A |
| MUC16 | A |
| AKR1B10 | A |
| FAM107A | B |
| SOCS2 | B |
| ARHGEF15 | B |
| NPR1 | B |
| TSPAN7 | B |
| ELMO1 | B |
| CCDC3 | B |
| S1PR1 | B |
| GIMAP1 | B |
| RAMP3 | B |
| APOLD1 | B |
| GIMAP7 | B |
| ANK2 | B |
| EMCN | B |
| PDE2A | B |
| SEMA3G | B |
| LCN6 | B |
| CLEC3B | B |
| EDNRB | B |
| SELP | B |
| ATOH8 | B |
| GIMAP6 | B |
| ITGA7 | B |
| TACR1 | B |
| GPX3 | B |
| CLDN5 | B |
| ITM2A | B |
| PPP1R16B | B |
| IL33 | B |
| RAB3C | B |
| CACNB2 | B |
| PDK4 | B |
| ABCB1 | B |
| TMTC1 | B |
| RUNDC3B | B |
| ZNF831 | B |
| CD300LG | B |
| CD36 | B |
| SNTG2 | B |
| ATP1A2 | B |
| FHL1 | B |
| CNTFR | B |
| PGM5 | B |
| DNASE1L3 | B |
| MMRN1 | B |
| C7 | B |
| GHR | B |
| MYRIP | B |
| ZNF385D | B |
| SRPX | B |
| MAPK10 | B |
| FBLN5 | B |
| SLIT3 | B |
| ASPA | B |
| CNR1 | B |
| DTNA | B |
| ABCA8 | B |
| LYVE1 | B |
| KIF19 | B |
| GFRA1 | B |
| C16orf89 | B |
| SCARA5 | B |
| RBP7 | B |
| DLG2 | B |
| NRN1 | B |
| GNG7 | B |
| NCAM1 | B |
| LGI2 | B |
| ATP1B2 | B |
| TSPAN33 | B |
| TPO | B |
| NOVA1 | B |
| GNAO1 | B |
| P2RY8 | B |
| KCNB1 | B |
| NAP1L2 | B |
| CPE | B |
| GNG2 | B |
| RELN | B |
| SPTB | B |
| DPT | B |
| RASD1 | B |
| SCN4A | B |
| ACSL6 | B |
| CD8A | B |
| LRRC2 | B |
| CXCL12 | B |
| CRHBP | B |
| RAB39B | B |
| XKR4 | B |
| ADIPOQ | B |
| SLCO1C1 | B |
| SLC8A3 | B |
| PKHD1L1 | B |
| TRPM6 | B |
| TOX | B |
| HLF | B |
| NTRK3 | B |
| COL14A1 | B |
| GFRA2 | B |
| AGTR1 | B |
| CBFA2T3 | B |
| TNR | B |
| SNAP25 | B |
| BMP6 | B |
| GZMM | B |
| SERPINE2 | B |
| TMC2 | B |
| LPL | B |
| KCNA2 | B |
| CNTN2 | B |
| UCHL1 | B |
| ZBTB16 | B |
| NLGN4X | B |
| SFRP1 | B |
| MADCAM1 | B |
| DCLK1 | B |
| KLRB1 | B |
| CACNA1I | B |
| NGFR | B |
| GNAZ | B |
| HSF5 | B |
| TRIM9 | B |
| AFF3 | B |
| FABP4 | B |
| NRXN1 | B |
| CHRDL1 | B |
| SCN3A | B |
| UNC13A | B |
| ADAMTS1 | B |
| RASGRP2 | B |
| PLCXD3 | B |
| SCML4 | B |
| ABCC9 | B |
| CMA1 | B |
| CACNA2D2 | B |
| RCSD1 | B |
| DHH | B |
| SPOCK2 | B |
| KCNMA1 | B |
| TSPAN11 | B |
| HSPB6 | B |
| CADM3 | B |
| ATP2A3 | B |
| MYOC | B |
| PDE3B | B |
| SCN3B | B |
| SLITRK2 | B |
| PI16 | B |
| CD5 | B |
| CBLN4 | B |
| ANGPTL1 | B |
| NTRK2 | B |
| KLHL32 | B |
| ZAP70 | B |
| KCNA6 | B |
| KIF5C | B |
| KRT27 | B |
| FKBP5 | B |
| SORCS1 | B |
| PTX3 | B |
| GIMAP5 | B |
| RTN1 | B |
| BMPER | B |
| OLFM1 | B |
| KL | B |
| CLEC10A | B |
| TRPM3 | B |
| SCG2 | B |
| KCNA5 | B |
| ACER1 | B |
| ABCD2 | B |
| GALNT8 | B |
| ASTN1 | B |
| DDX25 | B |
| CFD | B |
| PRKCB | B |
| HMGCLL1 | B |
| TMOD1 | B |
| SLC7A10 | B |
| PRUNE2 | B |
| SSTR2 | B |
| PACSIN1 | B |
| FBXL16 | B |
| CHL1 | B |
| LRRN3 | B |
| SLC22A17 | B |
| CIDEA | B |
| CNTNAP4 | B |
| SLC7A2 | B |
| GPR142 | B |
| IGF1 | B |
| ANKS1B | B |
| AOX1 | B |
| F10 | B |
| IL5RA | B |
| ADRA1A | B |
| GPM6A | B |
| GRIA3 | B |
| HEMGN | B |
| CTSG | B |
| XPNPEP2 | B |
| CD79B | B |
| SLC29A4 | B |
| B3GALT2 | B |
| IKZF1 | B |
| SPTBN4 | B |
| PCSK1 | B |
| CHRNB2 | B |
| SYT7 | B |
| RBM11 | B |
| GNG4 | B |
| DPYS | B |
| SCN7A | B |
| PTPN5 | B |
| NAT8L | B |
| KCNK3 | B |
| C12orf42 | B |
| SLC14A2 | B |
| RIC3 | B |
| SYP | B |
| KLHL14 | B |
| KHDRBS2 | B |
| SSTR4 | B |
| C10orf82 | B |
| NKAIN2 | B |
| GABRG1 | B |
| PCDH15 | B |
| TRDN | B |
| COL25A1 | B |
| PPM1E | B |
| BEND4 | B |
| TNMD | B |
| NLGN1 | B |
| DPYSL5 | B |
| THBS4 | B |
| XCR1 | B |
| OGN | B |
| DNAI2 | B |
| CLEC4C | B |
| STAB2 | B |
| STXBP5L | B |
| PYHIN1 | B |
| POU6F2 | B |
| MGAT4C | B |
| NRCAM | B |
| FMN2 | B |
| CACNA1B | B |
| SCRG1 | B |
| DCX | B |
| KCNA3 | B |
| TCEAL2 | B |
| KCNH6 | B |
| CLU | B |
| SLC6A4 | B |
| TCEAL5 | B |
| ADCY1 | B |
| CSN1S1 | B |
| ITK | B |
| ANGPTL5 | B |
| MS4A1 | B |
| ST18 | B |
| PNMA3 | B |
| LEP | B |
| TAL2 | B |
| RIMS4 | B |
| FCN2 | B |
| BSN | B |
| P2RY12 | B |
| CLGN | B |
| FAM163A | B |
| BEX2 | B |
| PLP1 | B |
| DIRAS1 | B |
| OPRD1 | B |
| CMTM5 | B |
| GDAP1L1 | B |
| MMD2 | B |
| FAM135B | B |
| ATRNL1 | B |
| SLITRK1 | B |
| MUSK | B |
| BTLA | B |
| SHD | B |
| PPP4R4 | B |
| SLC4A10 | B |
| KCNA1 | B |
| OXGR1 | B |
| AGTR2 | B |
| KCTD8 | B |
| MAS1L | B |
| GABRB1 | B |
| PPP1R1A | B |
| CCR7 | B |
| HEPACAM | B |
| INA | B |
| INSM1 | B |
| GHRL | B |
| KCNK17 | B |
| HFM1 | B |
| AGBL4 | B |
| GZMK | B |
| CBLN2 | B |
| PTPRT | B |
| EOMES | B |
| CNR2 | B |
| MAL | B |
| ADAMTS8 | B |
| OSR1 | B |
| RUNDC3A | B |
| TNNT3 | B |
| RXRG | B |
| CD22 | B |
| VIPR2 | B |
| CACNA1A | B |
| DUSP26 | B |
| PMP2 | B |
| TAGLN3 | B |
| BLK | B |
| RIMS1 | B |
| KRT72 | B |
| GRIA1 | B |
| VIT | B |
| B3GAT1 | B |
| SNAP91 | B |
| MT1JP | B |
| RIPPLY2 | B |
| ATP2B3 | B |
| TCL1A | B |
| ZNF157 | B |
| WNT4 | B |
| FLT3 | B |
| ADARB2 | B |
| GABRB3 | B |
| CCL21 | B |
| GLYAT | B |
| AQP4 | B |
| SLC6A13 | B |
| FRMPD1 | B |
| C1QTNF4 | B |
| OGDHL | B |
| KCNB2 | B |
| KCNJ3 | B |
| RERGL | B |
| CBLN1 | B |
| MAPK4 | B |
| ZNF80 | B |
| SEZ6 | B |
| MT1A | B |
| ADCYAP1 | B |
| SVOP | B |
| NKX2-2 | B |
| LINGO4 | B |
| VWA5B2 | B |
| SLC32A1 | B |
| SYT10 | B |
| PCSK1N | B |
| BEX1 | B |
| PROK1 | B |
| MYT1 | B |
| SLC12A1 | B |
| P2RX5 | B |
| ELAVL4 | B |
| RIMBP2 | B |
| TLR10 | B |
| THRSP | B |
| GPD1 | B |
| SCG5 | B |
| KCNMB2 | B |
| PRPH | B |
| ZDHHC22 | B |
| GALR1 | B |
| LINGO2 | B |
| SLC6A17 | B |
| PAX5 | B |
| KCNG3 | B |
| PAK3 | B |
| ECE2 | B |
| LGI3 | B |
| C1orf127 | B |
| SCG3 | B |
| TCF23 | B |
| TMEM132C | B |
| ANGPTL7 | B |
| UCN3 | B |
| GRM7 | B |
| PTPRN | B |
| DSCAML1 | B |
| USP41 | B |
| SYT4 | B |
| SERPINA10 | B |
| FCER2 | B |
| COL19A1 | B |
| HHATL | B |
| TMEM196 | B |
| CDH10 | B |
| CCL19 | B |
| KLB | B |
| CA10 | B |
| FEV | B |
| FCRL1 | B |
| NRSN1 | B |
| SELE | B |
| FOSB | B |
| CALY | B |
| ADH1A | B |
| SH3GL2 | B |
| CPA5 | B |
| SLC7A14 | B |
| CNGA3 | B |
| LGI1 | B |
| APLP1 | B |
| ACTL6B | B |
| HEPACAM2 | B |
| KCNC2 | B |
| KCNC1 | B |
| PENK | B |
| SLC8A2 | B |
| ATCAY | B |
| GABRA1 | B |
| GABRA2 | B |
| LBP | B |
| C1QL1 | B |
| SLC6A15 | B |
| STMN4 | B |
| CR2 | B |
| VPREB3 | B |
| FCRL3 | B |
| TSPAN19 | B |
| TMEM179 | B |
| TMEM132D | B |
| NOL4 | B |
| PLA2G2A | B |
| ASCL1 | B |
| FCRLA | B |
| KIF1A | B |
| NLRP4 | B |
| CDH12 | B |
| SPAG6 | B |
| SLC2A2 | B |
| ABCC8 | B |
| DPP6 | B |
| HAS1 | B |
| A1CF | B |
| PAPPA2 | B |
| CHRDL2 | B |
| CDH18 | B |
| HBB | B |
| SLC16A12 | B |
| MYT1L | B |
| CNTN5 | B |
| CHGB | B |
| PRLHR | B |
| MAP3K15 | B |
| GUCA2A | B |
| SORCS3 | B |
| ARX | B |
| CAMK2B | B |
| CD19 | B |
| CSF3 | B |
| IL6 | B |
| APOH | B |
| SYT5 | B |
| SMOC1 | B |
| SEZ6L | B |
| GRIA2 | B |
| CILP | B |
| VSTM2A | B |
| CABP7 | B |
| CXCL13 | B |
| HS6ST3 | B |
| CACNG5 | B |
| NPHS1 | B |
| FCRL2 | B |
| NR0B1 | B |
| PLA2G2D | B |
| SCGN | B |
| CD79A | B |
| SCRT2 | B |
| VGF | B |
| VIP | B |
| GPR119 | B |
| CHGA | B |
| GABRG2 | B |
| GABRA4 | B |
| CPLX2 | B |
| NPY | B |
| RFX6 | B |
| LHFPL4 | B |
| NTS | B |
| CARTPT | B |
| GRM4 | B |
| IGHM | B |
| PCSK2 | B |
| DPP10 | B |
| NEUROD1 | B |
| GJD2 | B |
| GC | B |
| CRYBA2 | B |
| DES | B |
| FFAR1 | B |
| PAH | B |
| GAD2 | B |
| SST | B |
