# Supplementary material for: Exploring prognostic and immunological characteristics of pancreatic ductal adenocarcinoma through comprehensive genomic analysis of tertiary lymphoid structures and CD8 + T-cells
Source: J Cancer Res Clin Oncol. 2024 Jun 8;150(6):300. doi: 10.1007/s00432-024-05824-0 (PMC11162401; doi:10.1007/s00432-024-05824-0)
Supplement: Supplementary file 3 — Supplementary Material 3 [file 432_2024_5824_MOESM3_ESM.docx]

**Table S3. TCGA-PAAD Clinical Features**

|  | Type | Location | Gender | Gender.I | Age | Age.I | Age.II | Grade | Grade.I | Grade.II | Size | Stage | TNM.Stage | TNM.Stage.I | TNM.Stage.II | TNM.Stage.III | T.Stage | T.Stage.I | T.Stage.II | N.Stage | os.status | Survival | os | T-C score | Group | Group.I | TIDE_score | TIDE_respond | mRNAsi | mDNAsi | cluster.I | cluster.II | CD8 |
| --- | --- | --- | --- | --- | --- | --- | --- | --- | --- | --- | --- | --- | --- | --- | --- | --- | --- | --- | --- | --- | --- | --- | --- | --- | --- | --- | --- | --- | --- | --- | --- | --- | --- |
| TCGA-HZ-A77P | Pancreas-Adenocarcinoma-Other Subtype | Head of Pancreas | MALE | 2 | 77 | >65 | 2 | G1 | 1 | G1 | 3.1 | 7thStage IIBT3N1MX | IIB | 4 | II | II | T3 | 3 | T3+T4 | N1 | 0 | Alive | 13 | -3.14158 | Low | 1 | 1.04 | FALSE | 0.213739 | 0.249222 | 1 | 1 | 0.0188 |
| TCGA-IB-AAUR | Pancreas-Adenocarcinoma Ductal Type | Head of Pancreas | MALE | 2 | 67 | >65 | 2 | G1 | 1 | G1 | 3.7 | 7thStage IIBT3N1M0 | IIB | 4 | II | II | T3 | 3 | T3+T4 | N1 | 0 | Alive | 128 | -3.00184 | Low | 1 | 1.85 | FALSE | 0.277986 | 0.186251 | 1 | 2 | 0.103 |
| TCGA-3A-A9IV | Pancreas-Adenocarcinoma-Other Subtype | Tail of Pancreas | FEMALE | 1 | 59 | 65<= | 1 | G1 | 1 | G1 | 3 | 7thStage IBT2N0MX | IB | 2 | I | I | T2 | 2 | T1+T2 | N0 | 0 | Alive | 967 | -24.2492 | Low | 1 | 0.19 | FALSE | 0.368221 | 0.394889 | 2 | 2 | 0.0279 |
| TCGA-2J-AABH | Pancreas-Adenocarcinoma-Other Subtype | Body of Pancreas | MALE | 2 | 61 | 65<= | 1 | G3 | 3 | G3+G4 | 2.2 | 7thStage IIAT3N0M0 | IIA | 3 | II | II | T3 | 3 | T3+T4 | N0 | 0 | Alive | 671 | -1.01494 | Low | 1 | -0.7 | TRUE | 0.389845 | 0.27558 | 2 | 2 | 0.00419 |
| TCGA-F2-A44H | Pancreas-Adenocarcinoma Ductal Type | Head of Pancreas | MALE | 2 | 65 | 65<= | 1 | G2 | 2 | G2 | 3.5 | 7thStage IIAT3N0M0 | IIA | 3 | II | II | T3 | 3 | T3+T4 | N0 | 0 | Alive | 158 | -4.09978 | Low | 1 | 0.24 | FALSE | 0.268619 | 0.187919 | 2 | 2 | 4.19E-22 |
| TCGA-HZ-7924 | Pancreas-Adenocarcinoma Ductal Type | Head of Pancreas | FEMALE | 1 | 60 | 65<= | 1 | G2 | 2 | G2 | 5 | 7thStage IIAT3N0MX | IIA | 3 | II | II | T3 | 3 | T3+T4 | N0 | 0 | Alive | 369 | -4.08833 | Low | 1 | -1.16 | TRUE | 0.548303 | 0.154767 | 1 | 2 | 0.014 |
| TCGA-HZ-A49G | Pancreas-Adenocarcinoma Ductal Type | Head of Pancreas | FEMALE | 1 | 58 | 65<= | 1 | G2 | 2 | G2 | 3 | 7thStage IIBT2N1MX | IIB | 4 | II | II | T2 | 2 | T1+T2 | N1 | 0 | Alive | 23 | -1.62514 | Low | 1 | -1.46 | TRUE | 0.242553 | 0.190655 | 1 | 2 | 0.00419 |
| TCGA-FB-AAQ2 | Pancreas-Adenocarcinoma Ductal Type | Head of Pancreas | FEMALE | 1 | 81 | >65 | 2 | G3 | 3 | G3+G4 | 5.5 | 7thStage IIBT3N1MX | IIB | 4 | II | II | T3 | 3 | T3+T4 | N1 | 1 | Dead | 153 | 7.517605 | High | 2 | 0.86 | FALSE | 0.687681 | 0.308286 | 2 | 1 | 0.00849 |
| TCGA-2L-AAQE | Pancreas-Adenocarcinoma Ductal Type | Head of Pancreas | MALE | 2 | 56 | 65<= | 1 | G2 | 2 | G2 | 4.5 | 7thStage IIBT3N1M0 | IIB | 4 | II | II | T3 | 3 | T3+T4 | N1 | 1 | Dead | 684 | 8.297747 | High | 2 | 0.67 | FALSE | 0.433379 | 0.388111 | 2 | 2 | 0.00691 |
| TCGA-IB-AAUM | Pancreas-Adenocarcinoma Ductal Type | Head of Pancreas | MALE | 2 | 76 | >65 | 2 | G3 | 3 | G3+G4 | 2.1 | 7thStage IIBT2N1M0 | IIB | 4 | II | II | T2 | 2 | T1+T2 | N1 | 0 | Alive | 8 | -7.98077 | Low | 1 | -1.38 | TRUE | 0.326699 | 0.286838 | 2 | 2 | 0.0291 |
| TCGA-FB-A4P6 | Pancreas-Adenocarcinoma Ductal Type | Head of Pancreas | MALE | 2 | 54 | 65<= | 1 | G1 | 1 | G1 | 4.5 | 7thStage IIBT3N1MX | IIB | 4 | II | II | T3 | 3 | T3+T4 | N1 | 0 | Alive | 7 | -5.22701 | Low | 1 | -0.87 | TRUE | 0.203014 | 0.059354 | 1 | 2 | 0.0414 |
| TCGA-3A-A9IO | Pancreas-Adenocarcinoma-Other Subtype | Body of Pancreas | MALE | 2 | 55 | 65<= | 1 | G1 | 1 | G1 | 3 | 6thTXNXMX | IB | 2 | I | I | T2 | 2 | T1+T2 | N0 | 0 | Alive | 1436 | -25.5363 | Low | 1 | -0.65 | TRUE | 0.564161 | 0.052135 | 1 | 2 | 0.0595 |
| TCGA-YB-A89D | Pancreas-Adenocarcinoma Ductal Type | Head of Pancreas | MALE | 2 | 59 | 65<= | 1 | G2 | 2 | G2 | 3.2 | 7thStage IIBT3N1MX | IIB | 4 | II | II | T3 | 3 | T3+T4 | N1 | 0 | Alive | 160 | -1.44858 | Low | 1 | 1.08 | FALSE | 0.163646 | 0.209217 | 2 | 2 | 1.79E-20 |
| TCGA-HZ-7925 | Pancreas-Adenocarcinoma Ductal Type | Head of Pancreas | MALE | 2 | 66 | >65 | 2 | G2 | 2 | G2 | 4.7 | 7thStage IIBT3N1MX | IIB | 4 | II | II | T3 | 3 | T3+T4 | N1 | 0 | Alive | 361 | 4.097651 | High | 2 | 2.01 | FALSE | 0.249973 | 0.322678 | 2 | 3 | 0.00668 |
| TCGA-IB-7654 | Pancreas-Adenocarcinoma Ductal Type | Head of Pancreas | MALE | 2 | 80 | >65 | 2 | G2 | 2 | G2 | 3 | 7thStage IIBT2N1M0 | IIB | 4 | II | II | T2 | 2 | T1+T2 | N1 | 1 | Dead | 476 | -2.25057 | Low | 1 | 0.21 | FALSE | 0.37043 | 0.301716 | 2 | 3 | 0.000587 |
| TCGA-XN-A8T5 | Pancreas-Adenocarcinoma-Other Subtype | Head of Pancreas | FEMALE | 1 | 53 | 65<= | 1 | G2 | 2 | G2 | 2.5 | 7thStage IBT2N0M0 | IB | 2 | I | I | T2 | 2 | T1+T2 | N0 | 0 | Alive | 720 | -3.78073 | Low | 1 | 1.21 | FALSE | 0.208087 | 0.215682 | 1 | 1 | 0.0472 |
| TCGA-3A-A9IS | Pancreas-Adenocarcinoma-Other Subtype | Body of Pancreas | MALE | 2 | 67 | >65 | 2 | G1 | 1 | G1 | 8 | 7thStage IBT2N0MX | IB | 2 | I | I | T2 | 2 | T1+T2 | N0 | 0 | Alive | 932 | -28.2429 | Low | 1 | -0.68 | TRUE | 0.66206 | 0.45578 | 1 | 2 | 0.0265 |
| TCGA-XD-AAUI | Pancreas-Adenocarcinoma Ductal Type | Head of Pancreas | FEMALE | 1 | 50 | 65<= | 1 | G2 | 2 | G2 | 3.5 | 7thStage IIBT3N1MX | IIB | 4 | II | II | T3 | 3 | T3+T4 | N1 | 0 | Alive | 202 | 3.051033 | High | 2 | 0.19 | FALSE | 0.390602 | 0.471743 | 1 | 3 | ######## |
| TCGA-HV-A7OL | Pancreas-Adenocarcinoma Ductal Type | Head of Pancreas | MALE | 2 | 70 | >65 | 2 | G2 | 2 | G2 | NA | 7thStage IIAT3N0MX | IIA | 3 | II | II | T3 | 3 | T3+T4 | N0 | 0 | Alive | 252 | 7.117901 | High | 2 | -1.16 | TRUE | 0.623404 | 0.243976 | 2 | 3 | 1.97E-18 |
| TCGA-HZ-8002 | Pancreas-Adenocarcinoma Ductal Type | Head of Pancreas | MALE | 2 | 61 | 65<= | 1 | G2 | 2 | G2 | 3.1 | 7thStage IIBT3N1MX | IIB | 4 | II | II | T3 | 3 | T3+T4 | N1 | 0 | Alive | 24 | -5.56665 | Low | 1 | -0.55 | TRUE | 0.248169 | 0.226339 | 1 | 2 | 0.0355 |
| TCGA-3A-A9IL | Pancreas-Adenocarcinoma-Other Subtype | Head of Pancreas | FEMALE | 1 | 39 | 65<= | 1 | G1 | 1 | G1 | 1.3 | 6thStage IT1MX | IA | 1 | I | I | T1 | 1 | T1+T2 | N0 | 0 | Alive | 2558 | -21.4601 | Low | 1 | -0.31 | TRUE | 0.356305 | 0.920108 | 1 | 2 | 0.0376 |
| TCGA-3A-A9J0 | Pancreas-Adenocarcinoma Ductal Type | Head of Pancreas | MALE | 2 | 75 | >65 | 2 | G2 | 2 | G2 | 4 | 7thStage IIBT3N1MX | IIB | 4 | II | II | T3 | 3 | T3+T4 | N1 | 0 | Alive | 377 | 7.181351 | High | 2 | 0.79 | FALSE | 0.40812 | 0.353797 | 2 | 3 | 0.00749 |
| TCGA-OE-A75W | Pancreas-Adenocarcinoma Ductal Type | Tail of Pancreas | MALE | 2 | 75 | >65 | 2 | G1 | 1 | G1 | 4.5 | 7thStage IIAT3N0M0 | IIA | 3 | II | II | T3 | 3 | T3+T4 | N0 | 0 | Alive | 110 | 6.181847 | High | 2 | 0.88 | FALSE | 0.424994 | 0.419776 | 2 | 3 | 0.0112 |
| TCGA-2J-AABF | Pancreas-Adenocarcinoma Ductal Type | Head of Pancreas | MALE | 2 | 73 | >65 | 2 | G3 | 3 | G3+G4 | 3.2 | 7thStage IIBT3N1M0 | IIB | 4 | II | II | T3 | 3 | T3+T4 | N1 | 1 | Dead | 691 | -0.85442 | Low | 1 | 0.67 | FALSE | 0.424105 | 0.366821 | 2 | 2 | 0.0237 |
| TCGA-FB-AAQ6 | Pancreas-Adenocarcinoma Ductal Type | Tail of Pancreas | MALE | 2 | 85 | >65 | 2 | G2 | 2 | G2 | 2 | 7thStage IAT1N0MX | IA | 1 | I | I | T1 | 1 | T1+T2 | N0 | 1 | Dead | 244 | 2.342133 | High | 2 | 0.18 | FALSE | 0.572254 | 0.279941 | 2 | 2 | 0.00579 |
| TCGA-PZ-A5RE | Pancreas-Adenocarcinoma Ductal Type | Head of Pancreas | FEMALE | 1 | 44 | 65<= | 1 | G3 | 3 | G3+G4 | 3 | 7thStage IIBT3N1MX | IIB | 4 | II | II | T3 | 3 | T3+T4 | N1 | 0 | Alive | 247 | 6.413764 | High | 2 | -0.29 | TRUE | 0.346992 | 0.424424 | 2 | 3 | ######## |
| TCGA-2J-AAB9 | Pancreas-Adenocarcinoma Ductal Type | Head of Pancreas | FEMALE | 1 | 70 | >65 | 2 | G1 | 1 | G1 | 4 | 7thStage IIBT3N1M0 | IIB | 4 | II | II | T3 | 3 | T3+T4 | N1 | 1 | Dead | 627 | 1.462385 | High | 2 | 0.78 | FALSE | 0.288303 | 0.319331 | 1 | 3 | 0.00359 |
| TCGA-2L-AAQJ | Pancreas-Adenocarcinoma Ductal Type | Head of Pancreas | FEMALE | 1 | 49 | 65<= | 1 | G2 | 2 | G2 | 3 | 7thStage IIIT4N0MX | III | 5 | III | III+IV | T4 | 4 | T3+T4 | N0 | 1 | Dead | 394 | 5.155878 | High | 2 | -0.81 | TRUE | 0.427507 | 0.290473 | 2 | 3 | ######## |
| TCGA-HZ-A4BH | Pancreas-Adenocarcinoma Ductal Type | Head of Pancreas | MALE | 2 | 75 | >65 | 2 | G3 | 3 | G3+G4 | 2.5 | 7thStage IIBT3N1bMX | IIB | 4 | II | II | T3 | 3 | T3+T4 | N1 | 0 | Alive | 194 | 1.756202 | High | 2 | 0.63 | FALSE | 0.332681 | 0.303814 | 2 | 2 | 0.0127 |
| TCGA-FB-AAPU | Pancreas-Adenocarcinoma Ductal Type | Head of Pancreas | FEMALE | 1 | 41 | 65<= | 1 | G2 | 2 | G2 | 4 | 7thStage IIAT3N0MX | IIA | 3 | II | II | T3 | 3 | T3+T4 | N0 | 1 | Dead | 381 | 2.468658 | High | 2 | -0.88 | TRUE | 0.681738 | 0.368657 | 2 | 2 | 0.013 |
| TCGA-Z5-AAPL | Pancreas-Adenocarcinoma Ductal Type | Other (please specify) | FEMALE | 1 | 74 | >65 | 2 | G1 | 1 | G1 | NA | 7thStage IIAT3N0M0 | IIA | 3 | II | II | T3 | 3 | T3+T4 | N0 | 0 | Alive | 21 | -1.94882 | Low | 1 | 1.2 | FALSE | 0.663665 | 0.291597 | 1 | 2 | 0.179 |
| TCGA-FB-AAPQ | Pancreas-Adenocarcinoma Ductal Type | Head of Pancreas | MALE | 2 | 65 | 65<= | 1 | G2 | 2 | G2 | 5 | 7thStage IIBT3N1MX | IIB | 4 | II | II | T3 | 3 | T3+T4 | N1 | 1 | Dead | 1130 | 2.405274 | High | 2 | -0.51 | TRUE | 0.393839 | 0.264378 | 2 | 2 | ######## |
| TCGA-HZ-A9TJ | Pancreas-Adenocarcinoma-Other Subtype | Tail of Pancreas | MALE | 2 | 70 | >65 | 2 | G2 | 2 | G2 | 8 | 7thStage IVT3N0M1 | IV | 6 | IV | III+IV | T3 | 3 | T3+T4 | N0 | 0 | Alive | 603 | 0.553259 | Low | 1 | -0.24 | TRUE | 0.540712 | 0.24011 | 2 | 2 | 0.0123 |
| TCGA-IB-7897 | Pancreas-Adenocarcinoma Ductal Type | Head of Pancreas | FEMALE | 1 | 53 | 65<= | 1 | G2 | 2 | G2 | 3.5 | 6thStage IIBT3N1M0 | IIB | 4 | II | II | T3 | 3 | T3+T4 | N1 | 1 | Dead | 486 | -12.8045 | Low | 1 | 0.09 | FALSE | 0.234936 | 0.244993 | 1 | 2 | 0.128 |
| TCGA-3A-A9I5 | Pancreas-Adenocarcinoma Ductal Type | Other (please specify) | MALE | 2 | 57 | 65<= | 1 | G1 | 1 | G1 | 3 | 6thN0MX | IB | 2 | I | I | T2 | 2 | T1+T2 | N0 | 0 | Alive | 1612 | -4.88872 | Low | 1 | 0.88 | FALSE | 0.514996 | 0.301837 | 2 | 1 | 0 |
| TCGA-HZ-8315 | Pancreas-Adenocarcinoma Ductal Type | Head of Pancreas | FEMALE | 1 | 54 | 65<= | 1 | G2 | 2 | G2 | 6 | 7thStage IIAT3N0MX | IIA | 3 | II | II | T3 | 3 | T3+T4 | N0 | 0 | Alive | 28 | 3.849409 | High | 2 | -0.18 | TRUE | 0.139596 | 0.259404 | 2 | 3 | 0.0176 |
| TCGA-HZ-A8P1 | Pancreas-Adenocarcinoma Ductal Type | Tail of Pancreas | MALE | 2 | 81 | >65 | 2 | G1 | 1 | G1 | 5 | 7thStage IBT2N0MX | IB | 2 | I | I | T2 | 2 | T1+T2 | N0 | 0 | Alive | 7 | 1.655211 | High | 2 | -0.76 | TRUE | 0.654136 | 0.130367 | 2 | 2 | 0.0174 |
| TCGA-IB-A7M4 | Pancreas-Adenocarcinoma Ductal Type | Body of Pancreas | MALE | 2 | 81 | >65 | 2 | G3 | 3 | G3+G4 | NA | 7thStage IIBT3N1MX | IIB | 4 | II | II | T3 | 3 | T3+T4 | N1 | 0 | Alive | 181 | 9.07223 | High | 2 | -0.07 | TRUE | 0.676717 | 0.284885 | 2 | 3 | 0.0189 |
| TCGA-IB-7647 | Pancreas-Adenocarcinoma Ductal Type | Head of Pancreas | MALE | 2 | 41 | 65<= | 1 | G2 | 2 | G2 | 6 | 6thStage IIBT3N1M0 | IIB | 4 | II | II | T3 | 3 | T3+T4 | N1 | 1 | Dead | 666 | -2.19653 | Low | 1 | -0.16 | TRUE | 0.553334 | 0.226021 | 2 | 2 | 0.00795 |
| TCGA-FB-AAPZ | Pancreas-Adenocarcinoma Ductal Type | Head of Pancreas | MALE | 2 | 54 | 65<= | 1 | G3 | 3 | G3+G4 | 3.8 | 7thStage IIBT2N1MX | IIB | 4 | II | II | T2 | 2 | T1+T2 | N1 | 0 | Alive | 226 | 4.687154 | High | 2 | -0.02 | TRUE | 0.302392 | 0.099475 | 1 | 3 | 0.0262 |
| TCGA-RB-AA9M | Pancreas-Adenocarcinoma Ductal Type | Tail of Pancreas | MALE | 2 | 43 | 65<= | 1 | G3 | 3 | G3+G4 | 2 | 7thStage IIBT1N1MX | IIB | 4 | II | II | T1 | 1 | T1+T2 | N1 | 0 | Alive | 42 | -0.6768 | Low | 1 | 1.09 | FALSE | 0.27051 | 0.251789 | 1 | 3 | 0.0174 |
| TCGA-HZ-8638 | Pancreas-Colloid (mucinous non-cystic) Carcinoma | Head of Pancreas | FEMALE | 1 | 84 | >65 | 2 | G4 | 4 | G3+G4 | 0.3 | 7thStage IAT1N0MX | IA | 1 | I | I | T1 | 1 | T1+T2 | N0 | 0 | Alive | 91 | -0.4369 | Low | 1 | -0.83 | TRUE | 0.420426 | 0.163935 | 2 | 1 | 0.0332 |
| TCGA-LB-A8F3 | Pancreas-Adenocarcinoma Ductal Type | Head of Pancreas | FEMALE | 1 | 64 | 65<= | 1 | G1 | 1 | G1 | 5 | 7thStage IIAT3N0MX | IIA | 3 | II | II | T3 | 3 | T3+T4 | N0 | 0 | Alive | 35 | -2.39536 | Low | 1 | 0.36 | FALSE | 0.446231 | 0.147954 | 2 | 1 | 1.94E-18 |
| TCGA-IB-A7LX | Pancreas-Adenocarcinoma Ductal Type | Head of Pancreas | MALE | 2 | 57 | 65<= | 1 | G2 | 2 | G2 | 5.5 | 7thStage IIBT3N1MX | IIB | 4 | II | II | T3 | 3 | T3+T4 | N1 | 1 | Dead | 250 | 11.69749 | High | 2 | 0.6 | FALSE | 0.376552 | 0.329017 | 2 | 1 | 0.0282 |
| TCGA-IB-7888 | Pancreas-Adenocarcinoma Ductal Type | Head of Pancreas | FEMALE | 1 | 66 | >65 | 2 | G2 | 2 | G2 | 3 | 6thStage IIAT3N0M0 | IIA | 3 | II | II | T3 | 3 | T3+T4 | N0 | 1 | Dead | 1332 | -8.37234 | Low | 1 | 0.39 | FALSE | 0.383459 | 0.237919 | 1 | 2 | 0.0131 |
| TCGA-S4-A8RM | Pancreas-Adenocarcinoma Ductal Type | Other (please specify) | MALE | 2 | 67 | >65 | 2 | G3 | 3 | G3+G4 | 6 | 7thStage IIBT3N1MX | IIB | 4 | II | II | T3 | 3 | T3+T4 | N1 | 0 | Alive | 397 | 0.161182 | Low | 1 | -1.35 | TRUE | 0.493534 | 0.135084 | 2 | 2 | 0.0504 |
| TCGA-FB-AAPS | Pancreas-Adenocarcinoma Ductal Type | Head of Pancreas | FEMALE | 1 | 62 | 65<= | 1 | G2 | 2 | G2 | 3 | 7thStage IIBT2N1MX | IIB | 4 | II | II | T2 | 2 | T1+T2 | N1 | 0 | Alive | 228 | 0.334459 | Low | 1 | 1.05 | FALSE | 0.013824 | 0.359496 | 1 | 1 | 0.0374 |
| TCGA-IB-AAUO | Pancreas-Adenocarcinoma Ductal Type | Head of Pancreas | FEMALE | 1 | 64 | 65<= | 1 | G3 | 3 | G3+G4 | 2.7 | 7thStage IIBT3N1M0 | IIB | 4 | II | II | T3 | 3 | T3+T4 | N1 | 1 | Dead | 239 | 8.212712 | High | 2 | -0.17 | TRUE | 0.493428 | 0.250221 | 2 | 1 | 0.00234 |
| TCGA-3A-A9IB | Pancreas-Adenocarcinoma Ductal Type | Head of Pancreas | FEMALE | 1 | 69 | >65 | 2 | G3 | 3 | G3+G4 | 4 | 7thStage IIBT3N1MX | IIB | 4 | II | II | T3 | 3 | T3+T4 | N1 | 1 | Dead | 224 | 6.562071 | High | 2 | 1.85 | FALSE | 0.237437 | 0.400333 | 2 | 3 | ######## |
| TCGA-FB-AAPP | Pancreas-Adenocarcinoma Ductal Type | Head of Pancreas | MALE | 2 | 71 | >65 | 2 | G3 | 3 | G3+G4 | 5.5 | 7thStage IIBT3N1MX | IIB | 4 | II | II | T3 | 3 | T3+T4 | N1 | 1 | Dead | 485 | -1.77128 | Low | 1 | -1.03 | TRUE | 0.906196 | 0.403857 | 1 | 1 | 0.00593 |
| TCGA-F2-A7TX | Pancreas-Adenocarcinoma Ductal Type | Head of Pancreas | MALE | 2 | 77 | >65 | 2 | G3 | 3 | G3+G4 | 3.6 | 7thStage IIBT3N1M0 | IIB | 4 | II | II | T3 | 3 | T3+T4 | N1 | 1 | Dead | 95 | 4.602901 | High | 2 | -0.04 | TRUE | 0.66891 | 0.478228 | 2 | 3 | 0.0292 |
| TCGA-Q3-AA2A | Pancreas-Adenocarcinoma Ductal Type | Head of Pancreas | FEMALE | 1 | 64 | 65<= | 1 | G1 | 1 | G1 | NA | 7thStage IBT3N1MX | IIB | 4 | II | II | T3 | 3 | T3+T4 | N1 | 0 | Alive | 94 | 5.336673 | High | 2 | -0.41 | TRUE | 0.401269 | 0.238099 | 2 | 3 | 0 |
| TCGA-LB-A9Q5 | Pancreas-Adenocarcinoma Ductal Type | Head of Pancreas | FEMALE | 1 | 63 | 65<= | 1 | G3 | 3 | G3+G4 | 3 | 7thStage IIBT3N1MX | IIB | 4 | II | II | T3 | 3 | T3+T4 | N1 | 0 | Alive | 155 | -1.11971 | Low | 1 | -1.27 | TRUE | 0.358603 | 0.245009 | 2 | 3 | ######## |
| TCGA-XD-AAUH | Pancreas-Adenocarcinoma Ductal Type | Head of Pancreas | FEMALE | 1 | 57 | 65<= | 1 | G2 | 2 | G2 | 2.8 | 7thStage IIBT3N1M0 | IIB | 4 | II | II | T3 | 3 | T3+T4 | N1 | 0 | Alive | 164 | -12.0788 | Low | 1 | 0.99 | FALSE | 0.273449 | 0.281636 | 1 | 2 | 0.074 |
| TCGA-IB-7649 | Pancreas-Adenocarcinoma Ductal Type | Head of Pancreas | FEMALE | 1 | 73 | >65 | 2 | G2 | 2 | G2 | 4 | 6thStage IIBT3N1M0 | IIB | 4 | II | II | T3 | 3 | T3+T4 | N1 | 1 | Dead | 467 | -4.99166 | Low | 1 | -0.03 | TRUE | 0.336431 | 0.287951 | 1 | 2 | 0.00742 |
| TCGA-IB-A5SO | Pancreas-Adenocarcinoma Ductal Type | Head of Pancreas | MALE | 2 | 71 | >65 | 2 | G2 | 2 | G2 | 3.8 | 7thStage IIBT3N1M0 | IIB | 4 | II | II | T3 | 3 | T3+T4 | N1 | 0 | Alive | 329 | -0.50111 | Low | 1 | 0.25 | FALSE | 0.162357 | 0.336427 | 1 | 2 | 0.019 |
| TCGA-3A-A9IJ | Pancreas-Adenocarcinoma-Other Subtype | Head of Pancreas | MALE | 2 | 65 | 65<= | 1 | G1 | 1 | G1 | 3.5 | 6thStage IBT2N0MX | IB | 2 | I | I | T2 | 2 | T1+T2 | N0 | 0 | Alive | 1854 | -24.8509 | Low | 1 | -0.09 | TRUE | 0.605449 | 0.257771 | 1 | 2 | 0.0328 |
| TCGA-FB-A5VM | Pancreas-Adenocarcinoma Ductal Type | Head of Pancreas | MALE | 2 | 74 | >65 | 2 | G3 | 3 | G3+G4 | 4.5 | 7thStage IBT2N0M0 | IB | 2 | I | I | T2 | 2 | T1+T2 | N0 | 0 | Alive | 75 | 9.770589 | High | 2 | 0.58 | FALSE | 0.623046 | 0.456527 | 2 | 1 | 0.00114 |
| TCGA-IB-8127 | Pancreas-Adenocarcinoma Ductal Type | Head of Pancreas | MALE | 2 | 59 | 65<= | 1 | G2 | 2 | G2 | 4.5 | 7thStage IIBT3N1M0 | IIB | 4 | II | II | T3 | 3 | T3+T4 | N1 | 0 | Alive | 194 | 5.253328 | High | 2 | -0.03 | TRUE | 0.31226 | 0.223846 | 2 | 2 | 0.0284 |
| TCGA-3E-AAAZ | Pancreas-Adenocarcinoma Ductal Type | Head of Pancreas | MALE | 2 | 71 | >65 | 2 | G2 | 2 | G2 | 3.2 | 6thStage IIAT3N0MX | IIA | 3 | II | II | T3 | 3 | T3+T4 | N0 | 1 | Dead | 2182 | 3.854741 | High | 2 | 1.57 | FALSE | 0.396945 | 0.305414 | 2 | 1 | 0.0152 |
| TCGA-2J-AABU | Pancreas-Adenocarcinoma Ductal Type | Head of Pancreas | MALE | 2 | 56 | 65<= | 1 | G3 | 3 | G3+G4 | 3.3 | 7thStage IIBT3N1M0 | IIB | 4 | II | II | T3 | 3 | T3+T4 | N1 | 1 | Dead | 277 | 10.16516 | High | 2 | -0.06 | TRUE | 0.306244 | 0.560209 | 2 | 1 | 0.0174 |
| TCGA-HV-AA8V | Pancreas-Adenocarcinoma Ductal Type | Head of Pancreas | MALE | 2 | 50 | 65<= | 1 | G3 | 3 | G3+G4 | NA | 7thStage IIBT3N1MX | IIB | 4 | II | II | T3 | 3 | T3+T4 | N1 | 0 | Alive | 910 | 4.299879 | High | 2 | 1.94 | FALSE | 0.059312 | 0.33605 | 2 | 3 | 0.00612 |
| TCGA-IB-AAUU | Pancreas-Adenocarcinoma Ductal Type | Head of Pancreas | MALE | 2 | 35 | 65<= | 1 | G3 | 3 | G3+G4 | 3 | 7thStage IIBT3N1M0 | IIB | 4 | II | II | T3 | 3 | T3+T4 | N1 | 0 | Alive | 153 | 2.368834 | High | 2 | -1.03 | TRUE | 0.39752 | 0.372765 | 2 | 2 | 0.00265 |
| TCGA-IB-A5SS | Pancreas-Adenocarcinoma Ductal Type | Tail of Pancreas | FEMALE | 1 | 64 | 65<= | 1 | G3 | 3 | G3+G4 | 4.5 | 7thStage IIBT3N1M0 | IIB | 4 | II | II | T3 | 3 | T3+T4 | N1 | 1 | Dead | 460 | 10.86277 | High | 2 | 1.85 | FALSE | 0.287067 | 0.316789 | 2 | 1 | ######## |
| TCGA-3A-A9IU | Pancreas-Adenocarcinoma Ductal Type | Head of Pancreas | MALE | 2 | 65 | 65<= | 1 | G3 | 3 | G3+G4 | 4 | 7thStage IIBT3N1MX | IIB | 4 | II | II | T3 | 3 | T3+T4 | N1 | 1 | Dead | 458 | 8.315888 | High | 2 | 0.43 | FALSE | 0.311412 | 0.279108 | 2 | 3 | 0.00259 |
| TCGA-3A-A9IN | Pancreas-Adenocarcinoma-Other Subtype | Body of Pancreas | FEMALE | 1 | 62 | 65<= | 1 | G2 | 2 | G2 | 5 | 6thStage IBT2NXMX | IB | 2 | I | I | T2 | 2 | T1+T2 | N0 | 0 | Alive | 2084 | -22.439 | Low | 1 | -0.01 | TRUE | 0.213025 | 0.575609 | 1 | 2 | 0.00581 |
| TCGA-FB-A7DR | Pancreas-Colloid (mucinous non-cystic) Carcinoma | Head of Pancreas | MALE | 2 | 48 | 65<= | 1 | G2 | 2 | G2 | 3 | 7thStage IIAT3NXM0 | IIA | 3 | II | II | T3 | 3 | T3+T4 | N0 | 0 | Alive | 166 | 2.978812 | High | 2 | 1.54 | FALSE | 0.15257 | 0.258001 | 1 | 1 | 0 |
| TCGA-FB-AAQ0 | Pancreas-Adenocarcinoma Ductal Type | Head of Pancreas | MALE | 2 | 68 | >65 | 2 | G3 | 3 | G3+G4 | 2.7 | 7thStage IIAT3N0MX | IIA | 3 | II | II | T3 | 3 | T3+T4 | N0 | 1 | Dead | 473 | 5.690164 | High | 2 | 0.08 | FALSE | 0.484342 | 0.398795 | 2 | 2 | 0.0105 |
| TCGA-3A-A9IR | Pancreas-Adenocarcinoma-Other Subtype | Tail of Pancreas | FEMALE | 1 | 64 | 65<= | 1 | G1 | 1 | G1 | 4 | 7thStage IBT2N0MX | IB | 2 | I | I | T2 | 2 | T1+T2 | N0 | 0 | Alive | 1164 | -24.3305 | Low | 1 | -0.85 | TRUE | 0.638761 | 0 | 1 | 2 | 0.0796 |
| TCGA-IB-7891 | Pancreas-Adenocarcinoma Ductal Type | Head of Pancreas | FEMALE | 1 | 49 | 65<= | 1 | G1 | 1 | G1 | 2.3 | 7thStage IIBT3N1M0 | IIB | 4 | II | II | T3 | 3 | T3+T4 | N1 | 0 | Alive | 488 | -5.41307 | Low | 1 | 0.08 | FALSE | 0.370099 | 0.22046 | 1 | 2 | 0.0262 |
| TCGA-2J-AABI | Pancreas-Adenocarcinoma-Other Subtype | Head of Pancreas | FEMALE | 1 | 55 | 65<= | 1 | G3 | 3 | G3+G4 | 3.5 | 7thStage IIAT3N0M0 | IIA | 3 | II | II | T3 | 3 | T3+T4 | N0 | 0 | Alive | 330 | 7.505995 | High | 2 | 0.51 | FALSE | 0.456214 | 0.378158 | 2 | 1 | 0.00416 |
| TCGA-IB-AAUS | Pancreas-Adenocarcinoma Ductal Type | Other (please specify) | FEMALE | 1 | 84 | >65 | 2 | G2 | 2 | G2 | 4 | 7thStage IIBT3N1M0 | IIB | 4 | II | II | T3 | 3 | T3+T4 | N1 | 0 | Alive | 179 | 1.780984 | High | 2 | 1.27 | FALSE | 0.185201 | 0.168108 | 1 | 3 | 0.0397 |
| TCGA-FB-AAPY | Pancreas-Adenocarcinoma Ductal Type | Head of Pancreas | MALE | 2 | 71 | >65 | 2 | G2 | 2 | G2 | 3.5 | 7thStage IIBT2N1MX | IIB | 4 | II | II | T2 | 2 | T1+T2 | N1 | 1 | Dead | 1059 | 0.049321 | Low | 1 | -1.75 | TRUE | 0.374217 | 0.334691 | 2 | 2 | 0.0177 |
| TCGA-F2-A44G | Pancreas-Adenocarcinoma Ductal Type | Head of Pancreas | FEMALE | 1 | 79 | >65 | 2 | G2 | 2 | G2 | 3.5 | 7thStage IIBT3N1M0 | IIB | 4 | II | II | T3 | 3 | T3+T4 | N1 | 0 | Alive | 153 | 8.393413 | High | 2 | 0.34 | FALSE | 0.467559 | 0.169952 | 2 | 3 | ######## |
| TCGA-HV-A5A3 | Pancreas-Adenocarcinoma Ductal Type | Body of Pancreas | MALE | 2 | 50 | 65<= | 1 | G2 | 2 | G2 | NA | 7thStage IIAT3NXMX | IIA | 3 | II | II | T3 | 3 | T3+T4 | N0 | 1 | Dead | 128 | 9.992893 | High | 2 | 0.1 | FALSE | 0.324896 | 0.20938 | 2 | 1 | 3.54E-22 |
| TCGA-US-A774 | Pancreas-Adenocarcinoma Ductal Type | Head of Pancreas | FEMALE | 1 | 76 | >65 | 2 | G3 | 3 | G3+G4 | 4 | 7thStage IIBT3N1MX | IIB | 4 | II | II | T3 | 3 | T3+T4 | N1 | 1 | Dead | 695 | 0.74308 | Low | 1 | 0.69 | FALSE | 0.293697 | 0.456965 | 2 | 2 | 0.0113 |
| TCGA-IB-A5SP | Pancreas-Adenocarcinoma Ductal Type | Head of Pancreas | MALE | 2 | 77 | >65 | 2 | G2 | 2 | G2 | 3 | 7thStage IIAT3N0M0 | IIA | 3 | II | II | T3 | 3 | T3+T4 | N0 | 0 | Alive | 300 | -0.10676 | Low | 1 | -1.51 | TRUE | 0.671535 | 0.592824 | 2 | 2 | 2.90E-19 |
| TCGA-US-A77E | Pancreas-Adenocarcinoma-Other Subtype | Head of Pancreas | MALE | 2 | 73 | >65 | 2 | G3 | 3 | G3+G4 | 3.5 | 7thStage IIBT3N1MX | IIB | 4 | II | II | T3 | 3 | T3+T4 | N1 | 1 | Dead | 430 | 3.439275 | High | 2 | 1.01 | FALSE | 0.230056 | 0.518413 | 2 | 2 | 0 |
| TCGA-S4-A8RP | Pancreas-Adenocarcinoma Ductal Type | Head of Pancreas | FEMALE | 1 | 77 | >65 | 2 | G3 | 3 | G3+G4 | 2.8 | 7thStage IIBT3N1MX | IIB | 4 | II | II | T3 | 3 | T3+T4 | N1 | 1 | Dead | 702 | -0.63467 | Low | 1 | -0.16 | TRUE | 0.244028 | 0.188952 | 1 | 2 | 0.0291 |
| TCGA-M8-A5N4 | Pancreas-Adenocarcinoma Ductal Type | Head of Pancreas | FEMALE | 1 | 48 | 65<= | 1 | G2 | 2 | G2 | 3.4 | 6thStage IIAT3N0M0 | IIA | 3 | II | II | T3 | 3 | T3+T4 | N0 | 0 | Alive | 584 | 7.253379 | High | 2 | -0.47 | TRUE | 0.276848 | 0.160094 | 2 | 3 | 0.00459 |
| TCGA-HZ-8001 | Pancreas-Adenocarcinoma Ductal Type | Head of Pancreas | MALE | 2 | 69 | >65 | 2 | G2 | 2 | G2 | 3 | 7thStage IIIT4N0MX | III | 5 | III | III+IV | T4 | 4 | T3+T4 | N0 | 0 | Alive | 19 | -2.39099 | Low | 1 | 0.82 | FALSE | 0.344843 | 0.224273 | 1 | 1 | 0.03 |
| TCGA-3A-A9I7 | Pancreas-Adenocarcinoma Ductal Type | Head of Pancreas | MALE | 2 | 45 | 65<= | 1 | G2 | 2 | G2 | NA | 7thStage IIBT3N1MX | IIB | 4 | II | II | T3 | 3 | T3+T4 | N1 | 0 | Alive | 718 | 2.811638 | High | 2 | 0.21 | FALSE | 0.208351 | 0.374372 | 2 | 2 | 0.00948 |
| TCGA-F2-A8YN | Pancreas-Adenocarcinoma Ductal Type | Head of Pancreas | MALE | 2 | 76 | >65 | 2 | G2 | 2 | G2 | 4.8 | 7thStage IIAT3N0M0 | IIA | 3 | II | II | T3 | 3 | T3+T4 | N0 | 0 | Alive | 167 | 7.166914 | High | 2 | 0.73 | FALSE | 0.391571 | 0.411274 | 2 | 1 | ######## |
| TCGA-IB-7645 | Pancreas-Adenocarcinoma Ductal Type | Head of Pancreas | FEMALE | 1 | 44 | 65<= | 1 | G1 | 1 | G1 | 4.5 | 6thStage IIBT3N1M0 | IIB | 4 | II | II | T3 | 3 | T3+T4 | N1 | 1 | Dead | 1502 | -4.13677 | Low | 1 | 0.29 | FALSE | 0.262043 | 0.189863 | 1 | 2 | 0.0582 |
| TCGA-IB-7890 | Pancreas-Adenocarcinoma Ductal Type | Tail of Pancreas | MALE | 2 | 73 | >65 | 2 | G3 | 3 | G3+G4 | 2.8 | 6thStage IBT2N0M0 | IB | 2 | I | I | T2 | 2 | T1+T2 | N0 | 1 | Dead | 598 | 3.819146 | High | 2 | 2.16 | FALSE | 0.134884 | 0.299916 | 2 | 1 | 0.00618 |
| TCGA-US-A776 | Pancreas-Colloid (mucinous non-cystic) Carcinoma | Head of Pancreas | MALE | 2 | 61 | 65<= | 1 | G2 | 2 | G2 | 3 | 7thStage IIAT3N0MX | IIA | 3 | II | II | T3 | 3 | T3+T4 | N0 | 0 | Alive | 844 | -3.01902 | Low | 1 | -0.93 | TRUE | 0.856433 | 0.735201 | 1 | 1 | 0.0125 |
| TCGA-FB-A4P5 | Pancreas-Adenocarcinoma Ductal Type | Head of Pancreas | FEMALE | 1 | 69 | >65 | 2 | G2 | 2 | G2 | 4.5 | 7thStage IIBT3N1MX | IIB | 4 | II | II | T3 | 3 | T3+T4 | N1 | 0 | Alive | 4 | -1.24152 | Low | 1 | 1.72 | FALSE | 0.339101 | 0.327762 | 1 | 3 | 0.092 |
| TCGA-HZ-7926 | Pancreas-Adenocarcinoma Ductal Type | Head of Pancreas | MALE | 2 | 57 | 65<= | 1 | G1 | 1 | G1 | 3.5 | 7thStage IIBT3N1MX | IIB | 4 | II | II | T3 | 3 | T3+T4 | N1 | 0 | Alive | 8 | 3.218212 | High | 2 | -0.6 | TRUE | 0.373734 | 0.240442 | 1 | 3 | 0.0125 |
| TCGA-IB-A6UF | Pancreas-Adenocarcinoma Ductal Type | Head of Pancreas | MALE | 2 | 63 | 65<= | 1 | G2 | 2 | G2 | 3.3 | 7thStage IIBT3N1M0 | IIB | 4 | II | II | T3 | 3 | T3+T4 | N1 | 0 | Alive | 248 | 4.954491 | High | 2 | 0.68 | FALSE | 0.326538 | 0.285876 | 2 | 1 | 0.000364 |
| TCGA-HZ-7918 | Pancreas-Adenocarcinoma Ductal Type | Head of Pancreas | MALE | 2 | 72 | >65 | 2 | G3 | 3 | G3+G4 | 5.5 | 7thStage IIBT3N1M0 | IIB | 4 | II | II | T3 | 3 | T3+T4 | N1 | 0 | Alive | 28 | -6.85401 | Low | 1 | -0.11 | TRUE | 0.506056 | 0.325265 | 2 | 2 | 0.0657 |
| TCGA-3A-A9IH | Pancreas-Adenocarcinoma Ductal Type | Body of Pancreas | FEMALE | 1 | 66 | >65 | 2 | G2 | 2 | G2 | 2 | 7thStage IAT1N0MX | IA | 1 | I | I | T1 | 1 | T1+T2 | N0 | 0 | Alive | 874 | 3.574073 | High | 2 | 0.37 | FALSE | 0.398407 | 0.345417 | 2 | 3 | ######## |
| TCGA-FB-A78T | Pancreas-Adenocarcinoma Ductal Type | Head of Pancreas | FEMALE | 1 | 71 | >65 | 2 | G2 | 2 | G2 | 3.5 | 7thStage IIBT3N1M0 | IIB | 4 | II | II | T3 | 3 | T3+T4 | N1 | 0 | Alive | 1 | 0.383713 | Low | 1 | -1.92 | TRUE | 0.492108 | 0.312671 | 2 | 2 | 0.00401 |
| TCGA-2L-AAQA | Pancreas-Adenocarcinoma Ductal Type | Head of Pancreas | MALE | 2 | 76 | >65 | 2 | G2 | 2 | G2 | 12 | 7thStage IIBT3N1MX | IIB | 4 | II | II | T3 | 3 | T3+T4 | N1 | 1 | Dead | 143 | 9.334669 | High | 2 | 0.28 | FALSE | 0.519949 | 0.303643 | 2 | 3 | 0.00363 |
| TCGA-2J-AABE | Pancreas-Adenocarcinoma Ductal Type | Body of Pancreas | MALE | 2 | 73 | >65 | 2 | G2 | 2 | G2 | 5.8 | 7thStage IIAT3N0M0 | IIA | 3 | II | II | T3 | 3 | T3+T4 | N0 | 0 | Alive | 663 | 4.874189 | High | 2 | 0.53 | FALSE | 0.21734 | 0.317432 | 2 | 2 | ######## |
| TCGA-2L-AAQI | Pancreas-Adenocarcinoma Ductal Type | Head of Pancreas | MALE | 2 | 66 | >65 | 2 | G3 | 3 | G3+G4 | 4 | 7thStage IIBT3N1MX | IIB | 4 | II | II | T3 | 3 | T3+T4 | N1 | 1 | Dead | 103 | 2.87632 | High | 2 | -0.22 | TRUE | 0.438509 | 0.271887 | 2 | 3 | 4.38E-19 |
| TCGA-IB-7651 | Pancreas-Adenocarcinoma Ductal Type | Head of Pancreas | FEMALE | 1 | 64 | 65<= | 1 | G2 | 2 | G2 | 2.9 | 6thStage IIBT3N1M0 | IIB | 4 | II | II | T3 | 3 | T3+T4 | N1 | 1 | Dead | 603 | -1.23196 | Low | 1 | -0.08 | TRUE | 0.488887 | 0.419392 | 2 | 2 | 0.0287 |
| TCGA-3A-A9IX | Pancreas-Adenocarcinoma Ductal Type | Head of Pancreas | MALE | 2 | 40 | 65<= | 1 | G2 | 2 | G2 | 2 | 7thStage IAT1N0MX | IA | 1 | I | I | T1 | 1 | T1+T2 | N0 | 0 | Alive | 901 | -3.30084 | Low | 1 | -0.28 | TRUE | 0.21435 | 0.306564 | 1 | 2 | 0.0349 |
| TCGA-F2-7273 | Pancreas-Adenocarcinoma Ductal Type | Head of Pancreas | MALE | 2 | 54 | 65<= | 1 | G3 | 3 | G3+G4 | 4.2 | 7thStage IIBT3N1M0 | IIB | 4 | II | II | T3 | 3 | T3+T4 | N1 | 0 | Alive | 360 | -9.27622 | Low | 1 | -0.36 | TRUE | 0.213179 | 0.201297 | 1 | 2 | 0.025 |
| TCGA-2J-AAB8 | Pancreas-Adenocarcinoma Ductal Type | Head of Pancreas | MALE | 2 | 71 | >65 | 2 | G3 | 3 | G3+G4 | 3.5 | 7thStage IIBT3N1M0 | IIB | 4 | II | II | T3 | 3 | T3+T4 | N1 | 0 | Alive | 80 | 6.684426 | High | 2 | 1.79 | FALSE | 0.330979 | 0.30959 | 2 | 3 | 0.0258 |
| TCGA-HZ-8636 | Pancreas-Adenocarcinoma Ductal Type | Tail of Pancreas | FEMALE | 1 | 58 | 65<= | 1 | G3 | 3 | G3+G4 | 9 | 7thStage IVT3N0M1 | IV | 6 | IV | III+IV | T3 | 3 | T3+T4 | N0 | 0 | Alive | 5 | 1.874901 | High | 2 | 0.8 | FALSE | 0.252337 | 0.281145 | 2 | 2 | 0.0127 |
| TCGA-YH-A8SY | | Head of Pancreas | FEMALE | 1 | 73 | >65 | 2 | G2 | 2 | G2 | 4.1 | 7thT3N1MX | IIB | 4 | II | II | T3 | 3 | T3+T4 | N1 | 0 | Alive | 388 | 10.76341 | High | 2 | 2.41 | FALSE | 0.220788 | 0.416516 | 2 | 1 | 0 |
| TCGA-F2-6880 | Pancreas-Adenocarcinoma Ductal Type | Head of Pancreas | MALE | 2 | 70 | >65 | 2 | G1 | 1 | G1 | 2.2 | 6thStage IIBT3N1M0 | IIB | 4 | II | II | T3 | 3 | T3+T4 | N1 | 0 | Alive | 295 | -12.8017 | Low | 1 | -1.58 | TRUE | 0.522097 | 0.240711 | 1 | 2 | 0.00567 |
| TCGA-2J-AABO | Pancreas-Adenocarcinoma Ductal Type | Head of Pancreas | MALE | 2 | 43 | 65<= | 1 | G2 | 2 | G2 | 4.6 | 7thStage IIBT3N1M0 | IIB | 4 | II | II | T3 | 3 | T3+T4 | N1 | 0 | Alive | 345 | 4.700354 | High | 2 | 2.03 | FALSE | 0.249923 | 0.405226 | 1 | 3 | 0.014 |
| TCGA-IB-A6UG | Pancreas-Adenocarcinoma Ductal Type | Head of Pancreas | MALE | 2 | 65 | 65<= | 1 | G3 | 3 | G3+G4 | 3 | 7thStage IIBT3N1M0 | IIB | 4 | II | II | T3 | 3 | T3+T4 | N1 | 1 | Dead | 41 | 2.246699 | High | 2 | -0.49 | TRUE | 0.412819 | 0.340976 | 2 | 1 | 0.00658 |
| TCGA-2J-AABP | Pancreas-Undifferentiated Carcinoma | Tail of Pancreas | FEMALE | 1 | 58 | 65<= | 1 | G4 | 4 | G3+G4 | 7.2 | 7thStage IIBT3N1M0 | IIB | 4 | II | II | T3 | 3 | T3+T4 | N1 | 0 | Alive | 355 | -0.95041 | Low | 1 | 0.06 | FALSE | 0.555621 | 0.721455 | 1 | 1 | 6.88E-20 |
| TCGA-US-A779 | Pancreas-Adenocarcinoma Ductal Type | Head of Pancreas | FEMALE | 1 | 54 | 65<= | 1 | G1 | 1 | G1 | 3 | 7thStage IIBT3N1bMX | IIB | 4 | II | II | T3 | 3 | T3+T4 | N1 | 1 | Dead | 511 | 0.43969 | Low | 1 | -0.87 | TRUE | 0.591003 | 0.379448 | 2 | 2 | 2.58E-19 |
| TCGA-FB-A545 | Pancreas-Adenocarcinoma Ductal Type | Body of Pancreas | FEMALE | 1 | 72 | >65 | 2 | G2 | 2 | G2 | NA | 7thStage IIBT3N1M0 | IIB | 4 | II | II | T3 | 3 | T3+T4 | N1 | 0 | Alive | 1 | 9.150386 | High | 2 | 1.91 | FALSE | 0.337627 | 0.127436 | 2 | 2 | 2.80E-21 |
| TCGA-LB-A7SX | Pancreas-Adenocarcinoma Ductal Type | Head of Pancreas | FEMALE | 1 | 74 | >65 | 2 | G2 | 2 | G2 | 2.5 | 7thStage IIBT3N1MX | IIB | 4 | II | II | T3 | 3 | T3+T4 | N1 | 0 | Alive | 127 | 8.434012 | High | 2 | -0.21 | TRUE | 0.580474 | 0.921341 | 2 | 1 | 0.0131 |
| TCGA-HV-A5A5 | Pancreas-Adenocarcinoma Ductal Type | Head of Pancreas | FEMALE | 1 | 61 | 65<= | 1 | G2 | 2 | G2 | NA | 7thStage IIBT3N1M0 | IIB | 4 | II | II | T3 | 3 | T3+T4 | N1 | 0 | Alive | 289 | -5.3829 | Low | 1 | -0.71 | TRUE | 0.300056 | 0.132189 | 1 | 2 | 0.004 |
| TCGA-HZ-8003 | Pancreas-Adenocarcinoma Ductal Type | Head of Pancreas | FEMALE | 1 | 65 | 65<= | 1 | G2 | 2 | G2 | 2.8 | 7thStage IIBT3N1MX | IIB | 4 | II | II | T3 | 3 | T3+T4 | N1 | 0 | Alive | 21 | -7.26286 | Low | 1 | 0.5 | FALSE | 0.386624 | 0.212302 | 2 | 2 | 0.0329 |
| TCGA-IB-AAUQ | Pancreas-Adenocarcinoma Ductal Type | Tail of Pancreas | MALE | 2 | 50 | 65<= | 1 | G2 | 2 | G2 | 4.5 | 7thStage IIBT3N1M0 | IIB | 4 | II | II | T3 | 3 | T3+T4 | N1 | 1 | Dead | 183 | 1.602062 | High | 2 | 1.59 | FALSE | 0.230553 | 0.477262 | 1 | 1 | 0.000207 |
| TCGA-FB-AAQ1 | Pancreas-Adenocarcinoma Ductal Type | Head of Pancreas | MALE | 2 | 49 | 65<= | 1 | G2 | 2 | G2 | 6 | 7thStage IIBT3N1MX | IIB | 4 | II | II | T3 | 3 | T3+T4 | N1 | 1 | Dead | 123 | 3.72476 | High | 2 | -0.06 | TRUE | 0.507932 | 0.258834 | 2 | 1 | ######## |
| TCGA-HZ-8317 | Pancreas-Adenocarcinoma Ductal Type | Head of Pancreas | FEMALE | 1 | 69 | >65 | 2 | G1 | 1 | G1 | 3.1 | 7thStage IIBT3N1MX | IIB | 4 | II | II | T3 | 3 | T3+T4 | N1 | 0 | Alive | 16 | 1.280461 | High | 2 | 0.52 | FALSE | 0.271373 | 0.183957 | 2 | 1 | 3.15E-18 |
| TCGA-2J-AABA | Pancreas-Adenocarcinoma Ductal Type | Head of Pancreas | MALE | 2 | 55 | 65<= | 1 | G2 | 2 | G2 | 4.3 | 7thStage IIBT3N1M0 | IIB | 4 | II | II | T3 | 3 | T3+T4 | N1 | 1 | Dead | 607 | 5.294052 | High | 2 | 1.38 | FALSE | 0.230767 | 0.447742 | 1 | 3 | 0.0264 |
| TCGA-2J-AABV | Pancreas-Adenocarcinoma Ductal Type | Head of Pancreas | MALE | 2 | 74 | >65 | 2 | G4 | 4 | G3+G4 | 4.1 | 7thStage IIBT3N1M0 | IIB | 4 | II | II | T3 | 3 | T3+T4 | N1 | 1 | Dead | 652 | -4.66581 | Low | 1 | -1.28 | TRUE | 0.648523 | 0.41786 | 1 | 2 | 6.05E-19 |
| TCGA-2L-AAQM | Pancreas-Adenocarcinoma-Other Subtype | Other (please specify) | MALE | 2 | 52 | 65<= | 1 | G1 | 1 | G1 | 14 | 7thStage IIBT3N1MX | IIB | 4 | II | II | T3 | 3 | T3+T4 | N1 | 0 | Alive | 914 | -23.7904 | Low | 1 | -0.36 | TRUE | 0.325828 | 1 | 1 | 2 | 4.97E-19 |
| TCGA-H6-8124 | Pancreas-Adenocarcinoma Ductal Type | Head of Pancreas | FEMALE | 1 | 56 | 65<= | 1 | G3 | 3 | G3+G4 | 3 | 7thStage IIBT3N1M0 | IIB | 4 | II | II | T3 | 3 | T3+T4 | N1 | 0 | Alive | 181 | 6.495622 | High | 2 | 2.51 | FALSE | 0.272919 | 0.401594 | 2 | 2 | 0.0249 |
| TCGA-XD-AAUG | Pancreas-Adenocarcinoma Ductal Type | Other (please specify) | FEMALE | 1 | 66 | >65 | 2 | G2 | 2 | G2 | 3.2 | 7thStage IVT3N1M1 | IV | 6 | IV | III+IV | T3 | 3 | T3+T4 | N1 | 0 | Alive | 161 | -9.52655 | Low | 1 | 2.22 | FALSE | 0.003345 | 0.278466 | 1 | 2 | 0.0353 |
| TCGA-IB-7887 | Pancreas-Adenocarcinoma Ductal Type | Head of Pancreas | FEMALE | 1 | 62 | 65<= | 1 | G2 | 2 | G2 | 5.5 | 7thStage IIBT3N1M0 | IIB | 4 | II | II | T3 | 3 | T3+T4 | N1 | 1 | Dead | 110 | 6.375614 | High | 2 | -0.67 | TRUE | 0.379334 | 0.208302 | 2 | 3 | 0.0138 |
| TCGA-IB-7652 | Pancreas-Adenocarcinoma Ductal Type | Body of Pancreas | FEMALE | 1 | 49 | 65<= | 1 | G2 | 2 | G2 | 4.2 | 7thStage IIBT3N1M0 | IIB | 4 | II | II | T3 | 3 | T3+T4 | N1 | 0 | Alive | 476 | -1.61015 | Low | 1 | -0.8 | TRUE | 0.325474 | 0.267346 | 2 | 2 | ######## |
| TCGA-IB-A5SQ | Pancreas-Adenocarcinoma Ductal Type | Head of Pancreas | FEMALE | 1 | 56 | 65<= | 1 | G2 | 2 | G2 | 2.5 | 7thStage IBT2N0M0 | IB | 2 | I | I | T2 | 2 | T1+T2 | N0 | 1 | Dead | 219 | -2.46261 | Low | 1 | 1.74 | FALSE | 0.271775 | 0.299782 | 1 | 1 | 0.0156 |
| TCGA-F2-7276 | Pancreas-Adenocarcinoma Ductal Type | Head of Pancreas | MALE | 2 | 64 | 65<= | 1 | G1 | 1 | G1 | 5.5 | 7thStage IIBT3N1M0 | IIB | 4 | II | II | T3 | 3 | T3+T4 | N1 | 1 | Dead | 216 | -5.97118 | Low | 1 | -0.1 | TRUE | 0.24609 | 0.221797 | 1 | 2 | 0.0229 |
| TCGA-HZ-A4BK | Pancreas-Adenocarcinoma-Other Subtype | Head of Pancreas | MALE | 2 | 72 | >65 | 2 | G3 | 3 | G3+G4 | 4.7 | 7thStage IIBT3N1MX | IIB | 4 | II | II | T3 | 3 | T3+T4 | N1 | 0 | Alive | 46 | 1.106705 | Low | 1 | 0.87 | FALSE | 0.28264 | 0.193132 | 1 | 2 | 0.0275 |
| TCGA-3A-A9IZ | Pancreas-Adenocarcinoma Ductal Type | Head of Pancreas | MALE | 2 | 47 | 65<= | 1 | G2 | 2 | G2 | 4 | 7thStage IIBT3N1MX | IIB | 4 | II | II | T3 | 3 | T3+T4 | N1 | 1 | Dead | 308 | 6.901336 | High | 2 | 0.96 | FALSE | 0.466995 | 0.590044 | 2 | 3 | ######## |
| TCGA-IB-7886 | Pancreas-Adenocarcinoma Ductal Type | Head of Pancreas | MALE | 2 | 80 | >65 | 2 | G3 | 3 | G3+G4 | 6 | 7thStage IIBT3N1M0 | IIB | 4 | II | II | T3 | 3 | T3+T4 | N1 | 1 | Dead | 123 | 3.6758 | High | 2 | 0.16 | FALSE | 0.423507 | 0.4362 | 2 | 2 | 0.0136 |
| TCGA-IB-AAUV | Pancreas-Adenocarcinoma Ductal Type | Head of Pancreas | MALE | 2 | 49 | 65<= | 1 | G2 | 2 | G2 | 4.8 | 7thStage IIBT3N1M0 | IIB | 4 | II | II | T3 | 3 | T3+T4 | N1 | 0 | Alive | 229 | -8.93643 | Low | 1 | 0.52 | FALSE | 0 | 0.328327 | 1 | 2 | 0.0794 |
| TCGA-2J-AAB4 | Pancreas-Adenocarcinoma-Other Subtype | Other (please specify) | MALE | 2 | 48 | 65<= | 1 | G2 | 2 | G2 | 6.8 | 7thStage IIBT2N1M0 | IIB | 4 | II | II | T2 | 2 | T1+T2 | N1 | 0 | Alive | 729 | 0.025106 | Low | 1 | -0.86 | TRUE | 0.34655 | 0.392936 | 2 | 2 | 0.000149 |
| TCGA-US-A77J | Pancreas-Adenocarcinoma Ductal Type | Head of Pancreas | FEMALE | 1 | 81 | >65 | 2 | G2 | 2 | G2 | 2.5 | 7thStage IIBT3N1bMX | IIB | 4 | II | II | T3 | 3 | T3+T4 | N1 | 1 | Dead | 568 | -5.4818 | Low | 1 | 1.78 | FALSE | 0.348371 | 0.204737 | 1 | 2 | 0.0178 |
| TCGA-Q3-A5QY | Pancreas-Adenocarcinoma Ductal Type | Head of Pancreas | MALE | 2 | 58 | 65<= | 1 | G2 | 2 | G2 | 2.7 | 7thStage IIBT3N1MX | IIB | 4 | II | II | T3 | 3 | T3+T4 | N1 | 0 | Alive | 105 | -0.30426 | Low | 1 | 1.77 | FALSE | 0.420818 | 0.28506 | 1 | 1 | 0.112 |
| TCGA-IB-7646 | Pancreas-Adenocarcinoma Ductal Type | Head of Pancreas | MALE | 2 | 60 | 65<= | 1 | G2 | 2 | G2 | 4.5 | 6thStage IIBT3N1M0 | IIB | 4 | II | II | T3 | 3 | T3+T4 | N1 | 1 | Dead | 145 | 7.338037 | High | 2 | 0.56 | FALSE | 0.473531 | 0.288074 | 2 | 1 | 3.46E-22 |
| TCGA-HZ-A77O | Pancreas-Adenocarcinoma Ductal Type | Head of Pancreas | FEMALE | 1 | 77 | >65 | 2 | G2 | 2 | G2 | 2.8 | 7thStage IIBT2N1MX | IIB | 4 | II | II | T2 | 2 | T1+T2 | N1 | 0 | Alive | 11 | 6.98402 | High | 2 | 0.37 | FALSE | 0.562153 | 0.440894 | 2 | 3 | ######## |
| TCGA-2J-AABK | Pancreas-Adenocarcinoma Ductal Type | Other (please specify) | MALE | 2 | 71 | >65 | 2 | G2 | 2 | G2 | 2.8 | 7thStage IIBT3N1M0 | IIB | 4 | II | II | T3 | 3 | T3+T4 | N1 | 0 | Alive | 484 | -5.55411 | Low | 1 | -1.27 | TRUE | 0.537196 | 0.264296 | 2 | 2 | ######## |
| TCGA-HV-A5A4 | Pancreas-Adenocarcinoma Ductal Type | Body of Pancreas | FEMALE | 1 | 72 | >65 | 2 | G2 | 2 | G2 | NA | 7thStage IIAT3N0MX | IIA | 3 | II | II | T3 | 3 | T3+T4 | N0 | 0 | Alive | 232 | 0.504562 | Low | 1 | 0.46 | FALSE | 0.331956 | 0.213282 | 2 | 2 | 0.0117 |
| TCGA-2J-AAB6 | Pancreas-Adenocarcinoma Ductal Type | Body of Pancreas | MALE | 2 | 75 | >65 | 2 | G2 | 2 | G2 | 5 | 7thStage IIAT3N0M0 | IIA | 3 | II | II | T3 | 3 | T3+T4 | N0 | 1 | Dead | 293 | 9.161623 | High | 2 | 1.51 | FALSE | 0.377379 | 0.774108 | 2 | 1 | ######## |
| TCGA-2J-AABT | Pancreas-Adenocarcinoma Ductal Type | Head of Pancreas | FEMALE | 1 | 72 | >65 | 2 | G2 | 2 | G2 | 4.5 | 7thStage IIBT3N1M0 | IIB | 4 | II | II | T3 | 3 | T3+T4 | N1 | 0 | Alive | 319 | -2.0557 | Low | 1 | 0.29 | FALSE | 0.18097 | 0.290279 | 1 | 1 | 0.025 |
| TCGA-HZ-A77Q | Pancreas-Adenocarcinoma-Other Subtype | Head of Pancreas | FEMALE | 1 | 55 | 65<= | 1 | G2 | 2 | G2 | 2.8 | 7thStage IIBT3N1MX | IIB | 4 | II | II | T3 | 3 | T3+T4 | N1 | 0 | Alive | 33 | 3.220459 | High | 2 | 0.98 | FALSE | 0.143202 | 0.181159 | 1 | 3 | 0.00489 |
| TCGA-IB-7893 | Pancreas-Adenocarcinoma Ductal Type | Body of Pancreas | MALE | 2 | 64 | 65<= | 1 | G3 | 3 | G3+G4 | 7 | 6thStage IIAT3N0M0 | IIA | 3 | II | II | T3 | 3 | T3+T4 | N0 | 1 | Dead | 117 | 6.391836 | High | 2 | 2.48 | FALSE | 0.325233 | 0.224273 | 2 | 3 | 0.00242 |
| TCGA-3E-AAAY | Pancreas-Adenocarcinoma Ductal Type | Other (please specify) | MALE | 2 | 67 | >65 | 2 | G3 | 3 | G3+G4 | 3 | 6thStage IIBT3N1MX | IIB | 4 | II | II | T3 | 3 | T3+T4 | N1 | 0 | Alive | 2172 | -1.45536 | Low | 1 | 0.76 | FALSE | 0.309549 | 0.22542 | 1 | 2 | 0.0142 |
| TCGA-HZ-7922 | Pancreas-Adenocarcinoma Ductal Type | Head of Pancreas | FEMALE | 1 | 61 | 65<= | 1 | G1 | 1 | G1 | 3 | 7thStage IIBT3N1MX | IIB | 4 | II | II | T3 | 3 | T3+T4 | N1 | 0 | Alive | 4 | 4.643229 | High | 2 | 1.44 | FALSE | 0.282341 | 0.416488 | 2 | 3 | 0.0114 |
| TCGA-RB-A7B8 | Pancreas-Adenocarcinoma-Other Subtype | Head of Pancreas | FEMALE | 1 | 81 | >65 | 2 | G2 | 2 | G2 | 2.3 | 7thStage IIBT3N1MX | IIB | 4 | II | II | T3 | 3 | T3+T4 | N1 | 0 | Alive | 36 | 1.135903 | High | 2 | 1.03 | FALSE | 0.223414 | 0.462189 | 2 | 3 | 0.015 |
| TCGA-HZ-7289 | Pancreas-Adenocarcinoma-Other Subtype | Head of Pancreas | MALE | 2 | 77 | >65 | 2 | G1 | 1 | G1 | 4.5 | 7thStage IIBT3N1MX | IIB | 4 | II | II | T3 | 3 | T3+T4 | N1 | 0 | Alive | 240 | -0.67813 | Low | 1 | -0.6 | TRUE | 0.805107 | 0.411932 | 1 | 1 | 4.87E-19 |
| TCGA-YY-A8LH | Pancreas-Adenocarcinoma Ductal Type | Tail of Pancreas | FEMALE | 1 | 61 | 65<= | 1 | G3 | 3 | G3+G4 | 4.2 | 6thStage IIBT3N1MX | IIB | 4 | II | II | T3 | 3 | T3+T4 | N1 | 0 | Alive | 1834 | 3.92626 | High | 2 | -0.92 | TRUE | 0.589031 | 0.236433 | 2 | 2 | 0.011 |
| TCGA-HZ-7923 | Pancreas-Adenocarcinoma Ductal Type | Head of Pancreas | MALE | 2 | 65 | 65<= | 1 | G2 | 2 | G2 | 3 | 7thStage IIAT3N0MX | IIA | 3 | II | II | T3 | 3 | T3+T4 | N0 | 0 | Alive | 8 | -9.24663 | Low | 1 | 0.89 | FALSE | 0.131084 | 0.246578 | 1 | 2 | 0.0807 |
| TCGA-H8-A6C1 | Pancreas-Adenocarcinoma-Other Subtype | Head of Pancreas | MALE | 2 | 53 | 65<= | 1 | G2 | 2 | G2 | 3.1 | 7thStage IIAT3N0MX | IIA | 3 | II | II | T3 | 3 | T3+T4 | N0 | 0 | Alive | 396 | -0.0156 | Low | 1 | -0.5 | TRUE | 0.465865 | 0.669665 | 2 | 2 | 0.00983 |
| TCGA-US-A77G | Pancreas-Adenocarcinoma Ductal Type | Head of Pancreas | MALE | 2 | 64 | 65<= | 1 | G2 | 2 | G2 | 2 | 7thStage IIBT3N1MX | IIB | 4 | II | II | T3 | 3 | T3+T4 | N1 | 1 | Dead | 12 | 0.997131 | Low | 1 | -1.7 | TRUE | 0.597519 | 0.403142 | 2 | 2 | 3.15E-20 |
| TCGA-3A-A9IC | Pancreas-Adenocarcinoma Ductal Type | Head of Pancreas | FEMALE | 1 | 61 | 65<= | 1 | G2 | 2 | G2 | 4 | 7thStage IIBT3N1MX | IIB | 4 | II | II | T3 | 3 | T3+T4 | N1 | 1 | Dead | 738 | 7.032185 | High | 2 | 2.19 | FALSE | 0.24114 | 0.349375 | 2 | 1 | 0.00751 |
| TCGA-IB-A5ST | Pancreas-Adenocarcinoma Ductal Type | Head of Pancreas | FEMALE | 1 | 64 | 65<= | 1 | G2 | 2 | G2 | 3.2 | 7thStage IIBT3N1M0 | IIB | 4 | II | II | T3 | 3 | T3+T4 | N1 | 0 | Alive | 8 | -0.82244 | Low | 1 | 0.99 | FALSE | 0.38009 | 0.374202 | 1 | 2 | 0 |
| TCGA-IB-AAUW | Pancreas-Adenocarcinoma Ductal Type | Head of Pancreas | FEMALE | 1 | 63 | 65<= | 1 | G3 | 3 | G3+G4 | 3.5 | 7thStage IIBT3N1M0 | IIB | 4 | II | II | T3 | 3 | T3+T4 | N1 | 0 | Alive | 179 | -8.0333 | Low | 1 | 0.1 | FALSE | 0.247303 | 0.212837 | 1 | 2 | 0.0284 |
| TCGA-3A-A9I9 | Pancreas-Adenocarcinoma Ductal Type | Head of Pancreas | MALE | 2 | 67 | >65 | 2 | G2 | 2 | G2 | 2.5 | 7thStage IIAT3N0MX | IIA | 3 | II | II | T3 | 3 | T3+T4 | N0 | 1 | Dead | 634 | -0.7395 | Low | 1 | -1.11 | TRUE | 0.320593 | 0.53661 | 2 | 2 | 2.65E-20 |
| TCGA-2J-AABR | Pancreas-Adenocarcinoma Ductal Type | Other (please specify) | FEMALE | 1 | 60 | 65<= | 1 | G3 | 3 | G3+G4 | 3.2 | 7thStage IIAT3N0M0 | IIA | 3 | II | II | T3 | 3 | T3+T4 | N0 | 0 | Alive | 327 | -0.93939 | Low | 1 | 0.68 | FALSE | 0.253225 | 0.246876 | 1 | 3 | 0.0519 |
| TCGA-H6-A45N | Pancreas-Adenocarcinoma-Other Subtype | Tail of Pancreas | FEMALE | 1 | 88 | >65 | 2 | G3 | 3 | G3+G4 | 9 | 7thStage IIBT3N1MX | IIB | 4 | II | II | T3 | 3 | T3+T4 | N1 | 0 | Alive | 233 | -2.94285 | Low | 1 | 1.23 | FALSE | 0.142469 | 0.143787 | 1 | 3 | 0.0189 |
| TCGA-FB-AAQ3 | Pancreas-Adenocarcinoma Ductal Type | Head of Pancreas | FEMALE | 1 | 65 | 65<= | 1 | G2 | 2 | G2 | 2.5 | 7thStage IIBT3N1MX | IIB | 4 | II | II | T3 | 3 | T3+T4 | N1 | 1 | Dead | 31 | 5.501264 | High | 2 | 0.16 | FALSE | 0.465898 | 0.250397 | 2 | 3 | 3.46E-19 |
| TCGA-HV-AA8X | Pancreas-Adenocarcinoma Ductal Type | Body of Pancreas | FEMALE | 1 | 75 | >65 | 2 | G2 | 2 | G2 | NA | 7thStage IIBT2N1MX | IIB | 4 | II | II | T2 | 2 | T1+T2 | N1 | 1 | Dead | 532 | 3.269883 | High | 2 | -0.09 | TRUE | 0.59959 | 0.1727 | 2 | 2 | 4.42E-20 |
| TCGA-IB-8126 | Pancreas-Adenocarcinoma Ductal Type | Head of Pancreas | FEMALE | 1 | 79 | >65 | 2 | G1 | 1 | G1 | 3.5 | 7thStage IIIT4N1M0 | III | 5 | III | III+IV | T4 | 4 | T3+T4 | N1 | 0 | Alive | 17 | -9.12524 | Low | 1 | 0.22 | FALSE | 0.257772 | 0.119611 | 2 | 2 | 0.0703 |
| TCGA-HV-A7OP | Pancreas-Adenocarcinoma-Other Subtype | Head of Pancreas | MALE | 2 | 72 | >65 | 2 | G2 | 2 | G2 | 6.5 | 7thStage IIBT3N1M0 | IIB | 4 | II | II | T3 | 3 | T3+T4 | N1 | 0 | Alive | 859 | -1.34496 | Low | 1 | -0.83 | TRUE | 1 | 0.502992 | 1 | 1 | 2.07E-18 |
| TCGA-F2-6879 | Pancreas-Adenocarcinoma-Other Subtype | Head of Pancreas | MALE | 2 | 57 | 65<= | 1 | G2 | 2 | G2 | 4.5 | 6thStage IIBT3N1M0 | IIB | 4 | II | II | T3 | 3 | T3+T4 | N1 | 1 | Dead | 334 | 5.028941 | High | 2 | 0.48 | FALSE | 0.463127 | 0.269887 | 2 | 2 | 0.0032 |
| TCGA-S4-A8RO | Pancreas-Adenocarcinoma Ductal Type | Head of Pancreas | FEMALE | 1 | 75 | >65 | 2 | G2 | 2 | G2 | 4 | 7thStage IIBT3N1MX | IIB | 4 | II | II | T3 | 3 | T3+T4 | N1 | 0 | Alive | 197 | 5.398965 | High | 2 | 0.05 | FALSE | 0.71743 | 0.280466 | 2 | 3 | 0.00433 |
| TCGA-HZ-A49H | Pancreas-Adenocarcinoma Ductal Type | Head of Pancreas | FEMALE | 1 | 68 | >65 | 2 | G2 | 2 | G2 | 2.8 | 7thStage IIBT3N1MX | IIB | 4 | II | II | T3 | 3 | T3+T4 | N1 | 0 | Alive | 29 | -6.0315 | Low | 1 | 0.42 | FALSE | 0.33928 | 0.241206 | 1 | 2 | 0.0268 |
| TCGA-L1-A7W4 | Pancreas-Adenocarcinoma Ductal Type | Head of Pancreas | FEMALE | 1 | 48 | 65<= | 1 | G3 | 3 | G3+G4 | 2.8 | 7thStage IIBT3N1M0 | IIB | 4 | II | II | T3 | 3 | T3+T4 | N1 | 0 | Alive | 164 | 7.72577 | High | 2 | 1.27 | FALSE | 0.557432 | 0.383424 | 2 | 1 | 0.00607 |
| TCGA-RL-AAAS | Pancreas-Adenocarcinoma Ductal Type | Head of Pancreas | FEMALE | 1 | 60 | 65<= | 1 | G2 | 2 | G2 | 2 | 7thStage IBT2N0M0 | IB | 2 | I | I | T2 | 2 | T1+T2 | N0 | 0 | Alive | 9 | -4.15928 | Low | 1 | 0.83 | FALSE | 0.296206 | 0.242412 | 1 | 2 | 0.0318 |
| TCGA-XN-A8T3 | Pancreas-Adenocarcinoma Ductal Type | Head of Pancreas | MALE | 2 | 67 | >65 | 2 | G2 | 2 | G2 | 2.4 | 7thStage IBT2N0M0 | IB | 2 | I | I | T2 | 2 | T1+T2 | N0 | 0 | Alive | 951 | 1.668279 | High | 2 | 1.6 | FALSE | 0.214189 | 0.334195 | 1 | 1 | 0.0212 |
| TCGA-IB-AAUN | Pancreas-Adenocarcinoma Ductal Type | Head of Pancreas | FEMALE | 1 | 74 | >65 | 2 | G2 | 2 | G2 | 2.3 | 7thStage IBT2N0M0 | IB | 2 | I | I | T2 | 2 | T1+T2 | N0 | 1 | Dead | 144 | 6.395452 | High | 2 | 0.58 | FALSE | 0.386273 | 0.177761 | 1 | 3 | 0.00974 |
| TCGA-HZ-8005 | Pancreas-Adenocarcinoma Ductal Type | Other (please specify) | MALE | 2 | 81 | >65 | 2 | G3 | 3 | G3+G4 | 4.5 | 7thStage IIBT3N1MX | IIB | 4 | II | II | T3 | 3 | T3+T4 | N1 | 1 | Dead | 120 | 10.05701 | High | 2 | 1.35 | FALSE | 0.412444 | 0.37057 | 2 | 1 | 0.0102 |
| TCGA-XD-AAUL | Pancreas-Adenocarcinoma Ductal Type | Head of Pancreas | MALE | 2 | 56 | 65<= | 1 | G2 | 2 | G2 | 2 | 7thStage IIAT3N0MX | IIA | 3 | II | II | T3 | 3 | T3+T4 | N0 | 0 | Alive | 188 | 6.103687 | High | 2 | 0.81 | FALSE | 0.17663 | 0.501375 | 2 | 3 | 9.51E-20 |
| TCGA-IB-AAUP | Pancreas-Adenocarcinoma Ductal Type | Head of Pancreas | MALE | 2 | 68 | >65 | 2 | G2 | 2 | G2 | 2.7 | 7thStage IIBT3N1M0 | IIB | 4 | II | II | T3 | 3 | T3+T4 | N1 | 0 | Alive | 290 | -0.65872 | Low | 1 | 1.54 | FALSE | 0.297524 | 0.328768 | 2 | 2 | 0.0259 |
| TCGA-HV-A5A6 | Pancreas-Adenocarcinoma Ductal Type | Head of Pancreas | FEMALE | 1 | 65 | 65<= | 1 | G1 | 1 | G1 | NA | 7thStage IIBT3N1bM0 | IIB | 4 | II | II | T3 | 3 | T3+T4 | N1 | 0 | Alive | 1953 | 6.392641 | High | 2 | 1.25 | FALSE | 0.397398 | 0.380486 | 2 | 3 | 0.00983 |
| TCGA-HZ-8519 | Pancreas-Adenocarcinoma-Other Subtype | Head of Pancreas | MALE | 2 | 73 | >65 | 2 | G3 | 3 | G3+G4 | 1.8 | 7thStage IAT1N0MX | IA | 1 | I | I | T1 | 1 | T1+T2 | N0 | 0 | Alive | 3 | -14.0187 | Low | 1 | -0.03 | TRUE | 0.295895 | 0.248531 | 1 | 1 | 0.0769 |
| TCGA-IB-7885 | Pancreas-Adenocarcinoma Ductal Type | Head of Pancreas | FEMALE | 1 | 78 | >65 | 2 | G2 | 2 | G2 | 2.4 | 7thStage IIBT3N1M0 | IIB | 4 | II | II | T3 | 3 | T3+T4 | N1 | 0 | Alive | 851 | 5.187938 | High | 2 | 0.37 | FALSE | 0.311703 | 0.259488 | 1 | 2 | 1.80E-20 |
| TCGA-2L-AAQL | Pancreas-Adenocarcinoma Ductal Type | Head of Pancreas | MALE | 2 | 82 | >65 | 2 | G3 | 3 | G3+G4 | 4 | 7thStage IIBT3N1MX | IIB | 4 | II | II | T3 | 3 | T3+T4 | N1 | 1 | Dead | 292 | -1.91123 | Low | 1 | -0.16 | TRUE | 0.38407 | 0.216381 | 2 | 2 | ######## |
| TCGA-IB-AAUT | Pancreas-Colloid (mucinous non-cystic) Carcinoma | Head of Pancreas | MALE | 2 | 65 | 65<= | 1 | G1 | 1 | G1 | NA | 7thStage IIBT2N1M0 | IIB | 4 | II | II | T2 | 2 | T1+T2 | N1 | 0 | Alive | 188 | -7.90231 | Low | 1 | 0.81 | FALSE | 0.161273 | 0.311458 | 1 | 2 | 0.0587 |
| TCGA-IB-7644 | Pancreas-Adenocarcinoma Ductal Type | Head of Pancreas | FEMALE | 1 | 65 | 65<= | 1 | G2 | 2 | G2 | 2.3 | 7thStage IVT3N1M1 | IV | 6 | IV | III+IV | T3 | 3 | T3+T4 | N1 | 0 | Alive | 347 | 1.535176 | High | 2 | 0.35 | FALSE | 0.495605 | 0.508834 | 2 | 2 | 0.0042 |
| TCGA-IB-7889 | Pancreas-Adenocarcinoma Ductal Type | Head of Pancreas | FEMALE | 1 | 85 | >65 | 2 | G1 | 1 | G1 | 2.5 | 7thStage IIBT3N1M0 | IIB | 4 | II | II | T3 | 3 | T3+T4 | N1 | 1 | Dead | 481 | 2.990133 | High | 2 | 0.21 | FALSE | 0.466871 | 0.222664 | 2 | 3 | 0.00214 |
| TCGA-2J-AAB1 | Pancreas-Adenocarcinoma-Other Subtype | Head of Pancreas | MALE | 2 | 65 | 65<= | 1 | G3 | 3 | G3+G4 | 4.5 | 7thStage IIBT3N1M0 | IIB | 4 | II | II | T3 | 3 | T3+T4 | N1 | 1 | Dead | 66 | 3.267564 | High | 2 | -1.07 | TRUE | 0.416064 | 0.415974 | 1 | 3 | 0.018 |
| TCGA-HZ-7919 | Pancreas-Adenocarcinoma Ductal Type | Head of Pancreas | FEMALE | 1 | 52 | 65<= | 1 | G2 | 2 | G2 | 4 | 7thStage IIBT3N1M0 | IIB | 4 | II | II | T3 | 3 | T3+T4 | N1 | 0 | Alive | 20 | 3.220217 | High | 2 | 0.34 | FALSE | 0.400348 | 0.324964 | 2 | 3 | 0.0184 |
