# Supplementary material for: Exploring prognostic and immunological characteristics of pancreatic ductal adenocarcinoma through comprehensive genomic analysis of tertiary lymphoid structures and CD8 + T-cells
Source: J Cancer Res Clin Oncol. 2024 Jun 8;150(6):300. doi: 10.1007/s00432-024-05824-0 (PMC11162401; doi:10.1007/s00432-024-05824-0)
Supplement: Supplementary file 4 — Supplementary Material 4 [file 432_2024_5824_MOESM4_ESM.docx]

**Table S4. Connectivity MAP**

| **Rank** | **Score** | **Type** | **Name** | **Description** | **MOA** |  |
| --- | --- | --- | --- | --- | --- | --- |
| 1 | 97.92 | cp | RO-90-7501 | Beta amyloid inhibitor | Beta amyloid inhibitor | |
| 2 | 96.62 | cp | LY-303511 | Casein kinase inhibitor | Casein kinase inhibitor, MTOR inhibitor, PI3K inhibitor | |
| 3 | 95.98 | cp | TG-101348 | FLT3 inhibitor | FLT3 inhibitor, JAK inhibitor | |
| 4 | 92.74 | cp | scriptaid | HDAC inhibitor | HDAC inhibitor | |
| 5 | 92.72 | cp | ISOX | HDAC inhibitor | HDAC inhibitor | |
| 6 | 92.51 | cp | XMD-892 | MAP kinase inhibitor | MAP kinase inhibitor | |
| 7 | 92.01 | cp | doxorubicin | Topoisomerase inhibitor | Topoisomerase inhibitor | |
| 8 | 91.91 | cp | HC-toxin | HDAC inhibitor | HDAC inhibitor | |
| 9 | 90.21 | cp | XMD-1150 | Leucine rich repeat kinase inhibitor | Leucine rich repeat kinase inhibitor | |
| 10 | 89.45 | cp | loxoprofen | Cyclooxygenase inhibitor | Cyclooxygenase inhibitor, Prostanoid receptor antagonist | |
| 11 | 88.29 | cp | vorinostat | HDAC inhibitor | HDAC inhibitor | |
| 12 | 87.95 | cp | afatinib | EGFR inhibitor | EGFR inhibitor | |
| 13 | 87.75 | cp | panobinostat | HDAC inhibitor | HDAC inhibitor | |
| 14 | 87.11 | cp | baeomycesic-acid | Lipoxygenase inhibitor | Lipoxygenase inhibitor | |
| 15 | 86.53 | cp | SB-221284 | Serotonin receptor antagonist | Serotonin receptor antagonist | |
| 16 | 86.07 | cp | apicidin | HDAC inhibitor | HDAC inhibitor | |
| 17 | 85.88 | cp | trichostatin-a | HDAC inhibitor | HDAC inhibitor, CDK activator, ID1 inhibitor | |
| 18 | 85.44 | cp | leucodin | Melanin inhibitor | Melanin inhibitor | |
| 19 | 85.03 | cp | THM-I-94 | HDAC inhibitor | HDAC inhibitor | |
| 20 | 79.39 | cp | verrucarin-a | Protein synthesis inhibitor | Protein synthesis inhibitor | |
| 21 | 79.24 | cp | cephaeline | Protein synthesis inhibitor | Protein synthesis inhibitor | |
| 22 | 78.17 | cp | idarubicin | Topoisomerase inhibitor | Topoisomerase inhibitor | |
| 23 | 77.36 | cp | tyrphostin-AG-1478 | EGFR inhibitor | EGFR inhibitor | |
| 24 | 76.79 | cp | CGP-60474 | CDK inhibitor | CDK inhibitor | |
| 25 | 76.78 | cp | solifenacin | Acetylcholine receptor antagonist | Acetylcholine receptor antagonist | |
| 26 | 76.41 | cp | tivozanib | VEGFR inhibitor | VEGFR inhibitor | |
| 27 | 76.05 | cp | SB-202190 | p38 MAPK inhibitor | p38 MAPK inhibitor | |
| 28 | 75.12 | cp | dacinostat | HDAC inhibitor | HDAC inhibitor | |
| 29 | 72.27 | cp | ZG-10 | JNK inhibitor | JNK inhibitor | |
| 30 | 72.02 | cp | palonosetron | Serotonin receptor antagonist | Serotonin receptor antagonist | |
| 31 | 71.46 | cp | NCH-51 | HDAC inhibitor | HDAC inhibitor | |
| 32 | 69.11 | cp | sorafenib | FLT3 inhibitor | FLT3 inhibitor, KIT inhibitor, PDGFR receptor inhibitor, RAF inhibitor, VEGFR inhibitor, RET tyrosine kinase inhibitor | |
| 33 | 66.52 | cp | NU-7441 | DNA dependent protein kinase inhibitor | DNA dependent protein kinase inhibitor, P-glycoprotein inhibitor | |
| 34 | 66.52 | cp | sarmentogenin | ATPase inhibitor | ATPase inhibitor | |
| 35 | 65.68 | cp | belinostat | HDAC inhibitor | HDAC inhibitor | |
| 36 | 63.53 | cp | 6-aminochrysene | Transferase inhibitor | Transferase inhibitor | |
| 37 | 63.04 | cp | brefeldin-a | Protein synthesis inhibitor | Protein synthesis inhibitor, Brefeldin A inhibited guanine nucleotide exchange protein inhibitor, Golgi-specific brefeldin A-resistance guanine nucleotide exchange factor inhibitor | |
| 38 | 61.35 | cp | W-12 | Calmodulin antagonist | Calmodulin antagonist | |
| 39 | 61 | cp | staurosporine | PKC inhibitor | PKC inhibitor | |
| 40 | 60.54 | cp | lypressin | Vasopressin receptor agonist | Vasopressin receptor agonist | |
| 41 | 60.49 | cp | dobutamine | Adrenergic receptor agonist | Adrenergic receptor agonist | |
| 42 | 60.45 | cp | emetine | Protein synthesis inhibitor | Protein synthesis inhibitor | |
| 43 | 60.12 | cp | TWS-119 | Glycogen synthase kinase inhibitor | Glycogen synthase kinase inhibitor | |
| 44 | 59.61 | cp | homoharringtonine | Protein synthesis inhibitor | Protein synthesis inhibitor | |
| 45 | 59.19 | cp | scopoline | Acetylcholine receptor antagonist | Acetylcholine receptor antagonist | |
| 46 | 58.5 | cp | RS-67506 | Serotonin receptor partial agonist | Serotonin receptor partial agonist | |
| 47 | 57.43 | cp | pyroxamide | HDAC inhibitor | HDAC inhibitor | |
| 48 | 56.73 | cp | sulfinpyrazone | Uricosuric blocker | Uricosuric blocker | |
| 49 | 56.25 | cp | BI-2536 | PLK inhibitor | PLK inhibitor | |
| 50 | 56.04 | cp | JAK3-inhibitor-I | JAK inhibitor | JAK inhibitor | |
| 51 | 55.32 | cp | tetramethylsilane | Internal standard for NMR spectroscopy | Internal standard for NMR spectroscopy | |
| 52 | 54.96 | cp | dactinomycin | RNA polymerase inhibitor | RNA polymerase inhibitor | |
| 53 | 54.88 | cp | W-7 | Calmodulin antagonist | Calmodulin antagonist, Potassium channel blocker | |
| 54 | 53.45 | cp | GSK-3-inhibitor-II | PKC inhibitor | PKC inhibitor | |
| 55 | 52.65 | cp | erythrosine | Coloring agent | Coloring agent | |
| 56 | 52.62 | cp | NNC-63-0532 | Opioid receptor agonist | Opioid receptor agonist | |
| 57 | 52.48 | cp | dihydro-7-desacetyldeoxygedunin | HSP inhibitor | HSP inhibitor | |
| 58 | 52.46 | cp | W-5 | Calmodulin antagonist | Calmodulin antagonist | |
| 59 | 52.14 | cp | prometon | Photosynthesis inhibitor | Photosynthesis inhibitor | |
| 60 | 51.08 | cp | NVP-AUY922 | HSP inhibitor | HSP inhibitor | |
| 61 | 50.52 | cp | heraclenol | Vitamin K antagonist | Vitamin K antagonist | |
| 62 | 50.49 | cp | dichloroacetic-acid | Pyruvate dehydrogenase kinase inhibitor | Pyruvate dehydrogenase kinase inhibitor | |
| 63 | 47.25 | cp | ST-638 | Tyrosine kinase inhibitor | Tyrosine kinase inhibitor | |
| 64 | 46.91 | cp | NSC-23766 | Ras GTPase inhibitor | Ras GTPase inhibitor | |
| 65 | 46.23 | cp | GSK-1059615 | PI3K inhibitor | PI3K inhibitor | |
| 66 | 46.02 | cp | tianeptine | Selective serotonin reuptake enhancer (SSRE) | Selective serotonin reuptake enhancer (SSRE) | |
| 67 | 45.79 | cp | mocimycin | Protein synthesis inhibitor | Protein synthesis inhibitor | |
| 68 | 45.05 | cp | tipifarnib | Farnesyltransferase inhibitor | Farnesyltransferase inhibitor | |
| 69 | 43.51 | cp | pidorubicine | Topoisomerase inhibitor | Topoisomerase inhibitor | |
| 70 | 41.36 | cp | PIK-90 | PI3K inhibitor | PI3K inhibitor | |
| 71 | 40.62 | cp | aminopurvalanol-a | Tyrosine kinase inhibitor | Tyrosine kinase inhibitor, CDK inhibitor | |
| 72 | 40.42 | cp | 17-hydroxyprogesterone-caproate | progesterone receptor agonist | progesterone receptor agonist | |
| 73 | 40.4 | cp | nitrazepam | Benzodiazepine receptor agonist | Benzodiazepine receptor agonist | |
| 74 | 39.08 | cp | diphemanil | Acetylcholine receptor antagonist | Acetylcholine receptor antagonist | |
| 75 | 38.92 | cp | HG-6-64-01 | RAF inhibitor | RAF inhibitor | |
| 76 | 38.21 | cp | byssochlamic-acid | Mycotoxin | Mycotoxin | |
| 77 | 37.8 | cp | TPCA-1 | IKK inhibitor | IKK inhibitor | |
| 78 | 37.43 | cp | eicosadienoic-acid | -666 | -666 |  |
| 79 | 37.21 | cp | ciglitazone | PPAR receptor agonist | PPAR receptor agonist | |
| 80 | 36.64 | cp | CS-110266 | Dopamine receptor agonist | Dopamine receptor agonist | |
| 81 | 36.49 | cp | PU-H71 | HSP inhibitor | HSP inhibitor | |
| 82 | 36.11 | cp | scandenin | Plant compound with antimicrobial activity | Plant compound with antimicrobial activity | |
| 83 | 35.35 | cp | yohimbine | Adrenergic receptor antagonist | Adrenergic receptor antagonist | |
| 84 | 35.29 | cp | liquiritigenin | Aromatase inhibitor | Aromatase inhibitor | |
| 85 | 34 | cp | taurodeoxycholic-acid | Bile acid | Bile acid | |
| 86 | 33.91 | cp | 1-monopalmitin | P-glycoprotein inhibitor | P-glycoprotein inhibitor | |
| 87 | 33.6 | cp | bifonazole | Sterol demethylase inhibitor | Sterol demethylase inhibitor | |
| 88 | 33.6 | cp | flutamide | Androgen receptor antagonist | Androgen receptor antagonist | |
| 89 | 33.31 | cp | gatifloxacin | Bacterial DNA gyrase inhibitor | Bacterial DNA gyrase inhibitor | |
| 90 | 33.06 | cp | salbutamol | Adrenergic receptor agonist | Adrenergic receptor agonist | |
| 91 | 32.88 | cp | etilefrine | Adrenergic receptor agonist | Adrenergic receptor agonist | |
| 92 | 32.79 | cp | UBP-302 | Glutamate receptor antagonist | Glutamate receptor antagonist | |
| 93 | 31.77 | cp | EBPC | Aldose reductase inhibitor | Aldose reductase inhibitor | |
| 94 | 31.72 | cp | fasudil | Rho associated kinase inhibitor | Rho associated kinase inhibitor | |
| 95 | 31.3 | cp | PI-828 | PI3K inhibitor | PI3K inhibitor | |
| 96 | 30.89 | cp | tyrphostin-46 | Tyrosine kinase inhibitor | Tyrosine kinase inhibitor | |
| 97 | 30.76 | cp | AG-879 | Angiogenesis inhibitor | Angiogenesis inhibitor, Tyrosine kinase inhibitor, VEGFR inhibitor | |
| 98 | 30.33 | cp | LY-364947 | TGF beta receptor inhibitor | TGF beta receptor inhibitor, p38 MAPK inhibitor | |
| 99 | 30.18 | cp | BRL-52537 | Opioid receptor agonist | Opioid receptor agonist | |
| 100 | 29.89 | cp | tyrphostin-AG-1296 | FLT3 inhibitor | FLT3 inhibitor | |
| 101 | 29.36 | cp | butabindide | Tripeptidyl peptidase inhibitor | Tripeptidyl peptidase inhibitor | |
| 102 | 29.22 | cp | T-0156 | Phosphodiesterase inhibitor | Phosphodiesterase inhibitor | |
| 103 | 29.17 | cp | prunetin | Breast cancer resistance protein inhibitor | Breast cancer resistance protein inhibitor | |
| 104 | 28.9 | cp | BRD-K34437622 | Thymidylate synthase inhibitor | Thymidylate synthase inhibitor | |
| 105 | 28.64 | cp | nicotinamide | Protein synthesis stimulant | Protein synthesis stimulant | |
| 106 | 28.44 | cp | dihydroxyphenylglycine | Glutamate receptor agonist | Glutamate receptor agonist | |
| 107 | 28.37 | cp | quercetagetin | PIM inhibitor | PIM inhibitor | |
| 108 | 28.36 | cp | geranylgeraniol | Farnesyltransferase inhibitor | Farnesyltransferase inhibitor | |
| 109 | 28.24 | cp | ochratoxin-a | Phenylalanyl tRNA synthetase inhibitor | Phenylalanyl tRNA synthetase inhibitor | |
| 110 | 27.95 | cp | BD-1063 | Sigma receptor antagonist | Sigma receptor antagonist | |
| 111 | 27.73 | cp | GSK-0660 | PPAR receptor antagonist | PPAR receptor antagonist | |
| 112 | 27.23 | cp | kawain | Calcium channel modulator | Calcium channel modulator, MTOR inhibitor, Sodium channel blocker | |
| 113 | 27.12 | cp | bergenin | Interleukin inhibitor | Interleukin inhibitor | |
| 114 | 27 | cp | alvocidib | CDK inhibitor | CDK inhibitor | |
| 115 | 26.98 | cp | PD-158780 | EGFR inhibitor | EGFR inhibitor | |
| 116 | 26.74 | cp | WR-216174 | PFMRK inhibitor | PFMRK inhibitor | |
| 117 | 26.69 | cp | propranolol | Adrenergic receptor antagonist | Adrenergic receptor antagonist | |
| 118 | 26.61 | cp | farnesylthiotriazole | PPMTase inhibitor | PPMTase inhibitor | |
| 119 | 26.48 | cp | pramipexole | Dopamine receptor agonist | Dopamine receptor agonist | |
| 120 | 26.22 | cp | MDL-29951 | Glutamate receptor antagonist | Glutamate receptor antagonist | |
| 121 | 26.02 | cp | givinostat | HDAC inhibitor | HDAC inhibitor | |
| 122 | 25.94 | cp | RO-25-6981 | Ionotropic glutamate receptor antagonist | Ionotropic glutamate receptor antagonist, Monamine transporter modulator | |
| 123 | 25.56 | cp | 7,4'-dihydroxyflavone | Opioid receptor antagonist | Opioid receptor antagonist | |
| 124 | 25.42 | cp | acadesine | AMPK activator | AMPK activator | |
| 125 | 25.07 | cp | ZK-93423 | Benzodiazepine receptor agonist | Benzodiazepine receptor agonist | |
| 126 | 24.66 | cp | busulfan | DNA inhibitor | DNA inhibitor | |
| 127 | 23.98 | cp | SC-68376 | p38 MAPK inhibitor | p38 MAPK inhibitor | |
| 128 | 23.53 | cp | artesunate | DNA synthesis inhibitor | DNA synthesis inhibitor | |
| 129 | 23.05 | cp | arachidonamide | Cannabinoid receptor agonist | Cannabinoid receptor agonist | |
| 130 | 22.92 | cp | deoxycholic-acid | G protein-coupled receptor agonist | G protein-coupled receptor agonist, Biliverdin reductase A activator, Unidentified pharmacological activity | |
| 131 | 22.41 | cp | KU-0060648 | DNA dependent protein kinase inhibitor | DNA dependent protein kinase inhibitor, PI3K inhibitor | |
| 132 | 22.25 | cp | alfaxalone | Chloride channel agonist | Chloride channel agonist, GABA receptor agonist | |
| 133 | 22.19 | cp | TC-2559 | Acetylcholine receptor agonist | Acetylcholine receptor agonist | |
| 134 | 22.1 | cp | fludroxycortide | Glucocorticoid receptor agonist | Glucocorticoid receptor agonist | |
| 135 | 21.53 | cp | alfadolone | GABA receptor agonist | GABA receptor agonist | |
| 136 | 21.4 | cp | antimycin-a | ATP synthase inhibitor | ATP synthase inhibitor | |
| 137 | 21.3 | cp | mifobate | PPAR receptor antagonist | PPAR receptor antagonist | |
| 138 | 21.28 | cp | GBR-13069 | Dopamine uptake inhibitor | Dopamine uptake inhibitor | |
| 139 | 20.84 | cp | tetrindole | Monoamine oxidase inhibitor | Monoamine oxidase inhibitor | |
| 140 | 20.69 | cp | moxifloxacin | Bacterial DNA gyrase inhibitor | Bacterial DNA gyrase inhibitor | |
| 141 | 20.61 | cp | propoxycaine | Local anesthetic | Local anesthetic | |
| 142 | 20.44 | cp | carbenoxolone | 11-beta-HSD1 inhibitor | 11-beta-HSD1 inhibitor | |
| 143 | 20.44 | cp | phentermine | Dopamine uptake inhibitor | Dopamine uptake inhibitor, Serotonin reuptake inhibitor | |
| 144 | 20.43 | cp | PD-123319 | Angiotensin receptor antagonist | Angiotensin receptor antagonist | |
| 145 | 20.33 | cp | ellipticine | Topoisomerase inhibitor | Topoisomerase inhibitor | |
| 146 | 20.09 | cp | CGP-57380 | MAP kinase inhibitor | MAP kinase inhibitor | |
| 147 | 19.99 | cp | gamma-linolenic-acid | Cyclooxygenase inhibitor | Cyclooxygenase inhibitor, Prostanoid receptor agonist | |
| 148 | 19.98 | cp | WZ-4-145 | EGFR inhibitor | EGFR inhibitor | |
| 149 | 19.74 | cp | goserelin | Gonadotropin releasing factor hormone receptor agonist | Gonadotropin releasing factor hormone receptor agonist, Luteinizing hormone releasing hormone agonist | |
| 150 | 19.73 | cp | norethisterone | Progesterone receptor agonist | Progesterone receptor agonist | |
| 151 | 19.56 | cp | canertinib | EGFR inhibitor | EGFR inhibitor | |
| 152 | 19.5 | cp | midostaurin | FLT3 inhibitor | FLT3 inhibitor, KIT inhibitor, PKC inhibitor | |
| 153 | 19.27 | cp | 3-matida | Glutamate receptor antagonist | Glutamate receptor antagonist | |
| 154 | 19.19 | cp | vidarabine | Antiviral | Antiviral | |
| 155 | 19.18 | cp | naltrexone | Opioid receptor antagonist | Opioid receptor antagonist | |
| 156 | 19.06 | cp | selegiline | Monoamine oxidase inhibitor | Monoamine oxidase inhibitor | |
| 157 | 18.75 | cp | SKF-86002 | p38 MAPK inhibitor | p38 MAPK inhibitor | |
| 158 | 18.73 | cp | ketoprofen | Cyclooxygenase inhibitor | Cyclooxygenase inhibitor | |
| 159 | 18.49 | cp | GR-135531 | Melatonin receptor agonist | Melatonin receptor agonist | |
| 160 | 18.37 | cp | pinocembrin | CYP1B1 inhibitor | CYP1B1 inhibitor | |
| 161 | 18.32 | cp | III606050 | Cytochrome P450 inhibitor | Cytochrome P450 inhibitor | |
| 162 | 18.31 | cp | thiazolidinecarboxylic-acid | Reducing agent | Reducing agent | |
| 163 | 18.11 | cp | biochanin-a | Estrogen receptor agonist | Estrogen receptor agonist | |
| 164 | 18.07 | cp | bis-tyrphostin | EGFR inhibitor | EGFR inhibitor | |
| 165 | 17.91 | cp | digitoxin | ATPase inhibitor | ATPase inhibitor | |
| 166 | 17.56 | cp | leflunomide | Dihydroorotate dehydrogenase inhibitor | Dihydroorotate dehydrogenase inhibitor, PDGFR receptor inhibitor | |
| 167 | 17.38 | cp | quipazine | Serotonin receptor agonist | Serotonin receptor agonist | |
| 168 | 17.37 | cp | phloretin | Sodium/glucose cotransporter inhibitor | Sodium/glucose cotransporter inhibitor | |
| 169 | 17.31 | cp | QL-X-138 | MTOR inhibitor | MTOR inhibitor | |
| 170 | 17.28 | cp | droxinostat | HDAC inhibitor | HDAC inhibitor | |
| 171 | 17.03 | cp | floxuridine | DNA synthesis inhibitor | DNA synthesis inhibitor | |
| 172 | 16.93 | cp | MBCQ | Phosphodiesterase inhibitor | Phosphodiesterase inhibitor | |
| 173 | 16.86 | cp | lasalocid | Bacterial permeability inducer | Bacterial permeability inducer | |
| 174 | 16.81 | cp | U-46619 | Thromboxane receptor agonist | Thromboxane receptor agonist | |
| 175 | 16.71 | cp | flavanone | 11-beta-HSD1 inhibitor | 11-beta-HSD1 inhibitor | |
| 176 | 16.68 | cp | LE-300 | Dopamine receptor antagonist | Dopamine receptor antagonist | |
| 177 | 16.19 | cp | trimethobenzamide | Histamine receptor antagonist | Histamine receptor antagonist | |
| 178 | 15.96 | cp | loratadine | Histamine receptor antagonist | Histamine receptor antagonist | |
| 179 | 15.89 | cp | ampiroxicam | Cyclooxygenase inhibitor | Cyclooxygenase inhibitor | |
| 180 | 15.8 | cp | chromomycin-a3 | DNA binding agent | DNA binding agent | |
| 181 | 15.48 | cp | tubastatin-a | HDAC inhibitor | HDAC inhibitor | |
| 182 | 15.47 | cp | sulmazole | Adenosine receptor antagonist | Adenosine receptor antagonist | |
| 183 | 15.38 | cp | dipropyl-5ct | Serotonin receptor agonist | Serotonin receptor agonist | |
| 184 | 15.21 | cp | ZD-7114 | Adrenergic receptor agonist | Adrenergic receptor agonist | |
| 185 | 15.17 | cp | enobosarm | Androgen receptor modulator | Androgen receptor modulator | |
| 186 | 15.16 | cp | indirubin | CDK inhibitor | CDK inhibitor, Glycogen synthase kinase inhibitor, Leucine rich repeat kinase inhibitor, SRC inhibitor, Serum/glucocorticoid regulated kinase inhibitor | |
| 187 | 14.89 | cp | minoxidil | KATP activator | KATP activator, KIR6 channel (KATP) activator, Vasodilator | |
| 188 | 14.71 | cp | JLK-6 | Gamma secretase inhibitor | Gamma secretase inhibitor, Anti-amyloidogenic | |
| 189 | 14.69 | cp | AR-A014418 | Glycogen synthase kinase inhibitor | Glycogen synthase kinase inhibitor | |
| 190 | 14.69 | cp | SD-169 | p38 MAPK inhibitor | p38 MAPK inhibitor | |
| 191 | 14.55 | cp | GR-113808 | Serotonin receptor antagonist | Serotonin receptor antagonist | |
| 192 | 14.46 | cp | JW-7-24-1 | LCK Inhibitor | LCK Inhibitor | |
| 193 | 14.14 | cp | Y-134 | Estrogen receptor antagonist | Estrogen receptor antagonist | |
| 194 | 13.93 | cp | altizide | Thiazide diuretic | Thiazide diuretic | |
| 195 | 13.92 | cp | dantron | Laxative | Laxative |  |
| 196 | 13.81 | cp | piperine | Monoamine oxidase inhibitor | Monoamine oxidase inhibitor | |
| 197 | 13.63 | cp | H-7 | PKA inhibitor | PKA inhibitor | |
| 198 | 13.3 | cp | calyculin | Protein phosphatase inhibitor | Protein phosphatase inhibitor | |
| 199 | 13.08 | cp | chromanol | Potassium channel blocker | Potassium channel blocker | |
| 200 | 13.07 | cp | naltrindole | Opioid receptor antagonist | Opioid receptor antagonist | |
| 201 | 13.02 | cp | ZM-306416 | ABL inhibitor | ABL inhibitor, SRC inhibitor, VEGFR inhibitor | |
| 202 | 12.96 | cp | monastrol | Kinesin-like spindle protein inhibitor | Kinesin-like spindle protein inhibitor | |
| 203 | 12.63 | cp | procyclidine | Acetylcholine receptor antagonist | Acetylcholine receptor antagonist | |
| 204 | 12.45 | cp | nilutamide | Androgen receptor antagonist | Androgen receptor antagonist | |
| 205 | 12.27 | cp | procaterol | Adrenergic receptor agonist | Adrenergic receptor agonist | |
| 206 | 12.13 | cp | tiabendazole | Angiogenesis inhibitor | Angiogenesis inhibitor | |
| 207 | 11.99 | cp | ceforanide | Penicillin binding protein inhibitor | Penicillin binding protein inhibitor | |
| 208 | 11.96 | cp | 2-(4-methoxybenzylthio)-6-methylpyrimidin-4-ol | Matrix metalloprotease inhibitor | Matrix metalloprotease inhibitor | |
| 209 | 11.95 | cp | AT-7519 | CDK inhibitor | CDK inhibitor, Cell cycle inhibitor | |
| 210 | 11.92 | cp | norketamine | Glutamate receptor antagonist | Glutamate receptor antagonist | |
| 211 | 11.86 | cp | PP-1 | SRC inhibitor | SRC inhibitor, ABL inhibitor | |
| 212 | 11.74 | cp | KU-0063794 | MTOR inhibitor | MTOR inhibitor | |
| 213 | 11.62 | cp | ZSTK-474 | PI3K inhibitor | PI3K inhibitor | |
| 214 | 11.53 | cp | veliparib | PARP inhibitor | PARP inhibitor | |
| 215 | 11.47 | cp | BVT-948 | Tyrosine phosphatase inhibitor | Tyrosine phosphatase inhibitor | |
| 216 | 11.46 | cp | latrunculin-b | Actin polymerization inhibitor | Actin polymerization inhibitor, Unidentified pharmacological activity | |
| 217 | 11.38 | cp | rifampicin | RNA polymerase inhibitor | RNA polymerase inhibitor | |
| 218 | 10.99 | cp | JNK-9L | JNK inhibitor | JNK inhibitor | |
| 219 | 10.85 | cp | pirarubicin | Topoisomerase inhibitor | Topoisomerase inhibitor | |
| 220 | 10.68 | cp | esmolol | Adrenergic receptor antagonist | Adrenergic receptor antagonist | |
| 221 | 10.65 | cp | BIBX-1382 | EGFR inhibitor | EGFR inhibitor, Tyrosine kinase inhibitor | |
| 222 | 10.61 | cp | L-690488 | Inositol monophosphatase inhibitor | Inositol monophosphatase inhibitor | |
| 223 | 10.49 | cp | U-74389F | Lipid peroxidase inhibitor | Lipid peroxidase inhibitor | |
| 224 | 10.37 | cp | digitoxigenin | ATPase inhibitor | ATPase inhibitor | |
| 225 | 10.29 | cp | etazolate | Phosphodiesterase inhibitor | Phosphodiesterase inhibitor | |
| 226 | 10.03 | cp | GW-5074 | Leucine rich repeat kinase inhibitor | Leucine rich repeat kinase inhibitor, RAF inhibitor | |
| 227 | 9.99 | cp | ouabain | ATPase inhibitor | ATPase inhibitor | |
| 228 | 9.9 | cp | PLX-4720 | RAF inhibitor | RAF inhibitor | |
| 229 | 9.86 | cp | lestaurtinib | FLT3 inhibitor | FLT3 inhibitor, Growth factor receptor inhibitor, JAK inhibitor | |
| 230 | 9.83 | cp | SA-94315 | Caspase inhibitor | Caspase inhibitor | |
| 231 | 9.8 | cp | nitazoxanide | Pyruvate ferredoxin oxidoreductase inhibitor | Pyruvate ferredoxin oxidoreductase inhibitor | |
| 232 | 9.77 | cp | chlorphensin | Muscle relaxant | Muscle relaxant | |
| 233 | 9.74 | cp | 5-iodotubercidin | Adenosine kinase inhibitor | Adenosine kinase inhibitor | |
| 234 | 9.73 | cp | gelsemine | Acetylcholine receptor antagonist | Acetylcholine receptor antagonist, Glycine receptor antagonist | |
| 235 | 9.55 | cp | pentylenetetrazol | GABA receptor antagonist | GABA receptor antagonist | |
| 236 | 9.44 | cp | IBC-293 | Hydroxycarboxylic acid receptor agonist | Hydroxycarboxylic acid receptor agonist | |
| 237 | 9.42 | cp | alprenolol | Adrenergic receptor antagonist | Adrenergic receptor antagonist | |
| 238 | 9.32 | cp | GR-144053 | Integrin antagonist | Integrin antagonist | |
| 239 | 9.14 | cp | forskolin | Adenylyl cyclase activator | Adenylyl cyclase activator | |
| 240 | 9.05 | cp | H-8 | PKA inhibitor | PKA inhibitor | |
| 241 | 8.82 | cp | piperacillin | Bacterial cell wall synthesis inhibitor | Bacterial cell wall synthesis inhibitor | |
| 242 | 8.59 | cp | efavirenz | HIV protease inhibitor | HIV protease inhibitor | |
| 243 | 8.54 | cp | praziquantel | Anthelmintic | Anthelmintic | |
| 244 | 8.35 | cp | PP-30 | RAF inhibitor | RAF inhibitor | |
| 245 | 8.29 | cp | denbufylline | Phosphodiesterase inhibitor | Phosphodiesterase inhibitor | |
| 246 | 8.28 | cp | bisindolylmaleimide-ix | CDK inhibitor | CDK inhibitor, PKC inhibitor | |
| 247 | 8.27 | cp | methyl-2,5-dihydroxycinnamate | EGFR inhibitor | EGFR inhibitor, Tyrosine kinase inhibitor | |
| 248 | 8.24 | cp | Ala-Ala-Phe-CMK | Tripeptidyl peptidase inhibitor | Tripeptidyl peptidase inhibitor | |
| 249 | 8.16 | cp | puromycin | Protein synthesis inhibitor | Protein synthesis inhibitor | |
| 250 | 8.14 | cp | RS-45041-190 | Imidazoline receptor agonist | Imidazoline receptor agonist | |
| 251 | 8.14 | cp | bemesetron | Serotonin receptor antagonist | Serotonin receptor antagonist | |
| 252 | 8.11 | cp | T-0070907 | PPAR receptor antagonist | PPAR receptor antagonist | |
| 253 | 8.08 | cp | DAU-5884 | Acetylcholine receptor antagonist | Acetylcholine receptor antagonist | |
| 254 | 8.08 | cp | AZD-7762 | CHK inhibitor | CHK inhibitor | |
| 255 | 8.06 | cp | MR-16728 | Acetylcholine release enhancer | Acetylcholine release enhancer, Acetylcholine release stimulant | |
| 256 | 8 | cp | EI-247 | IGF-1 inhibitor | IGF-1 inhibitor | |
| 257 | 7.87 | cp | HSP90-inhibitor | HSP inhibitor | HSP inhibitor | |
| 258 | 7.87 | cp | cyanopindolol | Adrenergic receptor antagonist | Adrenergic receptor antagonist | |
| 259 | 7.7 | cp | fludrocortisone | Glucocorticoid receptor agonist | Glucocorticoid receptor agonist, Mineralocorticoid receptor agonist | |
| 260 | 7.68 | cp | teniposide | Topoisomerase inhibitor | Topoisomerase inhibitor | |
| 261 | 7.58 | cp | AMN-082 | Glutamate receptor modulator | Glutamate receptor modulator, Glutamate receptor positive allosteric modulator | |
| 262 | 7.53 | cp | estradiol | Contraceptive agent | Contraceptive agent, Estrogen receptor agonist | |
| 263 | 7.49 | cp | tyrphostin-AG-527 | Protein tyrosine kinase inhibitor | Protein tyrosine kinase inhibitor | |
| 264 | 7.45 | cp | bithionol | Autotaxin inhibitor | Autotaxin inhibitor | |
| 265 | 7.36 | cp | L-BSO | Glutathione transferase inhibitor | Glutathione transferase inhibitor | |
| 266 | 7.33 | cp | norethindrone | Progesterone receptor agonist | Progesterone receptor agonist, Progestogen hormone | |
| 267 | 7.31 | cp | deltaline | Acetylcholine receptor antagonist | Acetylcholine receptor antagonist | |
| 268 | 7.28 | cp | PNU-96415E | Dopamine receptor antagonist | Dopamine receptor antagonist | |
| 269 | 7.14 | cp | demeclocycline | Bacterial 30S ribosomal subunit inhibitor | Bacterial 30S ribosomal subunit inhibitor | |
| 270 | 7.07 | cp | dibenzoylmethane | Antineoplastic | Antineoplastic | |
| 271 | 7.04 | cp | cefotaxime | Bacterial cell wall synthesis inhibitor | Bacterial cell wall synthesis inhibitor | |
| 272 | 7.04 | cp | velnacrine | cholinesterase inhibitor | cholinesterase inhibitor | |
| 273 | 6.89 | cp | orantinib | FGFR inhibitor | FGFR inhibitor, VEGFR inhibitor, PDGFR receptor inhibitor | |
| 274 | 6.89 | cp | TUL-XXI039 | Serine/threonine kinase inhibitor | Serine/threonine kinase inhibitor | |
| 275 | 6.8 | cp | zacopride | Serotonin receptor antagonist | Serotonin receptor antagonist | |
| 276 | 6.8 | cp | mitoxantrone | Topoisomerase inhibitor | Topoisomerase inhibitor | |
| 277 | 6.79 | cp | tyrphostin-AG-555 | EGFR inhibitor | EGFR inhibitor | |
| 278 | 6.6 | cp | etamivan | Respiratory stimulant | Respiratory stimulant | |
| 279 | 6.44 | cp | zosuquidar | P-glycoprotein inhibitor | P-glycoprotein inhibitor | |
| 280 | 6.42 | cp | erythromycin | NFkB pathway inhibitor | NFkB pathway inhibitor | |
| 281 | 6.41 | cp | niflumic-acid | Cyclooxygenase inhibitor | Cyclooxygenase inhibitor | |
| 282 | 6.27 | cp | dihydrodeoxygedunin | Growth factor receptor activator | Growth factor receptor activator | |
| 283 | 6.22 | cp | SIB-1757 | Glutamate receptor antagonist | Glutamate receptor antagonist | |
| 284 | 6.21 | cp | entinostat | HDAC inhibitor | HDAC inhibitor | |
| 285 | 6.13 | cp | pirlindole | Monoamine oxidase inhibitor | Monoamine oxidase inhibitor | |
| 286 | 6.12 | cp | nikkomycin | Chitin inhibitor | Chitin inhibitor | |
| 287 | 5.95 | cp | BIIB021 | HSP inhibitor | HSP inhibitor | |
| 288 | 5.9 | cp | HA-14-1 | BCL inhibitor | BCL inhibitor | |
| 289 | 5.81 | cp | tenofovir | Reverse transcriptase inhibitor | Reverse transcriptase inhibitor, Cytochrome P450 inhibitor, DNA polymerase inhibitor, HIV integrase inhibitor | |
| 290 | 5.81 | cp | JAK3-Inhibitor-II | JAK inhibitor | JAK inhibitor | |
| 291 | 5.77 | cp | cefazolin | Bacterial cell wall synthesis inhibitor | Bacterial cell wall synthesis inhibitor | |
| 292 | 5.71 | cp | ziprasidone | Dopamine receptor antagonist | Dopamine receptor antagonist, Serotonin receptor antagonist | |
| 293 | 5.67 | cp | dasatinib | BCR-ABL kinase inhibitor | BCR-ABL kinase inhibitor, Ephrin inhibitor, KIT inhibitor, PDGFR receptor inhibitor, SRC inhibitor, Tyrosine kinase inhibitor | |
| 294 | 5.45 | cp | dexchlorpheniramine | Histamine receptor antagonist | Histamine receptor antagonist | |
| 295 | 5.36 | cp | CP466722 | ATM kinase inhibitor | ATM kinase inhibitor | |
| 296 | 5.34 | cp | BU-224 | Imidazoline receptor ligand | Imidazoline receptor ligand | |
| 297 | 5.29 | cp | troxipide | Glucosamine synthetase stimulant | Glucosamine synthetase stimulant | |
| 298 | 5.29 | cp | CGS-21680 | Adenosine receptor agonist | Adenosine receptor agonist | |
| 299 | 5.25 | cp | radicicol | HSP inhibitor | HSP inhibitor | |
| 300 | 5.25 | cp | westcort | Glucocorticoid receptor agonist | Glucocorticoid receptor agonist | |
| 301 | 5.22 | cp | taxifolin | Opioid receptor antagonist | Opioid receptor antagonist | |
| 302 | 5.22 | cp | pidotimod | Interferon receptor agonist | Interferon receptor agonist, Interleukin receptor agonist | |
| 303 | 5.21 | cp | triptolide | RNA polymerase inhibitor | RNA polymerase inhibitor | |
| 304 | 5.15 | cp | RHO-kinase-inhibitor-II | Rho associated kinase inhibitor | Rho associated kinase inhibitor | |
| 305 | 5.14 | cp | BRD-K63784565 | Topoisomerase inhibitor | Topoisomerase inhibitor | |
| 306 | 5.04 | cp | JX-401 | p38 MAPK inhibitor | p38 MAPK inhibitor | |
| 307 | 5.04 | cp | methoxsalen | DNA synthesis inhibitor | DNA synthesis inhibitor | |
| 308 | 5 | cp | atracurium | Acetylcholine receptor antagonist | Acetylcholine receptor antagonist | |
| 309 | 4.99 | cp | SB-203186 | Serotonin receptor antagonist | Serotonin receptor antagonist | |
| 310 | 4.96 | cp | altretamine | DNA synthesis inhibitor | DNA synthesis inhibitor | |
| 311 | 4.93 | cp | pravastatin | HMGCR inhibitor | HMGCR inhibitor | |
| 312 | 4.86 | cp | fenbufen | Cyclooxygenase inhibitor | Cyclooxygenase inhibitor | |
| 313 | 4.85 | cp | rhodomyrtoxin-b | sodium fluorescein uptake inhibitor | sodium fluorescein uptake inhibitor | |
| 314 | 4.81 | cp | purvalanol-b | Tyrosine kinase inhibitor | Tyrosine kinase inhibitor | |
| 315 | 4.76 | cp | dehydrocholic-acid | choleretic agent | choleretic agent | |
| 316 | 4.76 | cp | dantrolene | Calcium channel blocker | Calcium channel blocker | |
| 317 | 4.72 | cp | ergocryptine | Dopamine agonist | Dopamine agonist | |
| 318 | 4.72 | cp | VU-0415374-1 | Glutamate receptor modulator | Glutamate receptor modulator | |
| 319 | 4.69 | cp | STO-609 | Calmodulin antagonist | Calmodulin antagonist | |
| 320 | 4.67 | cp | cediranib | KIT inhibitor | KIT inhibitor, VEGFR inhibitor | |
| 321 | 4.67 | cp | bufalin | ATPase inhibitor | ATPase inhibitor | |
| 322 | 4.62 | cp | otenzepad | Acetylcholine receptor antagonist | Acetylcholine receptor antagonist | |
| 323 | 4.62 | cp | iproniazid | Monoamine oxidase inhibitor | Monoamine oxidase inhibitor | |
| 324 | 4.51 | cp | rivaroxaban | Coagulation inhibitor | Coagulation inhibitor | |
| 325 | 4.49 | cp | dichlorobenzamil | Sodium/calcium exchange inhibitor | Sodium/calcium exchange inhibitor | |
| 326 | 4.37 | cp | lysergol | Ergoline alkaloid | Ergoline alkaloid | |
| 327 | 4.37 | cp | nevirapine | Reverse transcriptase inhibitor | Reverse transcriptase inhibitor | |
| 328 | 4.3 | cp | KU-C103428N | CDC inhibitor | CDC inhibitor, Rho associated kinase inhibitor | |
| 329 | 4.29 | cp | cosmosiin | Cytochrome P450 inhibitor | Cytochrome P450 inhibitor | |
| 330 | 4.27 | cp | fatostatin | SREBP inhibitor | SREBP inhibitor | |
| 331 | 4.24 | cp | PD-198306 | MAP kinase inhibitor | MAP kinase inhibitor, MEK inhibitor | |
| 332 | 4.22 | cp | reversine | Aurora kinase inhibitor | Aurora kinase inhibitor | |
| 333 | 4.16 | cp | phenformin | AMPK activator | AMPK activator | |
| 334 | 4.15 | cp | amonafide | Topoisomerase inhibitor | Topoisomerase inhibitor | |
| 335 | 4.14 | cp | MK-2206 | AKT inhibitor | AKT inhibitor | |
| 336 | 4.12 | cp | ticlopidine | Purinergic receptor antagonist | Purinergic receptor antagonist | |
| 337 | 4.05 | cp | calpeptin | Calpain inhibitor | Calpain inhibitor | |
| 338 | 4.05 | cp | IKK-16 | IKK inhibitor | IKK inhibitor | |
| 339 | 3.91 | cp | penicillin | Bacterial cell wall synthesis inhibitor | Bacterial cell wall synthesis inhibitor | |
| 340 | 3.88 | cp | thenoyltrifluoroacetone | Chelating agent | Chelating agent | |
| 341 | 3.88 | cp | RG-13022 | PDGFR receptor inhibitor | PDGFR receptor inhibitor | |
| 342 | 3.82 | cp | AKT-inhibitor-1-2 | AKT inhibitor | AKT inhibitor | |
| 343 | 3.81 | cp | mifepristone | Glucocorticoid receptor antagonist | Glucocorticoid receptor antagonist, Progesterone receptor antagonist | |
| 344 | 3.79 | cp | levocabastine | Histamine receptor antagonist | Histamine receptor antagonist | |
| 345 | 3.77 | cp | pentobarbital | Barbiturate antiepileptic | Barbiturate antiepileptic, GABA receptor modulator | |
| 346 | 3.75 | cp | etoposide | Topoisomerase inhibitor | Topoisomerase inhibitor | |
| 347 | 3.74 | cp | KIN001-242 | Protein kinase inhibitor | Protein kinase inhibitor | |
| 348 | 3.74 | cp | MW-STK33-3B | Potassium channel activator | Potassium channel activator | |
| 349 | 3.7 | cp | naftopidil | Adrenergic receptor antagonist | Adrenergic receptor antagonist | |
| 350 | 3.66 | cp | avrainvillamide-analog-2 | nucleophosmin inhibitor | nucleophosmin inhibitor | |
| 351 | 3.65 | cp | trimebutine | Opioid receptor agonist | Opioid receptor agonist | |
| 352 | 3.63 | cp | mepivacaine | Potassium channel blocker | Potassium channel blocker, Sodium channel blocker | |
| 353 | 3.63 | cp | flavokavain-b | Antineoplastic | Antineoplastic | |
| 354 | 3.62 | cp | malonoben | Protein tyrosine kinase inhibitor | Protein tyrosine kinase inhibitor | |
| 355 | 3.59 | cp | KU-C103885 | Cystic fibrosis transmembrane conductance regulator inhibitor | Cystic fibrosis transmembrane conductance regulator inhibitor | |
| 356 | 3.56 | cp | hydroxycholesterol | LXR agonist | LXR agonist | |
| 357 | 3.56 | cp | fenobam | Glutamate receptor antagonist | Glutamate receptor antagonist | |
| 358 | 3.55 | cp | carbofuran | Cholinesterase inhibitor | Cholinesterase inhibitor | |
| 359 | 3.53 | cp | tetrahydropalmatine | Serotonin release inhibitor | Serotonin release inhibitor | |
| 360 | 3.52 | cp | XMD-885 | Leucine rich repeat kinase inhibitor | Leucine rich repeat kinase inhibitor, MAP kinase inhibitor | |
| 361 | 3.52 | cp | FR-180204 | -666 | -666 |  |
| 362 | 3.52 | cp | dactolisib | MTOR inhibitor | MTOR inhibitor, PI3K inhibitor, Protein kinase inhibitor | |
| 363 | 3.49 | cp | nornicotine | Acetylcholine receptor agonist | Acetylcholine receptor agonist | |
| 364 | 3.49 | cp | trap-101 | Nociceptin/orphanin FQ (NOP) receptor antagonist | Nociceptin/orphanin FQ (NOP) receptor antagonist, Opioid receptor antagonist | |
| 365 | 3.49 | cp | SA-792709 | Retinoid receptor agonist | Retinoid receptor agonist | |
| 366 | 3.46 | cp | QL-XI-92 | DDR1 inhibitor | DDR1 inhibitor | |
| 367 | 3.42 | cp | tyrphostin-AG-126 | ERK1 and ERK2 phosphorylation inhibitor | ERK1 and ERK2 phosphorylation inhibitor | |
| 368 | 3.42 | cp | oxalomalic-acid | Isocitrate dehydrogenase inhibitor | Isocitrate dehydrogenase inhibitor | |
| 369 | 3.42 | cp | meropenem | Bacterial cell wall synthesis inhibitor | Bacterial cell wall synthesis inhibitor | |
| 370 | 3.4 | cp | daphnetin | Protein kinase inhibitor | Protein kinase inhibitor | |
| 371 | 3.35 | cp | didanosine | Nucleoside reverse transcriptase inhibitor | Nucleoside reverse transcriptase inhibitor | |
| 372 | 3.35 | cp | WAY-629 | Serotonin receptor agonist | Serotonin receptor agonist | |
| 373 | 3.31 | cp | nalbuphine | Opioid receptor agonist | Opioid receptor agonist, Opioid receptor antagonist | |
| 374 | 3.3 | cp | VU-0400193-3 | Glutamate receptor modulator | Glutamate receptor modulator | |
| 375 | 3.29 | cp | triclabendazole | Microtubule inhibitor | Microtubule inhibitor | |
| 376 | 3.28 | cp | KIN001-220 | Aurora kinase inhibitor | Aurora kinase inhibitor | |
| 377 | 3.27 | cp | piceid | ICAM1 inhibitor | ICAM1 inhibitor | |
| 378 | 3.24 | cp | WT-171 | HDAC inhibitor | HDAC inhibitor | |
| 379 | 3.24 | cp | WAY-161503 | Serotonin receptor agonist | Serotonin receptor agonist | |
| 380 | 3.22 | cp | selinidin | Mast cell stabilizer | Mast cell stabilizer | |
| 381 | 3.21 | cp | EI-231 | Casein kinase inhibitor | Casein kinase inhibitor | |
| 382 | 3.18 | cp | carmofur | Thymidylate synthase inhibitor | Thymidylate synthase inhibitor | |
| 383 | 3.16 | cp | nateglinide | Insulin secretagogue | Insulin secretagogue | |
| 384 | 3.13 | cp | JNJ-10191584 | Histamine receptor antagonist | Histamine receptor antagonist | |
| 385 | 3.06 | cp | cardiogenol-c | Cardiomyogenesis inducer | Cardiomyogenesis inducer | |
| 386 | 3.03 | cp | equol | Estrogen receptor agonist | Estrogen receptor agonist | |
| 387 | 3.03 | cp | vicriviroc | CC chemokine receptor antagonist | CC chemokine receptor antagonist | |
| 388 | 3.03 | cp | EI-346-erlotinib-analog | EGFR inhibitor | EGFR inhibitor | |
| 389 | 3.03 | cp | dehydrocholic-acid | Bile acid | Bile acid | |
| 390 | 3.03 | cp | prostaglandin-e1 | Prostanoid receptor agonist | Prostanoid receptor agonist | |
| 391 | 3.03 | cp | NVP-TAE684 | ALK inhibitor | ALK inhibitor | |
| 392 | 2.96 | cp | mocetinostat | HDAC inhibitor | HDAC inhibitor | |
| 393 | 2.96 | cp | cefalexin | Bacterial cell wall synthesis inhibitor | Bacterial cell wall synthesis inhibitor | |
| 394 | 2.96 | cp | WAY-170523 | Metalloproteinase inhibitor | Metalloproteinase inhibitor | |
| 395 | 2.92 | cp | VX-702 | p38 MAPK inhibitor | p38 MAPK inhibitor | |
| 396 | 2.92 | cp | niacin | NAD precursor with lipid lowering effects | NAD precursor with lipid lowering effects, Vitamin B | |
| 397 | 2.92 | cp | BML-257 | AKT inhibitor | AKT inhibitor, HCV inhibitor | |
| 398 | 2.92 | cp | AG-592 | Tyrosine kinase inhibitor | Tyrosine kinase inhibitor | |
| 399 | 2.91 | cp | PF-04217903 | c-Met inhibitor | c-Met inhibitor | |
| 400 | 2.89 | cp | PRE-084 | Sigma receptor agonist | Sigma receptor agonist | |
| 401 | 2.85 | cp | tolterodine | Acetylcholine receptor antagonist | Acetylcholine receptor antagonist | |
| 402 | 2.82 | cp | APHA-compound-8 | HDAC inhibitor | HDAC inhibitor | |
| 403 | 2.82 | cp | tracazolate | GABA receptor modulator | GABA receptor modulator | |
| 404 | 2.82 | cp | OBAA | Phospholipase inhibitor | Phospholipase inhibitor | |
| 405 | 2.78 | cp | epothilone-a | Microtubule stabilizing agent | Microtubule stabilizing agent | |
| 406 | 2.75 | cp | latrepirdine | Glutamate receptor antagonist | Glutamate receptor antagonist | |
| 407 | 2.75 | cp | xylazine | Adrenergic receptor agonist | Adrenergic receptor agonist | |
| 408 | 2.75 | cp | maprotiline | Norepinephrine reuptake inhibitor | Norepinephrine reuptake inhibitor, Tricyclic antidepressant | |
| 409 | 2.71 | cp | lofexidine | Adrenergic receptor agonist | Adrenergic receptor agonist | |
| 410 | 2.71 | cp | alprazolam | Benzodiazepine receptor agonist | Benzodiazepine receptor agonist | |
| 411 | 2.71 | cp | chlorprothixene | Dopamine receptor antagonist | Dopamine receptor antagonist | |
| 412 | 2.69 | cp | tramadol | Norepinephrine reuptake inhibitor | Norepinephrine reuptake inhibitor, Opioid receptor agonist, Serotonin reuptake inhibitor | |
| 413 | 2.66 | cp | selumetinib | MEK inhibitor | MEK inhibitor | |
| 414 | 2.64 | cp | sulfasalazine | Antirheumatic | Antirheumatic, NFkB pathway inhibitor | |
| 415 | 2.64 | cp | calcifediol | Vitamin D receptor agonist | Vitamin D receptor agonist | |
| 416 | 2.61 | cp | enalaprilat | ACE inhibitor | ACE inhibitor | |
| 417 | 2.61 | cp | LE-135 | Retinoid receptor agonist | Retinoid receptor agonist | |
| 418 | 2.6 | cp | tyrphostin-AG-1288 | TNF production inhibitor | TNF production inhibitor | |
| 419 | 2.6 | cp | RO-3306 | CDK inhibitor | CDK inhibitor | |
| 420 | 2.54 | cp | tibolone | Androgen receptor agonist | Androgen receptor agonist, Estrogen receptor agonist, Progesterone receptor agonist | |
| 421 | 2.5 | cp | 4,5,6,7-tetrabromobenzotriazole | Casein kinase inhibitor | Casein kinase inhibitor | |
| 422 | 2.5 | cp | SCH-442416 | Adenosine receptor antagonist | Adenosine receptor antagonist | |
| 423 | 2.5 | cp | riluzole | Glutamate inhibitor | Glutamate inhibitor | |
| 424 | 2.5 | cp | L-741626 | Dopamine receptor antagonist | Dopamine receptor antagonist | |
| 425 | 2.5 | cp | YM-90709 | IL5 inhibitor | IL5 inhibitor | |
| 426 | 2.5 | cp | cladribine | Adenosine deaminase inhibitor | Adenosine deaminase inhibitor, Ribonucleoside reductase inhibitor | |
| 427 | 2.5 | cp | PJ-34 | PARP inhibitor | PARP inhibitor | |
| 428 | 2.47 | cp | SKF-77434 | Dopamine receptor agonist | Dopamine receptor agonist | |
| 429 | 2.47 | cp | GSK-1070916 | Aurora kinase inhibitor | Aurora kinase inhibitor | |
| 430 | 2.36 | cp | dofetilide | Potassium channel blocker | Potassium channel blocker | |
| 431 | 2.36 | cp | RS-504393 | CC chemokine receptor antagonist | CC chemokine receptor antagonist | |
| 432 | 2.33 | cp | pazufloxacin | Topoisomerase inhibitor | Topoisomerase inhibitor | |
| 433 | 2.33 | cp | bongkrek-acid | Mitochondrial ADP, ATP translocase inhibitor | Mitochondrial ADP, ATP translocase inhibitor | |
| 434 | 2.33 | cp | ZD-7155 | Angiotensin receptor antagonist | Angiotensin receptor antagonist | |
| 435 | 2.33 | cp | prostaglandin-b2 | cAMP inhibitor | cAMP inhibitor | |
| 436 | 2.33 | cp | Cyclo-[Arg-Gly-Asp-D-Phe-Val] | integrin antagonist | integrin antagonist | |
| 437 | 2.33 | cp | benzyl-quinazolin-4-yl-amine | EGFR inhibitor | EGFR inhibitor | |
| 438 | 2.33 | cp | olmesartan | Angiotensin receptor antagonist | Angiotensin receptor antagonist | |
| 439 | 2.32 | cp | NU-1025 | PARP inhibitor | PARP inhibitor, DNA dependent protein kinase inhibitor | |
| 440 | 2.29 | cp | AG-957 | Protein tyrosine kinase inhibitor | Protein tyrosine kinase inhibitor | |
| 441 | 2.29 | cp | clopidogrel | Purinergic receptor antagonist | Purinergic receptor antagonist | |
| 442 | 2.26 | cp | anastrozole | Aromatase inhibitor | Aromatase inhibitor | |
| 443 | 2.26 | cp | formoterol | Adrenergic receptor agonist | Adrenergic receptor agonist | |
| 444 | 2.24 | cp | haloperidol | Dopamine receptor antagonist | Dopamine receptor antagonist, Psychoactive drug | |
| 445 | 2.23 | cp | prilocaine | Local anesthetic | Local anesthetic | |
| 446 | 2.22 | cp | BIX-01338 | Histone lysine methyltransferase inhibitor | Histone lysine methyltransferase inhibitor | |
| 447 | 2.22 | cp | IKK-2-inhibitor-V | IKK inhibitor | IKK inhibitor, NFkB pathway inhibitor | |
| 448 | 2.18 | cp | WYE-125132 | MTOR inhibitor | MTOR inhibitor | |
| 449 | 2.15 | cp | mead-acid | KPL-1 tumor suppressor | KPL-1 tumor suppressor | |
| 450 | 2.15 | cp | H-9 | PKA inhibitor | PKA inhibitor | |
| 451 | 2.15 | cp | CGS-12066B | Serotonin receptor agonist | Serotonin receptor agonist | |
| 452 | 2.11 | cp | ZK-164015 | Estrogen receptor antagonist | Estrogen receptor antagonist | |
| 453 | 2.11 | cp | hippeastrine | Plant alkaloid | Plant alkaloid | |
| 454 | 2.1 | cp | ubenimex | Leukotriene inhibitor | Leukotriene inhibitor | |
| 455 | 2.08 | cp | paxilline | Potassium channel blocker | Potassium channel blocker | |
| 456 | 2.08 | cp | dihydroergocristine | Adrenergic receptor antagonist | Adrenergic receptor antagonist, Prolactin inhibitor | |
| 457 | 2.08 | cp | saracatinib | SRC inhibitor | SRC inhibitor | |
| 458 | 2.06 | cp | mibefradil | T-type calcium channel blocker | T-type calcium channel blocker | |
| 459 | 2.04 | cp | acetyl-geranyl-cysteine | Isoprenylated protein methylation inhibitor | Isoprenylated protein methylation inhibitor | |
| 460 | 2.04 | cp | propylpyrazole | Estrogen receptor agonist | Estrogen receptor agonist | |
| 461 | 2.04 | cp | HA-1004 | Calcium channel blocker | Calcium channel blocker | |
| 462 | 2.04 | cp | diphenidol | Acetylcholine receptor agonist | Acetylcholine receptor agonist | |
| 463 | 2.04 | cp | labetalol | Adrenergic receptor antagonist | Adrenergic receptor antagonist | |
| 464 | 2.01 | cp | miglitol | Glucosidase inhibitor | Glucosidase inhibitor | |
| 465 | 2.01 | cp | honokiol | AKT inhibitor | AKT inhibitor | |
| 466 | 2.01 | cp | SB-366791 | TRPV antagonist | TRPV antagonist | |
| 467 | 2.01 | cp | GYKI-52466 | Glutamate receptor antagonist | Glutamate receptor antagonist, Kainate receptor antagonist | |
| 468 | 2.01 | cp | caffeic-acid | Lipoxygenase inhibitor | Lipoxygenase inhibitor, HIV integrase inhibitor, NFkB pathway inhibitor, Nitric oxide production inhibitor, PPAR receptor modulator, TNF production inhibitor, Tumor necrosis factor production inhibitor | |
| 469 | 2.01 | cp | irbesartan | Angiotensin receptor antagonist | Angiotensin receptor antagonist | |
| 470 | 2 | cp | estradiol-cypionate | Estrogen receptor agonist | Estrogen receptor agonist | |
| 471 | 1.97 | cp | PD-166793 | Metalloproteinase inhibitor | Metalloproteinase inhibitor, Collagenase inhibitor | |
| 472 | 1.97 | cp | kenpaullone | CDK inhibitor | CDK inhibitor, Glycogen synthase kinase inhibitor | |
| 473 | 1.97 | cp | DNQX | Glutamate receptor antagonist | Glutamate receptor antagonist | |
| 474 | 1.94 | cp | everolimus | MTOR inhibitor | MTOR inhibitor | |
| 475 | 1.94 | cp | thiostrepton | FOXM1 inhibitor | FOXM1 inhibitor, Protein synthesis inhibitor | |
| 476 | 1.94 | cp | quercetin | Polar auxin transport inhibitor | Polar auxin transport inhibitor | |
| 477 | 1.94 | cp | MMPX | Phosphodiesterase inhibitor | Phosphodiesterase inhibitor | |
| 478 | 1.94 | cp | cortisone | Glucocorticoid receptor agonist | Glucocorticoid receptor agonist | |
| 479 | 1.93 | cp | torasemide | Electrolyte reabsorption inhibitor | Electrolyte reabsorption inhibitor, Thromboxane receptor antagonist | |
| 480 | 1.92 | cp | prednisolone | Glucocorticoid receptor agonist | Glucocorticoid receptor agonist | |
| 481 | 1.9 | cp | PF-543 | Sphingosine kinase inhibitor | Sphingosine kinase inhibitor | |
| 482 | 1.9 | cp | diethyltoluamide | DEET activator of fly antenna ionotropic receptor IR40a | DEET activator of fly antenna ionotropic receptor IR40a | |
| 483 | 1.9 | cp | IB-MECA | Adenosine receptor agonist | Adenosine receptor agonist | |
| 484 | 1.9 | cp | gabazine | GABA receptor antagonist | GABA receptor antagonist | |
| 485 | 1.9 | cp | ispinesib | Kinesin-like spindle protein inhibitor | Kinesin-like spindle protein inhibitor | |
| 486 | 1.88 | cp | oxiconazole | Bacterial cell wall synthesis inhibitor | Bacterial cell wall synthesis inhibitor | |
| 487 | 1.87 | cp | phenelzine | Monoamine oxidase inhibitor | Monoamine oxidase inhibitor | |
| 488 | 1.87 | cp | PI-103 | MTOR inhibitor | MTOR inhibitor, PI3K inhibitor | |
| 489 | 1.87 | cp | MDL-28170 | Calpain inhibitor | Calpain inhibitor | |
| 490 | 1.87 | cp | propylthiouracil | Thyroid peroxidase inhibitor | Thyroid peroxidase inhibitor | |
| 491 | 1.87 | cp | eliprodil | Glutamate receptor antagonist | Glutamate receptor antagonist | |
| 492 | 1.83 | cp | acepromazine | Dopamine receptor antagonist | Dopamine receptor antagonist | |
| 493 | 1.83 | cp | fluoxetine | Selective serotonin reuptake inhibitor (SSRI) | Selective serotonin reuptake inhibitor (SSRI) | |
| 494 | 1.83 | cp | bepridil | Calcium channel blocker | Calcium channel blocker, L-type calcium channel blocker | |
| 495 | 1.8 | cp | dihydrexidine | Dopamine receptor agonist | Dopamine receptor agonist | |
| 496 | 1.8 | cp | L-745870 | Dopamine receptor antagonist | Dopamine receptor antagonist | |
| 497 | 1.8 | cp | cefotiam | Bacterial cell wall synthesis inhibitor | Bacterial cell wall synthesis inhibitor | |
| 498 | 1.78 | cp | nimetazepam | GABA receptor agonist | GABA receptor agonist | |
| 499 | 1.76 | cp | SCH-23390 | Dopamine receptor antagonist | Dopamine receptor antagonist | |
| 500 | 1.76 | cp | rhamnetin | HDAC inhibitor | HDAC inhibitor | |
| 501 | 1.71 | cp | indinavir | HIV protease inhibitor | HIV protease inhibitor | |
| 502 | 1.69 | cp | FR-139317 | Endothelin receptor antagonist | Endothelin receptor antagonist | |
| 503 | 1.69 | cp | dopamine | Dopamine receptor agonist | Dopamine receptor agonist | |
| 504 | 1.69 | cp | PD-0325901 | MEK inhibitor | MEK inhibitor, MAP kinase inhibitor, Protein kinase inhibitor | |
| 505 | 1.66 | cp | MRS-1220 | Adenosine receptor antagonist | Adenosine receptor antagonist | |
| 506 | 1.66 | cp | doxylamine | Histamine receptor antagonist | Histamine receptor antagonist | |
| 507 | 1.66 | cp | homatropine | Acetylcholine receptor antagonist | Acetylcholine receptor antagonist | |
| 508 | 1.62 | cp | apigenin | Casein kinase inhibitor | Casein kinase inhibitor, Cell proliferation inhibitor, Cytochrome P450 inhibitor | |
| 509 | 1.62 | cp | tyrphostin-AG-538 | IGF-1 inhibitor | IGF-1 inhibitor | |
| 510 | 1.59 | cp | ibudilast | Leukotriene receptor antagonist | Leukotriene receptor antagonist, Phosphodiesterase inhibitor | |
| 511 | 1.59 | cp | pheniramine | Histamine receptor antagonist | Histamine receptor antagonist | |
| 512 | 1.58 | cp | secoisolariciresinol | Antioxidant | Antioxidant | |
| 513 | 1.58 | cp | mepacrine | Cytokine production inhibitor | Cytokine production inhibitor, NFkB pathway inhibitor, TP53 activator | |
| 514 | 1.55 | cp | razoxane | Chelating agent | Chelating agent, Topoisomerase inhibitor | |
| 515 | 1.55 | cp | raclopride | Dopamine receptor antagonist | Dopamine receptor antagonist | |
| 516 | 1.55 | cp | erythromycin | NFkB pathway inhibitor | NFkB pathway inhibitor | |
| 517 | 1.55 | cp | daidzein | Estrogen receptor agonist | Estrogen receptor agonist | |
| 518 | 1.52 | cp | piretanide | Glucocorticoid receptor agonist | Glucocorticoid receptor agonist | |
| 519 | 1.52 | cp | NU-7026 | DNA dependent protein kinase inhibitor | DNA dependent protein kinase inhibitor, MTOR inhibitor, PI3K inhibitor | |
| 520 | 1.47 | cp | olvanil | TRPV agonist | TRPV agonist | |
| 521 | 1.47 | cp | eudesmic-acid | -666 | -666 |  |
| 522 | 1.45 | cp | estriol | Estrogen receptor agonist | Estrogen receptor agonist | |
| 523 | 1.44 | cp | benzbromarone | Chloride channel blocker | Chloride channel blocker | |
| 524 | 1.44 | cp | FTI-276 | Farnesyltransferase inhibitor | Farnesyltransferase inhibitor | |
| 525 | 1.44 | cp | L-701252 | Glutamate receptor antagonist | Glutamate receptor antagonist | |
| 526 | 1.44 | cp | benzthiazide | Carbonic anhydrase inhibitor | Carbonic anhydrase inhibitor | |
| 527 | 1.41 | cp | GDC-0879 | RAF inhibitor | RAF inhibitor | |
| 528 | 1.41 | cp | KUC103420N | -666 | -666 |  |
| 529 | 1.41 | cp | BMS-641988 | Androgen receptor antagonist | Androgen receptor antagonist | |
| 530 | 1.41 | cp | seneciphylline | Cytochrome P450 inhibitor | Cytochrome P450 inhibitor | |
| 531 | 1.41 | cp | NAS-181 | Serotonin receptor antagonist | Serotonin receptor antagonist | |
| 532 | 1.41 | cp | digoxin | ATPase inhibitor | ATPase inhibitor | |
| 533 | 1.37 | cp | pantoprazole | ATPase inhibitor | ATPase inhibitor | |
| 534 | 1.37 | cp | mestranol | Estrogen receptor agonist | Estrogen receptor agonist | |
| 535 | 1.37 | cp | RS-39604 | Serotonin receptor antagonist | Serotonin receptor antagonist | |
| 536 | 1.36 | cp | fenpiverinium | Acetylcholine receptor antagonist | Acetylcholine receptor antagonist | |
| 537 | 1.34 | cp | JWE-035 | Aurora kinase inhibitor | Aurora kinase inhibitor | |
| 538 | 1.34 | cp | pterostilbene | Cyclooxygenase inhibitor | Cyclooxygenase inhibitor, PPAR receptor agonist | |
| 539 | 1.34 | cp | scopolamine | Acetylcholine receptor antagonist | Acetylcholine receptor antagonist | |
| 540 | 1.34 | cp | tomelukast | Leukotriene receptor antagonist | Leukotriene receptor antagonist | |
| 541 | 1.34 | cp | UNC-0321 | Histone lysine methyltransferase inhibitor | Histone lysine methyltransferase inhibitor | |
| 542 | 1.3 | cp | parecoxib | Cyclooxygenase inhibitor | Cyclooxygenase inhibitor | |
| 543 | 1.3 | cp | dibutyrylcyclic-gmp | cGMP analog | cGMP analog | |
| 544 | 1.3 | cp | xanthoxyline | Antifungal | Antifungal | |
| 545 | 1.3 | cp | SB-408124 | Orexin receptor antagonist | Orexin receptor antagonist | |
| 546 | 1.3 | cp | rutin | Antioxidant | Antioxidant, Capillary stabilizing agent, Nitric oxide scavenger | |
| 547 | 1.27 | cp | benactyzine | Acetylcholine receptor antagonist | Acetylcholine receptor antagonist | |
| 548 | 1.27 | cp | YS-035 | Calcium channel blocker | Calcium channel blocker | |
| 549 | 1.27 | cp | SB-205384 | GABA receptor modulator | GABA receptor modulator | |
| 550 | 1.27 | cp | fananserin | Dopamine receptor antagonist | Dopamine receptor antagonist, Serotonin receptor antagonist | |
| 551 | 1.27 | cp | resveratrol | Cytochrome P450 inhibitor | Cytochrome P450 inhibitor, SIRT activator | |
| 552 | 1.23 | cp | cinacalcet | Calcium channel activator | Calcium channel activator | |
| 553 | 1.23 | cp | AZD-8055 | MTOR inhibitor | MTOR inhibitor | |
| 554 | 1.23 | cp | danusertib | Aurora kinase inhibitor | Aurora kinase inhibitor, Growth factor receptor inhibitor | |
| 555 | 1.22 | cp | cephalotaxine | Protein synthesis inhibitor | Protein synthesis inhibitor | |
| 556 | 1.21 | cp | fusaric-acid | Dopamine beta hydroxylase inhibitor | Dopamine beta hydroxylase inhibitor | |
| 557 | 1.2 | cp | XL-147 | PI3K inhibitor | PI3K inhibitor | |
| 558 | 1.16 | cp | RO-60-0175 | Serotonin receptor agonist | Serotonin receptor agonist | |
| 559 | 1.16 | cp | purmorphamine | Smoothened receptor agonist | Smoothened receptor agonist | |
| 560 | 1.13 | cp | eplerenone | Cytochrome P450 inhibitor | Cytochrome P450 inhibitor | |
| 561 | 1.13 | cp | mirtazapine | Adrenergic receptor antagonist | Adrenergic receptor antagonist, Serotonin receptor antagonist | |
| 562 | 1.13 | cp | levomepromazine | Dopamine receptor antagonist | Dopamine receptor antagonist | |
| 563 | 1.12 | cp | PSB-1115 | Adenosine receptor antagonist | Adenosine receptor antagonist | |
| 564 | 1.09 | cp | CGS-20625 | Benzodiazepine receptor agonist | Benzodiazepine receptor agonist, GABA benzodiazepine site receptor partial agonist | |
| 565 | 1.09 | cp | tetramisole | Immunostimulant | Immunostimulant | |
| 566 | 1.09 | cp | linezolid | Bacterial 50S ribosomal subunit inhibitor | Bacterial 50S ribosomal subunit inhibitor | |
| 567 | 1.08 | cp | phosphodiesterase-V-inhibitor-II | Phosphodiesterase inhibitor | Phosphodiesterase inhibitor | |
| 568 | 1.08 | cp | noretynodrel | Progestogen hormone | Progestogen hormone | |
| 569 | 1.06 | cp | delcorine | Antiarrhythmic | Antiarrhythmic, Ganglionic blocker | |
| 570 | 1.06 | cp | indolophenanthridine | CALY activator | CALY activator | |
| 571 | 1.06 | cp | AM-281 | Cannabinoid receptor antagonist | Cannabinoid receptor antagonist | |
| 572 | 1.06 | cp | PD-98059 | MEK inhibitor | MEK inhibitor, MAP kinase inhibitor | |
| 573 | 1.02 | cp | tangeritin | Cell cycle inhibitor | Cell cycle inhibitor | |
| 574 | 1.02 | cp | norgestrel | Progesterone receptor agonist | Progesterone receptor agonist | |
| 575 | 1.02 | cp | DMAB-anabaseine | Adrenergic receptor agonist | Adrenergic receptor agonist | |
| 576 | 1.02 | cp | arvanil | TRPV agonist | TRPV agonist | |
| 577 | 1.02 | cp | diloxanide | Protein synthesis inhibitor | Protein synthesis inhibitor | |
| 578 | 1 | cp | z-leu3-VS | Proteasome inhibitor | Proteasome inhibitor | |
| 579 | 0.99 | cp | masitinib | KIT inhibitor | KIT inhibitor, PDGFR receptor inhibitor, SRC inhibitor | |
| 580 | 0.99 | cp | alitretinoin | Retinoid receptor agonist | Retinoid receptor agonist | |
| 581 | 0.99 | cp | SYK-inhibitor | SYK inhibitor | SYK inhibitor | |
| 582 | 0.99 | cp | metoprolol | Adrenergic receptor antagonist | Adrenergic receptor antagonist | |
| 583 | 0.99 | cp | colforsin | Adenylyl cyclase activator | Adenylyl cyclase activator, Adenylate cyclase stimulant | |
| 584 | 0.99 | cp | amsacrine | Topoisomerase inhibitor | Topoisomerase inhibitor | |
| 585 | 0.99 | cp | minaprine | Serotonin reuptake inhibitor | Serotonin reuptake inhibitor | |
| 586 | 0.98 | cp | phenoxybenzamine | Adrenergic receptor antagonist | Adrenergic receptor antagonist | |
| 587 | 0.95 | cp | cefpodoxime | Bacterial cell wall synthesis inhibitor | Bacterial cell wall synthesis inhibitor | |
| 588 | 0.95 | cp | epigallocatechin | Nitric oxide synthase inhibitor | Nitric oxide synthase inhibitor, Bacterial DNA gyrase inhibitor | |
| 589 | 0.95 | cp | PPT | Estrogen receptor agonist | Estrogen receptor agonist | |
| 590 | 0.95 | cp | GR-127935 | Serotonin receptor antagonist | Serotonin receptor antagonist | |
| 591 | 0.95 | cp | BRD-K65285700 | Cannabinoid receptor agonist | Cannabinoid receptor agonist | |
| 592 | 0.95 | cp | CPCCOEt | Glutamate receptor antagonist | Glutamate receptor antagonist | |
| 593 | 0.95 | cp | bifemelane | Acetylcholine release stimulant | Acetylcholine release stimulant, Adrenergic transmitter uptake inhibitor, Reducing agent | |
| 594 | 0.95 | cp | RHO-kinase-inhibitor-III[rockout] | Rho associated kinase inhibitor | Rho associated kinase inhibitor | |
| 595 | 0.92 | cp | dextrorphan | Glutamate receptor antagonist | Glutamate receptor antagonist | |
| 596 | 0.92 | cp | W-13 | Calmodulin antagonist | Calmodulin antagonist | |
| 597 | 0.92 | cp | SKF-89976A | GABA uptake inhibitor | GABA uptake inhibitor | |
| 598 | 0.92 | cp | mycophenolate-mofetil | Dehydrogenase inhibitor | Dehydrogenase inhibitor, Hydroxycarboxylic acid receptor agonist, Immunosuppressant, Inosine monophosphate dehydrogenase inhibitor, Inositol monophosphatase inhibitor | |
| 599 | 0.92 | cp | GW-6471 | PPAR receptor antagonist | PPAR receptor antagonist | |
| 600 | 0.92 | cp | cirazoline | Adrenergic receptor agonist | Adrenergic receptor agonist | |
| 601 | 0.92 | cp | CL-218872 | GABA receptor agonist | GABA receptor agonist | |
| 602 | 0.89 | cp | tamoxifen | Estrogen receptor antagonist | Estrogen receptor antagonist, Selective estrogen receptor modulator (SERM) | |
| 603 | 0.88 | cp | RS-100329 | Adrenergic receptor antagonist | Adrenergic receptor antagonist | |
| 604 | 0.88 | cp | NNC-55-0396 | T-type calcium channel blocker | T-type calcium channel blocker | |
| 605 | 0.88 | cp | FK-888 | Tachykinin antagonist | Tachykinin antagonist | |
| 606 | 0.88 | cp | rofecoxib | Cyclooxygenase inhibitor | Cyclooxygenase inhibitor | |
| 607 | 0.88 | cp | dexamethasone | Glucocorticoid receptor agonist | Glucocorticoid receptor agonist, Cytochrome P450 inhibitor, Glucocorticoid receptor modulator, Corticosteroid agonist | |
| 608 | 0.88 | cp | milrinone | Phosphodiesterase inhibitor | Phosphodiesterase inhibitor | |
| 609 | 0.88 | cp | adipiodone | Contrast agent | Contrast agent | |
| 610 | 0.85 | cp | KI-16425 | Lysophosphatidic acid receptor antagonist | Lysophosphatidic acid receptor antagonist | |
| 611 | 0.85 | cp | sulforaphane | Antineoplastic | Antineoplastic, Aryl hydrocarbon receptor antagonist | |
| 612 | 0.85 | cp | bulleyaconitine-a | Non-opiod analgesic | Non-opiod analgesic | |
| 613 | 0.85 | cp | norcyclobenzaprine | Adrenergic receptor agonist | Adrenergic receptor agonist, Serotonin receptor antagonist | |
| 614 | 0.85 | cp | CDK2-5-inhibitor | CDK inhibitor | CDK inhibitor | |
| 615 | 0.84 | cp | hexamethylenebisacetamide | AKT inhibitor | AKT inhibitor, Differentiation inducer, NFkB pathway inhibitor | |
| 616 | 0.81 | cp | salsolinol | Monoamine oxidase inhibitor | Monoamine oxidase inhibitor, Tyrosine hydroxylase inhibitor | |
| 617 | 0.78 | cp | lomerizine | Calcium channel blocker | Calcium channel blocker | |
| 618 | 0.78 | cp | myriocin | Serine palmitoyltransferase inhibitor | Serine palmitoyltransferase inhibitor | |
| 619 | 0.78 | cp | flucloxacillin | Bacterial cell wall synthesis inhibitor | Bacterial cell wall synthesis inhibitor | |
| 620 | 0.78 | cp | DH-97 | Melatonin receptor antagonist | Melatonin receptor antagonist | |
| 621 | 0.78 | cp | bromocriptine | Dopamine receptor agonist | Dopamine receptor agonist | |
| 622 | 0.74 | cp | PD-184352 | MEK inhibitor | MEK inhibitor | |
| 623 | 0.74 | cp | nafadotride | Dopamine receptor antagonist | Dopamine receptor antagonist | |
| 624 | 0.74 | cp | flumetasone | Glucocorticoid receptor agonist | Glucocorticoid receptor agonist | |
| 625 | 0.74 | cp | diclofenamide | Carbonic anhydrase inhibitor | Carbonic anhydrase inhibitor | |
| 626 | 0.74 | cp | DAPT-GSI-IX | Gamma secretase inhibitor | Gamma secretase inhibitor | |
| 627 | 0.74 | cp | androsta-1,4-dien-3,17-dione | Aromatase inhibitor | Aromatase inhibitor | |
| 628 | 0.74 | cp | alfuzosin | Adrenergic receptor antagonist | Adrenergic receptor antagonist | |
| 629 | 0.71 | cp | isoeugenol | Nitric oxide production inhibitor | Nitric oxide production inhibitor | |
| 630 | 0.71 | cp | arcyriaflavin-a | CDK inhibitor | CDK inhibitor | |
| 631 | 0.7 | cp | nilotinib | ABL inhibitor | ABL inhibitor, BCR-ABL kinase inhibitor | |
| 632 | 0.7 | cp | rifapentine | RNA polymerase inhibitor | RNA polymerase inhibitor | |
| 633 | 0.7 | cp | EMF-bca1-57 | caspase inhibitor | caspase inhibitor | |
| 634 | 0.7 | cp | EMF-BCA1-64 | Caspase inhibitor | Caspase inhibitor | |
| 635 | 0.7 | cp | zolantidine | Histamine receptor antagonist | Histamine receptor antagonist | |
| 636 | 0.7 | cp | testosterone | androgen receptor agonist | androgen receptor agonist | |
| 637 | 0.7 | cp | methylergometrine | Dopamine receptor antagonist | Dopamine receptor antagonist, Serotonin receptor antagonist | |
| 638 | 0.7 | cp | dorsomorphin | AMPK inhibitor | AMPK inhibitor | |
| 639 | 0.7 | cp | dipyridamole | Phosphodiesterase inhibitor | Phosphodiesterase inhibitor | |
| 640 | 0.7 | cp | oxfendazole | Anthelmintic | Anthelmintic | |
| 641 | 0.7 | cp | buphenine | Adrenergic receptor agonist | Adrenergic receptor agonist | |
| 642 | 0.7 | cp | meclofenamic-acid | Cyclooxygenase inhibitor | Cyclooxygenase inhibitor, Prostanoid receptor antagonist | |
| 643 | 0.7 | cp | doxazosin | Adrenergic receptor antagonist | Adrenergic receptor antagonist | |
| 644 | 0.7 | cp | remacemide | Glutamate receptor antagonist | Glutamate receptor antagonist | |
| 645 | 0.7 | cp | tropicamide | Acetylcholine receptor antagonist | Acetylcholine receptor antagonist | |
| 646 | 0.67 | cp | sulpiride | Dopamine receptor antagonist | Dopamine receptor antagonist | |
| 647 | 0.67 | cp | fostamatinib | SYK inhibitor | SYK inhibitor | |
| 648 | 0.67 | cp | MEK1-2-inhibitor | MEK inhibitor | MEK inhibitor | |
| 649 | 0.67 | cp | finasteride | 5-alpha reductase inhibitor | 5-alpha reductase inhibitor | |
| 650 | 0.67 | cp | CI-966 | GAT inhibitor | GAT inhibitor | |
| 651 | 0.67 | cp | XE-991 | Potassium channel blocker | Potassium channel blocker | |
| 652 | 0.67 | cp | protein-tyrosine-phosphatase-inhibitor-IV | Tyrosine phosphatase inhibitor | Tyrosine phosphatase inhibitor | |
| 653 | 0.67 | cp | L-655708 | GABA receptor inverse agonist | GABA receptor inverse agonist | |
| 654 | 0.67 | cp | ICI-118551 | Adrenergic receptor antagonist | Adrenergic receptor antagonist | |
| 655 | 0.67 | cp | tozasertib | Aurora kinase inhibitor | Aurora kinase inhibitor, BCR-ABL kinase inhibitor, FLT3 inhibitor, JAK inhibitor | |
| 656 | 0.64 | cp | cortisone | Glucocorticoid receptor agonist | Glucocorticoid receptor agonist | |
| 657 | 0.63 | cp | erlotinib | EGFR inhibitor | EGFR inhibitor | |
| 658 | 0.63 | cp | trioxsalen | DNA synthesis inhibitor | DNA synthesis inhibitor | |
| 659 | 0.63 | cp | isoflupredone | Glucocorticoid receptor agonist | Glucocorticoid receptor agonist | |
| 660 | 0.63 | cp | EO-1428 | p38 MAPK inhibitor | p38 MAPK inhibitor | |
| 661 | 0.63 | cp | enrofloxacin | Bacterial DNA gyrase inhibitor | Bacterial DNA gyrase inhibitor | |
| 662 | 0.63 | cp | benzopurpurin-4b | HIV entry inhibitor | HIV entry inhibitor | |
| 663 | 0.63 | cp | nimodipine | Calcium channel blocker | Calcium channel blocker | |
| 664 | 0.63 | cp | HTMT | Histamine receptor agonist | Histamine receptor agonist | |
| 665 | 0.63 | cp | rucaparib | PARP inhibitor | PARP inhibitor | |
| 666 | 0.63 | cp | indapamide | Thiazide diuretic | Thiazide diuretic | |
| 667 | 0.62 | cp | letrozole | Aromatase inhibitor | Aromatase inhibitor | |
| 668 | 0.6 | cp | acarbose | Glucosidase inhibitor | Glucosidase inhibitor | |
| 669 | 0.6 | cp | remoxipride | Dopamine receptor antagonist | Dopamine receptor antagonist | |
| 670 | 0.6 | cp | tebuthiuron | Photosynthesis inhibitor | Photosynthesis inhibitor | |
| 671 | 0.6 | cp | SIB-1893 | Glutamate receptor antagonist | Glutamate receptor antagonist | |
| 672 | 0.6 | cp | KU-55933 | ATM kinase inhibitor | ATM kinase inhibitor | |
| 673 | 0.6 | cp | benperidol | Dopamine receptor antagonist | Dopamine receptor antagonist | |
| 674 | 0.6 | cp | ipratropium | Acetylcholine receptor antagonist | Acetylcholine receptor antagonist | |
| 675 | 0.56 | cp | nelfinavir | HIV protease inhibitor | HIV protease inhibitor | |
| 676 | 0.56 | cp | midazolam | Benzodiazepine receptor agonist | Benzodiazepine receptor agonist | |
| 677 | 0.56 | cp | PF-3845 | FAAH inhibitor | FAAH inhibitor | |
| 678 | 0.56 | cp | AR-C133057XX | Nitric oxide synthase inhibitor | Nitric oxide synthase inhibitor | |
| 679 | 0.56 | cp | zatebradine | HCN channel blocker | HCN channel blocker | |
| 680 | 0.56 | cp | modafinil | Adrenergic receptor agonist | Adrenergic receptor agonist | |
| 681 | 0.56 | cp | GR-89696 | Opioid receptor agonist | Opioid receptor agonist | |
| 682 | 0.56 | cp | dicyclohexylurea | Epoxide hydolase inhibitor | Epoxide hydolase inhibitor | |
| 683 | 0.56 | cp | apoptosis-activator-II | Carboxylesterase inhibitor | Carboxylesterase inhibitor | |
| 684 | 0.56 | cp | amthamine | Histamine receptor agonist | Histamine receptor agonist | |
| 685 | 0.56 | cp | wortmannin | PI3K inhibitor | PI3K inhibitor | |
| 686 | 0.56 | cp | TAS-301 | Calcium-calmodulin dependent protein kinase inhibitor | Calcium-calmodulin dependent protein kinase inhibitor | |
| 687 | 0.55 | cp | BRD-K52219182 | Phosphodiesterase inhibitor | Phosphodiesterase inhibitor | |
| 688 | 0.53 | cp | zardaverine | Phosphodiesterase inhibitor | Phosphodiesterase inhibitor | |
| 689 | 0.53 | cp | SR-57227A | Serotonin receptor agonist | Serotonin receptor agonist | |
| 690 | 0.53 | cp | EXO-1 | ARF inhibitor | ARF inhibitor | |
| 691 | 0.53 | cp | protopine | Histamine receptor antagonist | Histamine receptor antagonist | |
| 692 | 0.53 | cp | cisapride | Serotonin receptor agonist | Serotonin receptor agonist | |
| 693 | 0.53 | cp | fexofenadine | Histamine receptor antagonist | Histamine receptor antagonist | |
| 694 | 0.53 | cp | racecadotril | Enkephalinase inhibitor | Enkephalinase inhibitor | |
| 695 | 0.53 | cp | geldanamycin | HSP inhibitor | HSP inhibitor | |
| 696 | 0.52 | cp | valaciclovir | DNA polymerase inhibitor | DNA polymerase inhibitor | |
| 697 | 0.49 | cp | AVA | Nucleophosmin inhibitor | Nucleophosmin inhibitor | |
| 698 | 0.49 | cp | kitasamycin | Protein synthesis inhibitor | Protein synthesis inhibitor | |
| 699 | 0.49 | cp | clarithromycin | Bacterial 50S ribosomal subunit inhibitor | Bacterial 50S ribosomal subunit inhibitor | |
| 700 | 0.49 | cp | UCL-2077 | Slow after hyperpolarization channel blocker | Slow after hyperpolarization channel blocker | |
| 701 | 0.49 | cp | vesamicol | Acetylcholinesterase inhibitor | Acetylcholinesterase inhibitor | |
| 702 | 0.49 | cp | ITE | Aryl hydrocarbon receptor agonist | Aryl hydrocarbon receptor agonist | |
| 703 | 0.49 | cp | PETCM | Caspase activator | Caspase activator | |
| 704 | 0.49 | cp | ML-9 | Myosin light chain kinase inhibitor | Myosin light chain kinase inhibitor | |
| 705 | 0.49 | cp | guanabenz | -666 | -666 |  |
| 706 | 0.49 | cp | nadolol | Adrenergic receptor antagonist | Adrenergic receptor antagonist | |
| 707 | 0.47 | cp | SA-792728 | Sphingosine kinase inhibitor | Sphingosine kinase inhibitor | |
| 708 | 0.46 | cp | benidipine | Calcium channel blocker | Calcium channel blocker | |
| 709 | 0.46 | cp | BMS-754807 | IGF-1 inhibitor | IGF-1 inhibitor | |
| 710 | 0.46 | cp | vanoxerine | Dopamine uptake inhibitor | Dopamine uptake inhibitor | |
| 711 | 0.46 | cp | tadalafil | Phosphodiesterase inhibitor | Phosphodiesterase inhibitor | |
| 712 | 0.46 | cp | mephentermine | Adrenergic receptor agonist | Adrenergic receptor agonist | |
| 713 | 0.46 | cp | phenazopyridine | Local anesthetic | Local anesthetic | |
| 714 | 0.42 | cp | thiocolchicoside | GABA receptor antagonist | GABA receptor antagonist | |
| 715 | 0.42 | cp | moxonidine | Imidazoline receptor agonist | Imidazoline receptor agonist | |
| 716 | 0.42 | cp | L-733060 | Tachykinin antagonist | Tachykinin antagonist | |
| 717 | 0.42 | cp | J-104129 | Acetylcholine receptor antagonist | Acetylcholine receptor antagonist | |
| 718 | 0.42 | cp | quinisocaine | Local anesthetic | Local anesthetic | |
| 719 | 0.42 | cp | BAY-36-7620 | Glutamate receptor antagonist | Glutamate receptor antagonist | |
| 720 | 0.42 | cp | propentofylline | Adenosine reuptake inhibitor | Adenosine reuptake inhibitor, Phosphodiesterase inhibitor | |
| 721 | 0.42 | cp | pirenperone | Serotonin receptor antagonist | Serotonin receptor antagonist | |
| 722 | 0.42 | cp | midodrine | Adrenergic receptor agonist | Adrenergic receptor agonist | |
| 723 | 0.42 | cp | linopirdine | Potassium channel blocker | Potassium channel blocker | |
| 724 | 0.42 | cp | dilazep | Adenosine reuptake inhibitor | Adenosine reuptake inhibitor | |
| 725 | 0.42 | cp | isotretinoin | Retinoid receptor agonist | Retinoid receptor agonist | |
| 726 | 0.42 | cp | glycopyrrolate | Acetylcholine receptor antagonist | Acetylcholine receptor antagonist | |
| 727 | 0.42 | cp | cetirizine | Histamine receptor antagonist | Histamine receptor antagonist | |
| 728 | 0.42 | cp | carbamazepine | Carboxamide antiepileptic | Carboxamide antiepileptic | |
| 729 | 0.41 | cp | ibuprofen | Cyclooxygenase inhibitor | Cyclooxygenase inhibitor | |
| 730 | 0.41 | cp | GTP-14564 | FLT3 inhibitor | FLT3 inhibitor, Tyrosine kinase inhibitor | |
| 731 | 0.4 | cp | NCS-382 | GABA receptor antagonist | GABA receptor antagonist | |
| 732 | 0.39 | cp | semaxanib | VEGFR inhibitor | VEGFR inhibitor | |
| 733 | 0.39 | cp | doconexent | PPAR receptor agonist | PPAR receptor agonist | |
| 734 | 0.39 | cp | betaxolol | Adrenergic receptor antagonist | Adrenergic receptor antagonist | |
| 735 | 0.39 | cp | ricinine | Casein kinase inhibitor | Casein kinase inhibitor | |
| 736 | 0.39 | cp | bisoprolol | Adrenergic receptor antagonist | Adrenergic receptor antagonist | |
| 737 | 0.39 | cp | asiatic-acid | Apoptosis stimulant | Apoptosis stimulant | |
| 738 | 0.39 | cp | gingerol | Nitric oxide synthase inhibitor | Nitric oxide synthase inhibitor | |
| 739 | 0.39 | cp | testosterone | Androgen receptor agonist | Androgen receptor agonist | |
| 740 | 0.37 | cp | mitotane | Antineoplastic | Antineoplastic | |
| 741 | 0.35 | cp | ifenprodil | Adrenergic receptor antagonist | Adrenergic receptor antagonist, Glutamate receptor antagonist | |
| 742 | 0.35 | cp | ethylestrenol | Progesterone receptor agonist | Progesterone receptor agonist | |
| 743 | 0.35 | cp | lidoflazine | Calcium channel blocker | Calcium channel blocker | |
| 744 | 0.35 | cp | DUP-697 | Cyclooxygenase inhibitor | Cyclooxygenase inhibitor | |
| 745 | 0.35 | cp | ditolylguanidine | Sigma receptor agonist | Sigma receptor agonist | |
| 746 | 0.35 | cp | pifithrin-mu | HSP inhibitor | HSP inhibitor | |
| 747 | 0.35 | cp | paclitaxel | Tubulin inhibitor | Tubulin inhibitor | |
| 748 | 0.35 | cp | niridazole | Phosphofructokinase inhibitor | Phosphofructokinase inhibitor | |
| 749 | 0.35 | cp | mefenamic-acid | Cyclooxygenase inhibitor | Cyclooxygenase inhibitor | |
| 750 | 0.35 | cp | zimelidine | Serotonin reuptake inhibitor | Serotonin reuptake inhibitor | |
| 751 | 0.35 | cp | ethotoin | Hydantoin antiepileptic | Hydantoin antiepileptic | |
| 752 | 0.32 | cp | rilmenidine | Imidazoline receptor agonist | Imidazoline receptor agonist | |
| 753 | 0.32 | cp | EMF-bca1-60 | caspase inhibitor | caspase inhibitor | |
| 754 | 0.32 | cp | importazole | Importin-beta transport receptor inhibitor | Importin-beta transport receptor inhibitor | |
| 755 | 0.32 | cp | SKF-81297 | Dopamine receptor agonist | Dopamine receptor agonist | |
| 756 | 0.32 | cp | piperlongumine | Glutathione transferase inhibitor | Glutathione transferase inhibitor | |
| 757 | 0.32 | cp | ketorolac | Cyclooxygenase inhibitor | Cyclooxygenase inhibitor | |
| 758 | 0.32 | cp | CGS-15943 | Adenosine receptor antagonist | Adenosine receptor antagonist | |
| 759 | 0.32 | cp | bisacodyl | Laxative | Laxative |  |
| 760 | 0.28 | cp | olanzapine | Dopamine receptor antagonist | Dopamine receptor antagonist, Serotonin receptor antagonist | |
| 761 | 0.28 | cp | MY-5445 | Phosphodiesterase inhibitor | Phosphodiesterase inhibitor, Platelet aggregation inhibitor | |
| 762 | 0.28 | cp | JAK3-inhibitor-V | JAK inhibitor | JAK inhibitor | |
| 763 | 0.28 | cp | zoxazolamine | Myorelaxant | Myorelaxant | |
| 764 | 0.28 | cp | bucladesine | Adenosine receptor agonist | Adenosine receptor agonist, cAMP stimulant | |
| 765 | 0.28 | cp | nicardipine | Calcium channel blocker | Calcium channel blocker | |
| 766 | 0.28 | cp | procainamide | Sodium channel blocker | Sodium channel blocker | |
| 767 | 0.28 | cp | flurbiprofen | Cyclooxygenase inhibitor | Cyclooxygenase inhibitor | |
| 768 | 0.28 | cp | tacrine | Acetylcholinesterase inhibitor | Acetylcholinesterase inhibitor, Acetylcholine release stimulant, Butyrylcholinesterase inhibitor, Potassium channel antagonist | |
| 769 | 0.25 | cp | ormetoprim | Bacterial antifolate | Bacterial antifolate | |
| 770 | 0.25 | cp | carpindolol | Adrenergic receptor antagonist | Adrenergic receptor antagonist, Serotonin receptor antagonist | |
| 771 | 0.25 | cp | DCEBIO | Potassium channel activator | Potassium channel activator | |
| 772 | 0.25 | cp | o-3M3FBS | phospholipase activator | phospholipase activator | |
| 773 | 0.25 | cp | ZM-39923 | JAK inhibitor | JAK inhibitor | |
| 774 | 0.25 | cp | fipronil | GABA gated chloride channel blocker | GABA gated chloride channel blocker, Chloride channel blocker | |
| 775 | 0.25 | cp | bromhexine | Mucolytic agent | Mucolytic agent | |
| 776 | 0.25 | cp | risperidone | Dopamine receptor antagonist | Dopamine receptor antagonist, Serotonin receptor antagonist | |
| 777 | 0.25 | cp | raltitrexed | Thymidylate synthase inhibitor | Thymidylate synthase inhibitor | |
| 778 | 0.25 | cp | triclosan | Enoyl-[acyl-carrier-protein] reductase [NADH] inhibitor | Enoyl-[acyl-carrier-protein] reductase [NADH] inhibitor | |
| 779 | 0.21 | cp | artemether | Antimalarial | Antimalarial | |
| 780 | 0.21 | cp | JZL-184 | Monoacylglucerol lipase inhibitor | Monoacylglucerol lipase inhibitor | |
| 781 | 0.21 | cp | telenzepine | Acetylcholine receptor antagonist | Acetylcholine receptor antagonist | |
| 782 | 0.21 | cp | PD-102807 | Acetylcholine receptor antagonist | Acetylcholine receptor antagonist | |
| 783 | 0.21 | cp | carprofen | Cyclooxygenase inhibitor | Cyclooxygenase inhibitor | |
| 784 | 0.21 | cp | thiorphan | Membrane metalloendopeptidase inhibitor | Membrane metalloendopeptidase inhibitor | |
| 785 | 0.21 | cp | oxprenolol | Adrenergic receptor antagonist | Adrenergic receptor antagonist | |
| 786 | 0.21 | cp | DMBI | PDGFR receptor inhibitor | PDGFR receptor inhibitor, VEGFR inhibitor | |
| 787 | 0.21 | cp | amperozide | Dopamine receptor antagonist | Dopamine receptor antagonist | |
| 788 | 0.21 | cp | GDC-0941 | PI3K inhibitor | PI3K inhibitor | |
| 789 | 0.21 | cp | decitabine | DNA methyltransferase inhibitor | DNA methyltransferase inhibitor | |
| 790 | 0.21 | cp | rottlerin | MAP kinase inhibitor | MAP kinase inhibitor, Protein kinase inhibitor | |
| 791 | 0.19 | cp | vincamine | Adrenergic receptor antagonist | Adrenergic receptor antagonist | |
| 792 | 0.18 | cp | cerivastatin | HMGCR inhibitor | HMGCR inhibitor | |
| 793 | 0.18 | cp | methylnorlichexanthone | Aurora kinase inhibitor | Aurora kinase inhibitor, PIM inhibitor, VEGFR inhibitor | |
| 794 | 0.18 | cp | pivmecillinam | Bacterial cell wall synthesis inhibitor | Bacterial cell wall synthesis inhibitor | |
| 795 | 0.18 | cp | escitalopram | Selective serotonin reuptake inhibitor (SSRI) | Selective serotonin reuptake inhibitor (SSRI) | |
| 796 | 0.18 | cp | chlortalidone | Carbonic anhydrase inhibitor | Carbonic anhydrase inhibitor | |
| 797 | 0.15 | cp | benzoxiquine | Anti-infective | Anti-infective | |
| 798 | 0.14 | cp | methotrexate | Dihydrofolate reductase inhibitor | Dihydrofolate reductase inhibitor | |
| 799 | 0.14 | cp | diphenoxylate | Opioid receptor agonist | Opioid receptor agonist | |
| 800 | 0.14 | cp | danazol | Estrogen receptor antagonist | Estrogen receptor antagonist, Progesterone receptor agonist | |
| 801 | 0.14 | cp | paroxetine | Selective serotonin reuptake inhibitor (SSRI) | Selective serotonin reuptake inhibitor (SSRI) | |
| 802 | 0.14 | cp | fluconazole | Sterol demethylase inhibitor | Sterol demethylase inhibitor | |
| 803 | 0.14 | cp | desipramine | Tricyclic antidepressant | Tricyclic antidepressant | |
| 804 | 0.14 | cp | orciprenaline | Adrenergic receptor agonist | Adrenergic receptor agonist | |
| 805 | 0.14 | cp | imatinib | BCR-ABL kinase inhibitor | BCR-ABL kinase inhibitor, KIT inhibitor, PDGFR receptor inhibitor | |
| 806 | 0.14 | cp | valdecoxib | Cyclooxygenase inhibitor | Cyclooxygenase inhibitor | |
| 807 | 0.11 | cp | itopride | Dopamine receptor antagonist | Dopamine receptor antagonist | |
| 808 | 0.11 | cp | amlodipine | Calcium channel blocker | Calcium channel blocker | |
| 809 | 0.11 | cp | diphenhydramine | Histamine receptor antagonist | Histamine receptor antagonist | |
| 810 | 0.11 | cp | atomoxetine | Norepinephrine transporter inhibitor | Norepinephrine transporter inhibitor | |
| 811 | 0.11 | cp | pentoxifylline | Phosphodiesterase inhibitor | Phosphodiesterase inhibitor | |
| 812 | 0.11 | cp | rosuvastatin | HMGCR inhibitor | HMGCR inhibitor | |
| 813 | 0.11 | cp | amodiaquine | Histamine receptor agonist | Histamine receptor agonist | |
| 814 | 0.11 | cp | proxymetacaine | Sodium channel blocker | Sodium channel blocker | |
| 815 | 0.11 | cp | SDM25N | Opioid receptor antagonist | Opioid receptor antagonist | |
| 816 | 0.11 | cp | RU-24969 | Serotonin receptor agonist | Serotonin receptor agonist | |
| 817 | 0.1 | cp | TGX-221 | PI3K inhibitor | PI3K inhibitor | |
| 818 | 0.09 | cp | talampicillin | Bacterial cell wall synthesis inhibitor | Bacterial cell wall synthesis inhibitor | |
| 819 | 0.09 | cp | selamectin | Nematocide | Nematocide | |
| 820 | 0.07 | cp | mesalazine | Cyclooxygenase inhibitor | Cyclooxygenase inhibitor, Lipoxygenase inhibitor, Prostanoid receptor antagonist | |
| 821 | 0.07 | cp | SID-26681509 | Cathepsin inhibitor | Cathepsin inhibitor | |
| 822 | 0.07 | cp | propafenone | Antiarrhythmic | Antiarrhythmic | |
| 823 | 0.07 | cp | nobiletin | MEK inhibitor | MEK inhibitor | |
| 824 | 0.07 | cp | GW-405833 | Cannabinoid receptor agonist | Cannabinoid receptor agonist | |
| 825 | 0.07 | cp | GBR-12935 | Dopamine uptake inhibitor | Dopamine uptake inhibitor | |
| 826 | 0.07 | cp | thiethylperazine | Dopamine receptor antagonist | Dopamine receptor antagonist | |
| 827 | 0.07 | cp | sertraline | Serotonin receptor antagonist | Serotonin receptor antagonist | |
| 828 | 0.07 | cp | butylparaben | DNA synthesis inhibitor | DNA synthesis inhibitor | |
| 829 | 0.07 | cp | niclosamide | DNA replication inhibitor | DNA replication inhibitor, STAT inhibitor | |
| 830 | 0.06 | cp | dosulepin | Norepinephrine reuptake inhibitor | Norepinephrine reuptake inhibitor, Tricyclic antidepressant, Serotonin reuptake inhibitor | |
| 831 | 0.05 | cp | clonidine | Adrenergic receptor agonist | Adrenergic receptor agonist | |
| 832 | 0.04 | cp | sibutramine | Serotonin reuptake inhibitor | Serotonin reuptake inhibitor | |
| 833 | 0.04 | cp | meptazinol | Opioid receptor agonist | Opioid receptor agonist | |
| 834 | 0.04 | cp | fluperlapine | Serotonin receptor antagonist | Serotonin receptor antagonist | |
| 835 | 0.04 | cp | thiotepa | Cytochrome P450 inhibitor | Cytochrome P450 inhibitor | |
| 836 | 0.04 | cp | ketotifen | Histamine receptor agonist | Histamine receptor agonist, Histamine receptor ligand, Leukotriene receptor antagonist, Phosphodiesterase inhibitor | |
| 837 | 0.04 | cp | cilostazol | Phosphodiesterase inhibitor | Phosphodiesterase inhibitor | |
| 838 | 0.04 | cp | caffeine | Adenosine receptor antagonist | Adenosine receptor antagonist, Diuretic, Phosphodiesterase inhibitor | |
| 839 | 0.04 | cp | vemurafenib | RAF inhibitor | RAF inhibitor | |
| 840 | 0.04 | cp | fluphenazine | Dopamine receptor antagonist | Dopamine receptor antagonist | |
| 841 | 0.04 | cp | nicotine | Acetylcholine receptor agonist | Acetylcholine receptor agonist | |
| 842 | 0.04 | cp | SA-792541 | CDC inhibitor | CDC inhibitor | |
| 843 | 0.04 | cp | triflupromazine | Dopamine receptor antagonist | Dopamine receptor antagonist | |
| 844 | 0.03 | cp | Merck60 | HDAC inhibitor | HDAC inhibitor | |
| 845 | 0.03 | cp | M-3M3FBS | phospholipase activator | phospholipase activator | |
| 846 | 0.03 | cp | zolmitriptan | Serotonin receptor agonist | Serotonin receptor agonist | |
| 847 | 0.03 | cp | saquinavir | HIV protease inhibitor | HIV protease inhibitor | |
| 848 | 0.03 | cp | lovastatin | HMGCR inhibitor | HMGCR inhibitor | |
| 849 | 0.03 | cp | dextromethorphan | Glutamate receptor antagonist | Glutamate receptor antagonist, Sigma receptor agonist | |
| 850 | 0.03 | cp | crotamiton | Antipruritic | Antipruritic | |
| 851 | 0.03 | cp | BAY-11-7821 | NFkB pathway inhibitor | NFkB pathway inhibitor | |
| 852 | 0.03 | cp | AM-580 | Retinoid receptor agonist | Retinoid receptor agonist | |
| 853 | 0.03 | cp | penitrem-a | Potassium channel blocker | Potassium channel blocker | |
| 854 | 0.03 | cp | droperidol | Dopamine receptor antagonist | Dopamine receptor antagonist | |
| 855 | 0.03 | cp | loperamide | Opioid receptor agonist | Opioid receptor agonist | |
| 856 | 0.03 | cp | terbinafine | Fungal squalene epoxidase inhibitor | Fungal squalene epoxidase inhibitor | |
| 857 | 0.02 | cp | PK-11195 | Benzodiazepine receptor antagonist | Benzodiazepine receptor antagonist | |
| 858 | 0.02 | cp | bendroflumethiazide | Sodium/potassium/chloride transporter inhibitor | Sodium/potassium/chloride transporter inhibitor | |
| 859 | 0.02 | cp | ZM-447439 | Aurora kinase inhibitor | Aurora kinase inhibitor | |
| 860 | 0.01 | cp | clomifene | Estrogen receptor antagonist | Estrogen receptor antagonist | |
| 861 | 0.01 | cp | clozapine | Dopamine receptor antagonist | Dopamine receptor antagonist, Serotonin receptor antagonist | |
| 862 | 0.01 | cp | celecoxib | Cyclooxygenase inhibitor | Cyclooxygenase inhibitor | |
| 863 | 0 | cp | alisertib | Aurora kinase inhibitor | Aurora kinase inhibitor | |
| 864 | 0 | cp | toremifene | Estrogen receptor antagonist | Estrogen receptor antagonist, Selective estrogen receptor modulator (SERM) | |
| 865 | 0 | cp | MK-5108 | Aurora kinase inhibitor | Aurora kinase inhibitor | |
| 866 | 0 | cp | AT-9283 | JAK inhibitor | JAK inhibitor, Aurora kinase inhibitor, ABL inhibitor, BCR-ABL kinase inhibitor, FLT3 inhibitor, Mitotic inhibitor, Protein kinase inhibitor | |
| 867 | 0 | cp | avrainvillamide-analog-4 | nucleophosmin inhibitor | nucleophosmin inhibitor | |
| 868 | 0 | cp | ENMD-2076 | FLT3 inhibitor | FLT3 inhibitor, VEGFR inhibitor, Aurora kinase inhibitor | |
| 869 | 0 | cp | avrainvillamide-analog-5 | nucleophosmin inhibitor | nucleophosmin inhibitor | |
| 870 | 0 | cp | benzohydroxamic-acid | Antifungal | Antifungal | |
| 871 | 0 | cp | theophylline | Adenosine receptor antagonist | Adenosine receptor antagonist | |
| 872 | 0 | cp | tetracycline | Bacterial 30S ribosomal subunit inhibitor | Bacterial 30S ribosomal subunit inhibitor | |
| 873 | 0 | cp | pyrazinamide | Fatty acid synthase inhibitor | Fatty acid synthase inhibitor | |
| 874 | 0 | cp | methimazole | Antithyroid | Antithyroid | |
| 875 | 0 | cp | chloramphenicol | Protein synthesis inhibitor | Protein synthesis inhibitor, Bacterial 50S ribosomal subunit inhibitor | |
| 876 | 0 | cp | captopril | ACE inhibitor | ACE inhibitor | |
| 877 | 0 | cp | cobalt(II)-chloride | HSP inducer | HSP inducer | |
| 878 | 0 | cp | dimercaptosuccinic-acid | Chelating agent | Chelating agent | |
| 879 | 0 | cp | entacapone | Catechol O methyltransferase inhibitor | Catechol O methyltransferase inhibitor | |
| 880 | 0 | cp | MPEP | Glutamate receptor antagonist | Glutamate receptor antagonist | |
| 881 | 0 | cp | 1-phenylbiguanide | Serotonin receptor agonist | Serotonin receptor agonist | |
| 882 | 0 | cp | BRD-A80383043 | Glutamate receptor agonist | Glutamate receptor agonist, Glutamate receptor antagonist | |
| 883 | 0 | cp | ascorbyl-palmitate | antioxidant | antioxidant | |
| 884 | 0 | cp | bicuculline | GABA receptor antagonist | GABA receptor antagonist | |
| 885 | 0 | cp | teicoplanin | Bacterial cell wall synthesis inhibitor | Bacterial cell wall synthesis inhibitor | |
| 886 | 0 | cp | oleoylethanolamide | Cannabinoid receptor agonist | Cannabinoid receptor agonist, Glucose dependent insulinotropic receptor agonist, Potassium channel blocker, PPAR receptor agonist | |
| 887 | 0 | cp | SB-525334 | TGF beta receptor inhibitor | TGF beta receptor inhibitor | |
| 888 | 0 | cp | elvitegravir | HIV integrase inhibitor | HIV integrase inhibitor | |
| 889 | 0 | cp | iloperidone | Dopamine receptor antagonist | Dopamine receptor antagonist, Serotonin receptor antagonist | |
| 890 | 0 | cp | AS-703026 | MEK inhibitor | MEK inhibitor | |
| 891 | 0 | cp | VX-745 | p38 MAPK inhibitor | p38 MAPK inhibitor | |
| 892 | 0 | cp | PIK-75 | DNA protein kinase inhibitor | DNA protein kinase inhibitor, PI3K inhibitor | |
| 893 | 0 | cp | brivanib | FGFR inhibitor | FGFR inhibitor, VEGFR inhibitor | |
| 894 | 0 | cp | bosutinib | ABL inhibitor | ABL inhibitor, BCR-ABL kinase inhibitor, SRC inhibitor | |
| 895 | 0 | cp | lopinavir | HIV protease inhibitor | HIV protease inhibitor | |
| 896 | 0 | cp | K3644 | Kinesin-like spindle protein inhibitor | Kinesin-like spindle protein inhibitor | |
| 897 | 0 | cp | crizotinib | ALK inhibitor | ALK inhibitor | |
| 898 | 0 | cp | SB-239063 | p38 MAPK inhibitor | p38 MAPK inhibitor | |
| 899 | 0 | cp | AZ-628 | RAF inhibitor | RAF inhibitor | |
| 900 | 0 | cp | PHA-793887 | CDK inhibitor | CDK inhibitor | |
| 901 | 0 | cp | BMS-345541 | IKK inhibitor | IKK inhibitor | |
| 902 | 0 | cp | PF-562271 | Focal adhesion kinase inhibitor | Focal adhesion kinase inhibitor | |
| 903 | 0 | cp | tetradecylthioacetic-acid | Lipid peroxidase inhibitor | Lipid peroxidase inhibitor | |
| 904 | 0 | cp | GR-235 | Estrogen receptor agonist | Estrogen receptor agonist, FXR antagonist, Progesterone receptor agonist | |
| 905 | 0 | cp | 4-hydroxyretinoic-acid | Retinoid receptor binder | Retinoid receptor binder | |
| 906 | 0 | cp | flupirtine | Glutamate receptor antagonist | Glutamate receptor antagonist | |
| 907 | 0 | cp | lavendustin-c | EGFR inhibitor | EGFR inhibitor | |
| 908 | 0 | cp | tyrphostin-51 | EGFR inhibitor | EGFR inhibitor | |
| 909 | 0 | cp | sphingosine | Ceramidase inhibitor | Ceramidase inhibitor | |
| 910 | 0 | cp | etomoxir | Carnitine palmitoyltransferase inhibitor | Carnitine palmitoyltransferase inhibitor | |
| 911 | 0 | cp | TCPOBOP | CAR agonist | CAR agonist | |
| 912 | 0 | cp | chenodeoxycholic-acid | 11-beta-HSD1 inhibitor | 11-beta-HSD1 inhibitor, FXR agonist | |
| 913 | 0 | cp | cholic-acid | Bile acid | Bile acid | |
| 914 | 0 | cp | retinyl | vitamin analog | vitamin analog | |
| 915 | 0 | cp | guggulsterone | Cholesterol inhibitor | Cholesterol inhibitor, Estrogen receptor agonist, FXR antagonist, IKK inhibitor, Pregnane X receptor agonist, Progesterone receptor agonist | |
| 916 | 0 | cp | celastrol | Anti-inflammatory | Anti-inflammatory, Antioxidant, NFkB pathway inhibitor, HSP inhibitor, Topoisomerase inhibitor | |
| 917 | 0 | cp | withaferin-a | IKK inhibitor | IKK inhibitor | |
| 918 | 0 | cp | CAY-10585 | HIF modulator | HIF modulator | |
| 919 | 0 | cp | somatostatin | Somatostatin receptor agonist | Somatostatin receptor agonist | |
| 920 | 0 | cp | QL-XII-47 | BTK inhibitor | BTK inhibitor, Cytoplasmic tyrosine protein kinase BMX inhibitor | |
| 921 | 0 | cp | CG-930 | JNK inhibitor | JNK inhibitor | |
| 922 | 0 | cp | torin-2 | MTOR inhibitor | MTOR inhibitor | |
| 923 | 0 | cp | A-443644 | AKT inhibitor | AKT inhibitor | |
| 924 | 0 | cp | AS-601245 | JNK inhibitor | JNK inhibitor | |
| 925 | 0 | cp | zeranol | Estrogen receptor agonist | Estrogen receptor agonist | |
| 926 | 0 | cp | oxcarbazepine | Sodium channel blocker | Sodium channel blocker | |
| 927 | 0 | cp | tremulacin | Lipoxygenase inhibitor | Lipoxygenase inhibitor | |
| 928 | 0 | cp | tosufloxacin | Bacterial DNA gyrase inhibitor | Bacterial DNA gyrase inhibitor | |
| 929 | 0 | cp | topiramate | Carbonic anhydrase inhibitor | Carbonic anhydrase inhibitor, Glutamate receptor antagonist, Kainate receptor antagonist | |
| 930 | 0 | cp | tinidazole | Antiprotozoal | Antiprotozoal | |
| 931 | 0 | cp | ropivacaine | Sodium channel blocker | Sodium channel blocker | |
| 932 | 0 | cp | rolitetracycline | Bacterial 30S ribosomal subunit inhibitor | Bacterial 30S ribosomal subunit inhibitor | |
| 933 | 0 | cp | ritonavir | HIV protease inhibitor | HIV protease inhibitor | |
| 934 | 0 | cp | rimcazole | Sigma receptor antagonist | Sigma receptor antagonist | |
| 935 | 0 | cp | reichstein | Androgen receptor antagonist | Androgen receptor antagonist | |
| 936 | 0 | cp | pancuronium | Acetylcholine receptor antagonist | Acetylcholine receptor antagonist | |
| 937 | 0 | cp | oxyphenonium | Cholinergic receptor antagonist | Cholinergic receptor antagonist | |
| 938 | 0 | cp | oligomycin-c | ATPase inhibitor | ATPase inhibitor, ATP synthase inhibitor | |
| 939 | 0 | cp | milnacipran | Serotonin reuptake inhibitor | Serotonin reuptake inhibitor | |
| 940 | 0 | cp | metronidazole | DNA inhibitor | DNA inhibitor, Antiprotozoal | |
| 941 | 0 | cp | methandriol | Androgenic steroid | Androgenic steroid | |
| 942 | 0 | cp | mecillinam | Bacterial cell wall synthesis inhibitor | Bacterial cell wall synthesis inhibitor | |
| 943 | 0 | cp | lorazepam | Benzodiazepine receptor agonist | Benzodiazepine receptor agonist | |
| 944 | 0 | cp | targinine | Nitric oxide synthase inhibitor | Nitric oxide synthase inhibitor | |
| 945 | 0 | cp | pitavastatin | HMGCR inhibitor | HMGCR inhibitor | |
| 946 | 0 | cp | ipriflavone | Bone resorption inhibitor | Bone resorption inhibitor | |
| 947 | 0 | cp | galantamine | Acetylcholinesterase inhibitor | Acetylcholinesterase inhibitor | |
| 948 | 0 | cp | dolasetron | Serotonin receptor antagonist | Serotonin receptor antagonist | |
| 949 | 0 | cp | desoximetasone | Glucocorticoid receptor agonist | Glucocorticoid receptor agonist | |
| 950 | 0 | cp | clotrimazole | Cytochrome P450 inhibitor | Cytochrome P450 inhibitor, Imidazoline receptor ligand | |
| 951 | 0 | cp | chlordiazepoxide | Benzodiazepine receptor agonist | Benzodiazepine receptor agonist | |
| 952 | 0 | cp | cefixime | Bacterial cell wall synthesis inhibitor | Bacterial cell wall synthesis inhibitor | |
| 953 | 0 | cp | cefdinir | Bacterial cell wall synthesis inhibitor | Bacterial cell wall synthesis inhibitor | |
| 954 | 0 | cp | benproperine | Antitussive | Antitussive | |
| 955 | 0 | cp | beclometasone | Glucocorticoid receptor agonist | Glucocorticoid receptor agonist | |
| 956 | 0 | cp | actarit | Interleukin receptor agonist | Interleukin receptor agonist | |
| 957 | 0 | cp | topotecan | Topoisomerase inhibitor | Topoisomerase inhibitor | |
| 958 | 0 | cp | epothilone | Microtubule inhibitor | Microtubule inhibitor | |
| 959 | 0 | cp | JNJ-7706621 | CDK inhibitor | CDK inhibitor | |
| 960 | 0 | cp | barasertib | Aurora kinase inhibitor | Aurora kinase inhibitor | |
| 961 | 0 | cp | YM-155 | Survivin inhibitor | Survivin inhibitor | |
| 962 | 0 | cp | tandutinib | FLT3 inhibitor | FLT3 inhibitor, KIT inhibitor, PDGFR receptor inhibitor | |
| 963 | 0 | cp | lenalidomide | Antineoplastic | Antineoplastic | |
| 964 | 0 | cp | lapatinib | EGFR inhibitor | EGFR inhibitor, ErbB2 inhibitor | |
| 965 | 0 | cp | varenicline | Acetylcholine receptor agonist | Acetylcholine receptor agonist | |
| 966 | 0 | cp | OM-137 | Aurora kinase inhibitor | Aurora kinase inhibitor | |
| 967 | 0 | cp | doxercalciferol | Vitamin D receptor agonist | Vitamin D receptor agonist | |
| 968 | 0 | cp | desloratadine | Histamine receptor antagonist | Histamine receptor antagonist | |
| 969 | 0 | cp | CAY-10415 | Insulin sensitizer | Insulin sensitizer | |
| 970 | 0 | cp | mofezolac | Cyclooxygenase inhibitor | Cyclooxygenase inhibitor | |
| 971 | 0 | cp | sirolimus | MTOR inhibitor | MTOR inhibitor | |
| 972 | 0 | cp | larixinic-acid | Compound that interacts with metal centers | Compound that interacts with metal centers | |
| 973 | 0 | cp | treprostinil | Prostacyclin analog | Prostacyclin analog | |
| 974 | 0 | cp | CAY-10578 | Casein kinase inhibitor | Casein kinase inhibitor | |
| 975 | 0 | cp | HU-211 | Glutamate receptor antagonist | Glutamate receptor antagonist | |
| 976 | 0 | cp | 1-benzylimidazole | Thromboxane synthase inhibitor | Thromboxane synthase inhibitor | |
| 977 | 0 | cp | HG-5-113-01 | Protein kinase inhibitor | Protein kinase inhibitor | |
| 978 | 0 | cp | KUC103904N | -666 | -666 |  |
| 979 | 0 | cp | KUC103898N | -666 | -666 |  |
| 980 | 0 | cp | cyproheptadine | Histamine receptor antagonist | Histamine receptor antagonist | |
| 981 | 0 | cp | terazosin | Adrenergic receptor antagonist | Adrenergic receptor antagonist | |
| 982 | 0 | cp | SB-205607 | Delta 1 opioid receptor agonist | Delta 1 opioid receptor agonist | |
| 983 | 0 | cp | methylprednisolone | Glucocorticoid receptor agonist | Glucocorticoid receptor agonist | |
| 984 | 0 | cp | lamivudine | Nucleoside reverse transcriptase inhibitor | Nucleoside reverse transcriptase inhibitor | |
| 985 | 0 | cp | ethambutol | Bacterial cell wall synthesis inhibitor | Bacterial cell wall synthesis inhibitor | |
| 986 | 0 | cp | VU-0420363-1 | SARS coronavirus 3C-like protease inhibitor | SARS coronavirus 3C-like protease inhibitor | |
| 987 | 0 | cp | VU-0413807-2 | Calcium channel blocker | Calcium channel blocker | |
| 988 | 0 | cp | VU-0400071-3 | Glutamate receptor modulator | Glutamate receptor modulator | |
| 989 | 0 | cp | myricetin | Androgen receptor agonist | Androgen receptor agonist, Cytochrome P450 inhibitor | |
| 990 | 0 | cp | CAM-9-026 | Membrane metalloendopeptidase inhibitor | Membrane metalloendopeptidase inhibitor | |
| 991 | 0 | cp | 2-(biphenyl-4-ylsulfonamido)pentanedioic-acid | Matrix metalloprotease inhibitor | Matrix metalloprotease inhibitor | |
| 992 | 0 | cp | KU-C103871 | GSP agonist | GSP agonist | |
| 993 | 0 | cp | LDN-193189 | Serine/threonine kinase inhibitor | Serine/threonine kinase inhibitor | |
| 994 | 0 | cp | temsirolimus | MTOR inhibitor | MTOR inhibitor | |
| 995 | 0 | cp | rilmenidine | Adrenergic receptor agonist | Adrenergic receptor agonist, Imidazoline receptor agonist | |
| 996 | 0 | cp | auranofin | NFkB pathway inhibitor | NFkB pathway inhibitor | |
| 997 | 0 | cp | SU-11652 | Tyrosine kinase inhibitor | Tyrosine kinase inhibitor | |
| 998 | 0 | cp | PT-630 | Dipeptidyl peptidase inhibitor | Dipeptidyl peptidase inhibitor | |
| 999 | 0 | cp | prima-1-met | thioredoxin inhibitor | thioredoxin inhibitor | |
| 1000 | 0 | cp | narciclasine | Coflilin signaling pathway activator | Coflilin signaling pathway activator, LIM kinase activator, Rho associated kinase activator | |
| 1001 | 0 | cp | BMS-299897 | Gamma secretase inhibitor | Gamma secretase inhibitor | |
| 1002 | 0 | cp | AKT-inhibitor-IV | AKT inhibitor | AKT inhibitor | |
| 1003 | 0 | cp | AG-14361 | PARP inhibitor | PARP inhibitor | |
| 1004 | 0 | cp | 2',5'-dideoxyadenosine | Adenylyl cyclase inhibitor | Adenylyl cyclase inhibitor | |
| 1005 | 0 | cp | 9-methyl-5H-6-thia-4,5-diaza-chrysene-6,6-dioxide | NFkB pathway inhibitor | NFkB pathway inhibitor | |
| 1006 | 0 | cp | SCH-79797 | Proteasome inhibitor | Proteasome inhibitor | |
| 1007 | 0 | cp | SN-38 | Topoisomerase inhibitor | Topoisomerase inhibitor | |
| 1008 | 0 | cp | JTC-801 | Opioid receptor antagonist | Opioid receptor antagonist | |
| 1009 | 0 | cp | siguazodan | Phosphodiesterase inhibitor | Phosphodiesterase inhibitor | |
| 1010 | 0 | cp | m-chlorophenylbiguanide | Serotonin receptor agonist | Serotonin receptor agonist | |
| 1011 | 0 | cp | coumestrol | Estrogen receptor agonist | Estrogen receptor agonist | |
| 1012 | 0 | cp | cinobufagin | ATPase inhibitor | ATPase inhibitor | |
| 1013 | 0 | cp | ZM-323881 | VEGFR inhibitor | VEGFR inhibitor | |
| 1014 | 0 | cp | ZD-2079 | Adrenergic receptor agonist | Adrenergic receptor agonist | |
| 1015 | 0 | cp | YM-976 | Phosphodiesterase inhibitor | Phosphodiesterase inhibitor | |
| 1016 | 0 | cp | azasetron | Serotonin receptor antagonist | Serotonin receptor antagonist | |
| 1017 | 0 | cp | xaliproden | Serotonin receptor agonist | Serotonin receptor agonist | |
| 1018 | 0 | cp | WAY-213613 | Glutamate inhibitor | Glutamate inhibitor | |
| 1019 | 0 | cp | tropanyl-3,5-dimethylbenzoate | Serotonin receptor antagonist | Serotonin receptor antagonist | |
| 1020 | 0 | cp | trans-7-hydroxy-pipat | Dopamine receptor ligand | Dopamine receptor ligand | |
| 1021 | 0 | cp | telmisartan | Angiotensin receptor antagonist | Angiotensin receptor antagonist | |
| 1022 | 0 | cp | TCB2 | Serotonin receptor agonist | Serotonin receptor agonist | |
| 1023 | 0 | cp | T-0901317 | LXR agonist | LXR agonist, ABC transporter expression enhancer, ROR inverse agonist | |
| 1024 | 0 | cp | strophanthidin | ATPase inhibitor | ATPase inhibitor | |
| 1025 | 0 | cp | skimmianine | Acetylcholinesterase inhibitor | Acetylcholinesterase inhibitor, Furoquinoline alkaloid | |
| 1026 | 0 | cp | securinine | GABA receptor antagonist | GABA receptor antagonist, TP53 activator | |
| 1027 | 0 | cp | scopolamine | Acetylcholine receptor antagonist | Acetylcholine receptor antagonist | |
| 1028 | 0 | cp | saclofen | GABA receptor antagonist | GABA receptor antagonist | |
| 1029 | 0 | cp | SU-1498 | VEGFR inhibitor | VEGFR inhibitor | |
| 1030 | 0 | cp | SU-4312 | PDGFR receptor inhibitor | PDGFR receptor inhibitor, Tyrosine kinase inhibitor, VEGFR inhibitor | |
| 1031 | 0 | cp | SR-59230A | Adrenergic receptor antagonist | Adrenergic receptor antagonist | |
| 1032 | 0 | cp | SR-142948 | Neurotensin receptor antagonist | Neurotensin receptor antagonist | |
| 1033 | 0 | cp | pyrazolanthrone | JNK inhibitor | JNK inhibitor | |
| 1034 | 0 | cp | skatole | Thrombin inhibitor | Thrombin inhibitor | |
| 1035 | 0 | cp | sinensetin | Cyclooxygenase inhibitor | Cyclooxygenase inhibitor | |
| 1036 | 0 | cp | SEW-2871 | Lysophospholipid receptor agonist | Lysophospholipid receptor agonist | |
| 1037 | 0 | cp | SDZ-WAG-994 | Adenosine receptor agonist | Adenosine receptor agonist | |
| 1038 | 0 | cp | SDZ-NKT-343 | Tachykinin antagonist | Tachykinin antagonist | |
| 1039 | 0 | cp | SDZ-205-557 | Serotonin receptor antagonist | Serotonin receptor antagonist | |
| 1040 | 0 | cp | SCH-58261 | Adenosine receptor antagonist | Adenosine receptor antagonist | |
| 1041 | 0 | cp | ecopipam | Dopamine receptor antagonist | Dopamine receptor antagonist | |
| 1042 | 0 | cp | SB-269970 | Serotonin receptor antagonist | Serotonin receptor antagonist | |
| 1043 | 0 | cp | SB-200646 | Serotonin receptor antagonist | Serotonin receptor antagonist | |
| 1044 | 0 | cp | salvinorin-a | Opioid receptor agonist | Opioid receptor agonist | |
| 1045 | 0 | cp | ryuvidine | Histone lysine methyltransferase inhibitor | Histone lysine methyltransferase inhibitor | |
| 1046 | 0 | cp | RO-19-4605 | GABA benzodiazepine site receptor inverse agonist | GABA benzodiazepine site receptor inverse agonist | |
| 1047 | 0 | cp | RO-04-5595 | Glutamate receptor antagonist | Glutamate receptor antagonist | |
| 1048 | 0 | cp | RS-17053 | Adrenergic receptor antagonist | Adrenergic receptor antagonist | |
| 1049 | 0 | cp | rotenonic-acid | Retinoid receptor antagonist | Retinoid receptor antagonist | |
| 1050 | 0 | cp | ritodrine | Adrenergic receptor agonist | Adrenergic receptor agonist | |
| 1051 | 0 | cp | RHC-80267 | Triacylglycerol lipase inhibitor | Triacylglycerol lipase inhibitor | |
| 1052 | 0 | cp | reserpine | Vesicular monoamine transporter inhibitor | Vesicular monoamine transporter inhibitor | |
| 1053 | 0 | cp | ranitidine | Histamine receptor antagonist | Histamine receptor antagonist | |
| 1054 | 0 | cp | R-96544 | Serotonin receptor antagonist | Serotonin receptor antagonist | |
| 1055 | 0 | cp | pyrrolidine-dithiocarbamate | NFkB pathway inhibitor | NFkB pathway inhibitor | |
| 1056 | 0 | cp | pirfenidone | TGF beta receptor inhibitor | TGF beta receptor inhibitor | |
| 1057 | 0 | cp | picrotoxin | GABA receptor antagonist | GABA receptor antagonist | |
| 1058 | 0 | cp | piceatannol | SYK inhibitor | SYK inhibitor | |
| 1059 | 0 | cp | phenanthridone | PARP inhibitor | PARP inhibitor | |
| 1060 | 0 | cp | psoromic-acid | Ras GTPase inhibitor | Ras GTPase inhibitor | |
| 1061 | 0 | cp | PSB-11 | Adenosine receptor antagonist | Adenosine receptor antagonist | |
| 1062 | 0 | cp | beta-CCP | Indoleamine 2,3-dioxygenase inhibitor | Indoleamine 2,3-dioxygenase inhibitor | |
| 1063 | 0 | cp | propofol | GABA receptor agonist | GABA receptor agonist | |
| 1064 | 0 | cp | PQ-401 | IGF-1 inhibitor | IGF-1 inhibitor, IGF-1R inhibitor | |
| 1065 | 0 | cp | PP-2 | SRC inhibitor | SRC inhibitor | |
| 1066 | 0 | cp | PNU-282987 | Cholinergic receptor agonist | Cholinergic receptor agonist | |
| 1067 | 0 | cp | PNU-120596 | Acetylcholine receptor agonist | Acetylcholine receptor agonist | |
| 1068 | 0 | cp | D-64406 | PDGFR receptor inhibitor | PDGFR receptor inhibitor | |
| 1069 | 0 | cp | SA-792987 | PKC inhibitor | PKC inhibitor | |
| 1070 | 0 | cp | parachlorophenol | Anti-infective | Anti-infective | |
| 1071 | 0 | cp | oxymetazoline | Adrenergic receptor agonist | Adrenergic receptor agonist | |
| 1072 | 0 | cp | oxindole-I | VEGFR inhibitor | VEGFR inhibitor | |
| 1073 | 0 | cp | oxybenzone | Lipase inhibitor | Lipase inhibitor | |
| 1074 | 0 | cp | ODQ | Guanylyl cyclase inhibitor | Guanylyl cyclase inhibitor | |
| 1075 | 0 | cp | O-1918 | Cannabinoid receptor antagonist | Cannabinoid receptor antagonist | |
| 1076 | 0 | cp | nomegestrol | Progesterone receptor agonist | Progesterone receptor agonist | |
| 1077 | 0 | cp | nimesulide | Cyclooxygenase inhibitor | Cyclooxygenase inhibitor | |
| 1078 | 0 | cp | nemonapride | Dopamine receptor antagonist | Dopamine receptor antagonist | |
| 1079 | 0 | cp | naproxen | Cyclooxygenase inhibitor | Cyclooxygenase inhibitor | |
| 1080 | 0 | cp | naltriben | Opioid receptor antagonist | Opioid receptor antagonist | |
| 1081 | 0 | cp | naloxone | Opioid receptor antagonist | Opioid receptor antagonist | |
| 1082 | 0 | cp | NSC-3852 | HDAC inhibitor | HDAC inhibitor | |
| 1083 | 0 | cp | noreleagnine | Monoamine oxidase inhibitor | Monoamine oxidase inhibitor, Serotonin receptor agonist | |
| 1084 | 0 | cp | nonoxynol-9 | Membrane integrity inhibitor | Membrane integrity inhibitor | |
| 1085 | 0 | cp | NNC-711 | GAT inhibitor | GAT inhibitor | |
| 1086 | 0 | cp | NNC-05-2090 | GAT inhibitor | GAT inhibitor, GABA uptake inhibitor | |
| 1087 | 0 | cp | NGB-2904 | Dopamine receptor antagonist | Dopamine receptor antagonist | |
| 1088 | 0 | cp | NBI-27914 | CRF receptor antagonist | CRF receptor antagonist | |
| 1089 | 0 | cp | NAN-190 | Serotonin receptor agonist | Serotonin receptor agonist | |
| 1090 | 0 | cp | moexipril | ACE inhibitor | ACE inhibitor | |
| 1091 | 0 | cp | mianserin | Serotonin receptor antagonist | Serotonin receptor antagonist | |
| 1092 | 0 | cp | methantheline | Acetylcholine receptor antagonist | Acetylcholine receptor antagonist | |
| 1093 | 0 | cp | meclocycline | Bacterial 30S ribosomal subunit inhibitor | Bacterial 30S ribosomal subunit inhibitor, Bacterial 50S ribosomal subunit inhibitor | |
| 1094 | 0 | cp | MRS-1845 | Calcium channel blocker | Calcium channel blocker | |
| 1095 | 0 | cp | MNITMT | Lymphocyte inhibitor | Lymphocyte inhibitor | |
| 1096 | 0 | cp | ML-3163 | p38 MAPK inhibitor | p38 MAPK inhibitor | |
| 1097 | 0 | cp | metoclopramide | Dopamine receptor antagonist | Dopamine receptor antagonist, Serotonin receptor antagonist | |
| 1098 | 0 | cp | methoprene-acid | Retinoid receptor agonist | Retinoid receptor agonist | |
| 1099 | 0 | cp | metaxalone | Muscle relaxant | Muscle relaxant | |
| 1100 | 0 | cp | l-erythro-MAPP | negative control for D-erythro-MAPP | negative control for D-erythro-MAPP | |
| 1101 | 0 | cp | mammea-a | other antibiotic | other antibiotic | |
| 1102 | 0 | cp | luzindole | Melatonin receptor antagonist | Melatonin receptor antagonist | |
| 1103 | 0 | cp | LY-278584 | Serotonin receptor antagonist | Serotonin receptor antagonist | |
| 1104 | 0 | cp | LY-16350 | Dopamine receptor agonist | Dopamine receptor agonist | |
| 1105 | 0 | cp | lawsone | Coloring agent | Coloring agent | |
| 1106 | 0 | cp | lavendustin-a | EGFR inhibitor | EGFR inhibitor | |
| 1107 | 0 | cp | L-750667 | Dopamine receptor antagonist | Dopamine receptor antagonist | |
| 1108 | 0 | cp | L-732138 | Tachykinin antagonist | Tachykinin antagonist | |
| 1109 | 0 | cp | L-152804 | Neuropeptide receptor antagonist | Neuropeptide receptor antagonist | |
| 1110 | 0 | cp | ketanserin | Serotonin receptor antagonist | Serotonin receptor antagonist | |
| 1111 | 0 | cp | KU-14R | Imidazoline receptor ligand | Imidazoline receptor ligand, Putative I3 antagonist | |
| 1112 | 0 | cp | KF-38789 | P-selectin inhibitor | P-selectin inhibitor | |
| 1113 | 0 | cp | kavain | Calcium channel modulator | Calcium channel modulator, Sodium channel blocker | |
| 1114 | 0 | cp | JTE-013 | Lysophospholipid receptor antagonist | Lysophospholipid receptor antagonist | |
| 1115 | 0 | cp | JAK3-inhibitor-VI | JAK inhibitor | JAK inhibitor | |
| 1116 | 0 | cp | irsogladine | Phosphodiesterase inhibitor | Phosphodiesterase inhibitor | |
| 1117 | 0 | cp | immepip | Histamine receptor agonist | Histamine receptor agonist | |
| 1118 | 0 | cp | ivermectin | GABA receptor agonist | GABA receptor agonist | |
| 1119 | 0 | cp | isamoltan | Adrenergic receptor antagonist | Adrenergic receptor antagonist | |
| 1120 | 0 | cp | irilin-a | Isoflavone | Isoflavone | |
| 1121 | 0 | cp | indole | aryl hydrocarbon receptor agonist | aryl hydrocarbon receptor agonist | |
| 1122 | 0 | cp | BRD-K66782112 | Histamine receptor antagonist | Histamine receptor antagonist | |
| 1123 | 0 | cp | bisbenzimide | DNA binding agent | DNA binding agent | |
| 1124 | 0 | cp | hexylcaine | Sodium channel blocker | Sodium channel blocker | |
| 1125 | 0 | cp | hydroquinidine | Antiarrhythmic | Antiarrhythmic | |
| 1126 | 0 | cp | hydrocortisone | Glucocorticoid receptor agonist | Glucocorticoid receptor agonist | |
| 1127 | 0 | cp | herniarin | Acetylcholinesterase inhibitor | Acetylcholinesterase inhibitor | |
| 1128 | 0 | cp | glimepiride | Insulin secretagogue | Insulin secretagogue | |
| 1129 | 0 | cp | gibberellic-acid | NFkB pathway inhibitor | NFkB pathway inhibitor | |
| 1130 | 0 | cp | GW-9508 | Free fatty acid receptor agonist | Free fatty acid receptor agonist, G protein-coupled receptor agonist | |
| 1131 | 0 | cp | GR-79236 | Adenosine receptor agonist | Adenosine receptor agonist | |
| 1132 | 0 | cp | GR-55562 | Serotonin receptor antagonist | Serotonin receptor antagonist | |
| 1133 | 0 | cp | GR-46611 | Serotonin receptor agonist | Serotonin receptor agonist | |
| 1134 | 0 | cp | GR-32191 | Thromboxane receptor antagonist | Thromboxane receptor antagonist | |
| 1135 | 0 | cp | BRD-K34608650 | Cannabinoid receptor agonist | Cannabinoid receptor agonist | |
| 1136 | 0 | cp | foliosidine | Plant alkaloid | Plant alkaloid | |
| 1137 | 0 | cp | fluvoxamine | Selective serotonin reuptake inhibitor (SSRI) | Selective serotonin reuptake inhibitor (SSRI) | |
| 1138 | 0 | cp | felodipine | Calcium channel blocker | Calcium channel blocker | |
| 1139 | 0 | cp | fraxidin | Carbonic anhydrase inhibitor | Carbonic anhydrase inhibitor | |
| 1140 | 0 | cp | FR-122047 | Cyclooxygenase inhibitor | Cyclooxygenase inhibitor | |
| 1141 | 0 | cp | FPL-55712 | Leukotriene receptor antagonist | Leukotriene receptor antagonist | |
| 1142 | 0 | cp | esculin | Antioxidant | Antioxidant | |
| 1143 | 0 | cp | eugenitol | Bacterial quorum sensing inhibitor | Bacterial quorum sensing inhibitor, Chromone, Monoamine oxidase inhibitor, Androgen receptor antagonist | |
| 1144 | 0 | cp | estradiol-valerate | Estrogen receptor agonist | Estrogen receptor agonist | |
| 1145 | 0 | cp | ergocornine | Dopamine receptor agonist | Dopamine receptor agonist | |
| 1146 | 0 | cp | ER-27319 | Mediator release inhibitor | Mediator release inhibitor, SYK inhibitor | |
| 1147 | 0 | cp | EMD-66684 | Angiotensin receptor antagonist | Angiotensin receptor antagonist | |
| 1148 | 0 | cp | digoxigenin | Steroid | Steroid |  |
| 1149 | 0 | cp | diflorasone | Corticosteroid agonist | Corticosteroid agonist, Glucocorticoid receptor agonist, Cytochrome P450 inhibitor, Immunosuppressant | |
| 1150 | 0 | cp | dehydroisoandosterone | GABA receptor modulator | GABA receptor modulator | |
| 1151 | 0 | cp | DY-131 | Estrogen receptor agonist | Estrogen receptor agonist | |
| 1152 | 0 | cp | DR-2313 | PARP inhibitor | PARP inhibitor | |
| 1153 | 0 | cp | docosatrienoic-acid | LTB4 inhibitor | LTB4 inhibitor | |
| 1154 | 0 | cp | DMEOB | glutamate receptor modulator | glutamate receptor modulator | |
| 1155 | 0 | cp | DMP-543 | Acetylcholine release stimulant | Acetylcholine release stimulant | |
| 1156 | 0 | cp | dictamnine | Furoquinoline alkaloid | Furoquinoline alkaloid | |
| 1157 | 0 | cp | ciclacillin | Bacterial cell wall synthesis inhibitor | Bacterial cell wall synthesis inhibitor | |
| 1158 | 0 | cp | cromakalim | Potassium channel activator | Potassium channel activator | |
| 1159 | 0 | cp | CO-102862 | Sodium channel blocker | Sodium channel blocker | |
| 1160 | 0 | cp | clomipramine | Serotonin transporter inhibitor (SERT) | Serotonin transporter inhibitor (SERT) | |
| 1161 | 0 | cp | ciprofibrate | PPAR receptor agonist | PPAR receptor agonist | |
| 1162 | 0 | cp | cimetidine | Histamine receptor antagonist | Histamine receptor antagonist | |
| 1163 | 0 | cp | cilostamide | Phosphodiesterase inhibitor | Phosphodiesterase inhibitor | |
| 1164 | 0 | cp | canrenoic-acid | Mineralocorticoid receptor antagonist | Mineralocorticoid receptor antagonist | |
| 1165 | 0 | cp | camptothecin | Topoisomerase inhibitor | Topoisomerase inhibitor | |
| 1166 | 0 | cp | cabergoline | Dopamine receptor agonist | Dopamine receptor agonist | |
| 1167 | 0 | cp | cymarin | ATPase inhibitor | ATPase inhibitor | |
| 1168 | 0 | cp | CP-94253 | Serotonin receptor agonist | Serotonin receptor agonist | |
| 1169 | 0 | cp | CP-93129 | Serotonin receptor agonist | Serotonin receptor agonist | |
| 1170 | 0 | cp | coumaric-acid | Antioxidant | Antioxidant | |
| 1171 | 0 | cp | CL-82198 | Metalloproteinase inhibitor | Metalloproteinase inhibitor | |
| 1172 | 0 | cp | CGP-53353 | EGFR inhibitor | EGFR inhibitor, PKC inhibitor | |
| 1173 | 0 | cp | CGP-37157 | L-type calcium channel blocker | L-type calcium channel blocker, Mitochondrial Na+/Ca2+ exchanger antagonist, Sodium/calcium exchange inhibitor | |
| 1174 | 0 | cp | CGP-12177 | Adrenergic receptor agonist | Adrenergic receptor agonist | |
| 1175 | 0 | cp | CGK-733 | ATR kinase inhibitor | ATR kinase inhibitor | |
| 1176 | 0 | cp | CCCP | Mitochondrial oxidative phosphorylation uncoupler | Mitochondrial oxidative phosphorylation uncoupler | |
| 1177 | 0 | cp | carbacyclin | IP receptor activator | IP receptor activator, PPAR receptor agonist | |
| 1178 | 0 | cp | boldine | Acetylcholine receptor antagonist | Acetylcholine receptor antagonist, Dopamine receptor antagonist | |
| 1179 | 0 | cp | biperiden | Acetylcholine receptor antagonist | Acetylcholine receptor antagonist | |
| 1180 | 0 | cp | BW-723C86 | Serotonin receptor agonist | Serotonin receptor agonist | |
| 1181 | 0 | cp | buddleoflavonoloside | Acetylcholinesterase inhibitor | Acetylcholinesterase inhibitor | |
| 1182 | 0 | cp | BU-239 | Imidazoline receptor agonist | Imidazoline receptor agonist, Imidazoline receptor ligand | |
| 1183 | 0 | cp | BRL-15572 | Serotonin receptor antagonist | Serotonin receptor antagonist | |
| 1184 | 0 | cp | BP-554 | Serotonin receptor agonist | Serotonin receptor agonist | |
| 1185 | 0 | cp | BMY-14802 | Sigma receptor antagonist | Sigma receptor antagonist | |
| 1186 | 0 | cp | BAY-59-3074 | Cannabinoid receptor partial agonist | Cannabinoid receptor partial agonist | |
| 1187 | 0 | cp | BAPTA-AM | Potassium channel blocker | Potassium channel blocker | |
| 1188 | 0 | cp | arecaidine | Acetylcholine receptor agonist | Acetylcholine receptor agonist | |
| 1189 | 0 | cp | aminogenistein | SRC inhibitor | SRC inhibitor | |
| 1190 | 0 | cp | amiloride | Sodium channel blocker | Sodium channel blocker | |
| 1191 | 0 | cp | aloisine | CDK inhibitor | CDK inhibitor, CFTR channel agonist, Glycogen synthase kinase inhibitor | |
| 1192 | 0 | cp | AZ-10417808 | Caspase inhibitor | Caspase inhibitor | |
| 1193 | 0 | cp | AY-9944 | Hedgehog pathway modulator | Hedgehog pathway modulator | |
| 1194 | 0 | cp | AQ-RA741 | Acetylcholine receptor antagonist | Acetylcholine receptor antagonist | |
| 1195 | 0 | cp | anandamide | Cannabinoid receptor agonist | Cannabinoid receptor agonist | |
| 1196 | 0 | cp | aminomethyltransferase | Nitric oxide synthase inhibitor | Nitric oxide synthase inhibitor | |
| 1197 | 0 | cp | AM-630 | Cannabinoid receptor antagonist | Cannabinoid receptor antagonist | |
| 1198 | 0 | cp | AM-404 | Cyclooxygenase inhibitor | Cyclooxygenase inhibitor, FAAH inhibitor, TRPV antagonist | |
| 1199 | 0 | cp | alverine | Muscle relaxant | Muscle relaxant | |
| 1200 | 0 | cp | tyrphostin-1 | EGFR inhibitor | EGFR inhibitor | |
| 1201 | 0 | cp | tyrphostin-AG-825 | Receptor tyrosine protein kinase inhibitor | Receptor tyrosine protein kinase inhibitor | |
| 1202 | 0 | cp | tyrphostin | EGFR inhibitor | EGFR inhibitor | |
| 1203 | 0 | cp | AG-494 | EGFR inhibitor | EGFR inhibitor, Tyrosine kinase inhibitor | |
| 1204 | 0 | cp | tyrphostin-AG-494 | EGFR inhibitor | EGFR inhibitor | |
| 1205 | 0 | cp | maackiain | Sodium/glucose cotransporter inhibitor | Sodium/glucose cotransporter inhibitor | |
| 1206 | 0 | cp | epibatidine | Acetylcholine receptor agonist | Acetylcholine receptor agonist | |
| 1207 | 0 | cp | ZK-756326 | CC chemokine receptor ligand | CC chemokine receptor ligand | |
| 1208 | 0 | cp | verteporfin | Photosensitizing agent | Photosensitizing agent | |
| 1209 | 0 | cp | VAMA-37 | DNA dependent protein kinase inhibitor | DNA dependent protein kinase inhibitor | |
| 1210 | 0 | cp | U-54494A | Opioid receptor agonist | Opioid receptor agonist | |
| 1211 | 0 | cp | tribenoside | Anti-inflammatory | Anti-inflammatory, Capillary stabilizing agent | |
| 1212 | 0 | cp | trimetozine | Sedative | Sedative |  |
| 1213 | 0 | cp | alimemazine | Histamine receptor agonist | Histamine receptor agonist | |
| 1214 | 0 | cp | tranylcypromine | Monoamine oxidase inhibitor | Monoamine oxidase inhibitor | |
| 1215 | 0 | cp | terfenadine | Histamine receptor antagonist | Histamine receptor antagonist | |
| 1216 | 0 | cp | spiperone | Dopamine receptor antagonist | Dopamine receptor antagonist | |
| 1217 | 0 | cp | SKF-96365 | Calcium channel blocker | Calcium channel blocker | |
| 1218 | 0 | cp | SB-258585 | Serotonin receptor antagonist | Serotonin receptor antagonist | |
| 1219 | 0 | cp | S-14506 | Serotonin receptor agonist | Serotonin receptor agonist | |
| 1220 | 0 | cp | resmethrin | Cytochrome P450 inhibitor | Cytochrome P450 inhibitor | |
| 1221 | 0 | cp | RX-821002 | Adrenergic receptor antagonist | Adrenergic receptor antagonist | |
| 1222 | 0 | cp | rolipram | Phosphodiesterase inhibitor | Phosphodiesterase inhibitor, Interleukin receptor antagonist | |
| 1223 | 0 | cp | Ro-04-6790 | Serotonin receptor antagonist | Serotonin receptor antagonist | |
| 1224 | 0 | cp | racephedrine | Adrenergic receptor agonist | Adrenergic receptor agonist | |
| 1225 | 0 | cp | QX-222 | Sodium channel blocker | Sodium channel blocker | |
| 1226 | 0 | cp | purvalanol-a | CDK inhibitor | CDK inhibitor, DYRK inhibitor | |
| 1227 | 0 | cp | PSB-069 | NTPDase inhibitor | NTPDase inhibitor | |
| 1228 | 0 | cp | proadifen | Nitric oxide synthase inhibitor | Nitric oxide synthase inhibitor | |
| 1229 | 0 | cp | prednisolone | Glucocorticoid receptor agonist | Glucocorticoid receptor agonist | |
| 1230 | 0 | cp | phenamil | TRPV antagonist | TRPV antagonist | |
| 1231 | 0 | cp | PHCCC | Glutamate receptor agonist | Glutamate receptor agonist | |
| 1232 | 0 | cp | naringenin | Aromatase inhibitor | Aromatase inhibitor, TRPV antagonist | |
| 1233 | 0 | cp | norgestimate | Progesterone receptor agonist | Progesterone receptor agonist | |
| 1234 | 0 | cp | N6-cyclopentyladenosine | Adenosine receptor agonist | Adenosine receptor agonist | |
| 1235 | 0 | cp | n-formylmethionylalanine | macrophage activator | macrophage activator | |
| 1236 | 0 | cp | mycophenolic-acid | Dehydrogenase inhibitor | Dehydrogenase inhibitor, Inositol monophosphatase inhibitor | |
| 1237 | 0 | cp | metyrapone | Cytochrome P450 inhibitor | Cytochrome P450 inhibitor | |
| 1238 | 0 | cp | mitomycin-c | DNA alkylating agent | DNA alkylating agent, DNA inhibitor, DNA synthesis inhibitor | |
| 1239 | 0 | cp | metolazone | Carbonic anhydrase inhibitor | Carbonic anhydrase inhibitor | |
| 1240 | 0 | cp | methysergide | Serotonin receptor antagonist | Serotonin receptor antagonist | |
| 1241 | 0 | cp | met-leu-phe | -666 | -666 |  |
| 1242 | 0 | cp | mebeverine | Acetylcholine receptor antagonist | Acetylcholine receptor antagonist | |
| 1243 | 0 | cp | laudanosine | Central nervous system agent | Central nervous system agent | |
| 1244 | 0 | cp | LY-83583 | Guanylyl cyclase inhibitor | Guanylyl cyclase inhibitor | |
| 1245 | 0 | cp | LY-294002 | MTOR inhibitor | MTOR inhibitor, PI3K inhibitor, DNA dependent protein kinase inhibitor, Phosphodiesterase inhibitor, PLK inhibitor | |
| 1246 | 0 | cp | LY-344864 | Serotonin receptor agonist | Serotonin receptor agonist | |
| 1247 | 0 | cp | lupanine | Sodium channel blocker | Sodium channel blocker | |
| 1248 | 0 | cp | loxapine | Dopamine receptor antagonist | Dopamine receptor antagonist, Dopamine receptor ligand, Serotonin receptor antagonist | |
| 1249 | 0 | cp | linoleic-acid | Oxidative stress inducer | Oxidative stress inducer | |
| 1250 | 0 | cp | l-stepholidine | Dopamine receptor antagonist | Dopamine receptor antagonist | |
| 1251 | 0 | cp | L-168049 | Glucagon receptor antagonist | Glucagon receptor antagonist | |
| 1252 | 0 | cp | icilin | TRPV agonist | TRPV agonist | |
| 1253 | 0 | cp | hydroquinine | Antiarrhythmic | Antiarrhythmic | |
| 1254 | 0 | cp | hydrocotarnine | Opioid receptor antagonist | Opioid receptor antagonist | |
| 1255 | 0 | cp | hydrocortisone | Glucocorticoid receptor agonist | Glucocorticoid receptor agonist | |
| 1256 | 0 | cp | hexylresorcinol | Local anesthetic | Local anesthetic | |
| 1257 | 0 | cp | gitoxigenin | ATPase inhibitor | ATPase inhibitor | |
| 1258 | 0 | cp | FGIN-1-43 | Benzodiazepine receptor agonist | Benzodiazepine receptor agonist | |
| 1259 | 0 | cp | ezetimibe | Niemann-Pick C1-like 1 protein antagonist | Niemann-Pick C1-like 1 protein antagonist, Cholesterol inhibitor | |
| 1260 | 0 | cp | etomidate | GABA receptor modulator | GABA receptor modulator | |
| 1261 | 0 | cp | ethoprop | Acetylcholinesterase inhibitor | Acetylcholinesterase inhibitor | |
| 1262 | 0 | cp | estradiol-benzoate | Estrogen receptor agonist | Estrogen receptor agonist | |
| 1263 | 0 | cp | ephedrine | Adrenergic receptor agonist | Adrenergic receptor agonist | |
| 1264 | 0 | cp | berbamine | Calmodulin antagonist | Calmodulin antagonist | |
| 1265 | 0 | cp | domperidone | Dopamine receptor antagonist | Dopamine receptor antagonist | |
| 1266 | 0 | cp | dipropyl-dopamine | Dopamine receptor agonist | Dopamine receptor agonist | |
| 1267 | 0 | cp | diclofenac | Cyclooxygenase inhibitor | Cyclooxygenase inhibitor | |
| 1268 | 0 | cp | desoxypeganine | Acetylcholinesterase inhibitor | Acetylcholinesterase inhibitor, Monoamine oxidase inhibitor | |
| 1269 | 0 | cp | deracoxib | Cyclooxygenase inhibitor | Cyclooxygenase inhibitor | |
| 1270 | 0 | cp | DCPIB | Chloride channel blocker | Chloride channel blocker, Gap junction modulator, Glutamate inhibitor | |
| 1271 | 0 | cp | daunorubicin | RNA synthesis inhibitor | RNA synthesis inhibitor, Topoisomerase inhibitor | |
| 1272 | 0 | cp | corynanthine | Adrenergic receptor antagonist | Adrenergic receptor antagonist | |
| 1273 | 0 | cp | CO-101244 | Ionotropic glutamate receptor antagonist | Ionotropic glutamate receptor antagonist | |
| 1274 | 0 | cp | CV-1808 | Adenosine receptor agonist | Adenosine receptor agonist | |
| 1275 | 0 | cp | coumarin | Vitamin K antagonist | Vitamin K antagonist | |
| 1276 | 0 | cp | clemastine | Histamine receptor antagonist | Histamine receptor antagonist | |
| 1277 | 0 | cp | chlormadinone | 5-alpha reductase inhibitor | 5-alpha reductase inhibitor, Progesterone receptor agonist | |
| 1278 | 0 | cp | carbinoxamine | Histamine receptor antagonist | Histamine receptor antagonist, L-type calcium channel blocker | |
| 1279 | 0 | cp | ceramide | Phosphoenolpyruvate carboxylase activator | Phosphoenolpyruvate carboxylase activator, Serine/threonine protein phosphatase activator | |
| 1280 | 0 | cp | BW-B70C | Lipoxygenase inhibitor | Lipoxygenase inhibitor | |
| 1281 | 0 | cp | BMS-182874 | Endothelin receptor antagonist | Endothelin receptor antagonist | |
| 1282 | 0 | cp | betamethasone | Glucocorticoid receptor agonist | Glucocorticoid receptor agonist | |
| 1283 | 0 | cp | asiaticoside | Antineoplastic | Antineoplastic | |
| 1284 | 0 | cp | anpirtoline | Serotonin receptor agonist | Serotonin receptor agonist | |
| 1285 | 0 | cp | CS-1657 | PARP inhibitor | PARP inhibitor | |
| 1286 | 0 | cp | ATPA | Glutamate receptor agonist | Glutamate receptor agonist | |
| 1287 | 0 | cp | amiodarone | Potassium channel blocker | Potassium channel blocker | |
| 1288 | 0 | cp | ALX-5407 | Glycine transporter inhibitor | Glycine transporter inhibitor | |
| 1289 | 0 | cp | alclometasone | Glucocorticoid receptor agonist | Glucocorticoid receptor agonist, Immunosuppressant | |
| 1290 | 0 | cp | tyrphostin-AG-82 | EGFR inhibitor | EGFR inhibitor | |
| 1291 | 0 | cp | palbociclib | CDK inhibitor | CDK inhibitor | |
| 1292 | 0 | cp | OSI-027 | MTOR inhibitor | MTOR inhibitor | |
| 1293 | 0 | cp | MLN-8054 | Aurora kinase inhibitor | Aurora kinase inhibitor, Mitotic inhibitor, Protein kinase inhibitor | |
| 1294 | 0 | cp | KIN001-127 | ITK inhibitor | ITK inhibitor | |
| 1295 | 0 | cp | GSK-461364 | PLK inhibitor | PLK inhibitor | |
| 1296 | 0 | cp | GSK-429286A | Rho associated kinase inhibitor | Rho associated kinase inhibitor | |
| 1297 | 0 | cp | quizartinib | FLT3 inhibitor | FLT3 inhibitor | |
| 1298 | 0 | cp | epinephrine | carbonic anhydrase activator | carbonic anhydrase activator | |
| 1299 | 0 | cp | prazosin | Adrenergic receptor antagonist | Adrenergic receptor antagonist | |
| 1300 | 0 | cp | iopanoic-acid | Radiopaque medium | Radiopaque medium | |
| 1301 | 0 | cp | hymecromone | Monoamine oxidase inhibitor | Monoamine oxidase inhibitor | |
| 1302 | 0 | cp | gliquidone | Sulfonylurea | Sulfonylurea | |
| 1303 | 0 | cp | bicalutamide | Androgen receptor antagonist | Androgen receptor antagonist | |
| 1304 | 0 | cp | bemegride | Chemoreceptor agonist | Chemoreceptor agonist | |
| 1305 | 0 | cp | clofibric-acid | PPAR receptor agonist | PPAR receptor agonist | |
| 1306 | 0 | cp | quipazine | Serotonin receptor agonist | Serotonin receptor agonist | |
| 1307 | 0 | cp | dibenzepin | Norepinephrine reuptake inhibitor | Norepinephrine reuptake inhibitor | |
| 1308 | 0 | cp | brompheniramine | Histamine receptor antagonist | Histamine receptor antagonist | |
| 1309 | 0 | cp | amisulpride | Dopamine receptor antagonist | Dopamine receptor antagonist | |
| 1310 | 0 | cp | sumatriptan | Serotonin receptor agonist | Serotonin receptor agonist | |
| 1311 | 0 | cp | isoxicam | Cyclooxygenase inhibitor | Cyclooxygenase inhibitor | |
| 1312 | 0 | cp | fenofibrate | PPAR receptor agonist | PPAR receptor agonist | |
| 1313 | 0 | cp | azacyclonol | Histamine receptor antagonist | Histamine receptor antagonist | |
| 1314 | 0 | cp | amoxicillin | Penicillin binding protein inhibitor | Penicillin binding protein inhibitor | |
| 1315 | 0 | cp | amitriptyline | Norepinephrine inhibitor | Norepinephrine inhibitor, Norepinephrine reuptake inhibitor, Serotonin receptor antagonist, Serotonin reuptake inhibitor | |
| 1316 | 0 | cp | pindolol | Adrenergic receptor antagonist | Adrenergic receptor antagonist, Serotonin receptor antagonist | |
| 1317 | 0 | cp | naphazoline | Adrenergic receptor agonist | Adrenergic receptor agonist | |
| 1318 | 0 | cp | benzonatate | Local anesthetic | Local anesthetic | |
| 1319 | 0 | cp | phylloquinone | Vitamin K | Vitamin K, Gamma carboxylase enzyme | |
| 1320 | 0 | cp | glipizide | Sulfonylurea | Sulfonylurea | |
| 1321 | 0 | cp | gestrinone | Progesterone receptor antagonist | Progesterone receptor antagonist | |
| 1322 | 0 | cp | ofloxacin | Bacterial DNA gyrase inhibitor | Bacterial DNA gyrase inhibitor | |
| 1323 | 0 | cp | acetylcholine | Acetylcholine receptor agonist | Acetylcholine receptor agonist | |
| 1324 | 0 | cp | amcinonide | Glucocorticoid receptor agonist | Glucocorticoid receptor agonist | |
| 1325 | 0 | cp | JNJ-38877605 | Tyrosine kinase inhibitor | Tyrosine kinase inhibitor | |
| 1326 | 0 | cp | sulindac | Cyclooxygenase inhibitor | Cyclooxygenase inhibitor | |
| 1327 | 0 | cp | rimantadine | Antiviral | Antiviral, RNA synthesis inhibitor | |
| 1328 | 0 | cp | doxycycline | Bacterial 30S ribosomal subunit inhibitor | Bacterial 30S ribosomal subunit inhibitor, Metalloproteinase inhibitor | |
| 1329 | 0 | cp | cefoxitin | Bacterial cell wall synthesis inhibitor | Bacterial cell wall synthesis inhibitor | |
| 1330 | 0 | cp | bosentan | Endothelin receptor antagonist | Endothelin receptor antagonist | |
| 1331 | 0 | cp | genipin | Choleretic agent | Choleretic agent | |
| 1332 | 0 | cp | zidovudine | Reverse transcriptase inhibitor | Reverse transcriptase inhibitor | |
| 1333 | 0 | cp | bethanechol | Acetylcholine receptor agonist | Acetylcholine receptor agonist | |
| 1334 | 0 | cp | aminolevulinic-acid | Oxidizing agent | Oxidizing agent | |
| 1335 | 0 | cp | phenylbutyrate | HDAC inhibitor | HDAC inhibitor | |
| 1336 | 0 | cp | ramipril | ACE inhibitor | ACE inhibitor | |
| 1337 | 0 | cp | griseofulvin | Tubulin inhibitor | Tubulin inhibitor | |
| 1338 | 0 | cp | enalapril | ACE inhibitor | ACE inhibitor | |
| 1339 | 0 | cp | iodoacetic-acid | Cysteine peptidase inhibitor | Cysteine peptidase inhibitor | |
| 1340 | 0 | cp | edaravone | Nootropic agent | Nootropic agent | |
| 1341 | 0 | cp | megestrol | progesterone receptor agonist | progesterone receptor agonist | |
| 1342 | 0 | cp | bimatoprost | Prostanoid receptor agonist | Prostanoid receptor agonist | |
| 1343 | 0 | cp | spectinomycin | Bacterial 30S ribosomal subunit inhibitor | Bacterial 30S ribosomal subunit inhibitor | |
| 1344 | 0 | cp | propantheline | Acetylcholine receptor antagonist | Acetylcholine receptor antagonist | |
| 1345 | 0 | cp | levonorgestrel | Estrogen receptor agonist | Estrogen receptor agonist, Glucocorticoid receptor antagonist, Progesterone receptor agonist, Progesterone receptor antagonist | |
| 1346 | 0 | cp | methapyrilene | Histamine receptor antagonist | Histamine receptor antagonist | |
| 1347 | 0 | cp | ascorbic-acid | Antioxidant | Antioxidant | |
| 1348 | 0 | cp | tetrahydrobiopterin | Nitric oxide stimulant | Nitric oxide stimulant, Nitric oxide synthase stimulant, Phenylalanine 4-hydroxylase stimulant | |
| 1349 | 0 | cp | montelukast | Leukotriene receptor antagonist | Leukotriene receptor antagonist | |
| 1350 | 0 | cp | norepinephrine | Adrenergic receptor agonist | Adrenergic receptor agonist | |
| 1351 | 0 | cp | sulfafurazole | Bacterial antifolate | Bacterial antifolate | |
| 1352 | 0 | cp | probenecid | Uricosuric blocker | Uricosuric blocker | |
| 1353 | 0 | cp | hydroflumethiazide | Sodium/potassium/chloride transporter inhibitor | Sodium/potassium/chloride transporter inhibitor | |
| 1354 | 0 | cp | triamcinolone | Glucocorticoid receptor agonist | Glucocorticoid receptor agonist, Corticosteroid agonist, Immunosuppressant | |
| 1355 | 0 | cp | pyridine-2-aldoxime | Acetylcholinesterase inhibitor | Acetylcholinesterase inhibitor | |
| 1356 | 0 | cp | mefloquine | Adenosine receptor antagonist | Adenosine receptor antagonist, Hemoglobin antagonist | |
| 1357 | 0 | cp | sulfacetamide | PABA antagonist | PABA antagonist | |
| 1358 | 0 | cp | fluocinolone | Glucocorticoid receptor agonist | Glucocorticoid receptor agonist, Corticosteroid agonist | |
| 1359 | 0 | cp | terbutaline | Adrenergic receptor agonist | Adrenergic receptor agonist | |
| 1360 | 0 | cp | oxybutynin | Acetylcholine receptor antagonist | Acetylcholine receptor antagonist | |
| 1361 | 0 | cp | mercaptopurine | Immunosuppressant | Immunosuppressant, Protein synthesis inhibitor, Purine antagonist | |
| 1362 | 0 | cp | flecainide | Sodium channel blocker | Sodium channel blocker | |
| 1363 | 0 | cp | raloxifene | Estrogen receptor antagonist | Estrogen receptor antagonist, Selective estrogen receptor modulator (SERM) | |
| 1364 | 0 | cp | edrophonium | Acetylcholinesterase inhibitor | Acetylcholinesterase inhibitor | |
| 1365 | 0 | cp | dapsone | Bacterial antifolate | Bacterial antifolate | |
| 1366 | 0 | cp | cycloserine | Bacterial cell wall synthesis inhibitor | Bacterial cell wall synthesis inhibitor | |
| 1367 | 0 | cp | cefuroxime | Bacterial cell wall synthesis inhibitor | Bacterial cell wall synthesis inhibitor | |
| 1368 | 0 | cp | azacitidine | DNA methyltransferase inhibitor | DNA methyltransferase inhibitor | |
| 1369 | 0 | cp | metformin | Insulin sensitizer | Insulin sensitizer | |
| 1370 | 0 | cp | marbofloxacin | Bacterial DNA gyrase inhibitor | Bacterial DNA gyrase inhibitor | |
| 1371 | 0 | cp | abiraterone | 17,20 lyase inhibitor | 17,20 lyase inhibitor, Androgen biosynthesis inhibitor, Cytochrome P450 inhibitor, Steroid sulfatase inhibitor | |
| 1372 | 0 | cp | bromfenac | Cyclooxygenase inhibitor | Cyclooxygenase inhibitor | |
| 1373 | 0 | cp | tienilic-acid | Sodium/potassium/chloride transporter inhibitor | Sodium/potassium/chloride transporter inhibitor | |
| 1374 | 0 | cp | tolcapone | Catechol O methyltransferase inhibitor | Catechol O methyltransferase inhibitor | |
| 1375 | 0 | cp | quinidine | Sodium channel blocker | Sodium channel blocker | |
| 1376 | 0 | cp | nomifensine | Dopamine uptake inhibitor | Dopamine uptake inhibitor, Noradrenaline uptake inhibitor | |
| 1377 | 0 | cp | streptozotocin | DNA alkylating agent | DNA alkylating agent | |
| 1378 | 0 | cp | tubacin | HDAC inhibitor | HDAC inhibitor | |
| 1379 | 0 | cp | danoprevir | HCV inhibitor | HCV inhibitor | |
| 1380 | 0 | cp | eicosatetraynoic-acid | Cyclooxygenase inhibitor | Cyclooxygenase inhibitor, Lipoxygenase inhibitor | |
| 1381 | 0 | cp | tyrphostin-A9 | Protein tyrosine kinase inhibitor | Protein tyrosine kinase inhibitor, Tyrosine kinase inhibitor | |
| 1382 | 0 | cp | androstenol | GABA receptor modulator | GABA receptor modulator | |
| 1383 | 0 | cp | CAY-10577 | Casein kinase inhibitor | Casein kinase inhibitor | |
| 1384 | 0 | cp | SNS-314 | Aurora kinase inhibitor | Aurora kinase inhibitor | |
| 1385 | 0 | cp | carbetocin | Oxytocin receptor agonist | Oxytocin receptor agonist | |
| 1386 | 0 | cp | cyclopamine | Smoothened receptor antagonist | Smoothened receptor antagonist | |
| 1387 | 0 | cp | tofacitinib | JAK inhibitor | JAK inhibitor | |
| 1388 | 0 | cp | sitagliptin | Dipeptidyl peptidase inhibitor | Dipeptidyl peptidase inhibitor | |
| 1389 | 0 | cp | zibotentan | Endothelin receptor antagonist | Endothelin receptor antagonist | |
| 1390 | 0 | cp | HY-11007 | BCR-ABL kinase inhibitor | BCR-ABL kinase inhibitor | |
| 1391 | 0 | cp | tunicamycin | GLCNAC phosphotransferase inhibitor | GLCNAC phosphotransferase inhibitor | |
| 1392 | 0 | cp | CAY-10470 | NFkB pathway inhibitor | NFkB pathway inhibitor | |
| 1393 | 0 | cp | BIX-01294 | Histone lysine methyltransferase inhibitor | Histone lysine methyltransferase inhibitor, DNA methyltransferase inhibitor | |
| 1394 | 0 | cp | xanthinol | Vasodilator | Vasodilator | |
| 1395 | 0 | cp | olopatadine | Histamine receptor antagonist | Histamine receptor antagonist | |
| 1396 | 0 | cp | nialamide | Monoamine oxidase inhibitor | Monoamine oxidase inhibitor | |
| 1397 | 0 | cp | doxapram | Potassium channel blocker | Potassium channel blocker | |
| 1398 | 0 | cp | balsalazide | Cyclooxygenase inhibitor | Cyclooxygenase inhibitor | |
| 1399 | 0 | cp | dicloxacillin | Bacterial cell wall synthesis inhibitor | Bacterial cell wall synthesis inhibitor | |
| 1400 | 0 | cp | carbidopa | Aromatic L-amino acid decarboxylase inhibitor | Aromatic L-amino acid decarboxylase inhibitor | |
| 1401 | 0 | cp | azithromycin | Bacterial 50S ribosomal subunit inhibitor | Bacterial 50S ribosomal subunit inhibitor | |
| 1402 | 0 | cp | VU-0404997-2 | Glutamate receptor modulator | Glutamate receptor modulator | |
| 1403 | 0 | cp | VU-0366037-2 | Glutamate receptor modulator | Glutamate receptor modulator | |
| 1404 | 0 | cp | mafenide | Carbonic anhydrase inhibitor | Carbonic anhydrase inhibitor | |
| 1405 | 0 | cp | menadione | Mitochondrial DNA polymerase inhibitor | Mitochondrial DNA polymerase inhibitor, Phosphatase inhibitor | |
| 1406 | 0 | cp | 3-amino-benzamide | PARP inhibitor | PARP inhibitor | |
| 1407 | 0 | cp | triacsin-c | Adrenergic receptor antagonist | Adrenergic receptor antagonist | |
| 1408 | 0 | cp | parbendazole | Tubulin inhibitor | Tubulin inhibitor | |
| 1409 | 0 | cp | linsitinib | IGF-1 inhibitor | IGF-1 inhibitor | |
| 1410 | 0 | cp | evodiamine | ATPase inhibitor | ATPase inhibitor, TRPV agonist | |
| 1411 | 0 | cp | DL-PDMP | Glucosyltransferase inhibitor | Glucosyltransferase inhibitor | |
| 1412 | 0 | cp | ABT-737 | BCL inhibitor | BCL inhibitor | |
| 1413 | 0 | cp | AC-55649 | Retinoid receptor agonist | Retinoid receptor agonist | |
| 1414 | 0 | cp | cytochalasin-b | Microtubule inhibitor | Microtubule inhibitor | |
| 1415 | 0 | cp | SCH-28080 | ATPase inhibitor | ATPase inhibitor | |
| 1416 | 0 | cp | NS-3694 | Glutamate receptor antagonist | Glutamate receptor antagonist | |
| 1417 | 0 | cp | BRD-K41143549 | Glutamate receptor antagonist | Glutamate receptor antagonist | |
| 1418 | 0 | cp | moracizine | Sodium channel blocker | Sodium channel blocker | |
| 1419 | 0 | cp | MDL-73005EF | Serotonin receptor antagonist | Serotonin receptor antagonist | |
| 1420 | 0 | cp | L-741742 | Dopamine receptor antagonist | Dopamine receptor antagonist | |
| 1421 | 0 | cp | isoxsuprine | Adrenergic receptor agonist | Adrenergic receptor agonist | |
| 1422 | 0 | cp | imipramine | Norepinephrine reuptake inhibitor | Norepinephrine reuptake inhibitor, Serotonin reuptake inhibitor | |
| 1423 | 0 | cp | glycodeoxycholic-acid | Apoptosis stimulant | Apoptosis stimulant | |
| 1424 | 0 | cp | gemfibrozil | Lipoprotein lipase activator | Lipoprotein lipase activator | |
| 1425 | 0 | cp | FG-7142 | GABA benzodiazepine site receptor inverse agonist | GABA benzodiazepine site receptor inverse agonist | |
| 1426 | 0 | cp | ethinyl-estradiol | DNA directed DNA polymerase stimulant | DNA directed DNA polymerase stimulant, Estrogenic component in oral contraceptives, Estrogen receptor agonist | |
| 1427 | 0 | cp | dipivefrine | Adrenergic receptor agonist | Adrenergic receptor agonist | |
| 1428 | 0 | cp | trimipramine | Norepinephrine reuptake inhibitor | Norepinephrine reuptake inhibitor, Tricyclic antidepressant | |
| 1429 | -0.01 | cp | tolbutamide | ATP channel blocker | ATP channel blocker | |
| 1430 | -0.02 | cp | WZ-4002 | EGFR inhibitor | EGFR inhibitor | |
| 1431 | -0.02 | cp | CP-724714 | EGFR inhibitor | EGFR inhibitor, Receptor tyrosine protein kinase inhibitor, Tyrosine kinase inhibitor | |
| 1432 | -0.02 | cp | ranolazine | Sodium channel blocker | Sodium channel blocker | |
| 1433 | -0.02 | cp | pifithrin-alpha | TP53 inhibitor | TP53 inhibitor | |
| 1434 | -0.02 | cp | promazine | Dopamine receptor antagonist | Dopamine receptor antagonist | |
| 1435 | -0.02 | cp | L-755507 | Adrenergic receptor agonist | Adrenergic receptor agonist | |
| 1436 | -0.02 | cp | bisphenol-a | PPAR receptor antagonist | PPAR receptor antagonist | |
| 1437 | -0.02 | cp | HDAC3-selective | HDAC inhibitor | HDAC inhibitor | |
| 1438 | -0.02 | cp | PD-173074 | FGFR inhibitor | FGFR inhibitor, VEGFR inhibitor | |
| 1439 | -0.02 | cp | 15-delta-prostaglandin-j2 | PPAR receptor agonist | PPAR receptor agonist | |
| 1440 | -0.03 | cp | disulfiram | Aldehyde dehydrogenase inhibitor | Aldehyde dehydrogenase inhibitor, TRPV agonist, DNA methyltransferase inhibitor | |
| 1441 | -0.03 | cp | mevastatin | HMGCR inhibitor | HMGCR inhibitor | |
| 1442 | -0.03 | cp | L-694247 | Serotonin receptor agonist | Serotonin receptor agonist | |
| 1443 | -0.03 | cp | calcipotriol | Vitamin D receptor agonist | Vitamin D receptor agonist | |
| 1444 | -0.03 | cp | enzastaurin | PKC inhibitor | PKC inhibitor | |
| 1445 | -0.03 | cp | procarbazine | Monoamine oxidase inhibitor | Monoamine oxidase inhibitor | |
| 1446 | -0.03 | cp | verapamil | Calcium channel blocker | Calcium channel blocker | |
| 1447 | -0.04 | cp | sulconazole | Sterol demethylase inhibitor | Sterol demethylase inhibitor | |
| 1448 | -0.04 | cp | azathioprine | Dehydrogenase inhibitor | Dehydrogenase inhibitor | |
| 1449 | -0.07 | cp | mesoridazine | Dopamine receptor antagonist | Dopamine receptor antagonist | |
| 1450 | -0.07 | cp | isocarboxazid | Monoamine oxidase inhibitor | Monoamine oxidase inhibitor | |
| 1451 | -0.07 | cp | equilin | Estrogen receptor agonist | Estrogen receptor agonist | |
| 1452 | -0.07 | cp | manumycin-a | Farnesyltransferase inhibitor | Farnesyltransferase inhibitor, NFkB pathway inhibitor | |
| 1453 | -0.07 | cp | rizatriptan | Serotonin receptor agonist | Serotonin receptor agonist | |
| 1454 | -0.07 | cp | 6-benzylaminopurine | Purinergic receptor activator | Purinergic receptor activator | |
| 1455 | -0.07 | cp | rabeprazole | ATPase inhibitor | ATPase inhibitor, Gastrin inhibitor | |
| 1456 | -0.07 | cp | elesclomol | Oxidative stress inducer | Oxidative stress inducer | |
| 1457 | -0.07 | cp | BAX-channel-blocker | Cytochrome C release inhibitor | Cytochrome C release inhibitor | |
| 1458 | -0.08 | cp | SKF-83566 | Dopamine receptor antagonist | Dopamine receptor antagonist | |
| 1459 | -0.11 | cp | latanoprost | Prostanoid receptor agonist | Prostanoid receptor agonist | |
| 1460 | -0.11 | cp | nafcillin | Bacterial cell wall synthesis inhibitor | Bacterial cell wall synthesis inhibitor | |
| 1461 | -0.11 | cp | melperone | Serotonin receptor antagonist | Serotonin receptor antagonist, Dopamine receptor antagonist | |
| 1462 | -0.11 | cp | quetiapine | Dopamine receptor antagonist | Dopamine receptor antagonist, Serotonin receptor antagonist | |
| 1463 | -0.11 | cp | prednicarbate | Phospholipase activator | Phospholipase activator | |
| 1464 | -0.11 | cp | prochlorperazine | Dopamine receptor antagonist | Dopamine receptor antagonist | |
| 1465 | -0.11 | cp | MRS-1334 | Adenosine receptor antagonist | Adenosine receptor antagonist | |
| 1466 | -0.11 | cp | indometacin | Cyclooxygenase inhibitor | Cyclooxygenase inhibitor | |
| 1467 | -0.11 | cp | anagrelide | Phosphodiesterase inhibitor | Phosphodiesterase inhibitor | |
| 1468 | -0.11 | cp | gefitinib | EGFR inhibitor | EGFR inhibitor | |
| 1469 | -0.11 | cp | urapidil | Adrenergic receptor antagonist | Adrenergic receptor antagonist | |
| 1470 | -0.11 | cp | tocainide | Sodium channel blocker | Sodium channel blocker | |
| 1471 | -0.11 | cp | meprylcaine | Local anesthetic | Local anesthetic | |
| 1472 | -0.11 | cp | cyclophosphamide | DNA alkylating agent | DNA alkylating agent | |
| 1473 | -0.11 | cp | AM-251 | Cannabinoid receptor antagonist | Cannabinoid receptor antagonist | |
| 1474 | -0.11 | cp | pimozide | Dopamine receptor antagonist | Dopamine receptor antagonist | |
| 1475 | -0.11 | cp | trifluoperazine | Dopamine receptor antagonist | Dopamine receptor antagonist | |
| 1476 | -0.11 | cp | venlafaxine | Adrenergic inhibitor | Adrenergic inhibitor, Norepinephrine reuptake inhibitor, Serotonin reuptake inhibitor | |
| 1477 | -0.12 | cp | bupropion | Dopamine uptake inhibitor | Dopamine uptake inhibitor | |
| 1478 | -0.13 | cp | cilnidipine | Calcium channel blocker | Calcium channel blocker | |
| 1479 | -0.14 | cp | peucedanin | Apoptosis stimulant | Apoptosis stimulant | |
| 1480 | -0.14 | cp | nefazodone | Adrenergic inhibitor | Adrenergic inhibitor, Norepinephrine reuptake inhibitor, Serotonin receptor antagonist, Serotonin reuptake inhibitor | |
| 1481 | -0.14 | cp | NS-1619 | Calcium channel activator | Calcium channel activator | |
| 1482 | -0.14 | cp | cinchonine | P-glycoprotein inhibitor | P-glycoprotein inhibitor | |
| 1483 | -0.14 | cp | nicorandil | Nitric oxide donor | Nitric oxide donor, Potassium channel activator | |
| 1484 | -0.14 | cp | moclobemide | Monoamine oxidase inhibitor | Monoamine oxidase inhibitor | |
| 1485 | -0.14 | cp | tretinoin | Retinoid receptor agonist | Retinoid receptor agonist, Retinoid receptor ligand | |
| 1486 | -0.14 | cp | chlorpromazine | Dopamine receptor antagonist | Dopamine receptor antagonist | |
| 1487 | -0.14 | cp | ondansetron | Serotonin receptor antagonist | Serotonin receptor antagonist | |
| 1488 | -0.15 | cp | 5-methoxytryptamine | Serotonin receptor agonist | Serotonin receptor agonist | |
| 1489 | -0.18 | cp | esomeprazole | ATPase inhibitor | ATPase inhibitor | |
| 1490 | -0.18 | cp | itraconazole | Cytochrome P450 inhibitor | Cytochrome P450 inhibitor | |
| 1491 | -0.18 | cp | PD-169316 | p38 MAPK inhibitor | p38 MAPK inhibitor | |
| 1492 | -0.18 | cp | lamotrigine | Serotonin receptor antagonist | Serotonin receptor antagonist, Sodium channel blocker | |
| 1493 | -0.18 | cp | clomethiazole | GABA receptor antagonist | GABA receptor antagonist, GABA receptor modulator | |
| 1494 | -0.18 | cp | tiaprofenic-acid | Cyclooxygenase inhibitor | Cyclooxygenase inhibitor | |
| 1495 | -0.18 | cp | indirubin | CDK inhibitor | CDK inhibitor | |
| 1496 | -0.18 | cp | fraxetin | Antioxidant | Antioxidant | |
| 1497 | -0.18 | cp | aliskiren | Antihypertensive | Antihypertensive, Peptidase inhibitor, Protease inhibitor, Renin inhibitor | |
| 1498 | -0.18 | cp | thioridazine | Dopamine receptor antagonist | Dopamine receptor antagonist | |
| 1499 | -0.18 | cp | beclometasone | Glucocorticoid receptor agonist | Glucocorticoid receptor agonist, Immunosuppressant | |
| 1500 | -0.18 | cp | arachidonyl-trifluoro-methane | Cytosolic phospholipase inhibitor | Cytosolic phospholipase inhibitor | |
| 1501 | -0.19 | cp | picotamide | Thromboxane receptor antagonist | Thromboxane receptor antagonist, Thromboxane synthase inhibitor | |
| 1502 | -0.2 | cp | PHA-665752 | c-Met inhibitor | c-Met inhibitor | |
| 1503 | -0.2 | cp | mexiletine | Sodium channel blocker | Sodium channel blocker | |
| 1504 | -0.21 | cp | synephrine | Adrenergic receptor agonist | Adrenergic receptor agonist | |
| 1505 | -0.21 | cp | rifabutin | Protein synthesis inhibitor | Protein synthesis inhibitor | |
| 1506 | -0.21 | cp | repaglinide | Insulin secretagogue | Insulin secretagogue | |
| 1507 | -0.21 | cp | nabumetone | Cyclooxygenase inhibitor | Cyclooxygenase inhibitor | |
| 1508 | -0.21 | cp | INCA-6 | Calcineurin inhibitor | Calcineurin inhibitor | |
| 1509 | -0.21 | cp | bumetanide | Solute carrier family member inhibitor | Solute carrier family member inhibitor | |
| 1510 | -0.23 | cp | FGIN-1-27 | Inositol monophosphatase inhibitor | Inositol monophosphatase inhibitor | |
| 1511 | -0.25 | cp | aripiprazole | Serotonin receptor agonist | Serotonin receptor agonist, Serotonin receptor antagonist | |
| 1512 | -0.25 | cp | pyrimethamine | Dihydrofolate reductase inhibitor | Dihydrofolate reductase inhibitor | |
| 1513 | -0.25 | cp | quinapril | ACE inhibitor | ACE inhibitor | |
| 1514 | -0.25 | cp | oxaprozin | Cyclooxygenase inhibitor | Cyclooxygenase inhibitor | |
| 1515 | -0.25 | cp | E-4031 | Potassium channel blocker | Potassium channel blocker | |
| 1516 | -0.25 | cp | AMG-9810 | TRPV antagonist | TRPV antagonist | |
| 1517 | -0.25 | cp | aminoglutethimide | Glucocorticoid receptor antagonist | Glucocorticoid receptor antagonist | |
| 1518 | -0.25 | cp | thiazolopyrimidine | CDC inhibitor | CDC inhibitor | |
| 1519 | -0.25 | cp | navitoclax | BCL inhibitor | BCL inhibitor | |
| 1520 | -0.25 | cp | iso-olomoucine | CDK inhibitor | CDK inhibitor | |
| 1521 | -0.25 | cp | Y-26763 | Potassium channel activator | Potassium channel activator | |
| 1522 | -0.28 | cp | mosapride | Serotonin receptor agonist | Serotonin receptor agonist | |
| 1523 | -0.28 | cp | BRD-K66896231 | Acetylcholinesterase inhibitor | Acetylcholinesterase inhibitor | |
| 1524 | -0.28 | cp | troglitazone | Insulin sensitizer | Insulin sensitizer, PPAR receptor agonist | |
| 1525 | -0.28 | cp | butein | EGFR inhibitor | EGFR inhibitor, SRC inhibitor | |
| 1526 | -0.28 | cp | oxotremorine | Acetylcholine receptor agonist | Acetylcholine receptor agonist, Cholinergic receptor agonist | |
| 1527 | -0.28 | cp | dihydroergotamine | Serotonin receptor agonist | Serotonin receptor agonist | |
| 1528 | -0.28 | cp | chloroxine | Opioid receptor antagonist | Opioid receptor antagonist | |
| 1529 | -0.28 | cp | secnidazole | Acetylcholinesterase inhibitor | Acetylcholinesterase inhibitor, Microtubule inhibitor, Antiprotozoal | |
| 1530 | -0.29 | cp | medetomidine | Adrenergic receptor agonist | Adrenergic receptor agonist | |
| 1531 | -0.32 | cp | trapidil | PDGFR receptor inhibitor | PDGFR receptor inhibitor | |
| 1532 | -0.32 | cp | LY-225910 | CCK receptor antagonist | CCK receptor antagonist | |
| 1533 | -0.32 | cp | aniracetam | Glutamate receptor agonist | Glutamate receptor agonist | |
| 1534 | -0.32 | cp | r(-)-propylnorapomorphine | Dopamine receptor agonist | Dopamine receptor agonist, TRPA1 modulator | |
| 1535 | -0.32 | cp | naftidrofuryl | Adrenergic receptor antagonist | Adrenergic receptor antagonist | |
| 1536 | -0.32 | cp | fluspirilene | Dopamine receptor antagonist | Dopamine receptor antagonist | |
| 1537 | -0.32 | cp | donepezil | Acetylcholinesterase inhibitor | Acetylcholinesterase inhibitor | |
| 1538 | -0.32 | cp | citalopram | Serotonin reuptake inhibitor | Serotonin reuptake inhibitor | |
| 1539 | -0.35 | cp | penciclovir | DNA directed DNA polymerase inhibitor | DNA directed DNA polymerase inhibitor | |
| 1540 | -0.35 | cp | flucytosine | Antifungal | Antifungal | |
| 1541 | -0.35 | cp | BJM-CSC-19 | MEK inhibitor | MEK inhibitor | |
| 1542 | -0.35 | cp | zileuton | Leukotriene inhibitor | Leukotriene inhibitor, Lipoxygenase inhibitor | |
| 1543 | -0.35 | cp | MT-21 | Caspase activator | Caspase activator | |
| 1544 | -0.35 | cp | prednisolone | Glucocorticoid receptor agonist | Glucocorticoid receptor agonist | |
| 1545 | -0.35 | cp | tolazoline | Adrenergic receptor antagonist | Adrenergic receptor antagonist | |
| 1546 | -0.37 | cp | lofepramine | Norepinephrine reuptake inhibitor | Norepinephrine reuptake inhibitor, Serotonin reuptake inhibitor | |
| 1547 | -0.39 | cp | TFMPP | Serotonin receptor agonist | Serotonin receptor agonist, Serotonin receptor antagonist | |
| 1548 | -0.39 | cp | phenethyl-isothiocyanate | Antineoplastic | Antineoplastic | |
| 1549 | -0.39 | cp | BMS-536924 | IGF-1 inhibitor | IGF-1 inhibitor | |
| 1550 | -0.39 | cp | AM-92016 | Potassium channel blocker | Potassium channel blocker | |
| 1551 | -0.39 | cp | piroxicam | Cyclooxygenase inhibitor | Cyclooxygenase inhibitor | |
| 1552 | -0.39 | cp | pergolide | Dopamine receptor agonist | Dopamine receptor agonist | |
| 1553 | -0.39 | cp | naproxol | Anti-inflammatory | Anti-inflammatory | |
| 1554 | -0.39 | cp | KI-8751 | VEGFR inhibitor | VEGFR inhibitor, KIT inhibitor, PDGFR receptor inhibitor | |
| 1555 | -0.39 | cp | imiloxan | Adrenergic receptor antagonist | Adrenergic receptor antagonist | |
| 1556 | -0.39 | cp | timolol | Adrenergic receptor antagonist | Adrenergic receptor antagonist | |
| 1557 | -0.42 | cp | quinine | Hemozoin biocrystallization inhibitor | Hemozoin biocrystallization inhibitor | |
| 1558 | -0.42 | cp | prostaglandin-a1 | HSP inducer | HSP inducer, NFkB pathway inhibitor | |
| 1559 | -0.42 | cp | primaquine | Antimalarial | Antimalarial, DNA inhibitor | |
| 1560 | -0.42 | cp | disopyramide | Sodium channel blocker | Sodium channel blocker | |
| 1561 | -0.42 | cp | ICI-204448 | Opioid receptor agonist | Opioid receptor agonist | |
| 1562 | -0.46 | cp | avrainvillamide-analog-6 | nucleophosmin inhibitor | nucleophosmin inhibitor | |
| 1563 | -0.46 | cp | SB-590885 | RAF inhibitor | RAF inhibitor | |
| 1564 | -0.46 | cp | methyllidocaine | antiarrhythmic medication | antiarrhythmic medication | |
| 1565 | -0.46 | cp | deguelin | NADH-ubiquinone oxidoreductase (Complex I) inhibitor | NADH-ubiquinone oxidoreductase (Complex I) inhibitor | |
| 1566 | -0.46 | cp | calycanthine | GABA release inhibitor | GABA release inhibitor | |
| 1567 | -0.46 | cp | limonin | HIV protease inhibitor | HIV protease inhibitor | |
| 1568 | -0.46 | cp | exemestane | Aromatase inhibitor | Aromatase inhibitor | |
| 1569 | -0.46 | cp | phenazone | Cyclooxygenase inhibitor | Cyclooxygenase inhibitor | |
| 1570 | -0.46 | cp | tolazamide | ATP channel blocker | ATP channel blocker | |
| 1571 | -0.46 | cp | chlorambucil | DNA inhibitor | DNA inhibitor | |
| 1572 | -0.46 | cp | XAV-939 | Tankyrase inhibitor | Tankyrase inhibitor | |
| 1573 | -0.49 | cp | desoxycorticosterone | Mineralocorticoid receptor agonist | Mineralocorticoid receptor agonist | |
| 1574 | -0.49 | cp | trazodone | Adrenergic receptor antagonist | Adrenergic receptor antagonist, Serotonin receptor antagonist, Serotonin reuptake inhibitor | |
| 1575 | -0.49 | cp | mesulergine | Dopamine receptor agonist | Dopamine receptor agonist | |
| 1576 | -0.49 | cp | meclozine | CAR agonist | CAR agonist | |
| 1577 | -0.49 | cp | salmeterol | Adrenergic receptor agonist | Adrenergic receptor agonist | |
| 1578 | -0.49 | cp | nifedipine | Calcium channel blocker | Calcium channel blocker | |
| 1579 | -0.5 | cp | benzo(a)pyrene | Carcinogen | Carcinogen | |
| 1580 | -0.52 | cp | icariin | Phosphodiesterase inhibitor | Phosphodiesterase inhibitor | |
| 1581 | -0.52 | cp | proguanil | Dihydrofolate reductase inhibitor | Dihydrofolate reductase inhibitor | |
| 1582 | -0.53 | cp | relcovaptan | Vasopressin receptor antagonist | Vasopressin receptor antagonist | |
| 1583 | -0.53 | cp | tosyllysyl-chloromethyl-ketone | Chymotrypsin inhibitor | Chymotrypsin inhibitor | |
| 1584 | -0.53 | cp | nefopam | Cyclooxygenase inhibitor | Cyclooxygenase inhibitor | |
| 1585 | -0.53 | cp | hinokitiol | Tyrosinase inhibitor | Tyrosinase inhibitor | |
| 1586 | -0.53 | cp | clobenpropit | Histamine receptor antagonist | Histamine receptor antagonist | |
| 1587 | -0.53 | cp | tyrphostin-AG-18 | EGFR inhibitor | EGFR inhibitor, Tyrosine kinase inhibitor | |
| 1588 | -0.53 | cp | Y-27152 | Potassium channel activator | Potassium channel activator | |
| 1589 | -0.53 | cp | pizotifen | Serotonin receptor antagonist | Serotonin receptor antagonist | |
| 1590 | -0.53 | cp | brazilin | Nitric oxide production inhibitor | Nitric oxide production inhibitor | |
| 1591 | -0.53 | cp | ropinirole | Dopamine receptor agonist | Dopamine receptor agonist | |
| 1592 | -0.53 | cp | AICA-ribonucleotide | AMPK activator | AMPK activator | |
| 1593 | -0.53 | cp | quinethazone | Thiazide diuretic | Thiazide diuretic | |
| 1594 | -0.56 | cp | alosetron | Serotonin receptor antagonist | Serotonin receptor antagonist | |
| 1595 | -0.56 | cp | ZK-93426 | Benzodiazepine receptor antagonist | Benzodiazepine receptor antagonist | |
| 1596 | -0.56 | cp | GS-39783 | GABA receptor modulator | GABA receptor modulator | |
| 1597 | -0.56 | cp | PSB-36 | Adenosine receptor antagonist | Adenosine receptor antagonist | |
| 1598 | -0.6 | cp | tropisetron | Serotonin receptor antagonist | Serotonin receptor antagonist | |
| 1599 | -0.6 | cp | IWR-1-ENDO | PARP inhibitor | PARP inhibitor | |
| 1600 | -0.6 | cp | NSC-632839 | Ubiquitin specific protease inhibitor | Ubiquitin specific protease inhibitor | |
| 1601 | -0.6 | cp | genistein | Tyrosine kinase inhibitor | Tyrosine kinase inhibitor | |
| 1602 | -0.6 | cp | PG-9 | Acetylcholine receptor agonist | Acetylcholine receptor agonist | |
| 1603 | -0.6 | cp | ML-7 | Myosin light chain kinase inhibitor | Myosin light chain kinase inhibitor | |
| 1604 | -0.6 | cp | kynuramine | Aryl hydrocarbon receptor activator | Aryl hydrocarbon receptor activator | |
| 1605 | -0.6 | cp | icosapent | Platelet aggregation inhibitor | Platelet aggregation inhibitor | |
| 1606 | -0.6 | cp | BMS-191011 | Potassium channel activator | Potassium channel activator | |
| 1607 | -0.62 | cp | triamterene | Sodium channel blocker | Sodium channel blocker | |
| 1608 | -0.63 | cp | oxymetholone | Androgen receptor agonist | Androgen receptor agonist | |
| 1609 | -0.63 | cp | cotinine | Nicotine metabolite | Nicotine metabolite | |
| 1610 | -0.63 | cp | proxyfan | Histamine receptor modulator | Histamine receptor modulator | |
| 1611 | -0.63 | cp | eicosatrienoic-acid | Vasodilator | Vasodilator | |
| 1612 | -0.63 | cp | cinanserin | Serotonin receptor antagonist | Serotonin receptor antagonist | |
| 1613 | -0.63 | cp | ambroxol | Sodium channel blocker | Sodium channel blocker | |
| 1614 | -0.63 | cp | tizanidine | Adrenergic receptor agonist | Adrenergic receptor agonist | |
| 1615 | -0.67 | cp | capecitabine | DNA synthesis inhibitor | DNA synthesis inhibitor, Thymidylate synthase inhibitor | |
| 1616 | -0.67 | cp | LY-165163 | Serotonin receptor antagonist | Serotonin receptor antagonist | |
| 1617 | -0.67 | cp | iodophenpropit | Histamine receptor antagonist | Histamine receptor antagonist | |
| 1618 | -0.67 | cp | flumazenil | Benzodiazepine receptor antagonist | Benzodiazepine receptor antagonist | |
| 1619 | -0.67 | cp | BD-1008 | Sigma receptor antagonist | Sigma receptor antagonist | |
| 1620 | -0.67 | cp | diflunisal | Prostanoid receptor antagonist | Prostanoid receptor antagonist | |
| 1621 | -0.68 | cp | megestrol | Progesterone receptor agonist | Progesterone receptor agonist | |
| 1622 | -0.7 | cp | phenothiazine | Dopamine receptor antagonist | Dopamine receptor antagonist | |
| 1623 | -0.7 | cp | fluoropyruvate | Pyruvate dehydrogenase kinase inhibitor | Pyruvate dehydrogenase kinase inhibitor | |
| 1624 | -0.7 | cp | nifurtimox | DNA inhibitor | DNA inhibitor | |
| 1625 | -0.7 | cp | felbamate | Glutamate receptor antagonist | Glutamate receptor antagonist | |
| 1626 | -0.7 | cp | pilocarpine | Acetylcholine receptor agonist | Acetylcholine receptor agonist | |
| 1627 | -0.7 | cp | RS-79948 | Adrenergic receptor antagonist | Adrenergic receptor antagonist | |
| 1628 | -0.74 | cp | syrosingopine | Vesicular monoamine transporter inhibitor | Vesicular monoamine transporter inhibitor | |
| 1629 | -0.74 | cp | SSR-69071 | Leukocyte elastase inhibitor | Leukocyte elastase inhibitor | |
| 1630 | -0.74 | cp | PCA-4248 | Platelet activating factor receptor antagonist | Platelet activating factor receptor antagonist | |
| 1631 | -0.74 | cp | SA-1478088 | -666 | -666 |  |
| 1632 | -0.74 | cp | fursultiamine | Vitamin B | Vitamin B | |
| 1633 | -0.74 | cp | diethylcarbamazine | Lipoxygenase inhibitor | Lipoxygenase inhibitor | |
| 1634 | -0.74 | cp | cimaterol | Adrenergic receptor agonist | Adrenergic receptor agonist | |
| 1635 | -0.74 | cp | nortriptyline | Tricyclic antidepressant | Tricyclic antidepressant | |
| 1636 | -0.74 | cp | metrizamide | Radiopaque medium | Radiopaque medium | |
| 1637 | -0.78 | cp | azelastine | Histamine receptor antagonist | Histamine receptor antagonist | |
| 1638 | -0.78 | cp | cytosporone-b | NUR77 receptor agonist | NUR77 receptor agonist | |
| 1639 | -0.78 | cp | tubaic-acid | Mitochondrial complex I inhibitor | Mitochondrial complex I inhibitor, NADH-ubiquinone oxidoreductase (Complex I) inhibitor | |
| 1640 | -0.78 | cp | MG-132 | Proteasome inhibitor | Proteasome inhibitor | |
| 1641 | -0.78 | cp | arctigenin | MEK inhibitor | MEK inhibitor | |
| 1642 | -0.78 | cp | SRC-kinase-inhibitor-I | SRC inhibitor | SRC inhibitor | |
| 1643 | -0.78 | cp | oligomycin-a | ATP synthase inhibitor | ATP synthase inhibitor, ATPase inhibitor | |
| 1644 | -0.78 | cp | RO-28-1675 | Glucokinase activator | Glucokinase activator | |
| 1645 | -0.78 | cp | helveticoside | ATPase inhibitor | ATPase inhibitor | |
| 1646 | -0.81 | cp | apafant | Platelet activating factor receptor antagonist | Platelet activating factor receptor antagonist | |
| 1647 | -0.81 | cp | RO-15-4513 | GABA benzodiazepine site receptor inverse agonist | GABA benzodiazepine site receptor inverse agonist | |
| 1648 | -0.81 | cp | roquinimex | Angiogenesis inhibitor | Angiogenesis inhibitor, TNF production inhibitor | |
| 1649 | -0.81 | cp | gavestinel | Glutamate receptor antagonist | Glutamate receptor antagonist | |
| 1650 | -0.81 | cp | econazole | Bacterial cell wall synthesis inhibitor | Bacterial cell wall synthesis inhibitor, Lanosterol demethylase inhibitor, Sterol demethylase inhibitor | |
| 1651 | -0.81 | cp | trequinsin | Phosphodiesterase inhibitor | Phosphodiesterase inhibitor | |
| 1652 | -0.81 | cp | alaproclate | Serotonin receptor antagonist | Serotonin receptor antagonist | |
| 1653 | -0.81 | cp | methocarbamol | Muscle relaxant | Muscle relaxant | |
| 1654 | -0.82 | cp | buflomedil | Adrenergic receptor antagonist | Adrenergic receptor antagonist, Calcium channel blocker | |
| 1655 | -0.83 | cp | L-161982 | Prostanoid receptor antagonist | Prostanoid receptor antagonist, Angiotensin receptor antagonist | |
| 1656 | -0.85 | cp | aminoindazole | Ionophore | Ionophore | |
| 1657 | -0.85 | cp | talniflumate | Cyclooxygenase inhibitor | Cyclooxygenase inhibitor | |
| 1658 | -0.85 | cp | thiothixene | Dopamine receptor antagonist | Dopamine receptor antagonist | |
| 1659 | -0.85 | cp | fexaramine | FXR agonist | FXR agonist | |
| 1660 | -0.85 | cp | molsidomine | Guanylyl cyclase activator | Guanylyl cyclase activator | |
| 1661 | -0.85 | cp | fludarabine | DNA synthesis inhibitor | DNA synthesis inhibitor, DNA repair enzyme inhibitor, Purine antagonist | |
| 1662 | -0.85 | cp | glibenclamide | Sulfonylurea | Sulfonylurea | |
| 1663 | -0.88 | cp | papaverine | Phosphodiesterase inhibitor | Phosphodiesterase inhibitor | |
| 1664 | -0.88 | cp | RO-16-6941 | Monoamine oxidase inhibitor | Monoamine oxidase inhibitor | |
| 1665 | -0.88 | cp | brinzolamide | Carbonic anhydrase inhibitor | Carbonic anhydrase inhibitor | |
| 1666 | -0.92 | cp | azauridine | Antiviral | Antiviral | |
| 1667 | -0.92 | cp | tranilast | Angiogenesis inhibitor | Angiogenesis inhibitor | |
| 1668 | -0.92 | cp | SC-560 | Cyclooxygenase inhibitor | Cyclooxygenase inhibitor | |
| 1669 | -0.92 | cp | O-2050 | Cannabinoid receptor antagonist | Cannabinoid receptor antagonist | |
| 1670 | -0.92 | cp | imperatorin | CDK inhibitor | CDK inhibitor | |
| 1671 | -0.92 | cp | BRL-54443 | Serotonin receptor agonist | Serotonin receptor agonist | |
| 1672 | -0.92 | cp | auraptene | Nitric oxide production inhibitor | Nitric oxide production inhibitor | |
| 1673 | -0.92 | cp | rhapontin | Apoptosis stimulant | Apoptosis stimulant | |
| 1674 | -0.93 | cp | methyl-angolensate | Apoptosis inhibitor | Apoptosis inhibitor | |
| 1675 | -0.94 | cp | SU-6656 | SRC inhibitor | SRC inhibitor | |
| 1676 | -0.95 | cp | perhexiline | Carnitine palmitoyltransferase inhibitor | Carnitine palmitoyltransferase inhibitor | |
| 1677 | -0.95 | cp | tenidap | Cyclooxygenase inhibitor | Cyclooxygenase inhibitor | |
| 1678 | -0.95 | cp | SC-9 | Protein tyrosine kinase activator | Protein tyrosine kinase activator | |
| 1679 | -0.95 | cp | RO-10-5824 | Dopamine receptor agonist | Dopamine receptor agonist | |
| 1680 | -0.95 | cp | simvastatin | HMGCR inhibitor | HMGCR inhibitor | |
| 1681 | -0.95 | cp | BRD-K88742110 | HDAC inhibitor | HDAC inhibitor | |
| 1682 | -0.95 | cp | eticlopride | Dopamine receptor antagonist | Dopamine receptor antagonist | |
| 1683 | -0.95 | cp | imiquimod | TLR agonist | TLR agonist, Interferon inducer | |
| 1684 | -0.97 | cp | devazepide | CCK receptor antagonist | CCK receptor antagonist | |
| 1685 | -0.99 | cp | calmidazolium | Calcium channel blocker | Calcium channel blocker, Calmodulin antagonist | |
| 1686 | -0.99 | cp | GANT-61 | GLI antagonist | GLI antagonist | |
| 1687 | -0.99 | cp | thioproperazine | Dopamine receptor antagonist | Dopamine receptor antagonist | |
| 1688 | -0.99 | cp | SB-334867 | Orexin receptor antagonist | Orexin receptor antagonist | |
| 1689 | -0.99 | cp | GW-441756 | Growth factor receptor inhibitor | Growth factor receptor inhibitor | |
| 1690 | -0.99 | cp | epirizole | Cyclooxygenase inhibitor | Cyclooxygenase inhibitor | |
| 1691 | -0.99 | cp | chaetocin | Histone lysine methyltransferase inhibitor | Histone lysine methyltransferase inhibitor | |
| 1692 | -1 | cp | KU-C103443N | CDC inhibitor | CDC inhibitor, Rho associated kinase inhibitor | |
| 1693 | -1.01 | cp | lithocholic-acid | FXR antagonist | FXR antagonist, MDM inhibitor, PXR ligand, Tyrosine phosphatase inhibitor, Vitamin D receptor agonist, Vitamin D receptor ligand | |
| 1694 | -1.01 | cp | scoulerine | Adrenergic receptor antagonist | Adrenergic receptor antagonist, GABA receptor antagonist, Serotonin receptor antagonist | |
| 1695 | -1.01 | cp | AG-99 | Tyrosine kinase inhibitor | Tyrosine kinase inhibitor | |
| 1696 | -1.02 | cp | n-(3-acetamidophenyl)-3-chlorobenzamide | Glutamate receptor antagonist | Glutamate receptor antagonist | |
| 1697 | -1.02 | cp | terguride | Dopamine receptor agonist | Dopamine receptor agonist, Serotonin receptor antagonist | |
| 1698 | -1.04 | cp | ibuprofen | Cyclooxygenase inhibitor | Cyclooxygenase inhibitor, NFkB pathway inhibitor | |
| 1699 | -1.06 | cp | nitrendipine | Calcium channel blocker | Calcium channel blocker | |
| 1700 | -1.06 | cp | MDL-72832 | Serotonin receptor agonist | Serotonin receptor agonist | |
| 1701 | -1.06 | cp | LY-341495 | Glutamate receptor antagonist | Glutamate receptor antagonist | |
| 1702 | -1.06 | cp | lonidamine | Glucokinase inhibitor | Glucokinase inhibitor | |
| 1703 | -1.06 | cp | IAA-94 | Chloride channel blocker | Chloride channel blocker | |
| 1704 | -1.06 | cp | GW-583340 | EGFR inhibitor | EGFR inhibitor, ErbB2 inhibitor, Receptor tyrosine protein kinase inhibitor | |
| 1705 | -1.06 | cp | FCCP | Mitochondrial oxidative phosphorylation uncoupler | Mitochondrial oxidative phosphorylation uncoupler | |
| 1706 | -1.06 | cp | farnesylthioacetic-acid | Inhibitor of methyl esterification of farnesylated proteins | Inhibitor of methyl esterification of farnesylated proteins | |
| 1707 | -1.06 | cp | famciclovir | DNA polymerase inhibitor | DNA polymerase inhibitor | |
| 1708 | -1.07 | cp | famotidine | Histamine receptor antagonist | Histamine receptor antagonist | |
| 1709 | -1.09 | cp | nifekalant | Potassium channel blocker | Potassium channel blocker | |
| 1710 | -1.09 | cp | methylene-blue | Guanylyl cyclase inhibitor | Guanylyl cyclase inhibitor, Monoamine oxidase inhibitor, Nitric oxide production inhibitor, Tau aggregation inhibitor | |
| 1711 | -1.09 | cp | SJ-172550 | MDM inhibitor | MDM inhibitor | |
| 1712 | -1.09 | cp | levcromakalim | Potassium channel activator | Potassium channel activator | |
| 1713 | -1.09 | cp | gabexate | Serine protease inhibitor | Serine protease inhibitor | |
| 1714 | -1.09 | cp | cilastatin | Dehydropeptidase inhibitor | Dehydropeptidase inhibitor | |
| 1715 | -1.09 | cp | BCL2-inhibitor | BCL inhibitor | BCL inhibitor | |
| 1716 | -1.09 | cp | triamcinolone | Glucocorticoid receptor agonist | Glucocorticoid receptor agonist | |
| 1717 | -1.09 | cp | carvedilol | Adrenergic receptor antagonist | Adrenergic receptor antagonist | |
| 1718 | -1.09 | cp | amoxapine | Norepinephrine reuptake inhibitor | Norepinephrine reuptake inhibitor | |
| 1719 | -1.1 | cp | TCS-359 | FLT3 inhibitor | FLT3 inhibitor | |
| 1720 | -1.13 | cp | ebelactone-b | Lipase inhibitor | Lipase inhibitor | |
| 1721 | -1.13 | cp | isoquercetin | Aldose reductase inhibitor | Aldose reductase inhibitor | |
| 1722 | -1.13 | cp | SANT-1 | Smoothened receptor antagonist | Smoothened receptor antagonist | |
| 1723 | -1.13 | cp | linoleamide | ACAT inhibitor | ACAT inhibitor | |
| 1724 | -1.13 | cp | CGP-13501 | GABA receptor modulator | GABA receptor modulator | |
| 1725 | -1.15 | cp | rufloxacin | Bacterial DNA gyrase inhibitor | Bacterial DNA gyrase inhibitor | |
| 1726 | -1.15 | cp | hydroxyfasudil | Rho associated kinase inhibitor | Rho associated kinase inhibitor | |
| 1727 | -1.16 | cp | orlistat | Lipase inhibitor | Lipase inhibitor | |
| 1728 | -1.16 | cp | HLI-373 | MDM inhibitor | MDM inhibitor | |
| 1729 | -1.16 | cp | tyrphostin-47 | EGFR inhibitor | EGFR inhibitor | |
| 1730 | -1.16 | cp | RG-14620 | EGFR inhibitor | EGFR inhibitor | |
| 1731 | -1.16 | cp | ergometrine | Adrenergic receptor agonist | Adrenergic receptor agonist, Serotonin receptor agonist | |
| 1732 | -1.16 | cp | mebendazole | Tubulin inhibitor | Tubulin inhibitor | |
| 1733 | -1.16 | cp | LY-2183240 | FAAH inhibitor | FAAH inhibitor | |
| 1734 | -1.18 | cp | practolol | Adrenergic receptor antagonist | Adrenergic receptor antagonist | |
| 1735 | -1.2 | cp | formestane | Aromatase inhibitor | Aromatase inhibitor | |
| 1736 | -1.2 | cp | KIN001-244 | Phosphoinositide dependent kinase inhibitor | Phosphoinositide dependent kinase inhibitor | |
| 1737 | -1.2 | cp | neratinib | EGFR inhibitor | EGFR inhibitor | |
| 1738 | -1.22 | cp | KB-R7943 | Sodium/calcium exchange inhibitor | Sodium/calcium exchange inhibitor | |
| 1739 | -1.23 | cp | dephostatin | Tyrosine phosphatase inhibitor | Tyrosine phosphatase inhibitor | |
| 1740 | -1.23 | cp | chlorphenamine | Histamine receptor antagonist | Histamine receptor antagonist | |
| 1741 | -1.27 | cp | linifanib | PDGFR receptor inhibitor | PDGFR receptor inhibitor, VEGFR inhibitor | |
| 1742 | -1.27 | cp | GR-206 | Aryl hydrocarbon receptor ligand | Aryl hydrocarbon receptor ligand | |
| 1743 | -1.27 | cp | 2-aminopurine | Serine/threonine kinase inhibitor | Serine/threonine kinase inhibitor | |
| 1744 | -1.27 | cp | capsazepine | TRPV agonist | TRPV agonist | |
| 1745 | -1.27 | cp | DPO-1 | Potassium channel blocker | Potassium channel blocker | |
| 1746 | -1.27 | cp | PTB1 | AMPK activator | AMPK activator | |
| 1747 | -1.3 | cp | SB-203580 | p38 MAPK inhibitor | p38 MAPK inhibitor | |
| 1748 | -1.3 | cp | SB-225002 | CC chemokine receptor antagonist | CC chemokine receptor antagonist | |
| 1749 | -1.3 | cp | dioxybenzone | Topical sunscreen agent | Topical sunscreen agent | |
| 1750 | -1.33 | cp | GW-4064 | FXR agonist | FXR agonist | |
| 1751 | -1.34 | cp | pargyline | Monoamine oxidase inhibitor | Monoamine oxidase inhibitor | |
| 1752 | -1.34 | cp | n-arachidonyl-GABA | cannabinoid receptor agonist | cannabinoid receptor agonist | |
| 1753 | -1.35 | cp | hypericin | Tyrosine kinase inhibitor | Tyrosine kinase inhibitor | |
| 1754 | -1.37 | cp | hispidin | PKC inhibitor | PKC inhibitor | |
| 1755 | -1.41 | cp | tamibarotene | Retinoid receptor agonist | Retinoid receptor agonist | |
| 1756 | -1.41 | cp | fluticasone | Glucocorticoid receptor agonist | Glucocorticoid receptor agonist | |
| 1757 | -1.41 | cp | diazepam | Benzodiazepine receptor agonist | Benzodiazepine receptor agonist | |
| 1758 | -1.41 | cp | nor-binaltorphimine | Opioid receptor antagonist | Opioid receptor antagonist | |
| 1759 | -1.41 | cp | erastin | Ion channel antagonist | Ion channel antagonist | |
| 1760 | -1.43 | cp | chelidonine | Tubulin inhibitor | Tubulin inhibitor | |
| 1761 | -1.44 | cp | ginsenoside | Steroid hormone receptor agonist | Steroid hormone receptor agonist | |
| 1762 | -1.44 | cp | pirenzepine | Acetylcholine receptor antagonist | Acetylcholine receptor antagonist | |
| 1763 | -1.44 | cp | fenretinide | Apoptosis stimulant | Apoptosis stimulant, Retinoid receptor agonist | |
| 1764 | -1.44 | cp | tacedinaline | HDAC inhibitor | HDAC inhibitor | |
| 1765 | -1.44 | cp | erbstatin-analog | EGFR inhibitor | EGFR inhibitor | |
| 1766 | -1.48 | cp | prostaglandin | Prostanoid receptor antagonist | Prostanoid receptor antagonist | |
| 1767 | -1.48 | cp | argatroban | Thrombin inhibitor | Thrombin inhibitor | |
| 1768 | -1.48 | cp | sulpiride | Dopamine receptor antagonist | Dopamine receptor antagonist | |
| 1769 | -1.48 | cp | rimexolone | Glucocorticoid receptor agonist | Glucocorticoid receptor agonist | |
| 1770 | -1.48 | cp | L-655240 | Thromboxane receptor antagonist | Thromboxane receptor antagonist | |
| 1771 | -1.48 | cp | hydrastinine | Haemostatic agent | Haemostatic agent | |
| 1772 | -1.49 | cp | lacidipine | Calcium channel blocker | Calcium channel blocker | |
| 1773 | -1.52 | cp | lidocaine | Histamine receptor agonist | Histamine receptor agonist | |
| 1774 | -1.55 | cp | GW-0742 | PPAR receptor agonist | PPAR receptor agonist, Insulin sensitizer | |
| 1775 | -1.56 | cp | benzanthrone | Aromatic hydrocarbon derivative | Aromatic hydrocarbon derivative | |
| 1776 | -1.57 | cp | chlortetracycline | Protein synthesis inhibitor | Protein synthesis inhibitor | |
| 1777 | -1.58 | cp | mead-ethanolamide | Cannabinoid receptor agonist | Cannabinoid receptor agonist | |
| 1778 | -1.59 | cp | curcumin | Cyclooxygenase inhibitor | Cyclooxygenase inhibitor, Histone acetyltransferase inhibitor, Lipoxygenase inhibitor, NFkB pathway inhibitor | |
| 1779 | -1.59 | cp | 7-nitroindazole | nitric oxide synthase inhibitor | nitric oxide synthase inhibitor | |
| 1780 | -1.59 | cp | RWJ-21757 | TLR agonist | TLR agonist | |
| 1781 | -1.59 | cp | nizatidine | Histamine receptor antagonist | Histamine receptor antagonist | |
| 1782 | -1.59 | cp | piceid | Glucosidase inhibitor | Glucosidase inhibitor | |
| 1783 | -1.62 | cp | PKCbeta-inhibitor | PKC inhibitor | PKC inhibitor | |
| 1784 | -1.62 | cp | fenoldopam | Dopamine receptor agonist | Dopamine receptor agonist | |
| 1785 | -1.62 | cp | sertaconazole | Sterol demethylase inhibitor | Sterol demethylase inhibitor | |
| 1786 | -1.63 | cp | morphothebaine | Adrenergic receptor antagonist | Adrenergic receptor antagonist | |
| 1787 | -1.64 | cp | NM-PP1 | Mutant kinase inhibitor | Mutant kinase inhibitor | |
| 1788 | -1.66 | cp | acamprosate | Glutamate receptor antagonist | Glutamate receptor antagonist | |
| 1789 | -1.66 | cp | ruxolitinib | JAK inhibitor | JAK inhibitor | |
| 1790 | -1.66 | cp | diethylstilbestrol | Estrogen receptor agonist | Estrogen receptor agonist | |
| 1791 | -1.69 | cp | NF-449 | Purinergic receptor antagonist | Purinergic receptor antagonist | |
| 1792 | -1.69 | cp | alpha-estradiol | Estrogen receptor agonist | Estrogen receptor agonist | |
| 1793 | -1.69 | cp | LFM-A13 | BTK inhibitor | BTK inhibitor | |
| 1794 | -1.69 | cp | AG-370 | PDGFR receptor inhibitor | PDGFR receptor inhibitor | |
| 1795 | -1.69 | cp | clofibrate | PPAR receptor agonist | PPAR receptor agonist | |
| 1796 | -1.73 | cp | fluvastatin | HMGCR inhibitor | HMGCR inhibitor | |
| 1797 | -1.75 | cp | articaine | Local anesthetic | Local anesthetic | |
| 1798 | -1.76 | cp | WZ-3146 | EGFR inhibitor | EGFR inhibitor | |
| 1799 | -1.76 | cp | GW-311616 | Leukocyte elastase inhibitor | Leukocyte elastase inhibitor | |
| 1800 | -1.76 | cp | altanserin | Serotonin receptor antagonist | Serotonin receptor antagonist | |
| 1801 | -1.78 | cp | atorvastatin | HMGCR inhibitor | HMGCR inhibitor | |
| 1802 | -1.8 | cp | penfluridol | T-type calcium channel blocker | T-type calcium channel blocker | |
| 1803 | -1.8 | cp | DPN | Estrogen receptor agonist | Estrogen receptor agonist | |
| 1804 | -1.83 | cp | tegafur | Thymidylate synthase inhibitor | Thymidylate synthase inhibitor | |
| 1805 | -1.83 | cp | U-99194 | Dopamine receptor antagonist | Dopamine receptor antagonist | |
| 1806 | -1.83 | cp | P-1075 | ATP channel activator | ATP channel activator, Potassium channel activator | |
| 1807 | -1.83 | cp | CGP-55845 | GABA receptor antagonist | GABA receptor antagonist | |
| 1808 | -1.83 | cp | RITA | MDM inhibitor | MDM inhibitor | |
| 1809 | -1.85 | cp | aprepitant | Tachykinin antagonist | Tachykinin antagonist | |
| 1810 | -1.87 | cp | dexbrompheniramine | Histamine receptor antagonist | Histamine receptor antagonist | |
| 1811 | -1.87 | cp | BRL-37344 | Adrenergic receptor agonist | Adrenergic receptor agonist | |
| 1812 | -1.88 | cp | mestinon | Cholinesterase inhibitor | Cholinesterase inhibitor | |
| 1813 | -1.9 | cp | nisoldipine | Calcium channel blocker | Calcium channel blocker | |
| 1814 | -1.9 | cp | SQ-22536 | Adenylyl cyclase inhibitor | Adenylyl cyclase inhibitor | |
| 1815 | -1.9 | cp | decafluorobutane | Contrast agent | Contrast agent | |
| 1816 | -1.9 | cp | carmoxirole | Dopamine receptor agonist | Dopamine receptor agonist | |
| 1817 | -1.9 | cp | bezafibrate | PPAR receptor agonist | PPAR receptor agonist | |
| 1818 | -1.91 | cp | proscillaridin | ATPase inhibitor | ATPase inhibitor | |
| 1819 | -1.91 | cp | NBQX | Glutamate receptor antagonist | Glutamate receptor antagonist | |
| 1820 | -1.92 | cp | estrone | Estrogen receptor agonist | Estrogen receptor agonist, Estrogenic hormone | |
| 1821 | -1.97 | cp | 5-nonyloxytryptamine | Serotonin receptor agonist | Serotonin receptor agonist | |
| 1822 | -1.97 | cp | pinacidil | ATP channel activator | ATP channel activator, Potassium channel activator | |
| 1823 | -1.97 | cp | cyproterone | Androgen receptor antagonist | Androgen receptor antagonist, Progesterone receptor agonist, Testosterone receptor antagonist | |
| 1824 | -1.97 | cp | sotalol | Adrenergic receptor antagonist | Adrenergic receptor antagonist | |
| 1825 | -2.01 | cp | MDM2-inhibitor | MDM inhibitor | MDM inhibitor | |
| 1826 | -2.01 | cp | oxybuprocaine | Local anesthetic | Local anesthetic | |
| 1827 | -2.01 | cp | sunitinib | FLT3 inhibitor | FLT3 inhibitor, KIT inhibitor, PDGFR receptor inhibitor, RET tyrosine kinase inhibitor, VEGFR inhibitor | |
| 1828 | -2.03 | cp | NTNCB | Neuropeptide receptor antagonist | Neuropeptide receptor antagonist | |
| 1829 | -2.04 | cp | wiskostatin | Neural Wiskott-Aldrich syndrome protein inhibitor | Neural Wiskott-Aldrich syndrome protein inhibitor | |
| 1830 | -2.04 | cp | desoxycortone | Mineralocorticoid receptor agonist | Mineralocorticoid receptor agonist | |
| 1831 | -2.04 | cp | PSB-06126 | NTPDase inhibitor | NTPDase inhibitor | |
| 1832 | -2.04 | cp | molindone | Dopamine receptor antagonist | Dopamine receptor antagonist | |
| 1833 | -2.06 | cp | rifaximin | RNA synthesis inhibitor | RNA synthesis inhibitor | |
| 1834 | -2.08 | cp | LY-288513 | CCK receptor antagonist | CCK receptor antagonist | |
| 1835 | -2.08 | cp | L-368899 | Oxytocin receptor antagonist | Oxytocin receptor antagonist | |
| 1836 | -2.08 | cp | cyclopenthiazide | Thiazide diuretic | Thiazide diuretic | |
| 1837 | -2.11 | cp | prothionamide | Mycobacterium tuberculosis enoyl-[acyl-carrier-protein] reductase [NADH] (inhA) inhibitor | Mycobacterium tuberculosis enoyl-[acyl-carrier-protein] reductase [NADH] (inhA) inhibitor | |
| 1838 | -2.11 | cp | NECA | Adenosine receptor agonist | Adenosine receptor agonist | |
| 1839 | -2.11 | cp | hexamethyleneamiloride | Sodium/hydrogen antiport inhibitor | Sodium/hydrogen antiport inhibitor | |
| 1840 | -2.11 | cp | CITCO | CAR agonist | CAR agonist | |
| 1841 | -2.18 | cp | pifithrin | Interleukin receptor antagonist | Interleukin receptor antagonist | |
| 1842 | -2.18 | cp | isoreserpine | Vesicular monoamine transporter inhibitor | Vesicular monoamine transporter inhibitor | |
| 1843 | -2.18 | cp | perphenazine | Dopamine receptor antagonist | Dopamine receptor antagonist | |
| 1844 | -2.19 | cp | atovaquone | Mitochondrial electron transport inhibitor | Mitochondrial electron transport inhibitor | |
| 1845 | -2.22 | cp | serdemetan | MDM inhibitor | MDM inhibitor | |
| 1846 | -2.22 | cp | medrysone | Glucocorticoid receptor agonist | Glucocorticoid receptor agonist | |
| 1847 | -2.22 | cp | brimonidine | Adrenergic receptor agonist | Adrenergic receptor agonist | |
| 1848 | -2.23 | cp | mephenytoin | Hydantoin antiepileptic | Hydantoin antiepileptic | |
| 1849 | -2.24 | cp | DPPE | Histamine receptor antagonist | Histamine receptor antagonist | |
| 1850 | -2.26 | cp | pefloxacin | Bacterial DNA gyrase inhibitor | Bacterial DNA gyrase inhibitor | |
| 1851 | -2.26 | cp | lisuride | Dopamine receptor agonist | Dopamine receptor agonist | |
| 1852 | -2.27 | cp | emodic-acid | Laxative | Laxative |  |
| 1853 | -2.29 | cp | androstenedione | Cytochrome P450 inhibitor | Cytochrome P450 inhibitor | |
| 1854 | -2.3 | cp | pazopanib | KIT inhibitor | KIT inhibitor, PDGFR receptor inhibitor, VEGFR inhibitor | |
| 1855 | -2.33 | cp | ICI-199441 | Opioid receptor agonist | Opioid receptor agonist | |
| 1856 | -2.33 | cp | flufenamic-acid | Chloride channel blocker | Chloride channel blocker | |
| 1857 | -2.33 | cp | epitestosterone | Inactive testosterone analog | Inactive testosterone analog | |
| 1858 | -2.33 | cp | thalidomide | TNF production inhibitor | TNF production inhibitor | |
| 1859 | -2.36 | cp | carteolol | Adrenergic receptor antagonist | Adrenergic receptor antagonist | |
| 1860 | -2.4 | cp | thioperamide | Histamine receptor antagonist | Histamine receptor antagonist | |
| 1861 | -2.43 | cp | toltrazuril | Antiprotozoal | Antiprotozoal | |
| 1862 | -2.43 | cp | RK-682 | Tyrosine phosphatase inhibitor | Tyrosine phosphatase inhibitor | |
| 1863 | -2.43 | cp | spironolactone | Mineralocorticoid receptor antagonist | Mineralocorticoid receptor antagonist | |
| 1864 | -2.47 | cp | levofloxacin | Bacterial DNA gyrase inhibitor | Bacterial DNA gyrase inhibitor | |
| 1865 | -2.47 | cp | BRD-K64835161 | -666 | -666 |  |
| 1866 | -2.47 | cp | VUF-5681 | Histamine receptor antagonist | Histamine receptor antagonist | |
| 1867 | -2.47 | cp | cyclazosin | Adrenergic receptor antagonist | Adrenergic receptor antagonist | |
| 1868 | -2.48 | cp | KN-93 | Calcium-calmodulin dependent protein kinase inhibitor | Calcium-calmodulin dependent protein kinase inhibitor | |
| 1869 | -2.48 | cp | phensuximide | Succinimide antiepileptic | Succinimide antiepileptic | |
| 1870 | -2.5 | cp | alpha-linolenic-acid | Omega 3 fatty acid stimulant | Omega 3 fatty acid stimulant | |
| 1871 | -2.5 | cp | quinpirole | Dopamine receptor agonist | Dopamine receptor agonist | |
| 1872 | -2.5 | cp | iloprost | Platelet aggregation inhibitor | Platelet aggregation inhibitor, Prostanoid receptor agonist | |
| 1873 | -2.54 | cp | lansoprazole | ATPase inhibitor | ATPase inhibitor | |
| 1874 | -2.57 | cp | guanfacine | Adrenergic receptor agonist | Adrenergic receptor agonist | |
| 1875 | -2.57 | cp | FPL-64176 | Calcium channel activator | Calcium channel activator | |
| 1876 | -2.61 | cp | corticosterone | mineralocorticoid receptor agonist | mineralocorticoid receptor agonist | |
| 1877 | -2.61 | cp | SB-415286 | Glycogen synthase kinase inhibitor | Glycogen synthase kinase inhibitor | |
| 1878 | -2.61 | cp | lobaric-acid | Tyrosine phosphatase inhibitor | Tyrosine phosphatase inhibitor | |
| 1879 | -2.61 | cp | acyclovir | DNA polymerase inhibitor | DNA polymerase inhibitor | |
| 1880 | -2.63 | cp | cyclopentolate | Acetylcholine receptor antagonist | Acetylcholine receptor antagonist | |
| 1881 | -2.64 | cp | granisetron | Serotonin receptor antagonist | Serotonin receptor antagonist | |
| 1882 | -2.66 | cp | harpagoside | Acetylcholinesterase inhibitor | Acetylcholinesterase inhibitor | |
| 1883 | -2.66 | cp | baccatin-III | Paclitaxel precursor | Paclitaxel precursor | |
| 1884 | -2.68 | cp | ryanodine | Calcium channel blocker | Calcium channel blocker | |
| 1885 | -2.68 | cp | lobelanidine | Acetylcholine receptor antagonist | Acetylcholine receptor antagonist, Dopamine receptor modulator, Opioid receptor antagonist, Vesicular monoamine transporter ligand | |
| 1886 | -2.68 | cp | KN-62 | Calcium-calmodulin dependent protein kinase inhibitor | Calcium-calmodulin dependent protein kinase inhibitor, Purinergic receptor antagonist | |
| 1887 | -2.68 | cp | D-4476 | TGF beta receptor inhibitor | TGF beta receptor inhibitor | |
| 1888 | -2.71 | cp | olomoucine | CDK inhibitor | CDK inhibitor | |
| 1889 | -2.71 | cp | naftifine | Fungal squalene epoxidase inhibitor | Fungal squalene epoxidase inhibitor | |
| 1890 | -2.71 | cp | FTase-inhibitor-B581 | Farnesyltransferase inhibitor | Farnesyltransferase inhibitor | |
| 1891 | -2.71 | cp | pemoline | Dopamine receptor agonist | Dopamine receptor agonist | |
| 1892 | -2.73 | cp | PIT | Purinergic receptor antagonist | Purinergic receptor antagonist | |
| 1893 | -2.74 | cp | L-803087 | Somatostatin receptor agonist | Somatostatin receptor agonist | |
| 1894 | -2.75 | cp | ketoconazole | Sterol demethylase inhibitor | Sterol demethylase inhibitor | |
| 1895 | -2.75 | cp | hesperidin | Flavanone glycoside | Flavanone glycoside | |
| 1896 | -2.75 | cp | chloroquine | Antimalarial | Antimalarial | |
| 1897 | -2.78 | cp | GW-501516 | PPAR receptor agonist | PPAR receptor agonist, Insulin sensitizer | |
| 1898 | -2.82 | cp | ebselen | H+/K+-ATPase inhibitor | H+/K+-ATPase inhibitor, Cyclooxygenase inhibitor, Glutathione peroxidase agonist, Nitric oxide synthase inhibitor | |
| 1899 | -2.82 | cp | VU-0400195-3 | Glutamate receptor modulator | Glutamate receptor modulator | |
| 1900 | -2.84 | cp | cefatrizine | Bacterial cell wall synthesis inhibitor | Bacterial cell wall synthesis inhibitor | |
| 1901 | -2.88 | cp | tetryzoline | Adrenergic receptor agonist | Adrenergic receptor agonist | |
| 1902 | -2.89 | cp | amlexanox | Histamine receptor modulator | Histamine receptor modulator | |
| 1903 | -2.92 | cp | isogedunin | HSP inhibitor | HSP inhibitor | |
| 1904 | -2.93 | cp | EI-273 | PKC inhibitor | PKC inhibitor | |
| 1905 | -3 | cp | BML-ST330 | Phospholipase inhibitor | Phospholipase inhibitor | |
| 1906 | -3 | cp | roscovitine | CDK inhibitor | CDK inhibitor | |
| 1907 | -3.01 | cp | CCMQ | Inhibitor of the binding of homoquinolinic acid to non-NMDA sensitive sites | Inhibitor of the binding of homoquinolinic acid to non-NMDA sensitive sites | |
| 1908 | -3.02 | cp | aristolochic-acid | Phospholipase inhibitor | Phospholipase inhibitor | |
| 1909 | -3.02 | cp | AS-605240 | PI3K inhibitor | PI3K inhibitor | |
| 1910 | -3.03 | cp | lylamine | Cannabinoid receptor agonist | Cannabinoid receptor agonist | |
| 1911 | -3.07 | cp | osthol | Calcium channel blocker | Calcium channel blocker | |
| 1912 | -3.1 | cp | calcitriol | Vitamin D receptor agonist | Vitamin D receptor agonist | |
| 1913 | -3.1 | cp | BH3I-1 | BCL inhibitor | BCL inhibitor | |
| 1914 | -3.12 | cp | TTNPB | Retinoid receptor agonist | Retinoid receptor agonist | |
| 1915 | -3.14 | cp | loreclezole | GABA receptor agonist | GABA receptor agonist | |
| 1916 | -3.14 | cp | doxepin | Histamine receptor antagonist | Histamine receptor antagonist | |
| 1917 | -3.16 | cp | NSC-119889 | Protein synthesis inhibitor | Protein synthesis inhibitor | |
| 1918 | -3.18 | cp | warfarin | Vitamin K antagonist | Vitamin K antagonist | |
| 1919 | -3.2 | cp | desmethylclozapine | Acetylcholine receptor agonist | Acetylcholine receptor agonist | |
| 1920 | -3.21 | cp | BRL-50481 | Phosphodiesterase inhibitor | Phosphodiesterase inhibitor | |
| 1921 | -3.22 | cp | cucurbitacin-i | JAK inhibitor | JAK inhibitor, Lipocortin synthesis stimulant, STAT inhibitor | |
| 1922 | -3.22 | cp | progesterone | Progesterone receptor agonist | Progesterone receptor agonist | |
| 1923 | -3.24 | cp | stiripentol | GABA uptake inhibitor | GABA uptake inhibitor | |
| 1924 | -3.24 | cp | ivachtin | Caspase inhibitor | Caspase inhibitor | |
| 1925 | -3.27 | cp | pseudopelletierine | Anthelmintic | Anthelmintic | |
| 1926 | -3.28 | cp | ribavirin | Antiviral | Antiviral | |
| 1927 | -3.35 | cp | motesanib | KIT inhibitor | KIT inhibitor, PDGFR receptor inhibitor, VEGFR inhibitor | |
| 1928 | -3.35 | cp | LY-320135 | Cannabinoid receptor antagonist | Cannabinoid receptor antagonist | |
| 1929 | -3.38 | cp | salicin | Anti-inflammatory | Anti-inflammatory | |
| 1930 | -3.39 | cp | z-prolyl-prolinal | Prolyl endopeptidase inhibitor | Prolyl endopeptidase inhibitor | |
| 1931 | -3.43 | cp | cinnarizine | Calcium channel blocker | Calcium channel blocker | |
| 1932 | -3.43 | cp | talipexole | Adrenergic receptor agonist | Adrenergic receptor agonist, Dopamine receptor agonist | |
| 1933 | -3.45 | cp | mefexamide | Psychoactive drug | Psychoactive drug | |
| 1934 | -3.47 | cp | fluocinonide | Glucocorticoid receptor agonist | Glucocorticoid receptor agonist | |
| 1935 | -3.49 | cp | levetiracetam | Calcium channel blocker | Calcium channel blocker | |
| 1936 | -3.49 | cp | iocetamic-acid | Radiopaque medium | Radiopaque medium | |
| 1937 | -3.54 | cp | andarine | Androgen receptor modulator | Androgen receptor modulator | |
| 1938 | -3.54 | cp | phenylbutazone | Cyclooxygenase inhibitor | Cyclooxygenase inhibitor, Prostanoid receptor antagonist | |
| 1939 | -3.56 | cp | TG100-115 | -666 | -666 |  |
| 1940 | -3.59 | cp | BCI-hydrochloride | Protein phosphatase inhibitor | Protein phosphatase inhibitor | |
| 1941 | -3.59 | cp | YM-298198 | Glutamate receptor antagonist | Glutamate receptor antagonist | |
| 1942 | -3.6 | cp | aminopentamide | Acetylcholine receptor antagonist | Acetylcholine receptor antagonist | |
| 1943 | -3.63 | cp | angiogenesis-inhibitor | Angiogenesis inhibitor | Angiogenesis inhibitor | |
| 1944 | -3.63 | cp | estropipate | Estrogen receptor agonist | Estrogen receptor agonist | |
| 1945 | -3.7 | cp | androstanol | CAR antagonist | CAR antagonist | |
| 1946 | -3.7 | cp | tacrolimus | Calcineurin inhibitor | Calcineurin inhibitor | |
| 1947 | -3.77 | cp | fluprostenol | Prostanoid receptor agonist | Prostanoid receptor agonist | |
| 1948 | -3.8 | cp | benzydamine | Membrane integrity inhibitor | Membrane integrity inhibitor, Prostanoid receptor antagonist, Prostanoid receptor inhibitor | |
| 1949 | -3.81 | cp | bisindolylmaleimide | CDK inhibitor | CDK inhibitor | |
| 1950 | -3.82 | cp | eprosartan | Angiotensin receptor antagonist | Angiotensin receptor antagonist | |
| 1951 | -3.82 | cp | vincristine | Tubulin inhibitor | Tubulin inhibitor | |
| 1952 | -3.82 | cp | L-689560 | Glutamate receptor antagonist | Glutamate receptor antagonist | |
| 1953 | -3.84 | cp | LY-255283 | Leukotriene receptor antagonist | Leukotriene receptor antagonist | |
| 1954 | -3.84 | cp | EMD-386088 | Serotonin receptor agonist | Serotonin receptor agonist | |
| 1955 | -3.86 | cp | 7b-cis | Exportin antagonist | Exportin antagonist | |
| 1956 | -3.89 | cp | rosiglitazone | Insulin sensitizer | Insulin sensitizer, PPAR receptor agonist | |
| 1957 | -3.95 | cp | JTE-907 | Cannabinoid receptor inverse agonist | Cannabinoid receptor inverse agonist | |
| 1958 | -3.96 | cp | veratridine | Sodium channel activator | Sodium channel activator | |
| 1959 | -3.98 | cp | avrainvillamide-analog-1 | nucleophosmin inhibitor | nucleophosmin inhibitor | |
| 1960 | -3.98 | cp | VU-0418946-1 | HIF modulator | HIF modulator | |
| 1961 | -3.99 | cp | tetrahydrocannabinol-7-oic-acid | Anti-inflammatory | Anti-inflammatory, Cannabinoid metabolite | |
| 1962 | -4.02 | cp | febuxostat | Xanthine oxidase inhibitor | Xanthine oxidase inhibitor | |
| 1963 | -4.03 | cp | PHTPP | Estrogen receptor antagonist | Estrogen receptor antagonist | |
| 1964 | -4.05 | cp | JWH-015 | Cannabinoid receptor agonist | Cannabinoid receptor agonist | |
| 1965 | -4.07 | cp | guaifenesin | Expectorant | Expectorant | |
| 1966 | -4.17 | cp | homosalate | HSP inducer | HSP inducer | |
| 1967 | -4.18 | cp | retinol | Retinoid receptor ligand | Retinoid receptor ligand | |
| 1968 | -4.18 | cp | U-18666A | Oxidosqualene cyclase inhibitor | Oxidosqualene cyclase inhibitor | |
| 1969 | -4.19 | cp | alrestatin | Aldose reductase inhibitor | Aldose reductase inhibitor | |
| 1970 | -4.22 | cp | memantine | Glutamate receptor antagonist | Glutamate receptor antagonist | |
| 1971 | -4.24 | cp | mometasone | Glucocorticoid receptor agonist | Glucocorticoid receptor agonist, Immunosuppressant | |
| 1972 | -4.28 | cp | WH-4023 | SRC inhibitor | SRC inhibitor | |
| 1973 | -4.28 | cp | guanabenz | Adrenergic receptor agonist | Adrenergic receptor agonist | |
| 1974 | -4.44 | cp | acetyl-farnesyl-cysteine | Inhibitor of methylation of endogenous isoprenylated proteins | Inhibitor of methylation of endogenous isoprenylated proteins | |
| 1975 | -4.46 | cp | fulvestrant | Estrogen receptor antagonist | Estrogen receptor antagonist | |
| 1976 | -4.48 | cp | valsartan | Angiotensin receptor antagonist | Angiotensin receptor antagonist | |
| 1977 | -4.48 | cp | glycocholic-acid | Cholesterol inhibitor | Cholesterol inhibitor | |
| 1978 | -4.51 | cp | dubinidine | Anti-epileptic | Anti-epileptic | |
| 1979 | -4.55 | cp | indatraline | Norepinephrine transporter inhibitor | Norepinephrine transporter inhibitor | |
| 1980 | -4.55 | cp | SB-218078 | CHK inhibitor | CHK inhibitor | |
| 1981 | -4.55 | cp | meloxicam | Cyclooxygenase inhibitor | Cyclooxygenase inhibitor | |
| 1982 | -4.66 | cp | HG-5-88-01 | Protein kinase inhibitor | Protein kinase inhibitor | |
| 1983 | -4.66 | cp | reboxetine | Adrenergic receptor antagonist | Adrenergic receptor antagonist | |
| 1984 | -4.66 | cp | umbelliferone | Cyclooxygenase inhibitor | Cyclooxygenase inhibitor | |
| 1985 | -4.76 | cp | prednisone | Glucocorticoid receptor agonist | Glucocorticoid receptor agonist | |
| 1986 | -4.83 | cp | farnesol | FXR agonist | FXR agonist, Monoamine oxidase inhibitor, NFkB pathway activator, PPAR receptor agonist | |
| 1987 | -4.87 | cp | GBR-12783 | Dopamine uptake inhibitor | Dopamine uptake inhibitor | |
| 1988 | -4.89 | cp | L-670596 | Prostanoid receptor antagonist | Prostanoid receptor antagonist | |
| 1989 | -4.93 | cp | tyrphostin-AG-556 | EGFR inhibitor | EGFR inhibitor | |
| 1990 | -4.94 | cp | QX-314 | Sodium channel blocker | Sodium channel blocker | |
| 1991 | -4.94 | cp | I-OMe-AG-538 | IGF-1 inhibitor | IGF-1 inhibitor | |
| 1992 | -4.96 | cp | spiramide | Dopamine receptor antagonist | Dopamine receptor antagonist, Serotonin receptor antagonist | |
| 1993 | -4.98 | cp | vinblastine | Microtubule inhibitor | Microtubule inhibitor, Tubulin inhibitor | |
| 1994 | -5 | cp | betulinic-acid | Apoptosis stimulant | Apoptosis stimulant, Caspase activator, Diacylglycerol O acyltransferase inhibitor, HIV integrase inhibitor, NFkB pathway activator, NFkB pathway inhibitor, SARS coronavirus 3C-like protease inhibitor, Topoisomerase inhibitor | |
| 1995 | -5 | cp | U-0126 | MEK inhibitor | MEK inhibitor | |
| 1996 | -5.03 | cp | betamethasone | Glucocorticoid receptor agonist | Glucocorticoid receptor agonist, Anti-inflammatory | |
| 1997 | -5.04 | cp | dihydrosamidin | Phospholipase inhibitor | Phospholipase inhibitor, Nitric oxide production inhibitor, Platelet activating factor receptor antagonist | |
| 1998 | -5.07 | cp | piribedil | Dopamine receptor agonist | Dopamine receptor agonist | |
| 1999 | -5.11 | cp | zalcitabine | Nucleoside reverse transcriptase inhibitor | Nucleoside reverse transcriptase inhibitor | |
| 2000 | -5.13 | cp | fosinopril | ACE inhibitor | ACE inhibitor | |
| 2001 | -5.14 | cp | riboflavin | Vitamin B | Vitamin B | |
| 2002 | -5.18 | cp | pregnenolone | Glutamate receptor modulator | Glutamate receptor modulator, Acetylcholine release enhancer, Dopamine release enhancer, GABA receptor negative allosteric modulator | |
| 2003 | -5.21 | cp | torin-1 | MTOR inhibitor | MTOR inhibitor, PI3K inhibitor | |
| 2004 | -5.21 | cp | ozagrel | Thromboxane synthase inhibitor | Thromboxane synthase inhibitor | |
| 2005 | -5.25 | cp | axitinib | PDGFR receptor inhibitor | PDGFR receptor inhibitor, VEGFR inhibitor | |
| 2006 | -5.32 | cp | nitrocaramiphen | Cholinergic receptor antagonist | Cholinergic receptor antagonist | |
| 2007 | -5.32 | cp | BAS-09104376 | HIV integrase inhibitor | HIV integrase inhibitor | |
| 2008 | -5.36 | cp | RO-08-2750 | NGF binding inhibitor | NGF binding inhibitor | |
| 2009 | -5.39 | cp | liothyronine | Thyroid hormone stimulant | Thyroid hormone stimulant | |
| 2010 | -5.43 | cp | AS-604850 | PI3K inhibitor | PI3K inhibitor | |
| 2011 | -5.48 | cp | vitexin | Antioxidant | Antioxidant | |
| 2012 | -5.51 | cp | ambelline | Plant alkaloid | Plant alkaloid | |
| 2013 | -5.53 | cp | heliomycin | ATP synthase inhibitor | ATP synthase inhibitor | |
| 2014 | -5.55 | cp | hyperoside | Glucosidase inhibitor | Glucosidase inhibitor | |
| 2015 | -5.56 | cp | primidone | GABA receptor antagonist | GABA receptor antagonist | |
| 2016 | -5.58 | cp | BD-1047 | Adrenergic receptor antagonist | Adrenergic receptor antagonist | |
| 2017 | -5.59 | cp | diazoxide | Potassium channel activator | Potassium channel activator | |
| 2018 | -5.63 | cp | cyclosporin-a | Calcineurin inhibitor | Calcineurin inhibitor | |
| 2019 | -5.65 | cp | reserpic-acid | Norepinephrine transporter inhibitor | Norepinephrine transporter inhibitor | |
| 2020 | -5.66 | cp | GW-1929 | PPAR receptor agonist | PPAR receptor agonist, Insulin sensitizer | |
| 2021 | -5.67 | cp | trimidox | Ribonucleotide reductase inhibitor | Ribonucleotide reductase inhibitor | |
| 2022 | -5.68 | cp | taurocholic-acid | Bile acid | Bile acid | |
| 2023 | -5.71 | cp | pseudoephedrine | Adrenergic receptor agonist | Adrenergic receptor agonist | |
| 2024 | -5.71 | cp | L-693403 | Sigma receptor agonist | Sigma receptor agonist, Sigma receptor antagonist | |
| 2025 | -5.74 | cp | TAK-715 | p38 MAPK inhibitor | p38 MAPK inhibitor | |
| 2026 | -5.78 | cp | tenoxicam | Cyclooxygenase inhibitor | Cyclooxygenase inhibitor | |
| 2027 | -5.84 | cp | gossypol | BCL inhibitor | BCL inhibitor, MCL1 inhibitor | |
| 2028 | -5.85 | cp | BX-912 | Pyruvate dehydrogenase kinase inhibitor | Pyruvate dehydrogenase kinase inhibitor | |
| 2029 | -5.87 | cp | VER-155008 | HSP inhibitor | HSP inhibitor | |
| 2030 | -5.88 | cp | SA-63133 | -666 | -666 |  |
| 2031 | -5.88 | cp | Y-27632 | Rho associated kinase inhibitor | Rho associated kinase inhibitor | |
| 2032 | -5.9 | cp | dicycloverine | Acetylcholine receptor antagonist | Acetylcholine receptor antagonist | |
| 2033 | -5.91 | cp | CGP-54626 | GABA receptor antagonist | GABA receptor antagonist | |
| 2034 | -5.93 | cp | penicillic-acid | other antibiotic | other antibiotic | |
| 2035 | -5.97 | cp | metanephrine | Epinephrine metabolite | Epinephrine metabolite | |
| 2036 | -5.97 | cp | stavudine | DNA directed DNA polymerase inhibitor | DNA directed DNA polymerase inhibitor, Reverse transcriptase inhibitor | |
| 2037 | -6.01 | cp | loteprednol | Glucocorticoid receptor agonist | Glucocorticoid receptor agonist, Phospholipase inhibitor | |
| 2038 | -6.13 | cp | ALW-II-49-7 | Ephrin inhibitor | Ephrin inhibitor | |
| 2039 | -6.18 | cp | terconazole | Sterol demethylase inhibitor | Sterol demethylase inhibitor | |
| 2040 | -6.2 | cp | eriodictyol | Cytochrome P450 inhibitor | Cytochrome P450 inhibitor | |
| 2041 | -6.21 | cp | terreic-acid | BTK inhibitor | BTK inhibitor | |
| 2042 | -6.35 | cp | NPC-15199 | ICAM1 antagonist | ICAM1 antagonist | |
| 2043 | -6.38 | cp | SB-431542 | TGF beta receptor inhibitor | TGF beta receptor inhibitor | |
| 2044 | -6.39 | cp | tegaserod | Serotonin receptor partial agonist | Serotonin receptor partial agonist | |
| 2045 | -6.43 | cp | tiagabine | GABA uptake inhibitor | GABA uptake inhibitor | |
| 2046 | -6.44 | cp | valproic-acid | HDAC inhibitor | HDAC inhibitor | |
| 2047 | -6.46 | cp | budesonide | Glucocorticoid receptor agonist | Glucocorticoid receptor agonist | |
| 2048 | -6.56 | cp | benzamil | Sodium channel blocker | Sodium channel blocker | |
| 2049 | -6.66 | cp | metixene | Acetylcholine receptor antagonist | Acetylcholine receptor antagonist | |
| 2050 | -6.75 | cp | CGP-71683 | Neuropeptide receptor antagonist | Neuropeptide receptor antagonist | |
| 2051 | -6.76 | cp | robustic-acid | cAMP inhibitor | cAMP inhibitor | |
| 2052 | -6.8 | cp | halometasone | Glucocorticoid receptor agonist | Glucocorticoid receptor agonist | |
| 2053 | -6.86 | cp | pepstatin | Aspartic protease inhibitor | Aspartic protease inhibitor | |
| 2054 | -6.91 | cp | phenacetin | Cyclooxygenase inhibitor | Cyclooxygenase inhibitor | |
| 2055 | -6.94 | cp | cytochalasin-d | Actin polymerization inhibitor | Actin polymerization inhibitor | |
| 2056 | -6.99 | cp | BMY-7378 | Adrenergic receptor antagonist | Adrenergic receptor antagonist, Serotonin receptor antagonist | |
| 2057 | -7.16 | cp | immethridine | Histamine receptor agonist | Histamine receptor agonist | |
| 2058 | -7.18 | cp | piperidolate | Acetylcholine receptor antagonist | Acetylcholine receptor antagonist | |
| 2059 | -7.26 | cp | GW-843682X | PLK inhibitor | PLK inhibitor | |
| 2060 | -7.26 | cp | BNTX | Opioid receptor antagonist | Opioid receptor antagonist | |
| 2061 | -7.27 | cp | zolpidem | Benzodiazepine receptor agonist | Benzodiazepine receptor agonist | |
| 2062 | -7.29 | cp | protriptyline | Tricyclic antidepressant | Tricyclic antidepressant | |
| 2063 | -7.3 | cp | roxatidine | Histamine receptor antagonist | Histamine receptor antagonist | |
| 2064 | -7.51 | cp | physostigmine | Acetylcholinesterase inhibitor | Acetylcholinesterase inhibitor, Cholinesterase inhibitor | |
| 2065 | -7.56 | cp | lobendazole | Anthelmintic | Anthelmintic | |
| 2066 | -7.6 | cp | temefos | Cholinesterase inhibitor | Cholinesterase inhibitor | |
| 2067 | -7.65 | cp | BIBU-1361 | EGFR inhibitor | EGFR inhibitor | |
| 2068 | -7.7 | cp | perospirone | Dopamine receptor antagonist | Dopamine receptor antagonist, Serotonin receptor antagonist | |
| 2069 | -7.81 | cp | dexamethasone | Glucocorticoid receptor agonist | Glucocorticoid receptor agonist | |
| 2070 | -7.86 | cp | medroxyprogesterone | progesterone receptor agonist | progesterone receptor agonist | |
| 2071 | -7.92 | cp | cromoglicic-acid | Immunosuppressant | Immunosuppressant | |
| 2072 | -8 | cp | RS-67333 | Serotonin receptor partial agonist | Serotonin receptor partial agonist | |
| 2073 | -8.01 | cp | palmitoylethanolamide | Cannabinoid receptor agonist | Cannabinoid receptor agonist | |
| 2074 | -8.04 | cp | fluticasone | Glucocorticoid receptor agonist | Glucocorticoid receptor agonist | |
| 2075 | -8.07 | cp | karakoline | Phytotoxin | Phytotoxin | |
| 2076 | -8.19 | cp | cetraxate | Mucus protecting agent | Mucus protecting agent | |
| 2077 | -8.21 | cp | RS-16566 | Serotonin receptor antagonist | Serotonin receptor antagonist | |
| 2078 | -8.21 | cp | PRL-3-inhibitor-I | Tyrosine phosphatase inhibitor | Tyrosine phosphatase inhibitor | |
| 2079 | -8.22 | cp | nicergoline | Adrenergic receptor antagonist | Adrenergic receptor antagonist | |
| 2080 | -8.24 | cp | idebenone | Calcium channel modulator | Calcium channel modulator | |
| 2081 | -8.28 | cp | methyllycaconitine | Acetylcholine receptor antagonist | Acetylcholine receptor antagonist | |
| 2082 | -8.39 | cp | clofazimine | GK0582 inhibitor | GK0582 inhibitor | |
| 2083 | -8.42 | cp | anisomycin | DNA synthesis inhibitor | DNA synthesis inhibitor | |
| 2084 | -8.43 | cp | phenolphthalein | Indicator dye | Indicator dye | |
| 2085 | -8.45 | cp | thapsigargin | ATPase inhibitor | ATPase inhibitor | |
| 2086 | -8.52 | cp | indoprofen | Cyclooxygenase inhibitor | Cyclooxygenase inhibitor, Prostanoid receptor antagonist | |
| 2087 | -8.53 | cp | ornidazole | Antiprotozoal | Antiprotozoal | |
| 2088 | -8.54 | cp | ipsapirone | Serotonin receptor agonist | Serotonin receptor agonist | |
| 2089 | -8.79 | cp | SR-95639A | Acetylcholine receptor agonist | Acetylcholine receptor agonist | |
| 2090 | -8.8 | cp | amantadine | Glutamate receptor antagonist | Glutamate receptor antagonist | |
| 2091 | -8.84 | cp | KIN001-055 | EGFR inhibitor | EGFR inhibitor, JAK inhibitor, Leukotriene inhibitor, Mediator release inhibitor | |
| 2092 | -9 | cp | mepyramine | Histamine receptor antagonist | Histamine receptor antagonist | |
| 2093 | -9.07 | cp | buspirone | Serotonin receptor agonist | Serotonin receptor agonist | |
| 2094 | -9.07 | cp | darinaparsin | Apoptosis stimulant | Apoptosis stimulant | |
| 2095 | -9.11 | cp | R-59022 | Diacylglycerol kinase inhibitor | Diacylglycerol kinase inhibitor, Protein kinase inhibitor | |
| 2096 | -9.12 | cp | epicatechin | Bacterial DNA gyrase inhibitor | Bacterial DNA gyrase inhibitor, Cyclooxygenase inhibitor, DNA polymerase inhibitor | |
| 2097 | -9.36 | cp | oxibendazole | Tubulin inhibitor | Tubulin inhibitor | |
| 2098 | -9.37 | cp | VX-222 | HCV inhibitor | HCV inhibitor | |
| 2099 | -9.37 | cp | depudecin | HDAC inhibitor | HDAC inhibitor | |
| 2100 | -9.55 | cp | W-9 | Calmodulin antagonist | Calmodulin antagonist | |
| 2101 | -9.68 | cp | REV-5901 | Leukotriene receptor antagonist | Leukotriene receptor antagonist, Lipoxygenase inhibitor | |
| 2102 | -9.78 | cp | CGP-20712 | Adrenergic receptor antagonist | Adrenergic receptor antagonist | |
| 2103 | -9.82 | cp | dovitinib | EGFR inhibitor | EGFR inhibitor, FLT3 inhibitor, FGFR inhibitor, PDGFR receptor inhibitor, VEGFR inhibitor | |
| 2104 | -9.88 | cp | erismodegib | Smoothened receptor antagonist | Smoothened receptor antagonist | |
| 2105 | -9.94 | cp | dydrogesterone | Progesterone receptor agonist | Progesterone receptor agonist | |
| 2106 | -10.24 | cp | prestwick-559 | Dopamine receptor agonist | Dopamine receptor agonist | |
| 2107 | -10.24 | cp | pipamperone | Dopamine receptor antagonist | Dopamine receptor antagonist | |
| 2108 | -10.32 | cp | methazolamide | Carbonic anhydrase inhibitor | Carbonic anhydrase inhibitor | |
| 2109 | -10.34 | cp | 10H-phenothiazin-10-yl)(p-tolyl)methanone | Butyrylcholinesterase inhibitor | Butyrylcholinesterase inhibitor | |
| 2110 | -10.41 | cp | pioglitazone | Insulin sensitizer | Insulin sensitizer, PPAR receptor agonist | |
| 2111 | -10.51 | cp | splitomycin | SIRT inhibitor | SIRT inhibitor | |
| 2112 | -10.55 | cp | CFM-1571 | Guanylate cyclase activator | Guanylate cyclase activator | |
| 2113 | -10.72 | cp | tyrphostin-AG-112 | Protein tyrosine kinase inhibitor | Protein tyrosine kinase inhibitor | |
| 2114 | -10.74 | cp | mupirocin | Isoleucyl-tRNA synthetase inhibitor | Isoleucyl-tRNA synthetase inhibitor | |
| 2115 | -10.89 | cp | tripelennamine | Histamine receptor antagonist | Histamine receptor antagonist | |
| 2116 | -10.95 | cp | eriochrome-black-t | Azo dye | Azo dye |  |
| 2117 | -10.97 | cp | suloctidil | Adrenergic receptor antagonist | Adrenergic receptor antagonist | |
| 2118 | -10.98 | cp | 3,3'-diindolylmethane | CHK inhibitor | CHK inhibitor, Cytochrome P450 activator, Indoleamine 2,3-dioxygenase inhibitor | |
| 2119 | -11.03 | cp | safinamide | Dopamine uptake inhibitor | Dopamine uptake inhibitor, Glutamate inhibitor, Monoamine oxidase inhibitor | |
| 2120 | -11.03 | cp | ICI-89406 | Adrenergic receptor antagonist | Adrenergic receptor antagonist | |
| 2121 | -11.06 | cp | tipifarnib-P2 | farnesyltransferase inhibitor | farnesyltransferase inhibitor | |
| 2122 | -11.09 | cp | JNJ-16259685 | Glutamate receptor antagonist | Glutamate receptor antagonist | |
| 2123 | -11.1 | cp | benzatropine | Acetylcholine receptor antagonist | Acetylcholine receptor antagonist | |
| 2124 | -11.23 | cp | hyperforin | Cyclooxygenase inhibitor | Cyclooxygenase inhibitor, Dopamine uptake inhibitor, Interleukin receptor antagonist, Lipoxygenase inhibitor, Serotonin reuptake inhibitor | |
| 2125 | -11.25 | cp | probucol | Atherogenesis inhibitor | Atherogenesis inhibitor | |
| 2126 | -11.26 | cp | SB-222200 | Tachykinin antagonist | Tachykinin antagonist | |
| 2127 | -11.41 | cp | ST-91 | Adrenergic receptor agonist | Adrenergic receptor agonist | |
| 2128 | -11.41 | cp | atenolol | Adrenergic receptor antagonist | Adrenergic receptor antagonist | |
| 2129 | -11.63 | cp | oxantel | Anthelmintic | Anthelmintic | |
| 2130 | -11.73 | cp | isoliquiritigenin | Guanylate cyclase activator | Guanylate cyclase activator | |
| 2131 | -11.74 | cp | NSC-94258 | Antineoplastic | Antineoplastic | |
| 2132 | -11.74 | cp | sildenafil | Phosphodiesterase inhibitor | Phosphodiesterase inhibitor | |
| 2133 | -11.83 | cp | tremorine | Acetylcholine receptor agonist | Acetylcholine receptor agonist | |
| 2134 | -11.88 | cp | phentolamine | Adrenergic receptor antagonist | Adrenergic receptor antagonist | |
| 2135 | -11.95 | cp | VEGF-receptor-2-kinase-inhibitor-IV | VEGFR inhibitor | VEGFR inhibitor | |
| 2136 | -12.07 | cp | gemcitabine | Ribonucleotide reductase inhibitor | Ribonucleotide reductase inhibitor | |
| 2137 | -12.2 | cp | ZM-241385 | Adenosine receptor antagonist | Adenosine receptor antagonist | |
| 2138 | -12.45 | cp | CA-074-Me | Cathepsin inhibitor | Cathepsin inhibitor | |
| 2139 | -12.63 | cp | brucine | Glycine receptor antagonist | Glycine receptor antagonist | |
| 2140 | -12.75 | cp | zebularine | DNA methyltransferase inhibitor | DNA methyltransferase inhibitor | |
| 2141 | -12.77 | cp | GSK-3-inhibitor-IX | Glycogen synthase kinase inhibitor | Glycogen synthase kinase inhibitor, Lipoxygenase inhibitor | |
| 2142 | -12.9 | cp | SRC-kinase-inhibitor-II | SRC inhibitor | SRC inhibitor | |
| 2143 | -12.9 | cp | catechin | Beta secretase inhibitor | Beta secretase inhibitor, Fatty acid synthase inhibitor, LDL antioxidant | |
| 2144 | -13.05 | cp | IKK-2-inhibitor | IKK inhibitor | IKK inhibitor, SYK inhibitor | |
| 2145 | -13.08 | cp | podophyllotoxin | Microtubule inhibitor | Microtubule inhibitor, Tubulin inhibitor | |
| 2146 | -13.24 | cp | niguldipine | Calcium channel blocker | Calcium channel blocker, Adrenergic receptor antagonist | |
| 2147 | -13.27 | cp | etacrynic-acid | Sodium/potassium/chloride transporter inhibitor | Sodium/potassium/chloride transporter inhibitor | |
| 2148 | -13.41 | cp | tenovins | SIRT inhibitor | SIRT inhibitor, TP53 activator | |
| 2149 | -13.44 | cp | SQ-29548 | Thromboxane receptor antagonist | Thromboxane receptor antagonist | |
| 2150 | -13.49 | cp | diphenyleneiodonium | Nitric oxide synthase inhibitor | Nitric oxide synthase inhibitor | |
| 2151 | -13.57 | cp | AZD-6482 | PI3K inhibitor | PI3K inhibitor | |
| 2152 | -13.74 | cp | MRS-1754 | Adenosine receptor antagonist | Adenosine receptor antagonist | |
| 2153 | -14.07 | cp | H-89 | PKA inhibitor | PKA inhibitor | |
| 2154 | -14.17 | cp | tosyl-phenylalanyl-chloromethyl-ketone | Chymotrypsin inhibitor | Chymotrypsin inhibitor | |
| 2155 | -14.32 | cp | albendazole | Anthelmintic | Anthelmintic, Tubulin inhibitor | |
| 2156 | -14.45 | cp | BU-226 | Imidazoline receptor ligand | Imidazoline receptor ligand | |
| 2157 | -14.46 | cp | 4-hydroxy-2-nonenal | Cytotoxic lipid peroxidation product | Cytotoxic lipid peroxidation product | |
| 2158 | -14.51 | cp | ganglioside | SRC activator | SRC activator | |
| 2159 | -14.53 | cp | zuclopenthixol | Dopamine receptor antagonist | Dopamine receptor antagonist | |
| 2160 | -14.54 | cp | losartan | Angiotensin receptor antagonist | Angiotensin receptor antagonist | |
| 2161 | -14.55 | cp | isorotenone | Mitochondrial complex I inhibitor | Mitochondrial complex I inhibitor, NADH-ubiquinone oxidoreductase (Complex I) inhibitor | |
| 2162 | -14.57 | cp | ampicillin | Bacterial cell wall synthesis inhibitor | Bacterial cell wall synthesis inhibitor | |
| 2163 | -14.76 | cp | GSK-1904529A | IGF-1 inhibitor | IGF-1 inhibitor, IGF-1R inhibitor, Insulin receptor ligand | |
| 2164 | -14.89 | cp | necrostatin-1 | RIPK inhibitor | RIPK inhibitor | |
| 2165 | -14.94 | cp | NSC-663284 | CDC inhibitor | CDC inhibitor | |
| 2166 | -15.03 | cp | phenytoin | Hydantoin antiepileptic | Hydantoin antiepileptic | |
| 2167 | -15.04 | cp | resorcinol | Phosphodiesterase inhibitor | Phosphodiesterase inhibitor | |
| 2168 | -15.05 | cp | flurofamide | Urease inhibitor | Urease inhibitor | |
| 2169 | -15.06 | cp | GR-103691 | Dopamine receptor antagonist | Dopamine receptor antagonist | |
| 2170 | -15.09 | cp | flunisolide | Cytochrome P450 inhibitor | Cytochrome P450 inhibitor | |
| 2171 | -15.29 | cp | mesna | Antioxidant | Antioxidant | |
| 2172 | -15.53 | cp | anabasine | Acetylcholine receptor agonist | Acetylcholine receptor agonist | |
| 2173 | -15.53 | cp | azaperone | Dopamine receptor antagonist | Dopamine receptor antagonist | |
| 2174 | -15.56 | cp | zaldaride | Calmodulin antagonist | Calmodulin antagonist | |
| 2175 | -15.61 | cp | cephalosporanic-acid | Bacterial cell wall synthesis inhibitor | Bacterial cell wall synthesis inhibitor | |
| 2176 | -15.63 | cp | flupentixol | Dopamine receptor antagonist | Dopamine receptor antagonist | |
| 2177 | -15.89 | cp | zaprinast | Phosphodiesterase inhibitor | Phosphodiesterase inhibitor | |
| 2178 | -15.89 | cp | PCO-400 | Potassium channel activator | Potassium channel activator | |
| 2179 | -16.18 | cp | UK-356618 | Metalloproteinase inhibitor | Metalloproteinase inhibitor | |
| 2180 | -16.45 | cp | fenoterol | Adrenergic receptor agonist | Adrenergic receptor agonist | |
| 2181 | -16.58 | cp | EHNA | Adenosine deaminase inhibitor | Adenosine deaminase inhibitor | |
| 2182 | -16.58 | cp | L-690330 | Inositol monophosphatase inhibitor | Inositol monophosphatase inhibitor | |
| 2183 | -17.35 | cp | nitrofural | Bacterial DNA inhibitor | Bacterial DNA inhibitor | |
| 2184 | -17.54 | cp | phenprobamate | Muscle relaxant | Muscle relaxant | |
| 2185 | -17.64 | cp | fillalbin | Increases arterial blood pressure | Increases arterial blood pressure | |
| 2186 | -17.83 | cp | austricine | Hypolipidemic | Hypolipidemic | |
| 2187 | -17.98 | cp | noscapine | Bradykinin receptor antagonist | Bradykinin receptor antagonist, Tubulin inhibitor | |
| 2188 | -18 | cp | pyrvinium-pamoate | AKT inhibitor | AKT inhibitor | |
| 2189 | -18.26 | cp | tiotidine | Histamine receptor antagonist | Histamine receptor antagonist | |
| 2190 | -18.37 | cp | IRL-2500 | Endothelin receptor antagonist | Endothelin receptor antagonist | |
| 2191 | -18.6 | cp | dinoprostone | Prostanoid receptor agonist | Prostanoid receptor agonist | |
| 2192 | -18.67 | cp | ilomastat | Matrix metalloprotease inhibitor | Matrix metalloprotease inhibitor | |
| 2193 | -18.83 | cp | UB-165 | Acetylcholine receptor agonist | Acetylcholine receptor agonist | |
| 2194 | -18.99 | cp | olaparib | PARP inhibitor | PARP inhibitor | |
| 2195 | -19.09 | cp | pirinixic-acid | PPAR receptor agonist | PPAR receptor agonist | |
| 2196 | -19.24 | cp | mepireserpate | Catecholamine depleting sympatholytic | Catecholamine depleting sympatholytic | |
| 2197 | -19.28 | cp | GR-159897 | Tachykinin antagonist | Tachykinin antagonist | |
| 2198 | -19.31 | cp | cercosporin | Photoactivated toxin | Photoactivated toxin | |
| 2199 | -19.35 | cp | TER-14687 | Inhibitor of translocation of PKCq in T cells | Inhibitor of translocation of PKCq in T cells | |
| 2200 | -19.55 | cp | cefaclor | Bacterial cell wall synthesis inhibitor | Bacterial cell wall synthesis inhibitor | |
| 2201 | -19.71 | cp | piperacetazine | Dopamine receptor antagonist | Dopamine receptor antagonist | |
| 2202 | -19.73 | cp | xanthohumol | ATPase inhibitor | ATPase inhibitor | |
| 2203 | -19.73 | cp | quinpirole | Dopamine receptor agonist | Dopamine receptor agonist | |
| 2204 | -19.79 | cp | cycloheximide | Protein synthesis inhibitor | Protein synthesis inhibitor | |
| 2205 | -19.93 | cp | SC-19220 | Prostanoid receptor antagonist | Prostanoid receptor antagonist | |
| 2206 | -20.04 | cp | lumicolchicine | Colchicine isomer, non-binder of microtubules | Colchicine isomer, non-binder of microtubules | |
| 2207 | -20.13 | cp | ARC-239 | Adrenergic receptor antagonist | Adrenergic receptor antagonist | |
| 2208 | -20.16 | cp | MAPP-D-erythro | Ceramidase inhibitor | Ceramidase inhibitor | |
| 2209 | -20.47 | cp | fluorometholone | Glucocorticoid receptor agonist | Glucocorticoid receptor agonist | |
| 2210 | -20.68 | cp | lobeline | Acetylcholine receptor antagonist | Acetylcholine receptor antagonist | |
| 2211 | -20.72 | cp | BI-78D3 | JNK inhibitor | JNK inhibitor | |
| 2212 | -20.72 | cp | OMDM-2 | FAAH inhibitor | FAAH inhibitor | |
| 2213 | -20.79 | cp | clocortolone | Glucocorticoid receptor agonist | Glucocorticoid receptor agonist, Steroid | |
| 2214 | -20.87 | cp | L-692585 | Growth hormone releasing peptide ligand agonist | Growth hormone releasing peptide ligand agonist | |
| 2215 | -20.93 | cp | naringin | Cytochrome P450 inhibitor | Cytochrome P450 inhibitor | |
| 2216 | -20.99 | cp | cinalukast | Leukotriene receptor antagonist | Leukotriene receptor antagonist | |
| 2217 | -21.47 | cp | parthenolide | NFkB pathway inhibitor | NFkB pathway inhibitor, Adiponectin receptor agonist | |
| 2218 | -21.6 | cp | AG-490 | EGFR inhibitor | EGFR inhibitor, ErbB2 inhibitor, JAK inhibitor | |
| 2219 | -21.72 | cp | hydrocortisone | Glucocorticoid receptor agonist | Glucocorticoid receptor agonist, Immunosuppressant, Corticosteroid agonist | |
| 2220 | -21.79 | cp | mestanolone | Androgenic steroid | Androgenic steroid | |
| 2221 | -21.82 | cp | profenamine | Butyrylcholinesterase inhibitor | Butyrylcholinesterase inhibitor, Cholinergic receptor antagonist | |
| 2222 | -21.9 | cp | RS-102221 | Serotonin receptor antagonist | Serotonin receptor antagonist | |
| 2223 | -21.9 | cp | MDL-11939 | Serotonin receptor antagonist | Serotonin receptor antagonist | |
| 2224 | -21.96 | cp | butoconazole | Bacterial cell wall synthesis inhibitor | Bacterial cell wall synthesis inhibitor | |
| 2225 | -22.12 | cp | temozolomide | DNA alkylating agent | DNA alkylating agent | |
| 2226 | -22.27 | cp | SR-27897 | CCK receptor antagonist | CCK receptor antagonist | |
| 2227 | -22.58 | cp | QS-11 | ARFGAP inhibitor | ARFGAP inhibitor | |
| 2228 | -22.6 | cp | KUC104502N | -666 | -666 |  |
| 2229 | -22.92 | cp | RU-28318 | Cytochrome P450 inhibitor | Cytochrome P450 inhibitor | |
| 2230 | -23.04 | cp | MK-1775 | WEE1 kinase inhibitor | WEE1 kinase inhibitor | |
| 2231 | -23.13 | cp | RS-56812 | Serotonin receptor partial agonist | Serotonin receptor partial agonist | |
| 2232 | -23.24 | cp | nisoxetine | Norepinephrine reuptake inhibitor | Norepinephrine reuptake inhibitor | |
| 2233 | -23.4 | cp | MLN-2238 | Proteasome inhibitor | Proteasome inhibitor | |
| 2234 | -23.57 | cp | PNU-22394 | Serotonin receptor agonist | Serotonin receptor agonist | |
| 2235 | -23.64 | cp | salubrinal | Eukaryotic translation initiation factor inhibitor | Eukaryotic translation initiation factor inhibitor | |
| 2236 | -24 | cp | irinotecan | Topoisomerase inhibitor | Topoisomerase inhibitor | |
| 2237 | -24.04 | cp | CP-55940 | Cannabinoid receptor agonist | Cannabinoid receptor agonist | |
| 2238 | -24.36 | cp | dienestrol | Estrogen receptor agonist | Estrogen receptor agonist | |
| 2239 | -24.4 | cp | CNQX | Glutamate receptor antagonist | Glutamate receptor antagonist | |
| 2240 | -24.45 | cp | betahistine | Histamine receptor agonist | Histamine receptor agonist, Histamine receptor antagonist | |
| 2241 | -24.76 | cp | 7,8-dihydro-L-biopterin | Dihydroneopterin aldolase inhibitor | Dihydroneopterin aldolase inhibitor | |
| 2242 | -24.78 | cp | tyrphostin-AG-835 | Protein tyrosine kinase inhibitor | Protein tyrosine kinase inhibitor | |
| 2243 | -24.81 | cp | benzylpenicillin | Penicillin binding protein inhibitor | Penicillin binding protein inhibitor | |
| 2244 | -25.41 | cp | fenbendazole | Tubulin inhibitor | Tubulin inhibitor | |
| 2245 | -25.87 | cp | PP-3 | EGFR inhibitor | EGFR inhibitor | |
| 2246 | -25.94 | cp | flavoxate | Acetylcholine receptor antagonist | Acetylcholine receptor antagonist | |
| 2247 | -26.03 | cp | spiroxatrine | Serotonin receptor antagonist | Serotonin receptor antagonist | |
| 2248 | -26.62 | cp | CGP-52432 | GABA receptor antagonist | GABA receptor antagonist | |
| 2249 | -26.67 | cp | benazepril | ACE inhibitor | ACE inhibitor | |
| 2250 | -26.7 | cp | triprolidine | Histamine receptor antagonist | Histamine receptor antagonist | |
| 2251 | -26.82 | cp | CDC | Lipoxygenase inhibitor | Lipoxygenase inhibitor | |
| 2252 | -27.07 | cp | tyrphostin-B44 | EGFR inhibitor | EGFR inhibitor | |
| 2253 | -27.08 | cp | vecuronium | Acetylcholine receptor antagonist | Acetylcholine receptor antagonist | |
| 2254 | -27.29 | cp | RS-102895 | CCR antagonist | CCR antagonist | |
| 2255 | -27.37 | cp | clobetasol | Glucocorticoid receptor agonist | Glucocorticoid receptor agonist | |
| 2256 | -28.06 | cp | zafirlukast | Leukotriene receptor antagonist | Leukotriene receptor antagonist | |
| 2257 | -28.13 | cp | SB-216641 | Serotonin receptor antagonist | Serotonin receptor antagonist | |
| 2258 | -28.29 | cp | diltiazem | Calcium channel blocker | Calcium channel blocker | |
| 2259 | -28.86 | cp | marmesin | Angiogenesis inhibitor | Angiogenesis inhibitor | |
| 2260 | -28.95 | cp | heliotrine | Pyrrolizidine alkaloid | Pyrrolizidine alkaloid | |
| 2261 | -29.05 | cp | GW-7647 | PPAR receptor agonist | PPAR receptor agonist | |
| 2262 | -29.39 | cp | BX-795 | IKK inhibitor | IKK inhibitor | |
| 2263 | -29.42 | cp | erythromycin | NFkB pathway inhibitor | NFkB pathway inhibitor | |
| 2264 | -29.59 | cp | 4,5-dianilinophthalimide | EGFR inhibitor | EGFR inhibitor | |
| 2265 | -29.9 | cp | 4-(2-Amino-ethyl)-benzenesulfonamide | carbonic anhydrase inhibitor | carbonic anhydrase inhibitor | |
| 2266 | -30.09 | cp | olmesartan | Angiotensin antagonist | Angiotensin antagonist | |
| 2267 | -30.26 | cp | zamifenacin | Acetylcholine receptor antagonist | Acetylcholine receptor antagonist | |
| 2268 | -30.39 | cp | LM-1685 | Cyclooxygenase inhibitor | Cyclooxygenase inhibitor | |
| 2269 | -30.88 | cp | iobenguane | Antineoplastic | Antineoplastic | |
| 2270 | -31.04 | cp | avrainvillamide-analog-3 | nucleophosmin inhibitor | nucleophosmin inhibitor | |
| 2271 | -31.44 | cp | homoveratrylamine | Dopamine analog | Dopamine analog | |
| 2272 | -31.46 | cp | hyoscyamine | Acetylcholine receptor antagonist | Acetylcholine receptor antagonist | |
| 2273 | -31.66 | cp | ALW-II-38-3 | Ephrin inhibitor | Ephrin inhibitor | |
| 2274 | -31.85 | cp | HDAC1-selective | HDAC inhibitor | HDAC inhibitor | |
| 2275 | -32.07 | cp | MAZ-51 | VEGFR inhibitor | VEGFR inhibitor | |
| 2276 | -32.18 | cp | 3-methyl-GABA | GABA aminotransferase activator | GABA aminotransferase activator | |
| 2277 | -32.46 | cp | vindesine | Tubulin inhibitor | Tubulin inhibitor | |
| 2278 | -32.64 | cp | lysylphenylalanyl-tyrosine | Heparin activation inhibitor | Heparin activation inhibitor | |
| 2279 | -32.68 | cp | orphenadrine | Acetylcholine receptor antagonist | Acetylcholine receptor antagonist | |
| 2280 | -32.93 | cp | GANT-58 | GLI antagonist | GLI antagonist | |
| 2281 | -33.22 | cp | L-701324 | Glutamate receptor antagonist | Glutamate receptor antagonist | |
| 2282 | -33.37 | cp | rescinnamine | ACE inhibitor | ACE inhibitor | |
| 2283 | -33.4 | cp | LY-456236 | Glutamate receptor antagonist | Glutamate receptor antagonist | |
| 2284 | -33.75 | cp | metergoline | Dopamine receptor agonist | Dopamine receptor agonist, Serotonin receptor antagonist | |
| 2285 | -33.84 | cp | cyclopiazonic-acid | ATPase inhibitor | ATPase inhibitor | |
| 2286 | -34.57 | cp | candesartan | Angiotensin receptor antagonist | Angiotensin receptor antagonist | |
| 2287 | -34.78 | cp | sunitinib | PLK inhibitor | PLK inhibitor | |
| 2288 | -34.99 | cp | clofarabine | Ribonucleoside reductase inhibitor | Ribonucleoside reductase inhibitor | |
| 2289 | -35.06 | cp | ponalrestat | Aldose reductase inhibitor | Aldose reductase inhibitor | |
| 2290 | -35.14 | cp | L-165041 | PPAR receptor agonist | PPAR receptor agonist | |
| 2291 | -35.21 | cp | aspirin | Cyclooxygenase inhibitor | Cyclooxygenase inhibitor | |
| 2292 | -36.33 | cp | hydralazine | Vasodilator | Vasodilator | |
| 2293 | -36.4 | cp | halcinonide | Glucocorticoid receptor agonist | Glucocorticoid receptor agonist | |
| 2294 | -36.64 | cp | HEAT | Adrenergic receptor antagonist | Adrenergic receptor antagonist | |
| 2295 | -36.68 | cp | damnacanthal | SRC inhibitor | SRC inhibitor | |
| 2296 | -37.03 | cp | T-98475 | Gonadotropin releasing factor hormone receptor antagonist | Gonadotropin releasing factor hormone receptor antagonist | |
| 2297 | -37.03 | cp | ritanserin | Serotonin receptor antagonist | Serotonin receptor antagonist | |
| 2298 | -37.16 | cp | CDK1-5-inhibitor | CDK inhibitor | CDK inhibitor, Glycogen synthase kinase inhibitor | |
| 2299 | -37.22 | cp | ataluren | CFTR channel agonist | CFTR channel agonist, Dystrophin stimulant | |
| 2300 | -37.23 | cp | MLN-4924 | Nedd activating enzyme inhibitor | Nedd activating enzyme inhibitor | |
| 2301 | -37.79 | cp | ganciclovir | DNA polymerase inhibitor | DNA polymerase inhibitor | |
| 2302 | -38.1 | cp | CD-437 | Retinoid receptor agonist | Retinoid receptor agonist | |
| 2303 | -38.29 | cp | levocetirizine | Histamine receptor antagonist | Histamine receptor antagonist | |
| 2304 | -38.57 | cp | clebopride | Dopamine receptor antagonist | Dopamine receptor antagonist | |
| 2305 | -38.61 | cp | ioxaglic-acid | Radiopaque medium | Radiopaque medium | |
| 2306 | -39.75 | cp | CGP-7930 | GABA receptor modulator | GABA receptor modulator, GABA receptor positive allosteric modulator | |
| 2307 | -40.37 | cp | pregnenolone | glutamate receptor modulator | glutamate receptor modulator | |
| 2308 | -40.5 | cp | strychnine | Acetylcholine receptor antagonist | Acetylcholine receptor antagonist | |
| 2309 | -40.97 | cp | diprotin-a | Dipeptidyl peptidase inhibitor | Dipeptidyl peptidase inhibitor | |
| 2310 | -41.34 | cp | diphencyprone | Immunostimulant | Immunostimulant | |
| 2311 | -41.72 | cp | solanine | Acetylcholinesterase inhibitor | Acetylcholinesterase inhibitor | |
| 2312 | -41.72 | cp | periplocymarin | Apoptosis stimulant | Apoptosis stimulant | |
| 2313 | -42.13 | cp | epoxycholesterol | LXR agonist | LXR agonist | |
| 2314 | -42.18 | cp | gamma-homolinolenic-acid | Cholesterol inhibitor | Cholesterol inhibitor, Platelet aggregation inhibitor, Prostanoid receptor agonist, Prostanoid receptor stimulant | |
| 2315 | -42.48 | cp | BAY-K8644 | Calcium channel activator | Calcium channel activator, L-type calcium channel activator | |
| 2316 | -42.87 | cp | arecaidine | Acetylcholine receptor agonist | Acetylcholine receptor agonist | |
| 2317 | -43.61 | cp | alvespimycin | HSP inhibitor | HSP inhibitor | |
| 2318 | -43.73 | cp | oleylethanolamide | Cannabinoid receptor agonist | Cannabinoid receptor agonist, Potassium channel blocker, Glucose dependent insulinotropic receptor agonist | |
| 2319 | -44.32 | cp | YK-4279 | Apoptosis stimulant | Apoptosis stimulant, EWS-FLI1 inhibitor | |
| 2320 | -44.36 | cp | SB-206553 | Serotonin receptor antagonist | Serotonin receptor antagonist | |
| 2321 | -44.7 | cp | gedunin | HSP inhibitor | HSP inhibitor | |
| 2322 | -44.85 | cp | BRD-K06817181 | JAK inhibitor | JAK inhibitor | |
| 2323 | -45.05 | cp | amylocaine | Local anesthetic | Local anesthetic | |
| 2324 | -45.42 | cp | GW-9662 | PPAR receptor antagonist | PPAR receptor antagonist | |
| 2325 | -45.47 | cp | docetaxel | Tubulin inhibitor | Tubulin inhibitor | |
| 2326 | -45.63 | cp | flunarizine | Calcium channel blocker | Calcium channel blocker | |
| 2327 | -45.65 | cp | PD-168077 | Dopamine receptor agonist | Dopamine receptor agonist | |
| 2328 | -45.75 | cp | biotin | Vitamin B | Vitamin B | |
| 2329 | -45.99 | cp | CMPD-1 | p38 MAPK inhibitor | p38 MAPK inhibitor | |
| 2330 | -46.42 | cp | etifenin | Compound used in hepatobiliary scans of the liver | Compound used in hepatobiliary scans of the liver | |
| 2331 | -46.53 | cp | evoxine | Furoquinoline alkaloid | Furoquinoline alkaloid | |
| 2332 | -46.61 | cp | FIT | Opioid receptor agonist | Opioid receptor agonist | |
| 2333 | -46.93 | cp | BML-190 | Cannabinoid receptor inverse agonist | Cannabinoid receptor inverse agonist | |
| 2334 | -46.98 | cp | MST-312 | Telomerase inhibitor | Telomerase inhibitor | |
| 2335 | -47.07 | cp | LY-2140023 | Glutamate receptor agonist | Glutamate receptor agonist | |
| 2336 | -47.87 | cp | LFM-A12 | EGFR inhibitor | EGFR inhibitor | |
| 2337 | -48.09 | cp | tetrabenazine | Vesicular monoamine transporter inhibitor | Vesicular monoamine transporter inhibitor | |
| 2338 | -48.41 | cp | YC-1 | Guanylyl cyclase activator | Guanylyl cyclase activator | |
| 2339 | -48.6 | cp | vinpocetine | Phosphodiesterase inhibitor | Phosphodiesterase inhibitor, Sodium channel blocker | |
| 2340 | -48.91 | cp | tolmetin | Cyclooxygenase inhibitor | Cyclooxygenase inhibitor | |
| 2341 | -49.79 | cp | perindopril | ACE inhibitor | ACE inhibitor | |
| 2342 | -49.95 | cp | GW-3965 | LXR agonist | LXR agonist, ABC transporter expression enhancer | |
| 2343 | -50.17 | cp | ZD-7288 | HCN channel blocker | HCN channel blocker | |
| 2344 | -50.54 | cp | clioquinol | Chelating agent | Chelating agent | |
| 2345 | -52.53 | cp | BMY-45778 | IP1 prostacyclin receptor agonist | IP1 prostacyclin receptor agonist | |
| 2346 | -52.59 | cp | WYE-354 | MTOR inhibitor | MTOR inhibitor | |
| 2347 | -53.08 | cp | leu-enkephalin | Opioid receptor agonist | Opioid receptor agonist | |
| 2348 | -53.66 | cp | retrorsine | Antimitotic | Antimitotic, Cytotoxic agent, Mutagenic | |
| 2349 | -54.14 | cp | acebutolol | Adrenergic receptor antagonist | Adrenergic receptor antagonist | |
| 2350 | -54.29 | cp | nTZDpa | PPAR receptor agonist | PPAR receptor agonist | |
| 2351 | -54.3 | cp | etodolac | Cyclooxygenase inhibitor | Cyclooxygenase inhibitor | |
| 2352 | -54.5 | cp | CAY-10618 | NAMPT inhibitor | NAMPT inhibitor | |
| 2353 | -54.91 | cp | 17-beta-estradiol | Estrogen receptor agonist | Estrogen receptor agonist | |
| 2354 | -55.01 | cp | entecavir | Reverse transcriptase inhibitor | Reverse transcriptase inhibitor, DNA replication inhibitor | |
| 2355 | -56.19 | cp | SB-218795 | Tachykinin antagonist | Tachykinin antagonist | |
| 2356 | -56.76 | cp | SA-792574 | Microtubule inhibitor | Microtubule inhibitor | |
| 2357 | -56.83 | cp | SB-216763 | Glycogen synthase kinase inhibitor | Glycogen synthase kinase inhibitor | |
| 2358 | -58.14 | cp | triciribine | AKT inhibitor | AKT inhibitor | |
| 2359 | -58.41 | cp | fluocinonide | Glucocorticoid receptor agonist | Glucocorticoid receptor agonist | |
| 2360 | -58.95 | cp | oleanolic-acid | G protein-coupled receptor agonist | G protein-coupled receptor agonist | |
| 2361 | -59.35 | cp | nomilin | HSP inhibitor | HSP inhibitor | |
| 2362 | -61.48 | cp | acetyl-geranygeranyl-cysteine | Inhibitor of methyl esterification of geranylgeranylated proteins | Inhibitor of methyl esterification of geranylgeranylated proteins | |
| 2363 | -61.53 | cp | CHEMBL-374350 | NFkB pathway inhibitor | NFkB pathway inhibitor | |
| 2364 | -61.8 | cp | maraviroc | CC chemokine receptor antagonist | CC chemokine receptor antagonist | |
| 2365 | -61.92 | cp | cilomilast | Phosphodiesterase inhibitor | Phosphodiesterase inhibitor | |
| 2366 | -61.98 | cp | hydrocortisone | Glucocorticoid receptor agonist | Glucocorticoid receptor agonist | |
| 2367 | -63.02 | cp | neurodazine | Neurogenesis of non-pluripotent C2C12 myoblast inducer | Neurogenesis of non-pluripotent C2C12 myoblast inducer | |
| 2368 | -63.16 | cp | MK-212 | Serotonin receptor agonist | Serotonin receptor agonist | |
| 2369 | -64.25 | cp | hemado | Adenosine receptor agonist | Adenosine receptor agonist | |
| 2370 | -64.95 | cp | isradipine | Calcium channel blocker | Calcium channel blocker | |
| 2371 | -65.09 | cp | VU-0418947-2 | HIF modulator | HIF modulator | |
| 2372 | -65.5 | cp | capsaicin | TRPV agonist | TRPV agonist | |
| 2373 | -66.07 | cp | vinburnine | Adrenergic receptor antagonist | Adrenergic receptor antagonist | |
| 2374 | -66.09 | cp | 16,16-dimethylprostaglandin-e2 | Prostanoid receptor agonist | Prostanoid receptor agonist | |
| 2375 | -66.43 | cp | acitretin | Retinoid receptor agonist | Retinoid receptor agonist | |
| 2376 | -66.93 | cp | voriconazole | Cytochrome P450 inhibitor | Cytochrome P450 inhibitor | |
| 2377 | -67.17 | cp | 1,2-dichlorobenzene | Hepatotoxicant | Hepatotoxicant, Organic compound | |
| 2378 | -68.6 | cp | AGK-2 | SIRT inhibitor | SIRT inhibitor | |
| 2379 | -69.24 | cp | eugenol | Androgen receptor antagonist | Androgen receptor antagonist | |
| 2380 | -69.41 | cp | tyrphostin-AG-1295 | PDGFR receptor inhibitor | PDGFR receptor inhibitor | |
| 2381 | -69.58 | cp | parthenolide | NFkB pathway inhibitor | NFkB pathway inhibitor | |
| 2382 | -70.27 | cp | JNJ-26854165 | HDAC inhibitor | HDAC inhibitor | |
| 2383 | -70.52 | cp | acetohydroxamic-acid | Urease inhibitor | Urease inhibitor | |
| 2384 | -70.87 | cp | altrenogest | Progestogen hormone | Progestogen hormone | |
| 2385 | -71.62 | cp | tubocurarine | Acetylcholine receptor antagonist | Acetylcholine receptor antagonist | |
| 2386 | -71.65 | cp | hydrastine | Tyrosine hydroxylase inhibitor | Tyrosine hydroxylase inhibitor | |
| 2387 | -72.34 | cp | rosmarinic-acid | GABA transaminase inhibitor | GABA transaminase inhibitor | |
| 2388 | -72.5 | cp | huperzine-a | Acetylcholinesterase inhibitor | Acetylcholinesterase inhibitor | |
| 2389 | -72.66 | cp | mirin | MRE11A exonuclease inhibitor | MRE11A exonuclease inhibitor | |
| 2390 | -73.09 | cp | SU-11274 | Hepatocyte growth factor receptor inhibitor | Hepatocyte growth factor receptor inhibitor, Tyrosine kinase inhibitor | |
| 2391 | -73.23 | cp | vinorelbine | Tubulin inhibitor | Tubulin inhibitor | |
| 2392 | -73.66 | cp | TW-37 | BCL inhibitor | BCL inhibitor | |
| 2393 | -74.2 | cp | embelin | HCV inhibitor | HCV inhibitor, XIAP inhibitor | |
| 2394 | -74.73 | cp | obatoclax | BCL inhibitor | BCL inhibitor | |
| 2395 | -75.01 | cp | hydrocortisone | Glucocorticoid receptor agonist | Glucocorticoid receptor agonist | |
| 2396 | -75.11 | cp | raltegravir | HIV integrase inhibitor | HIV integrase inhibitor | |
| 2397 | -75.67 | cp | quinoclamine | Algicide | Algicide |  |
| 2398 | -75.88 | cp | NPI-2358 | Tubulin inhibitor | Tubulin inhibitor | |
| 2399 | -76.37 | cp | ethisterone | Progestogen hormone | Progestogen hormone | |
| 2400 | -76.42 | cp | 5'-guanidinonaltrindole | Opioid receptor antagonist | Opioid receptor antagonist | |
| 2401 | -76.49 | cp | homochlorcyclizine | Antihistamine | Antihistamine | |
| 2402 | -76.65 | cp | deforolimus | MTOR inhibitor | MTOR inhibitor | |
| 2403 | -77.96 | cp | dexketoprofen | Cyclooxygenase inhibitor | Cyclooxygenase inhibitor | |
| 2404 | -78.9 | cp | depomedrol | Glucocorticoid receptor agonist | Glucocorticoid receptor agonist | |
| 2405 | -80.05 | cp | deferiprone | Chelating agent | Chelating agent | |
| 2406 | -81.28 | cp | alfacalcidol | Vitamin D receptor agonist | Vitamin D receptor agonist | |
| 2407 | -81.88 | cp | rhodomyrtoxin | Cytotoxic agent | Cytotoxic agent, DNA intercalator | |
| 2408 | -83.63 | cp | sappanone-a | Tyrosinase inhibitor | Tyrosinase inhibitor, Melanin inhibitor | |
| 2409 | -83.68 | cp | flubendazole | Tubulin inhibitor | Tubulin inhibitor | |
| 2410 | -87.19 | cp | HNHA | HDAC inhibitor | HDAC inhibitor | |
| 2411 | -87.94 | cp | ON-01910 | PLK inhibitor | PLK inhibitor | |
| 2412 | -88.98 | cp | nocodazole | Tubulin inhibitor | Tubulin inhibitor | |
| 2413 | -89.64 | cp | phorbol-12-myristate-13-acetate | PKC activator | PKC activator | |
| 2414 | -90.87 | cp | kinetin-riboside | Apoptosis stimulant | Apoptosis stimulant | |
| 2415 | -91.52 | cp | ABT-751 | Tubulin inhibitor | Tubulin inhibitor | |
| 2416 | -92.07 | cp | QW-BI-011 | Histone lysine methyltransferase inhibitor | Histone lysine methyltransferase inhibitor | |
| 2417 | -93.45 | cp | PAC-1 | Caspase activator | Caspase activator | |
| 2418 | -94.23 | cp | SAL-1 | Adenosine receptor antagonist | Adenosine receptor antagonist | |
| 2419 | -94.33 | cp | VU-0365114-2 | M5 modulator | M5 modulator | |
| 2420 | -94.72 | cp | ingenol | PKC activator | PKC activator | |
| 2421 | -94.77 | cp | HO-013 | PPAR receptor agonist | PPAR receptor agonist | |
| 2422 | -95.81 | cp | U-0124 | MEK inhibitor | MEK inhibitor | |
| 2423 | -96.41 | cp | prostratin | PKC activator | PKC activator | |
