# Supplementary figures and images for: Exploring prognostic and immunological characteristics of pancreatic ductal adenocarcinoma through comprehensive genomic analysis of tertiary lymphoid structures and CD8 + T-cells
Source: J Cancer Res Clin Oncol. 2024 Jun 8;150(6):300. doi: 10.1007/s00432-024-05824-0 (PMC11162401; doi:10.1007/s00432-024-05824-0)

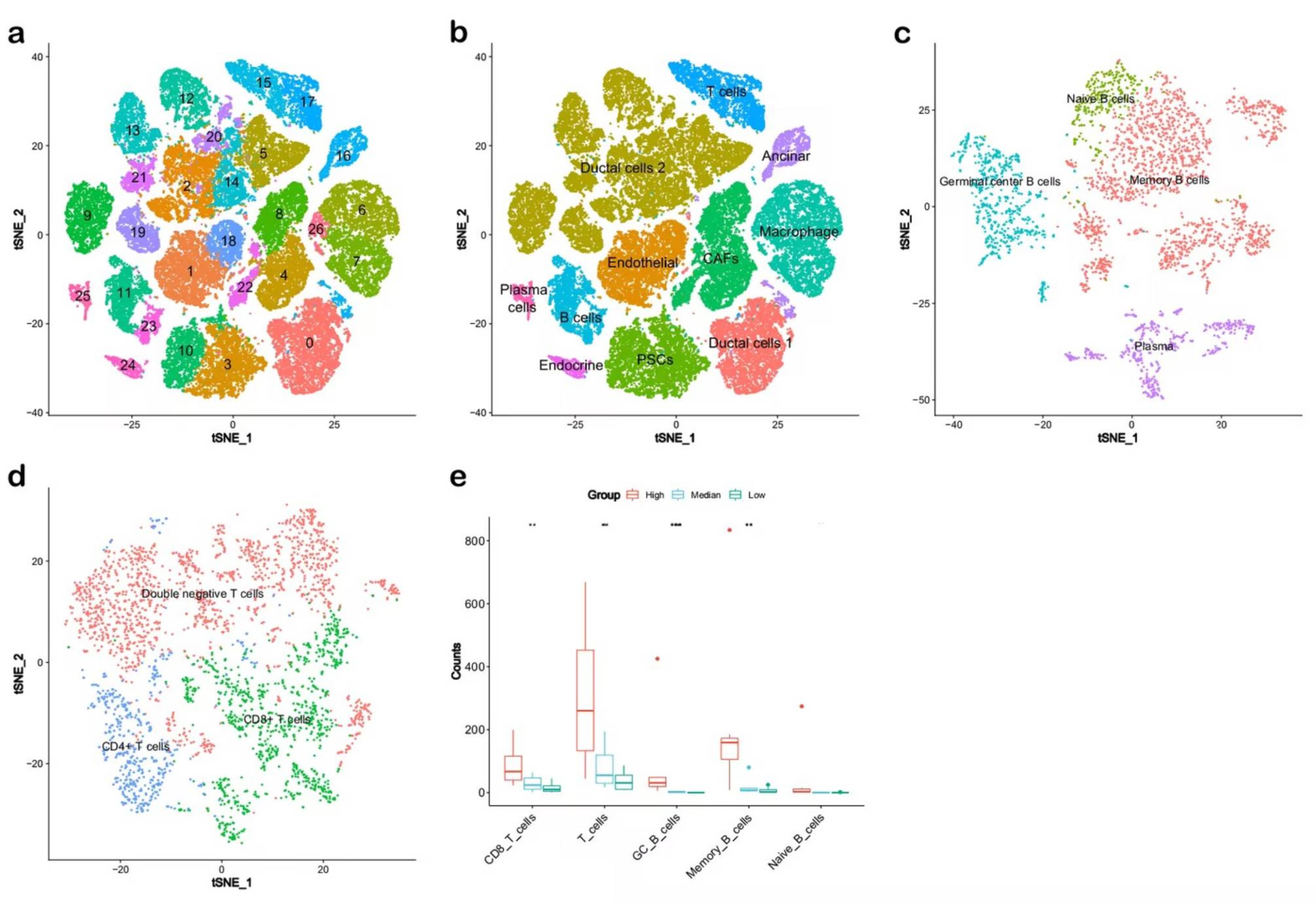

Supplement: Supplementary file 5 — Supplementary Material 5 [file 432_2024_5824_MOESM5_ESM.jpg]

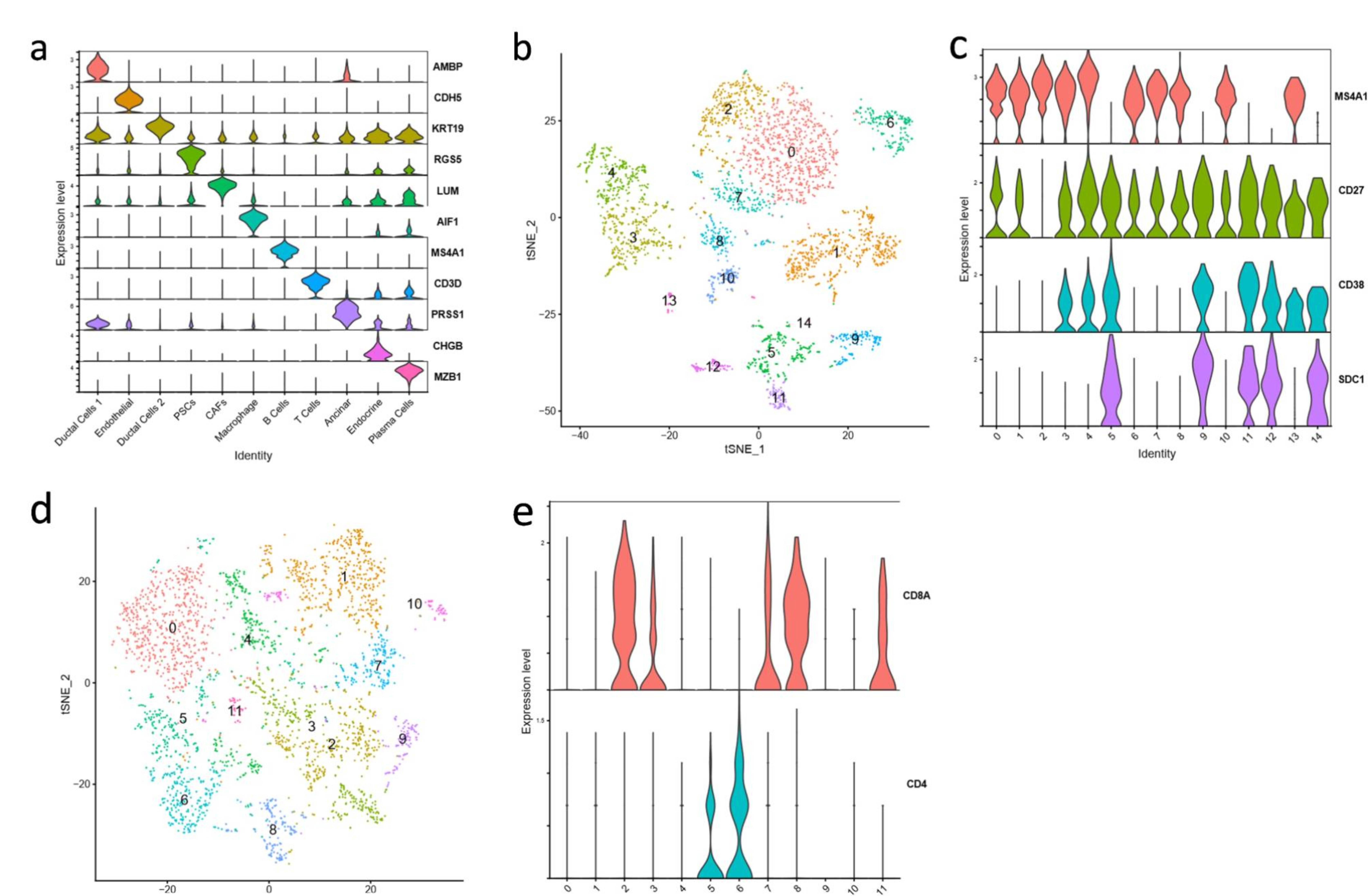

Supplement: Supplementary file 6 — Supplementary Material 6 [file 432_2024_5824_MOESM6_ESM.jpg]

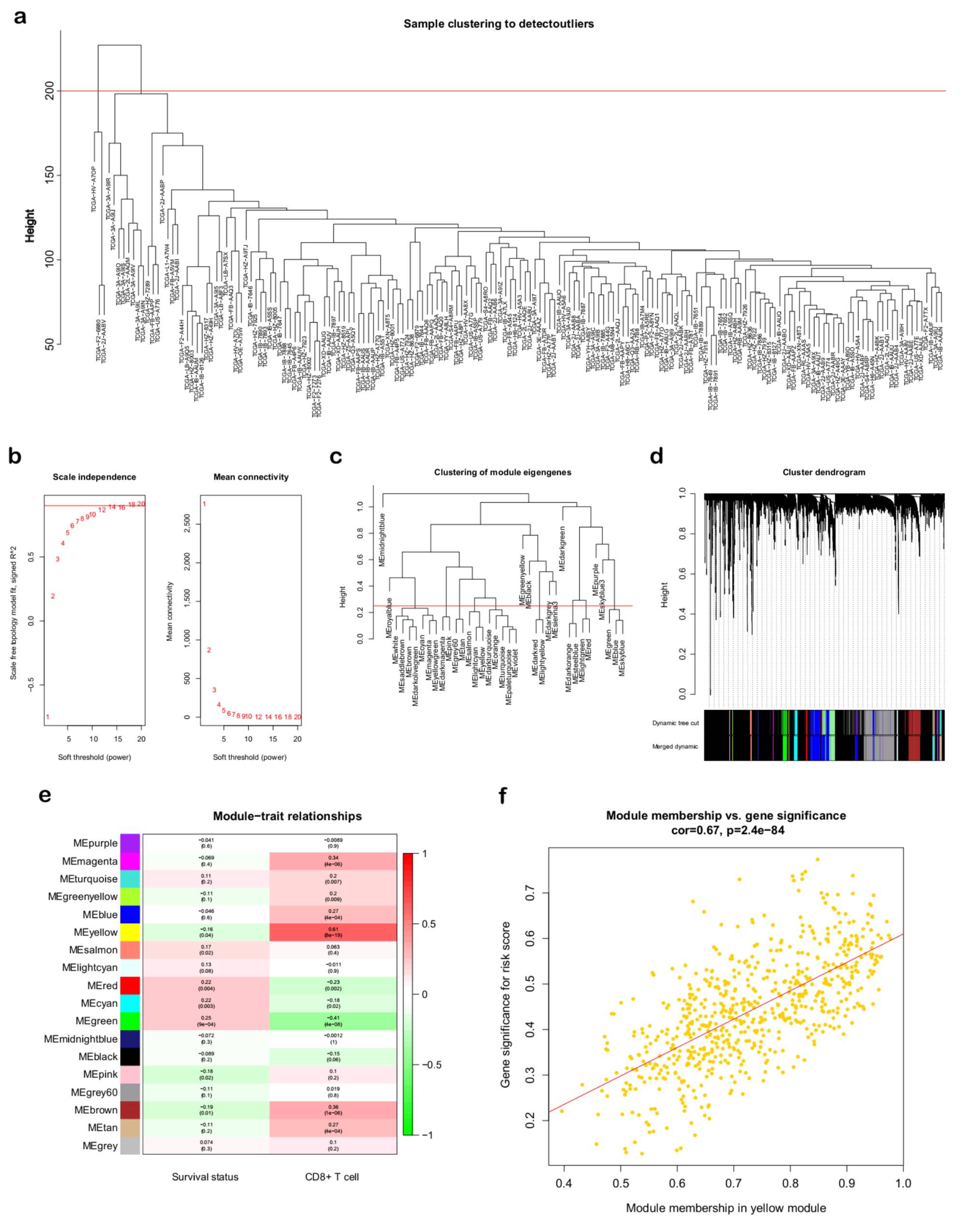

Supplement: Supplementary file 7 — Supplementary Material 7 [file 432_2024_5824_MOESM7_ESM.jpg]

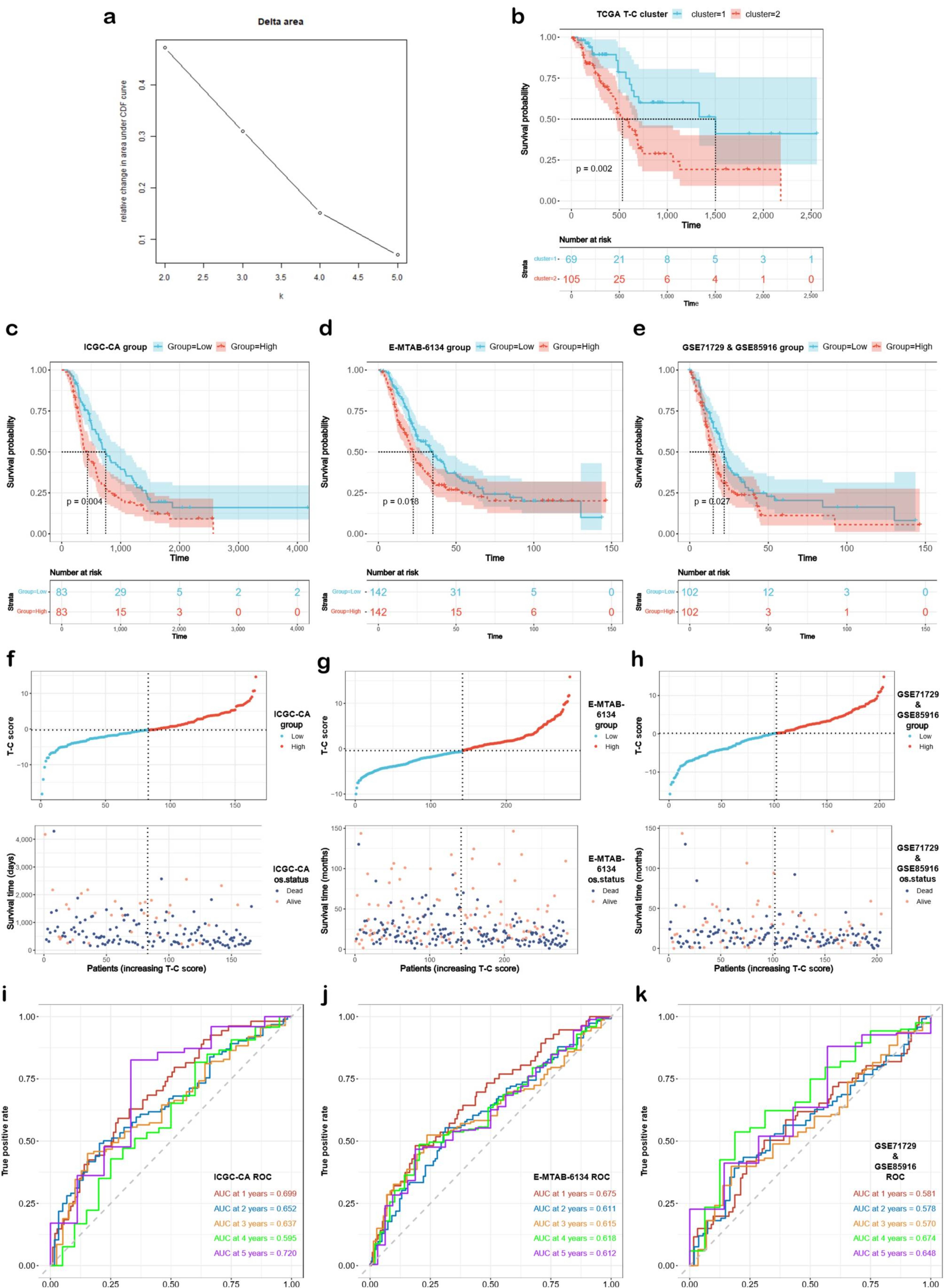

Supplement: Supplementary file 8 — Supplementary Material 8 [file 432_2024_5824_MOESM8_ESM.jpg]

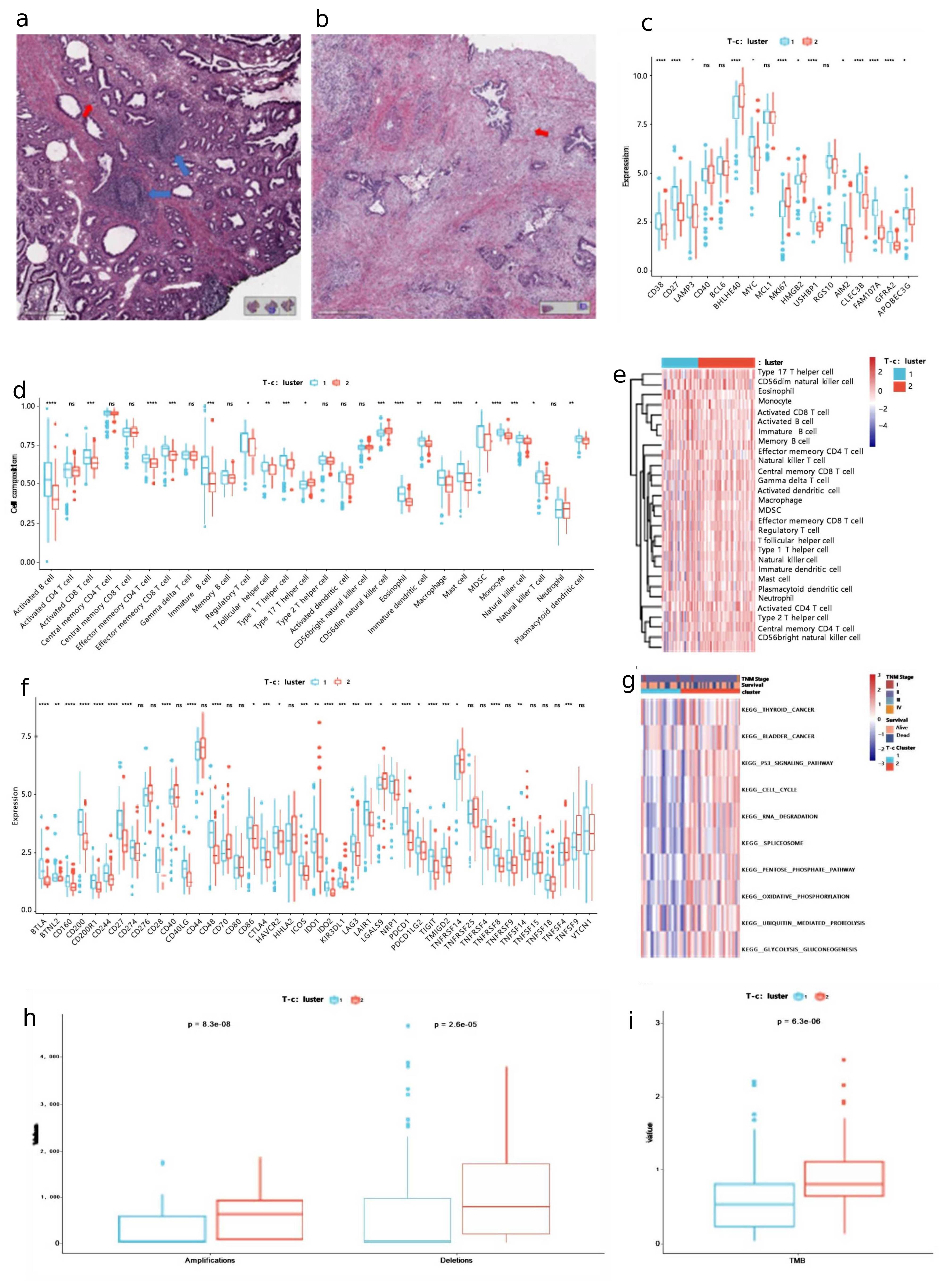

Supplement: Supplementary file 9 — Supplementary Material 9 [file 432_2024_5824_MOESM9_ESM.jpg]

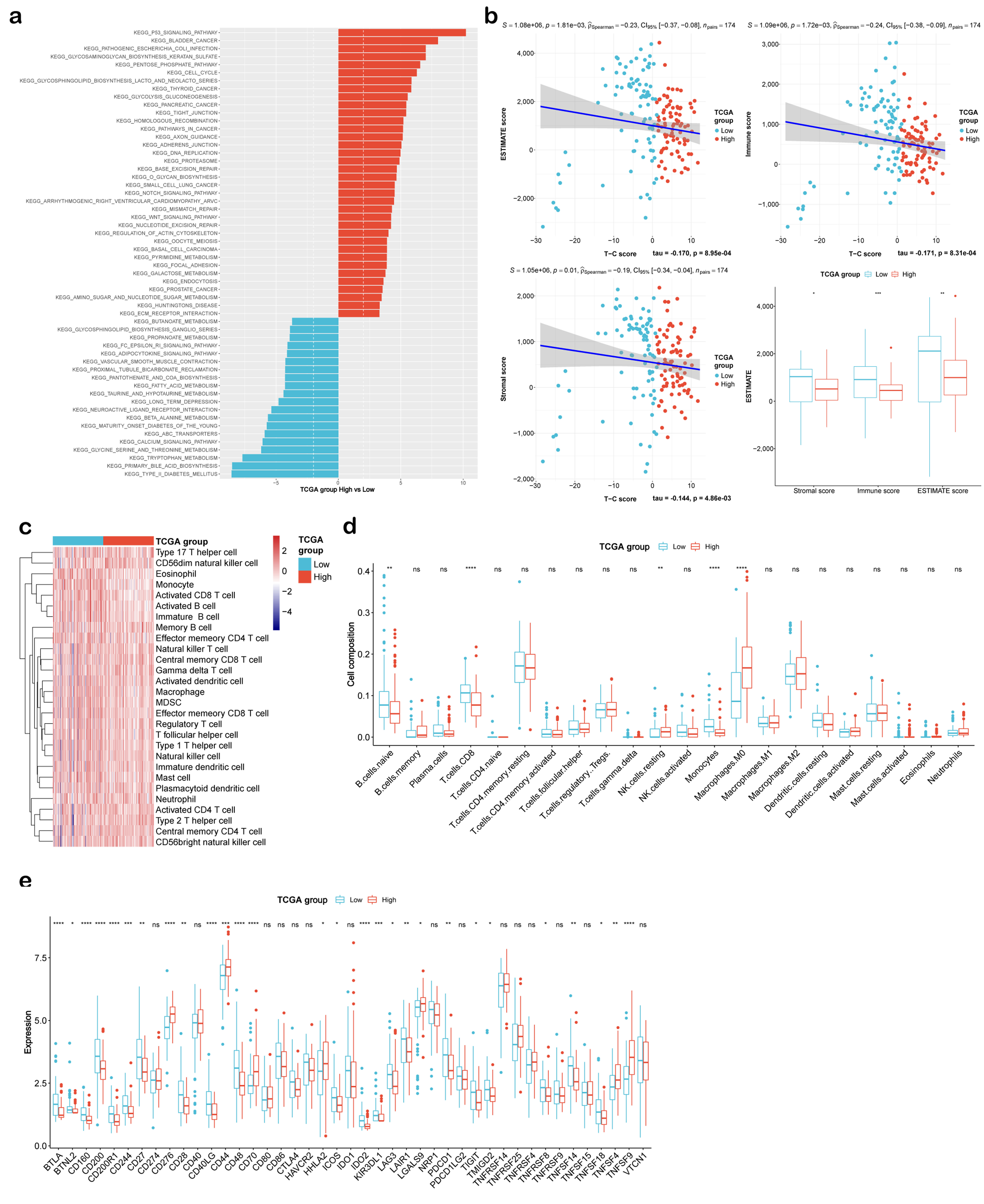

Supplement: Supplementary file 10 — Supplementary Material 10 [file 432_2024_5824_MOESM10_ESM.jpg]

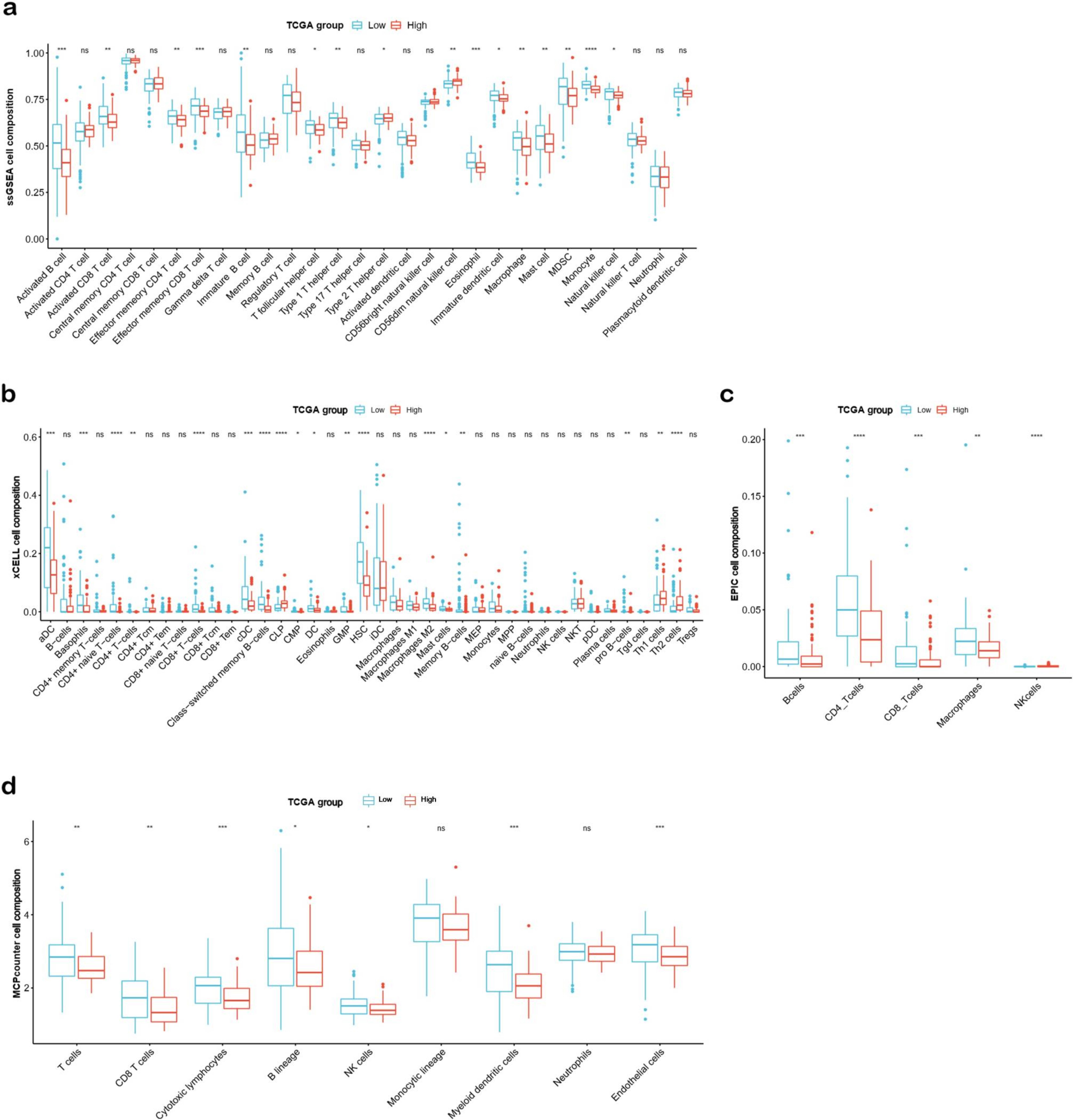

Supplement: Supplementary file 11 — Supplementary Material 11 [file 432_2024_5824_MOESM11_ESM.jpg]

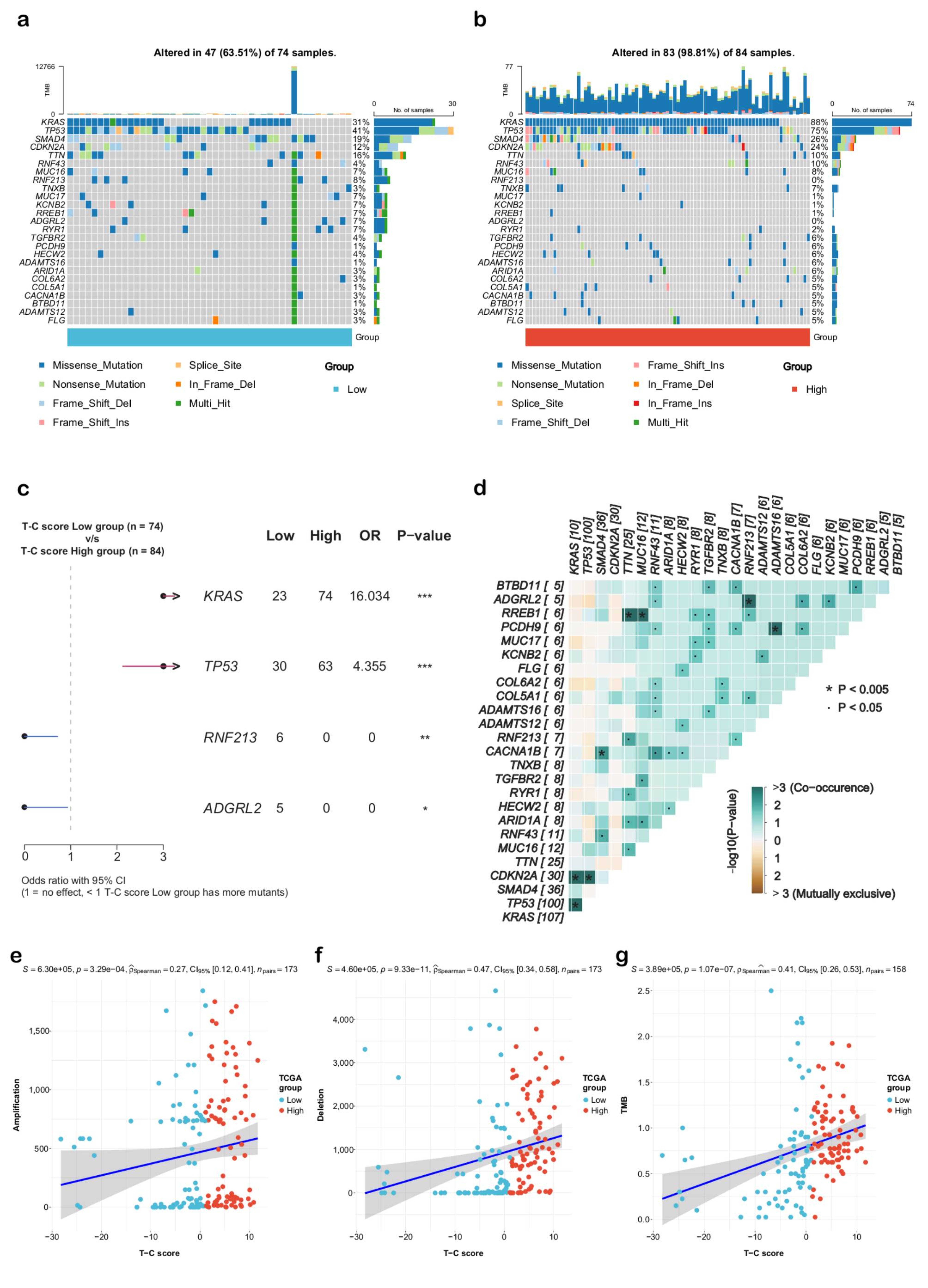

Supplement: Supplementary file 12 — Supplementary Material 12 [file 432_2024_5824_MOESM12_ESM.jpg]

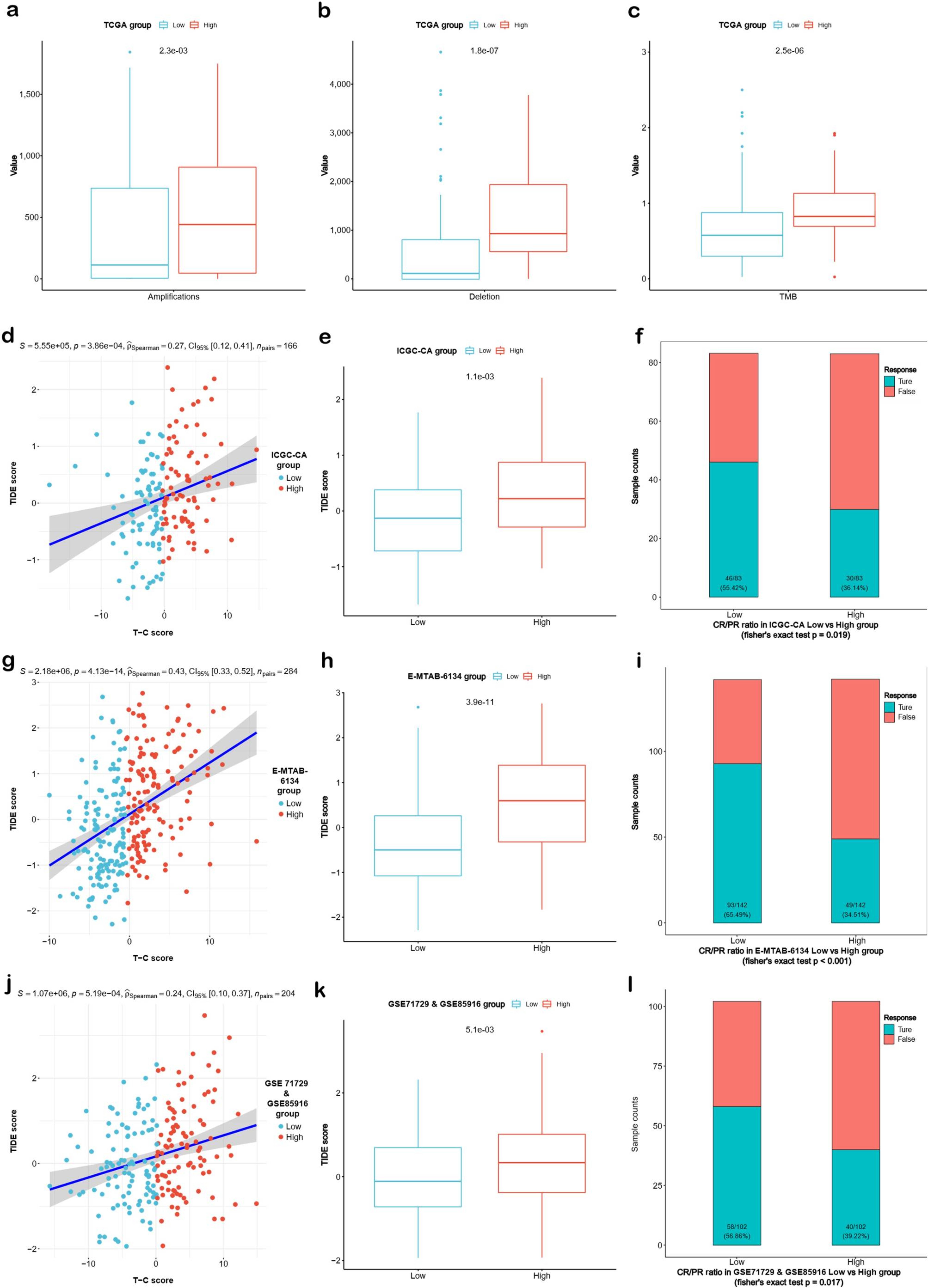

Supplement: Supplementary file 13 — Supplementary Material 13 [file 432_2024_5824_MOESM13_ESM.jpg]

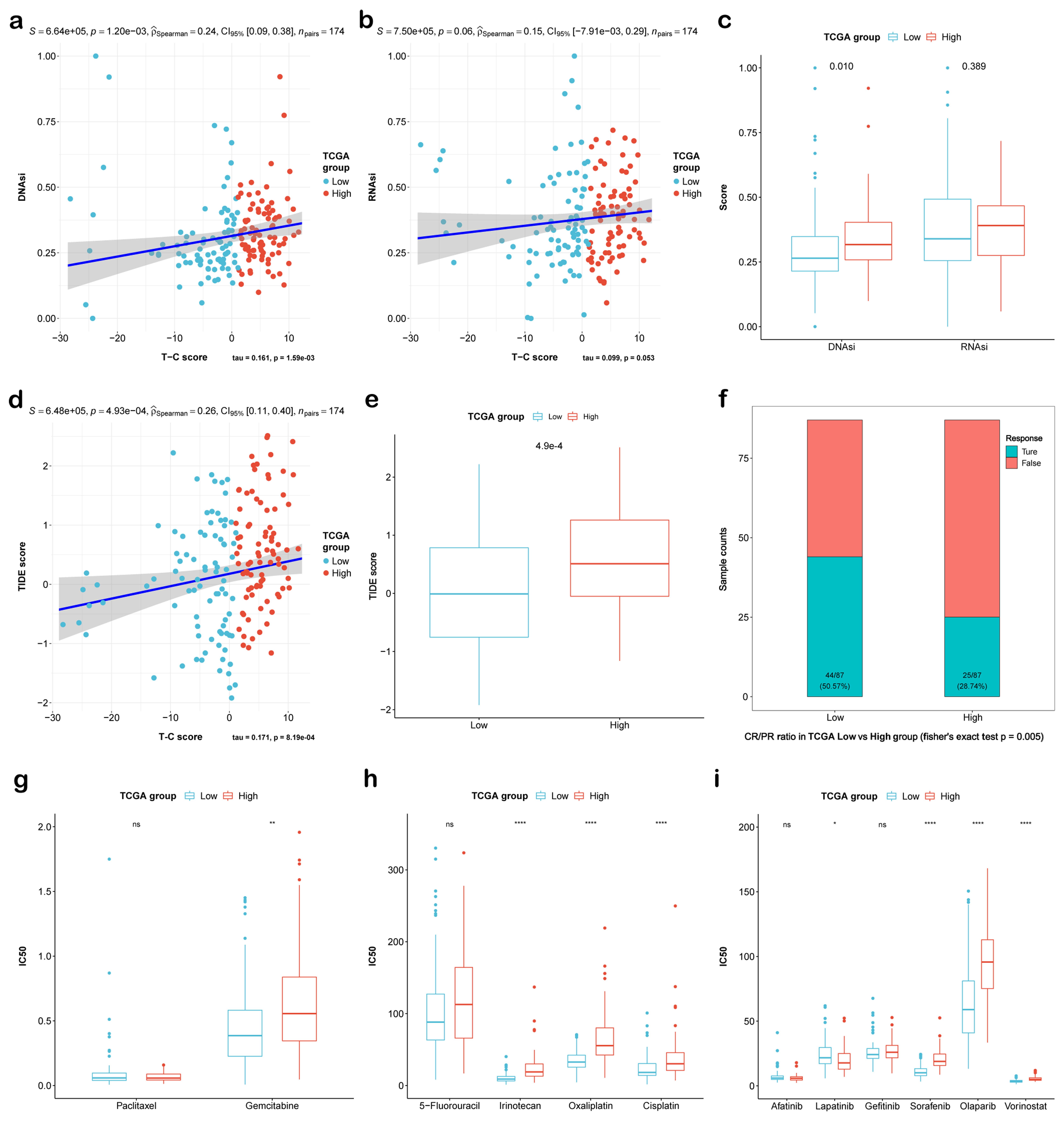

Supplement: Supplementary file 14 — Supplementary Material 14 [file 432_2024_5824_MOESM14_ESM.jpg]

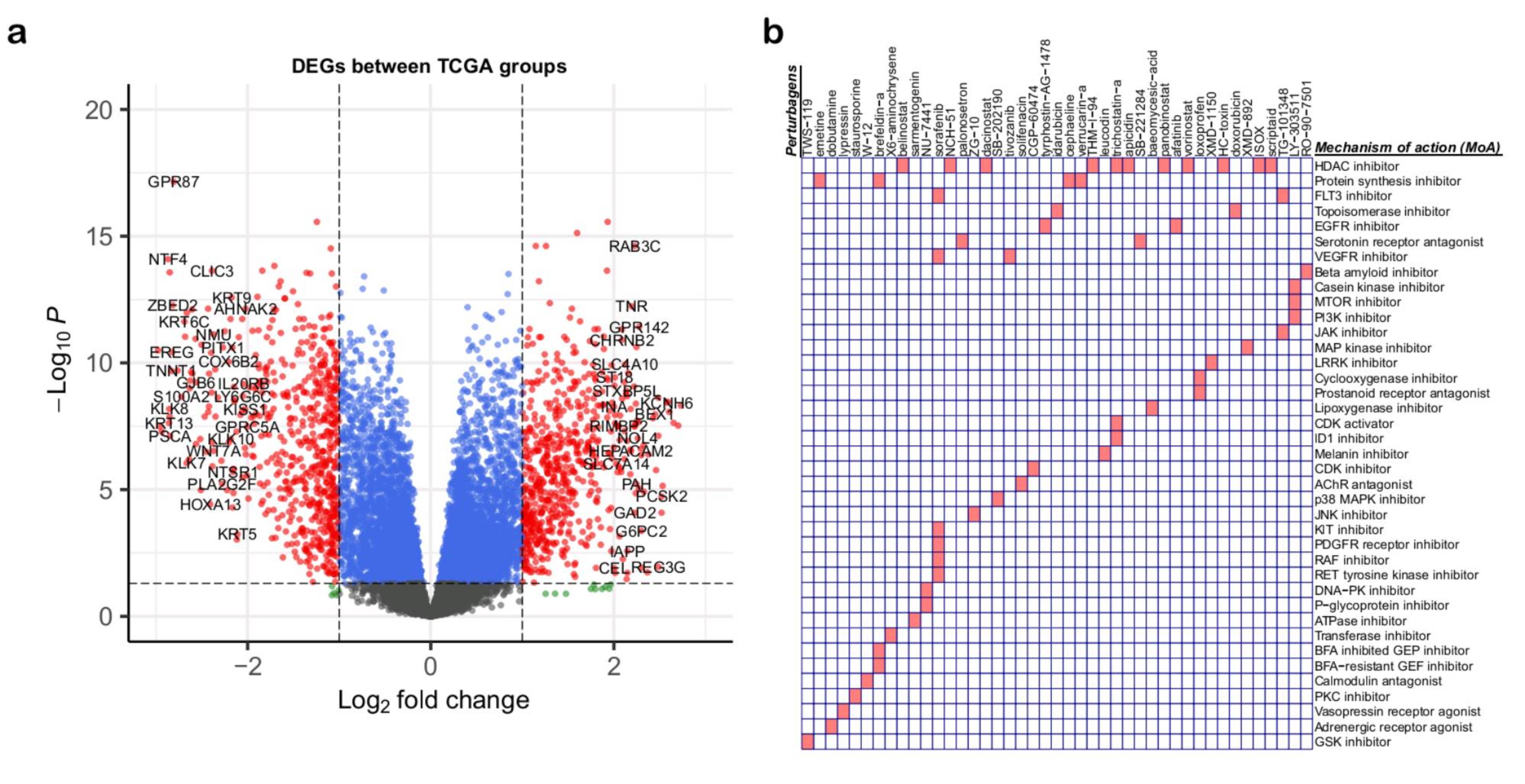

Supplement: Supplementary file 15 — Supplementary Material 15 [file 432_2024_5824_MOESM15_ESM.jpg]

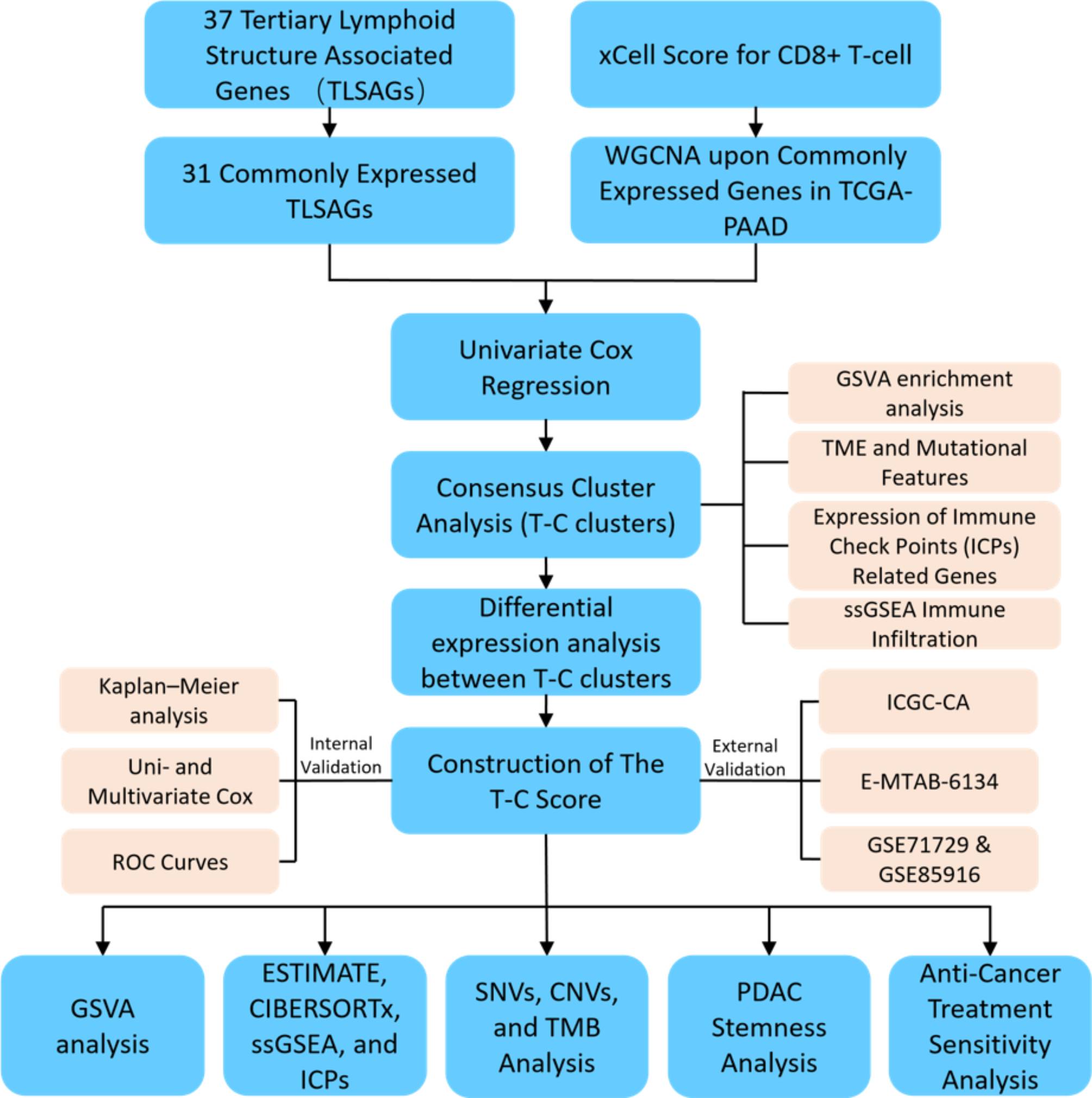

Supplement: Supplementary file 16 — Supplementary Material 16 [file 432_2024_5824_MOESM16_ESM.jpg]
