# Supplementary material for: Exploring prognostic and immunological characteristics of pancreatic ductal adenocarcinoma through comprehensive genomic analysis of tertiary lymphoid structures and CD8 + T-cells
Source: J Cancer Res Clin Oncol. 2024 Jun 8;150(6):300. doi: 10.1007/s00432-024-05824-0 (PMC11162401; doi:10.1007/s00432-024-05824-0)
Supplement: Supplementary file 17 — Supplementary Material 17 [file 432_2024_5824_MOESM17_ESM.docx]

**2 Materials and Methods**

A preprint has previously been published (Hu et al. 2023).

**2.1 Single-Cell RNA Sequencing Datasets Acquisition and Processing**

The single-cell RNA sequencing (scRNA-seq) data of tumor samples from 24 patients with PDAC was obtained from the Genome Sequence Archive (accession number CRA001160) and analyzed using the Seurat v4.1.0 R toolkit (Satija et al. 2015). The gene-cell matrixes were preprocessed by removing cells with low transcript counts (<200 transcripts/cell) or high mitochondrial gene expressions (>10%) and genes with low expression levels (detected in <3 cells). We applied the FindVariableFeatures and ScaleData functions to select variable genes and normalize gene expression values, respectively. The data was then transformed using the Seurat function SCTransform with the percent of mitochondrial expression and cell cycle score as regression factors (Hafemeister and Satija 2019). We employed JackStraw analysis to identify significant principal components (PCs) and visualized the heatmap focusing on PCs 1 to 50. For graph-based clustering, we used PCs 1 to 30 and a resolution of 1, resulting in 26 cell clusters that were projected to t-distributed stochastic neighbor embedding (t-SNE) dimensionality reduction. The FindAllMarkers functions were used to identify marker genes in different groups, and according to Junya Peng et al. (Peng et al. 2019), well-established cell type markers were used in characterizing the identities of cell types of these groups. B-cells were subsequently classified into distinct subpopulations: naïve B-cells (MS4A1+, CD27-, and CD38-), memory B-cells (MS4A1+, CD27+, and CD38-), germinal center (GC) B-cells (MS4A1+, CD27+, CD38+, and SDC1-), plasma cells (MS4A1-, CD27+, CD38+, and SDC1+) (Xia et al. 2023), the T-cells were subdivided into CD8+ T-cells, CD4+ T-cells, and double negative T-cells (CD4-, CD8-).

**2.2 Bulk RNA Sequencing Datasets Acquisition and Processing**

The STAR raw counts data and the related clinical information of PDAC patients were obtained from the PAAD cohort in The Cancer Genome Atlas (TCGA) database (https://portal.gdc.cancer.gov/projects/TCGA-PAAD/). For subsequent analyses, fragments per kilobase million (FPKM) values were downloaded from the TCGA database and then converted to transcripts per kilobase million (TPM) values. The TPM data was then transformed into log2(TPM+1) for further usage, and patients who had no survival data or 0 days of survival time were excluded. For further validation, the transcriptional data and the corresponding clinical data were obtained from PDAC cases in the International Cancer Genome Consortium (ICGC) database (ICGC-CA, https://dcc.icgc.org/releases/current/Projects/PACA-CA) and the ArrayExpress database (E-MTAB-6134, https://www.ebi.ac.uk/arrayexpress/experiments/E-MTAB-6134), the expression data from PDAC cases in GSE71729 and GSE85916 were downloaded from Gene Expression Omnibus (GEO) database (GSE71729, https://www.ncbi.nlm.nih.gov/geo/query/acc.cgi?acc=GSE71729/; GSE85916, https://www.ncbi.nlm.nih.gov/geo/query/acc.cgi?acc=GSE85916/). The ComBat function in R package ‘sva’ was used to correct batch effect between different datasets (Johnson et al. 2007).

**2.3 Tertiary Lymphoid Structure Associated Genes (TLSAGs)**

Through a review of previously published studies and bioinformatics analysis (Cabrita et al. 2020; Feng et al. 2021; Horeweg et al. 2022; Zhou et al. 2021), we identified 37 tertiary lymphoid structure-associated genes (TLSAGs). These 37 TLSAGs were then cross-referenced for intersections with commonly expressed genes in the above databases, identifying 31 of the TLSAGs selected for the subsequent analyses.

**2.4 Acquisition of CD8+ T Cell Enrichment Score and Weighted Correlation Network Analysis (WGCNA) of CD8+ T Cell Related Genes (****CTCRGs)**

A comprehensive set of 64 immune and non-immune cell types within the tumor microenvironment (TME) of TCGA-PAAD samples was obtained from xCell (http://xcell.ucsf.edu/). xCell is an enrichment algorithm comprising 6573 gene signatures for 64 immune and non-immune cell types (Aran et al. 2017). The enrichment score for CD8+ T cell was extracted for WGCNA (Zhang and Horvath 2005), which was conducted using the “WGCNA” R package to discern CD8+ T cell-related genes (CTCRGs) within the TCGA cohort (Wu et al. 2021). Pearson’s correlation for all pairwise gene combinations was employed to generate the WGCNA adjacency matrix. The optimal soft threshold power of β = 12 was determined through the “pickSoftThreshold” function to achieve a scale-free topology of the adjacency matrix. The adjacency matrix was then converted into a topological overlap matrix (TOM). Based on TOM-derived dissimilarity measures, with a minimum module size of 30 and cut height of 0.25, CTCRGs exhibiting similar expression patterns were grouped into the same gene module via average linkage hierarchical clustering. Subsequently, the correlation between module eigengenes (MEs) and the enrichment score for CD8+ T cells was examined to identify CTCRGs.

**2.5 Unsupervised Cluster Analysis**

Univariate Cox regression analysis was executed utilizing the “survival” R package to choose genes from both CTCRGs and ubiquitously expressed TLSAGs. Genes with p < 0.01 were selected for unsupervised cluster analysis. Subsequently, consensus clustering was conducted employing the “ConsensusClusterPlus” R package (Wilkerson and Hayes 2010). To ensure classification stability, parameters were set: the maximum k value at 5, repetitions at 1000, pItem at 0.8, and pFeature at 1. Additionally, partitioning around medoids (PAM) served as the clustering algorithm. (Hong et al. 2022; Yu et al. 2022).

**2.6 Construction and Validation of the T-C Score**

The “limma” R package was used to identify the differentially expressed genes (DEGs) across subtypes with the cutoff values as |log2 fold change (FC)| > 1 and adjusted.p < 0.05. Following this, unsupervised clustering analysis was conducted to categorize PDAC patients in the TCGA-PAAD cohort into discrete gene clusters based on the DEGs. The Boruta algorithm was employed to reduce the dimensions of the T-C gene signatures A and B with the settings: doTrace = 2 and maxRuns = 500 (Kursa 2010), while principal component analysis was utilized to extract PC1 as the signature score. The T-C score for each patient was then calculated using the equation: T-C score = ∑PC1A - ∑PC1B. In this equation, PC1A represents the first component of signature A, and PC1B denotes the first component of signature B. Patients in the TCGA cohort were subsequently stratified into low- and high-score groups based on the median T-C score. Kaplan–Meier survival curves were then plotted using the “survminer” R package, and survival-dependent receiver operating characteristic (ROC) curves for 1 to 5 years were generated using the “survivalROC” R package. Both univariate and multivariate Cox regression analyses were performed to validate the independent prognostic value of the T-C score. External validation of the T-C score was conducted in ICGC-CA, E-MTAB-6134, and the combined GSE71729 and GSE85916 cohort, employing the median T-C score as the cutoff point.

**2.7 Functional Enrichment Analysis**

Annotated gene sets “c2.cp.kegg.v2022.1.Hs.symbols.gmt” were downloaded from the GSEA website (<http://www.gsea-msigdb.org/gsea/downloads.jsp>). A gene set variation analysis (GSVA) was performed to identify differences in biological function between the T-C subtypes and the T-C score groups (Hanzelmann et al. 2013).

**2.8 Somatic Mutations (SNVs) and Copy-Number Variations (CNVs) Data**

The pertinent data on single nucleotide variants (SNVs) and copy number variants (CNVs) for TCGA-PAAD patients were obtained from the TCGA website. The SNVs were subsequently analyzed and visualized using the “maftools” R package (Mayakonda et al. 2018), and the CNVs of TCGA-PAAD patients were analyzed using the GISTIC 2.0 online version (<https://cloud.genepattern.org/gp/pages/index.jsf>). Additionally, the GSCA Lite database (http://bioinfo.life.hust.edu.cn/web/GSCA Lite/) (Liu et al. 2018), an online platform designed for Gene Set Cancer Analysis, was utilized to perform statistical analysis of heterozygous and homozygous CNV deletions and amplifications.

**2.9 Calculation of RNA stemness score (RNAsi) and DNA stemness score (DNAsi)**

The OCLR algorithm was employed to calculate the RNA-based stemness index (RNAsi) and the DNA methylation-based stemness index (DNAsi) (Malta et al. 2018). The workflow for generating these stemness indices (RNAsi and DNAsi) can be found at https://bioinformaticsfmrp.github.io/PanCanStem_Web/.

**2.10 Collection and Analysis of Immune-Related Data**

The immune cell infiltration for each PDAC sample in the TCGA-PAAD cohort was assessed using the CIBERSORTx web tool (https://cibersortx.stanford.edu/) based on the LM22 signature (Newman et al. 2019). The genetic signatures of 28 immune cells were obtained from the work of Charoentong et al. (Charoentong et al. 2017). A single-sample gene set enrichment analysis (ssGSEA) was then conducted to quantitatively assess the relative infiltration levels of 28 immune cells within the PDAC TME (Bindea et al. 2013). Immune cell infiltration analyses were also carried out using xCell (Aran et al. 2017), MCPcounter (Becht et al. 2016), and EPIC algorithms (Racle and Gfeller 2020). Furthermore, the ESTIMATE algorithm was employed to compute the ESTIMATE, immune, and stromal scores for each patient in the TCGA-PAAD cohort (Malta et al. 2018).

**2.11 Prediction of Therapeutic Benefits for Patients in Distinct T-C Score Group**

The Tumor Immune Dysfunction and Exclusion (TIDE) algorithm, a computational approach, was developed to predict patient responses to ICBs (He et al. 2018). The TIDE web module (http://tide.dfci.harvard.edu) was employed to assess the T-C score’s capacity to predict therapeutic responses to ICBs in PDAC patients from the TCGA-PAAD cohort (Pan et al. 2022; Qiu et al. 2021; Yu et al. 2022). Moreover, sensitivities to chemotherapeutic agents, such as paclitaxel, gemcitabine, 5-Fluorouracil, irinotecan, oxaliplatin, cisplatin, and multikinase inhibitors, including gefitinib, sorafenib, and olaparib, were estimated using the “oncoPredict” R package based on the Genomics of Drug Sensitivity in Cancer (GDSC) (Maeser et al. 2021; Yang et al. 2013). To compare therapeutic sensitivities between T-C score groups, the estimated half-maximal inhibitory concentration (IC50) for each patient in the TCGA cohort was calculated using ridge regression, and the accuracy of this prediction was assessed via tenfold cross-validation. The Connectivity Map database (CMap, https://clue.io/query) is a web-based tool to predict small molecules targeting cancer-related genes based on gene expression profiles (Lamb et al. 2006). To identify potential small molecular drugs for PDAC patients between the T-C score groups, DEGs between the high and low T-C score groups were determined using the ‘limma’ package and inputted into the CMap database. The CMap mode-of-action (MoA) analysis was also conducted to elucidate the underlying mechanisms of drug actions (Subramanian et al. 2017). The workflow of this study is demonstrated in Figure S12.

**2.12 Statistical Analysis**

All statistical analyses in this study were conducted using the R software (version 4.2.1). Comparisons between the two groups were conducted using the Wilcoxon test. The correlation coefficients were calculated through Spearman analysis after the nonnormal distribution was proved by the Shapiro-Wilks test. Kendall's Tau method was used for verification. Survival analysis for each dataset was conducted using the Kaplan–Meier analysis and log-rank test. The R package “survival” was used for univariate and multivariate Cox regression analyses. Unless stated otherwise, the statistical significance was considered with a two-tailed p < 0.05.**3. Results**

**3.1 The Role of B-cells in PDAC Cancer**

The role of B cells in PDAC cancer patients is an important area of study. In many different cancer types, B cell signatures are enriched in the tumors of patients who respond to ICB treatment compared to non-responding patients, and B cell markers are DEGs in the tumors of responders (Helmink et al. 2020). Therefore, it is vital to understand this in PDAC patients. The scRNA-seq profile was generated from 24 PDAC samples. After quality control, we acquired single-cell transcriptomes in a total of 53,395 PDAC cells, then we applied principal component analysis (PCA) on variably expressed genes across all cells, and 26 segregated cell groups were identified and visualized via t-SNE plot (Figure S1a). Then according to Junya Peng et al.(Peng et al. 2019), we identified cell types based on the expression of known markers for AMBP, CFTR, MMP7 (ductal cell 1), KRT19, KRT7, TSPAN8, SLPI (ductal cell 2), PRSS1, CTRB1, CTRB2, REG1B (acinar), CHGB, CHGA, INS, IAPP (endocrine cell), RGS5, ACTA2, PDGFRB, ADIRF (stellate cell), LUM, DCN, COL1A1 (fibroblast), CDH5, PLVAP, VWF, CLDN5 (endothelial cell), AIF1, CD64, CD14, CD68 (macrophage), CD3D, CD3E, CD4, CD8 (T cell), MS4A1, CD79A, CD79B, CD52 (B cell) and 11 cell types were recognized (Figure S1b, Figure S2a). The B-cells and plasma cells were divided into 14 subgroups based on t-SNE analysis (Figure S2b). According to previously reported B-cell markers, 4 B-cell subtypes were identified, including naïve B-cells (MS4A1+, CD27-, and CD38-), memory B-cells (MS4A1+, CD27+, and CD38-), germinal center (GC) B-cells (MS4A1+, CD27+, CD38+, and SDC1-), plasma cells (MS4A1-, CD27+, CD38+, and SDC1+) (Figure S1c, Figure S2c). T-cells were also extracted and further divided into CD8+ T cells, CD4+ T cells, and double negative T cells (CD4-, CD8-) (Figure S1d, Figure S2d-e). The presence of activated B-cells and GC B-cells represented the possibility of ongoing TLS formation. Since GC B-cells are crucial for TLS formation and exist in the center of TLS, we further divided GC B-cells by PDAC sample; 10 of the 24 PDAC cases had 0 GC B-cells, 8 cases had a GC B-cell count of 6 or less, and 6 cases had a GC B-cell count of more than 6. Thus, we stratified the patients with 0 GC B-cells into the “GC Low” group, patients with GC B-cell counts less than 6 into the “GC Median” group, and the rest of the patients into the “GC High” group. A Wilcoxon test showed that patients in the ‘GC High’ group had significantly higher infiltration of naïve B-cells, memory B-cells, and CD8+ T cells (Figure S1e-f). This finding coincided with the notion that the presence of TLS was coupled with the higher infiltration of tumor-associated CD8+ T cells (Cabrita et al. 2020).

**3.2 Identification of CTCRGs in PDAC**

CD8+ T-cells have a vital role in immune escape and immune treatment in cancer therapy. Generally, CD8+ T-cell enrichment is beneficial against PDAC development, but some subpopulations of CD8+ T-cells can develop pro-tumorigenic subtypes (Picard et al. 2023). Therefore, it is important to understand the complexities of CTCRGs in PDAC. A total of 64 immune and non-immune cell types for TCGA PDAC samples were downloaded from xCell (<http://xcell.ucsf.edu/>), then the abundance of CD8+ T cells was extracted. To identify key modules that were significantly correlated with the abundance of CD8+ T cells, we performed WGCNA on the TCGA-PAAD cohort. Clustering dendrograms of 14776 commonly expressed genes in the TCGA-PAAD cohort are shown in Figure S3a. Scale-free fit index and mean connectivity for various soft-thresholding powers are shown in Figure S3b. By setting the cut height = 0.25 and b = 12 (scale-free R^2^ = 0.9), 14776 genes were divided into eighteen independent co-expression modules (Figure S3c-e). As shown in the relative diagram of the module-trait relationship (Figure S3e), the yellow module, including 638 CTCRGs, was the most significant feature, and correlation analysis showed that it correlated with the abundance of CD8+ T cells (R=0.670, p<0.001, Figure S3f).

**3.3 Different TME and Mutational Features of The Two Clusters**

The results so far seemed to support the view that TLSAGs and CTCRGs are related to the outcomes of the PDAC cases. So, we next investigated whether the apparent differences in the TME indicated by these genes were also evident at the cellular level. Through the TCGA pathology slides, we confirmed that TLSs and immune cell infiltration were greater in the tumors of T-C cluster-1 patients than in T-C cluster-2 patients (Figure 2b, Figure S5a). The differences in the expression of previously recognized T-C genes and CD38 and CD27, the marker genes for GC B-cells, are shown in Figure S5c. CD38, CD27, 2/8 of the TLSAGs, and 5/7 of CTCRGs were highly expressed in T-C cluster-1, indicating higher infiltration of TLS and CD8+ T-cells for T-C cluster-1. Additionally, we investigated the impact of TLSs and CD8+ T-cells on TIME. The ssGSEA analysis utilizing 28 immune cells and the analysis of the expression of 43 Immune Check Point (ICP) related genes were performed. The results demonstrated that T-C cluster-1 had more infiltrated immune cells (Figure S5c-d), with significantly higher expression in 26 ICP genes (Figure S5e). The GSVA showed T-C cluster-2 had significant enrichment in tumorigenesis-related pathways, such as KEGG_THYROID_CANCER, KEGG_BLADDER_CANCER, KEGG_P53_SIGNALING_PATHWAY, KEGG_CELL_CYCLE, KEGG_RNA_DEGRADATION, and KEGG_SPLICEOSOME (Figure S5f). Approximately 95% of PDAC patients have gene alterations (Pleasance et al. 2020). Therefore, the mutational landscape was investigated between the two clusters. It showed that the T-C cluster-2 exhibited significantly higher gene alterations involved in amplifications, deletions, and tumor mutation burden (TMB) (all p< 0.001, Figure S5f-h).

**3.4 Relationship Between T-C Score and PDAC Tumor Stemness**

Growing evidence has indicated that the expression of stemness-related genes in various tumors is positively correlated with drug resistance, cancer recurrence, and tumor proliferation (Chen et al. 2021). Therefore, we calculated the stemness scores for PDAC patients in the TCGA cohort using the OCLR algorithm recently published by Malta et al. (Malta et al. 2018). Then, we assessed the correlation between the T-C score and the stemness score. The results revealed that the T-C score was significantly and positively correlated with DNAsi (R=0.24, p=0.001; tau=0.161, p=1.59e-03, Figure S10a), and the high T-C score group had significantly higher levels of DNAsi (p=0.010, Figure S10c). Although not significant, RNAsi also had a positive correlation with T-C score (R=0.15, p=0.060; tau=0.099, p=0.053, Figure S10b), and higher levels of RNAsi were also observed in the high T-C score group (p=0.389, Figure S10c).

**3.5 Relationship between T-C score and Anti-Cancer Treatment Sensitivity**

***3.5.1 Analyses across Databases***

The emergence of ICBs has brought new possibilities to anti-cancer treatment for numerous cancer types (Budimir et al. 2022). However, given the complex interactions between cancer cells and TIME, not all cancers respond to ICB treatment, and the heterogeneity of TME among patients plagued by the same cancer type also results in different responses to ICB therapy (Powles et al. 2014; Yi et al. 2022). Therefore, encouraged by the differences in immune cell infiltration and expression of ICPs between distinct T-C score groups, we speculated on the response to ICBs for the TCGA-PAAD, ICGC-CA, and E-MTAB-6134 cohorts via the TIDE web tool. Spearman correlation analysis showed that the T-C score was significantly and positively correlated with the TIDE score in the TCGA-PAAD cohort (R=0.26, p<0.001; tau=0.171, p=8.19e-04, Figure S10d), and patients in the high T-C score group had significantly higher levels of TIDE score (p<0.001, Figure S10e). As expected, the patients in the low T-C score group had a significantly higher response rate to the ICBs than the high T-C score group in the TCGA-PAAD cohort (p=0.005, Figure S10f). The same trend was also found in the ICGC-CA (Figure S9d-f), E-MTAB-6134 cohorts (Figure S9g-i), and GSE71729 & GSE85916 joint cohort (Figure S9j-l), this consistency across databases demonstrated the scientific basis and robustness of our classification strategy.

***3.5.2 Comparison of Various Drugs***

To date, systemic chemotherapy remains an indispensable part of the PDAC treatment strategy. The most widely used chemotherapy regimens are FOLFIRINOX and gemcitabine plus paclitaxel (Mizrahi et al. 2020), and with the development of multikinase inhibitors and histone deacetylase (HDAC) inhibitors, more choices are offered to the PDAC patients. Therefore, we further investigated the T-C score’s ability to deduce the response to the commonly used chemotherapeutic agents, multikinase inhibitors, and one HDAC inhibitor (vorinostat) in the TCGA-PAAD cohort. As a result, significantly lower IC50 levels of Gemcitabine, irinotecan, oxaliplatin, and cisplatin were discovered in the low T-C score group, which indicated that the patients in the low T-C score group might be more sensitive to chemotherapy (Figure S10g-h). Analyses of multikinase inhibitors revealed that the low T-C score group may be more susceptible to some multikinase inhibitors, including lapatinib, sorafenib, and olaparib (Figure S10i). The low T-C score group also observed a significantly lower estimated IC50 level of vorinostat, an HDAC inhibitor (Figure S10i).

***3.5.3 Usefulness of the T-C Score for Drug Selection***

Based on the DEGs between the two T-C score groups generated by ‘limma’ (Figure S11a), with |log2 FC| > 1, adjusted p < 0.05, the top 150 upregulated DEGs and 150 downregulated DEGs between the low and high T-C score groups were uploaded to the CMap database, to search for underlying drugs. As shown in Figure S11b, 43 potential small molecule drugs and 39 MoA were identified with CMap score > 60 (Table S4), in which the HDAC inhibitors accounted for the highest proportion among the inhibitors. In summary, these findings have indicated the potential value of the T-C score in selecting a more suitable therapeutic strategy for PDAC patients.

**References**

Aran D, Hu Z, Butte AJ (2017) xCell: digitally portraying the tissue cellular heterogeneity landscape. Genome Biol 18:220. <https://doi.org/10.1186/s13059-017-1349-1>

Becht E, Giraldo NA, Lacroix L et al (2016) Estimating the population abundance of tissue-infiltrating immune and stromal cell populations using gene expression. Genome Biol 17:218. <https://doi.org/10.1186/s13059-016-1070-5>

Bindea G, Mlecnik B, Tosolini M et al (2013) Spatiotemporal dynamics of intratumoral immune cells reveal the immune landscape in human cancer. Immunity 39:782-795. <https://doi.org/10.1016/j.immuni.2013.10.003>

Budimir N, Thomas GD, Dolina JS et al (2022) Reversing T-cell Exhaustion in Cancer: Lessons Learned from PD-1/PD-L1 Immune Checkpoint Blockade. Cancer Immunol Res 10:146-153. <https://doi.org/10.1158/2326-6066.Cir-21-0515>

Cabrita R, Lauss M, Sanna A et al (2020) Tertiary lymphoid structures improve immunotherapy and survival in melanoma. Nature 577:561-565. <https://doi.org/10.1038/s41586-019-1914-8>

Charoentong P, Finotello F, Angelova M et al (2017) Pan-cancer Immunogenomic Analyses Reveal Genotype-Immunophenotype Relationships and Predictors of Response to Checkpoint Blockade. Cell Rep 18:248-262. <https://doi.org/10.1016/j.celrep.2016.12.019>

Chen P, Hsu WH, Han J et al (2021) Cancer Stemness Meets Immunity: From Mechanism to Therapy. Cell Rep 34:108597. <https://doi.org/10.1016/j.celrep.2020.108597>

Feng H, Yang F, Qiao L et al (2021) Prognostic Significance of Gene Signature of Tertiary Lymphoid Structures in Patients With Lung Adenocarcinoma. Front Oncol 11:693234. <https://doi.org/10.3389/fonc.2021.693234>

Hafemeister C, Satija R (2019) Normalization and variance stabilization of single-cell RNA-seq data using regularized negative binomial regression. Genome Biol 20:296. <https://doi.org/10.1186/s13059-019-1874-1>

Hanzelmann S, Castelo R, Guinney J (2013) GSVA: gene set variation analysis for microarray and RNA-seq data. BMC Bioinformatics 14:7. <https://doi.org/10.1186/1471-2105-14-7>

He M, Jiang M, Zhou Y et al (2018) Impaired Gal-9 Dysregulates the PBMC-Induced Th1/Th2 Imbalance in Abortion-Prone Matings. J Immunol Res 2018:9517842. <https://doi.org/10.1155/2018/9517842>

Helmink BA, Reddy SM, Gao J et al (2020) B cells and tertiary lymphoid structures promote immunotherapy response. Nature 577:549-555. <https://doi.org/10.1038/s41586-019-1922-8>

Hong WF, Liu MY, Liang L et al (2022) Molecular Characteristics of T Cell-Mediated Tumor Killing in Hepatocellular Carcinoma. Front Immunol 13:868480. <https://doi.org/10.3389/fimmu.2022.868480>

Horeweg N, Workel HH, Loiero D et al (2022) Tertiary lymphoid structures critical for prognosis in endometrial cancer patients. Nat Commun 13:1373. <https://doi.org/10.1038/s41467-022-29040-x>

Hu H, Xu Y, Ai XN et al (2023) Comprehensive Genomic Analysis of the Prognostic and Immunological Characteristics of Tertiary Lymphoid structures and CD8+ T-cells in Pancreatic Ductal Adenocarcinoma, PREPRINT (Version 1) available at Research Square [<https://doi.org/10.21203/rs.3.rs-2860058/v1>].

Johnson WE, Li C, Rabinovic A (2007) Adjusting batch effects in microarray expression data using empirical Bayes methods. Biostatistics 8:118-127. <https://doi.org/10.1093/biostatistics/kxj037>

Kursa MB, Rudnicki, W. R. (2010) Feature Selection with the Boruta Package. J. Stat. Soft. 36:1-13. <https://doi.org/doi:10.18637/jss.v036.i11>

Lamb J, Crawford ED, Peck D et al (2006) The Connectivity Map: using gene-expression signatures to connect small molecules, genes, and disease. Science 313:1929-1935. <https://doi.org/10.1126/science.1132939>

Liu CJ, Hu FF, Xia MX et al (2018) GSCALite: a web server for gene set cancer analysis. Bioinformatics 34:3771-3772. <https://doi.org/10.1093/bioinformatics/bty411>

Maeser D, Gruener RF, Huang RS (2021) oncoPredict: an R package for predicting in vivo or cancer patient drug response and biomarkers from cell line screening data. Brief Bioinform 22:<https://doi.org/10.1093/bib/bbab260>

Malta TM, Sokolov A, Gentles AJ et al (2018) Machine Learning Identifies Stemness Features Associated with Oncogenic Dedifferentiation. Cell 173:338-354 e315. <https://doi.org/10.1016/j.cell.2018.03.034>

Mayakonda A, Lin DC, Assenov Y et al (2018) Maftools: efficient and comprehensive analysis of somatic variants in cancer. Genome Res 28:1747-1756. <https://doi.org/10.1101/gr.239244.118>

Mizrahi JD, Surana R, Valle JW et al (2020) Pancreatic cancer. Lancet 395:2008-2020. <https://doi.org/10.1016/S0140-6736(20)30974-0>

Newman AM, Steen CB, Liu CL et al (2019) Determining cell type abundance and expression from bulk tissues with digital cytometry. Nat Biotechnol 37:773-782. <https://doi.org/10.1038/s41587-019-0114-2>

Pan W, Song K, Zhang Y et al (2022) The molecular subtypes of triple negative breast cancer were defined and a ligand-receptor pair score model was constructed by comprehensive analysis of ligand-receptor pairs. Front Immunol 13:982486. <https://doi.org/10.3389/fimmu.2022.982486>

Peng J, Sun BF, Chen CY et al (2019) Single-cell RNA-seq highlights intra-tumoral heterogeneity and malignant progression in pancreatic ductal adenocarcinoma. Cell Res 29:725-738. <https://doi.org/10.1038/s41422-019-0195-y>

Picard FSR, Lutz V, Brichkina A et al (2023) IL-17A-producing CD8(+) T cells promote PDAC via induction of inflammatory cancer-associated fibroblasts. Gut 72:1510-1522. <https://doi.org/10.1136/gutjnl-2022-327855>

Pleasance E, Titmuss E, Williamson L et al (2020) Pan-cancer analysis of advanced patient tumors reveals interactions between therapy and genomic landscapes. Nat Cancer 1:452-468. <https://doi.org/10.1038/s43018-020-0050-6>

Powles T, Eder JP, Fine GD et al (2014) MPDL3280A (anti-PD-L1) treatment leads to clinical activity in metastatic bladder cancer. Nature 515:558-562. <https://doi.org/10.1038/nature13904>

Qiu C, Shi W, Wu H et al (2021) Identification of Molecular Subtypes and a Prognostic Signature Based on Inflammation-Related Genes in Colon Adenocarcinoma. Front Immunol 12:769685. <https://doi.org/10.3389/fimmu.2021.769685>

Racle J, Gfeller D (2020) EPIC: A Tool to Estimate the Proportions of Different Cell Types from Bulk Gene Expression Data. Methods Mol Biol 2120:233-248. <https://doi.org/10.1007/978-1-0716-0327-7_17>

Satija R, Farrell JA, Gennert D et al (2015) Spatial reconstruction of single-cell gene expression data. Nat Biotechnol 33:495-502. <https://doi.org/10.1038/nbt.3192>

Subramanian A, Narayan R, Corsello SM et al (2017) A Next Generation Connectivity Map: L1000 Platform and the First 1,000,000 Profiles. Cell 171:1437-1452 e1417. <https://doi.org/10.1016/j.cell.2017.10.049>

Wilkerson MD, Hayes DN (2010) ConsensusClusterPlus: a class discovery tool with confidence assessments and item tracking. Bioinformatics 26:1572-1573. <https://doi.org/10.1093/bioinformatics/btq170>

Wu X, Jiang D, Liu H et al (2021) CD8(+) T Cell-Based Molecular Classification With Heterogeneous Immunogenomic Landscapes and Clinical Significance of Clear Cell Renal Cell Carcinoma. Front Immunol 12:745945. <https://doi.org/10.3389/fimmu.2021.745945>

Xia J, Xie Z, Niu G et al (2023) Single-cell landscape and clinical outcomes of infiltrating B cells in colorectal cancer. Immunology 168:135-151. <https://doi.org/10.1111/imm.13568>

Yang W, Soares J, Greninger P et al (2013) Genomics of Drug Sensitivity in Cancer (GDSC): a resource for therapeutic biomarker discovery in cancer cells. Nucleic Acids Res 41:D955-961. <https://doi.org/10.1093/nar/gks1111>

Yi M, Zheng X, Niu M et al (2022) Combination strategies with PD-1/PD-L1 blockade: current advances and future directions. Mol Cancer 21:28. <https://doi.org/10.1186/s12943-021-01489-2>

Yu T, Tan H, Liu C et al (2022) Integratively Genomic Analysis Reveals the Prognostic and Immunological Characteristics of Pyroptosis and Ferroptosis in Pancreatic Cancer for Precision Immunotherapy. Front Cell Dev Biol 10:826879. <https://doi.org/10.3389/fcell.2022.826879>

Zhang B, Horvath S (2005) A general framework for weighted gene co-expression network analysis. Stat Appl Genet Mol Biol 4:Article17. <https://doi.org/10.2202/1544-6115.1128>

Zhou L, Xu B, Liu Y et al (2021) Tertiary lymphoid structure signatures are associated with survival and immunotherapy response in muscle-invasive bladder cancer. Oncoimmunology 10:1915574. <https://doi.org/10.1080/2162402X.2021.1915574>

**Legend of supplementary materials:**

**Figure S1**. The scRNA-seq of tumor-infiltrating B-cells in PDAC indicating the existence of TLSs and their relationship with CD8+ T-cell infiltration. (a) t-SNE projection of PDAC scRNA-seq data (GSA: CRA001160) annotated by cluster. (b) The t-SNE plot demonstrates the main cell types in PDAC. (c) The t-SNE plot demonstrates B-cell subtypes in PDAC. (d) The t-SNE plot demonstrates T-cell subtypes in PDAC. (e) The distribution of infiltrating T-cells and B-cells between different GC B-cell groups. (*p < 0.05; **p < 0.01; ***p < 0.001; ****p < 0.0001; Ns, not significant). scRNA-seq, single-cell RNA-sequencing; TLSs, tertiary lymphoid structures; t-SNE, t-distributed stochastic neighbor embedding; PDAC, pancreatic ductal adenocarcinoma; GC, germinal center.

**Figure S2. Diverse cell types in PDAC delineated by single-cell transcriptomic analysis.** (a) Box plots displaying the expression of representative well-known markers across the cell types identified in PDAC. (b) t-SNE projection of tumor-infiltrating B-cells in PDAC annotated by cluster. (c) Box plots displaying the expression of representative well-known markers of B-cell subtypes across clusters. (d) t-SNE projection of tumor-infiltrating B-cells in PDAC annotated by cluster. (e) Box plots displaying the expression of CD4 and CD8A in T-cells across clusters.

**Figure S3**. Identification of CTCRGs. (a) Clustering dendrograms of 14776 commonly expressed genes in the TCGA-PAAD cohort. (b) Analysis of scale-free fit index and mean connectivity for various soft-thresholding powers. (c) Clustering of module eigengenes. The red line shows the cut height (0.25). (d) Dendrogram of robust commonly expressed genes clustered based on a dissimilarity measure (1-TOM). (e) Heatmap of the correlation between module eigengenes and clinical traits of PDAC. Each cell contains a p-value and a correlation coefficient. (f) Scatter plot of module eigengenes related to the abundance of CD8+ T cells in the yellow module. CTCRGs, CD8+ T-cell related genes; TOM, topological overlap matrix.

**Figure S4. Stratifying the PDAC patients in the TCGA-PAAD cohort according to the expression profile of T-C genes and the validation of T-C score.** (a) The corresponding relative change in area under the cumulative distribution function (CDF) curves when cluster number changes from 2 to 5. The optimal k = 2. (b) Survival curves of patients in T-C cluster-1 and cluster-2. Survival analysis of the PDAC patients of ICGC-CA (c), E-MTAB-6134 (d), and the joint cohort of GSE71729 & GSE85916 (e) in Low and High T-C score groups (log-rank p < 0.001). Distribution of the T-C score and survival status of PDAC patients of ICGC-CA (f), E-MTAB-6134 (g), and the joint cohort of GSE71729 & GSE85916 (h). ROC curves for the 1-, 2-, 3-, 4-, and 5-year survival times based on the T-C score for PDAC patients of ICGC-CA (i), E-MTAB-6134 (j), and the joint cohort of GSE71729 & GSE85916 (k).

**Figure S5**. Stratifying the PDAC patients in the TCGA-PAAD cohort according to the expression profile of T-C genes and the mutational landscape between T-C clusters. TCGA slides showing a microscopic view of T-C cluster-1 patient TCGA slides showing a microscopic view of T-C cluster-1 patient (a) and T-C cluster-2 patient (b), with blue arrows showing TLSs and red arrows showing infiltrating immune cells. (c) the expression profile of GC B-cell marker genes (CD38, CD27) and T-C genes between the two T-C clusters. Box plot (d) and heatmap (e) display the results of the ssGSEA analysis utilizing 28 immune cells between the two T-C clusters. (f) The expression profile of 43 Immune Check Points (ICPs) related genes in PDAC between the two T-C clusters. (g) GSVA enrichment analysis of biological pathways between the two T-C clusters. The Amplifications, Deletions (h), and TMB (i) were evaluated between the T-C clusters. © (*p < 0.05; **p < 0.01; ***p < 0.001; ****p < 0.0001; Ns, not significant). TMB, tumor mutation burden.

**Figure S6**. Functional enrichment analysis between the T-C score groups and the correlation between the T-C score and TIME. (a) The GSVA analysis revealed significant enrichment in tumorigenesis-related pathways in the High T-C score group. Red bars indicate a high-scoring gene enrichment pathway and blue bars indicate a low-scoring gene enrichment pathway. (b) The ESTIMATE analysis demonstrated the T-C score was significantly and negatively correlated with the ESTIMATE Score, Immune Score, and Stromal Score. The low T-C score group had significantly higher levels of ESTIMATE score, Immune score, and Stromal score (c) A heatmap displaying the results of the ssGSEA analysis utilizing 28 immune cells between T-C score groups. (d) A CIBERSORTx Box plot demonstrated the infiltration levels of multiple immune cells between T-C score©oups. (e) Box plot Evaluating the expression levels of the ICPs-relevant genes between the distinct T-C score groups (*p < 0.05; **p < 0.01; ***p < 0.001; ****p < 0.0001; Ns, not significant). TIME, tumor immune microenvironment.

**Figure S7. Correlations between the T-C score and immune cell infiltration.** (a) Box plot displaying the results of the ssGSEA analysis utilizing 28 immune cells between the two T-C score groups of TCGA-PAAD. (b) Box plot displaying the differences in immune cell infiltration between T-C score groups of TCGA-PAAD using the xCELL algorithm. (c) Box plot displaying the differences in immune cell infiltration between T-C score groups of TCGA-PAAD using EPIC algorithm. (d) Box plot displaying the differences in immune cell infiltration between T-C score groups of TCGA-PAAD using the MCPcounter algorithm.

**Figure S8**. Correlations between the T-C score and tumor mutational profiles. The oncoplots depict the landscape of the 25 most frequently genomic altered genes in the Low (a) and High (b) T-C score groups. (c) A forest plot showed the mutated genes that were significantly different between the two groups. (d) The interactions among the mutated genes are shown in the oncoplots. Correlations between Amplification (e), Deletion (f), TMB (g), and T-C score. (*p < 0.05; **p < 0.01; ***p < 0.001).

**Figure S9. Correlations between the T-C score, tumor mutational landscape, and immune therapy.** The status of Amplification (a), Deletion (b), and TMB (c) between the distinct T-C score groups of TCGA-PAAD. (d) The correlation between TIDE score and T-C score in the ICGC-CA cohort. (e) The distribution of TIDE score between distinct T-C score groups in the ICGC-CA cohort. (f) Predicted clinical responses to the ICBs immunotherapy for patients of ICGC-CA cohort in the Low and High T-C score groups by TIDE analysis. (g) The correlation between TIDE score and T-C score in the E-MTAB-6134 cohort. (h) The distribution of TIDE score between distinct T-C score groups in the E-MTAB-6134 cohort. (i) Predicted clinical responses to the ICBs immunotherapy for patients of the E-MTAB-6134 cohort in the Low and High T-C score groups by TIDE analysis. (j) The correlation between TIDE score and T-C score in the GSE71729 & GSE85916 joint cohort. (k) The distribution of TIDE score between distinct T-C score groups in the GSE71729 & GSE85916 joint cohort. (l) Predicted clinical responses to the ICBs immunotherapy for patients of GSE71729 & GSE85916 joint cohort in the Low and High T-C score groups by TIDE analysis. (*p < 0.05; **p < 0.01; ***p < 0.001; ****p < 0.0001; Ns, not significant).

**Figure S10**. Relationship between T-C score and PDAC tumor stemness and the role of the P-F score in predicting the benefits from immunotherapy and chemotherapy. The correlations between DNAsi (a), RNAsi (b), and T-C score are displayed by scatter plot. (c) The distribution of DNAsi and RNAsi in distinct T-C score groups. (d) The correlation between TIDE score and T-C score in TCGA-PAAD cohort. (e) The distribution of TIDE score between distinct T-C score groups in TCGA-PAAD cohort. (f) The estimated clinical responses to the ICBs immunotherapy for patients of TCGA-PAAD cohort in the Low and High T-C score group by TIDE analysis. (g), (h), (i) The estimated IC50 levels of paclitaxel, gemcitabine, 5-fluorouracil, irinotecan, oxaliplatin, cisplatin, afatinib, lapatinib, gefitinib, sorafenib, olaparib, and vorinostat between the two T-C score groups. (*p < 0.05; **p < 0.01; ***p < 0.001; ****p < 0.0001; Ns, not significant). ICBs, immune checkpoint blockades.

**Figure S11**. CMap analysis based on the DEGs between the two T-C score groups. (a) DEGs between the two T-C score groups were identified by the ‘limma’ package. (b) heatmap depicting small-molecule compounds (columns) and their shared drug mechanisms of action (rows) through the CMap database.

**Figure S12. The workflow of the current study.**

**Table S1. TLSs Associated Genes and Their Median Expression Levels in Databases**

**Table S2. Gene Clusters Before Boruta**

**Table S3. TCGA-PAAD Clinical Features**

**Table S4. Connectivity MAP**
